# Orexins, Psychosis, and Antipsychotics: A Systematic Review of Studies of Orexin Levels and the Effects of Dual Orexin Receptor Antagonists (DORAs)

**DOI:** 10.3390/brainsci16040361

**Published:** 2026-03-27

**Authors:** Stella Margoni, Senad Hasaj, Guglielmo Donofrio, Georgios D. Kotzalidis, Tommaso Callovini, Mario Pinto, Camilla Scialpi, Matteo Bucci, Maria Benedetta Anesini, Gabriele Sani, Lorenzo Moccia, Delfina Janiri

**Affiliations:** 1 Department of Neurosciences, Section of Psychiatry, Università Cattolica del Sacro Cuore, 00168 Rome, Italy; stella.margoni98@gmail.com (S.M.); senadhasaj98@gmail.com (S.H.); guglielmodonofrio96@gmail.com (G.D.); giorgio.kotzalidis@gmail.com (G.D.K.); t.callovini@gmail.com (T.C.); mario.pinto@guest.policlinicogemelli.it (M.P.); camilla.scialpi@libero.it (C.S.); matteo.bucci01@icatt.it (M.B.); mbenedetta@hotmail.it (M.B.A.); or lorenzomoccia27@gmail.com (L.M.); delfina.janiri@unicatt.it (D.J.); 2 Department of Neurosciences, Head-Neck and Chest, Section of Psychiatry, Fondazione Policlinico Universitario Agostino Gemelli IRCCS, 00168 Rome, Italy

**Keywords:** schizophrenia, orexin/hypocretin system, antipsychotic drugs, sleep, dual orexin receptor antagonists, psychosis

## Abstract

**Highlights:**

**What are the main findings?**

- Dual orexin receptor antagonists interfere with dopaminergic transmission.
- Peripheral orexin levels are inconsistently altered in psychoses.

**What are the implications of the main findings?**

- Dual orexin receptor antagonists could potentially overcome some antipsychotic side effects, but this was not consistently shown in the psychoses.
- However, their use in schizophrenia still has to be confirmed.

**Abstract:**

**Background/Objectives**: Schizophrenia is a severe psychiatric disorder frequently characterised by sleep and circadian disturbances, which are closely linked to cognitive dysfunction, symptom exacerbation, and poor functional outcomes. A growing body of evidence implicates the orexin (hypocretin) system—an essential regulator of arousal, sleep–wake stability, metabolic processes, and motivated behaviour—in the pathophysiology and treatment response of psychotic disorders. We aimed to investigate the relationships between the orexinergic system and psychoses. **Methods**: On 3 March 2026, we searched the PubMed, Scopus, PsycInfo/Articles and Cinahl databases for studies dealing with the orexin system and psychotic disorders and treatment response. **Results**: We found 20 eligible studies reporting variable and inconsistent alterations in orexin signalling in patients with schizophrenia. Studies were mostly cross-sectional and heterogeneous in design. Antipsychotic medications interfere with orexin-dependent pathways, potentially contributing to both therapeutic effects and adverse outcomes such as sleep disruption and metabolic dysregulation. **Conclusions**: While evidence from preclinical studies could point to an influence of dopaminergic activity through orexinergic mechanisms, with possible attenuation of antipsychotic-induced motor side effects and improvement of attentional deficits associated with NMDA receptor hypofunction, the utility of dual orexin receptor antagonists (DORAs) in psychoses is unclear. Despite the high prevalence of insomnia in schizophrenia, its pharmacological management remains suboptimal, with current treatments often limited by reduced efficacy or tolerability concerns. DORAs, which are currently approved medications for the treatment of insomnia, represent a novel and mechanistically distinct therapeutic option that may improve sleep while modulating arousal- and cognition-related circuits relevant to psychosis.

## 1. Introduction

Schizophrenia is a chronic psychiatric disorder defined by persistent disturbances in perception, belief formation, motivation, and cognition. Although relatively infrequent at the population level, it contributes disproportionately to global disability, premature mortality, and socioeconomic burden [1]. Its onset typically occurs in early adulthood and often leads to lifelong functional impairment, placing substantial pressure on individuals, families, and healthcare systems [1].

Schizophrenia is usually characterised by negative symptoms, such as avolition, alogia, social withdrawal, anhedonia, and blunted affect mounting during the earlier phases of the disorder until a full-blown positive symptom outburst manifests; this is accompanied by cognitive symptoms, defective reasoning, logical leaps, bizarre behaviour, hygiene neglect, and disorganisation. Symptoms wax and wane and have an exacerbation-remitting course. Beyond its core psychopathological features, schizophrenia is associated with a broad range of medical and neurobiological comorbidities. Sleep–wake disturbances are highly prevalent and increasingly recognised not only as epiphenomena but as factors that can exacerbate symptom instability, cognitive dysfunction, and relapse risk [2,3]. Metabolic abnormalities represent another major contributor to morbidity and are strongly influenced by antipsychotic treatment, while cognitive deficits remain among the most robust predictors of long-term disability across illness stages [4]. These clinical domains—sleep, metabolism, arousal, and cognition—are closely intertwined and are all influenced by neurochemical systems targeted, directly or indirectly, by antipsychotic medications.

Within this broader physiological landscape, the orexin (hypocretin) neuropeptides have attracted increasing attention. Produced in the lateral hypothalamus, orexin-A and orexin-B regulate arousal, sleep architecture, appetite, autonomic tone, and reward-motivated behaviour [5]. They exert their effects through two G protein-coupled receptors, OX1R and OX2R, distributed across key neural circuits underlying vigilance, emotional regulation, and executive functioning [6]. Dysregulation of orexin signalling has been implicated across several psychiatric disorders, and accumulating evidence suggests that this system may contribute to symptom expression and treatment response in schizophrenia [7].

Importantly, orexinergic circuits overlap extensively with dopaminergic pathways that are central to both the pathophysiology of psychosis and the mechanism of action of antipsychotic drugs. Orexin neurons directly modulate midbrain dopamine neuron firing, influencing salience attribution, behavioural activation, and cognitive control [8,9,10]—processes that are disrupted in schizophrenia and pharmacologically targeted by antipsychotics [11,12]. In parallel, experimental studies indicate that the manipulation of orexin receptors can modify dopaminergic tone and behavioural outcomes relevant to antipsychotic efficacy and tolerability. For example, orexin-1 receptor antagonism has been shown to attenuate antipsychotic-induced catalepsy in preclinical models, suggesting a role for orexin signalling in extrapyramidal side effects [13].

Clinical evidence further supports a bidirectional interaction between antipsychotic treatment and the orexin system. Several studies have reported alterations in peripheral and central orexin-A levels in individuals with schizophrenia, with differences observed between drug-free and antipsychotic-treated patients [14]. Notably, elevations in circulating orexin-A have been associated with antipsychotic exposure independent of metabolic status, indicating a potential direct pharmacological effect [15,16]. Genetic variation in orexin receptors has also been linked to susceptibility to antipsychotic-induced weight gain, further implicating this system in treatment-related adverse outcomes [17]. Together, these findings suggest that orexin signalling may act as a dynamic modulator of both therapeutic and adverse effects of antipsychotic medications rather than as a static disease marker.

Despite these converging lines of evidence, the role of the orexin system in mediating antipsychotic effects has not been systematically synthesised. While orexin receptor antagonists—particularly dual orexin receptor antagonists (DORAs)—are now established treatments for insomnia and have shown promise in psychosis-relevant preclinical models [9], their relevance to schizophrenia must be understood within the broader framework of antipsychotic–orexin interactions. Clarifying how antipsychotic drugs influence orexin signalling, and how this interaction relates to sleep regulation, metabolic burden, cognitive outcomes, and motor side effects, is essential for evaluating the translational potential of orexin-targeting strategies.

The present review therefore aims to systematically examine the evidence linking antipsychotic treatment to alterations in the orexin/hypocretin system in schizophrenia and related psychotic disorders. By integrating clinical and preclinical findings, we seek to elucidate the mechanistic basis and clinical implications of antipsychotic–orexin interactions and identify key gaps that should be addressed in future translational and clinical research.

## 2. Materials and Methods

To identify articles investigating the relationship between orexins and antipsychotic treatment we used the following strategy on PubMed on 3 March 2026: (schizophr* OR psychosis OR psychotic OR paranoi* OR schizoaffect* OR antipsychotic* OR neuroleptic* OR phenothiazine* OR butyrophenon* OR “dopamine antagonist*” OR “dopamine receptor antagonist*” OR “substituted benzamide*” OR thioxanthene* OR dibenzoazepine* OR benzisoxazole* OR haloperidol OR chlorpromazine OR promazine OR thioridazine OR clothiapine OR loxapine OR clozapine OR quetiapine OR olanzapine OR fluperlapin* OR zotepine OR remoxipride OR sertindole OR risperidone OR paliperidone OR aripiprazole OR brexpiprazole OR cariprazine OR ziprasidone OR asenapine OR bromperidol OR spiroperidol OR spiperone OR perphenazine OR fluphenazine OR flupentixol* OR clopenthixol OR zuclopenthixol OR droperidol OR thioxanthene OR sulpiride OR sultopride OR metoclopramide OR amisulpride) AND (orexin* OR hypocretin* OR Hcrts OR OX1 OR OX2 OR OX1R OR OX2R OR suvorexant OR MK-4305 OR lemborexant OR daridorexant OR almorexant OR ACT-078573 OR filorexant OR MK-6096 OR seltorexant OR MIN-202 OR JNJ-42847922 OR JNJ-922 OR Fazamorexant OR YZJ-1139 OR Nivasorexant OR ACT-539313 OR Tebideutorexant OR JNJ-61393215 OR JNJ-3215 OR Vornorexant OR ORN-0829 OR TS-142 OR ACT-335827 OR EMPA OR GSK-649868 OR SB-649868 OR JNJ-10397049 OR RTIOX-276 OR SB-334867 OR SB-408124 OR TCS-OX2-29). This strategy was adapted to suit the requirements of the other databases that we consulted, in other words, Scopus (TITLE-ABS-KEY (schizophrenia OR schizophreniform OR psychosis OR psychotic OR paranoid OR schizoaffective OR antipsychotic OR neuroleptic OR phenothiazine OR butyrophenone OR dopamine antagonist OR dopamine receptor antagonist OR substituted benzamide OR thioxanthene OR dibenzoazepine OR benzisoxazole OR haloperidol OR chlorpromazine OR promazine OR thioridazine OR clothiapine OR loxapine OR clozapine OR quetiapine OR olanzapine OR fluperlapine OR zotepine OR remoxipride OR sertindole OR risperidone OR paliperidone OR aripiprazole OR brexpiprazole OR cariprazine OR ziprasidone OR asenapine OR bromperidol OR spiroperidol OR spiperone OR perphenazine OR fluphenazine OR flupentixol OR clopenthixol OR zuclopenthixol OR droperidol OR thioxanthene OR sulpiride OR sultopride OR metoclopramide OR amisulpride) AND TITLE-ABS-KEY (orexin OR orexins OR hypocretin OR hypocretins OR Hcrts OR OX1 OR OX2 OR OX1R OR OX2R OR suvorexant OR MK-4305 OR lemborexant OR daridorexant OR almorexant OR ACT-078573 OR filorexant OR MK-6096 OR seltorexant OR MIN-202 OR JNJ-42847922 OR JNJ-922 OR Fazamorexant OR YZJ-1139 OR Nivasorexant OR ACT-539313 OR Tebideutorexant OR JNJ-61393215 OR JNJ-3215 OR Vornorexant OR ORN-0829 OR TS-142 OR ACT-335827 OR EMPA OR GSK-649868 OR SB-649868 OR JNJ-10397049 OR RTIOX-276 OR SB-334867 OR SB-408124 OR TCS-OX2-29)) and PscINFO/PsycARTICLES as well as CINAHL (schizophr* OR psychosis OR psychotic OR paranoi* OR schizoaffect* OR antipsychotic* OR neuroleptic* OR phenothiazine* OR butyrophenon* OR “dopamine antagonist*” OR “dopamine receptor antagonist*” OR “substituted benzamide*” OR thioxanthene* OR dibenzoazepine* OR benzisoxazole* OR haloperidol OR chlorpromazine OR promazine OR thioridazine OR clothiapine OR loxapine OR clozapine OR quetiapine OR olanzapine OR fluperlapin* OR zotepine OR remoxipride OR sertindole OR risperidone OR paliperidone OR aripiprazole OR brexpiprazole OR cariprazine OR ziprasidone OR asenapine OR bromperidol OR spiroperidol OR spiperone OR perphenazine OR fluphenazine OR flupentixol* OR clopenthixol OR zuclopenthixol OR droperidol OR thioxanthene OR sulpiride OR sultopride OR metoclopramide OR amisulpride) AND (orexin* OR hypocretin* OR Hcrts OR OX1 OR OX2 OR OX1R OR OX2R OR suvorexant OR MK-4305 OR lemborexant OR daridorexant OR almorexant OR ACT-078573 OR filorexant OR MK-6096 OR seltorexant OR MIN-202 OR JNJ-42847922 OR JNJ-922 OR Fazamorexant OR YZJ-1139 OR Nivasorexant OR ACT-539313 OR Tebideutorexant OR JNJ-61393215 OR JNJ-3215 OR Vornorexant OR ORN-0829 OR TS-142 OR ACT-335827 OR EMPA OR GSK-649868 OR SB-649868 OR JNJ-10397049 OR RTIOX-276 OR SB-334867 OR SB-408124 OR TCS-OX2-29)). All database searches were performed on the same date.

Inclusion/exclusion criteria. Included were studies investigating the orexin/hypocretin system in relation to antipsychotic treatment in patients with schizophrenia or related psychotic disorders. Eligible studies examined orexin signalling, orexin receptors, or orexin receptor antagonists in clinical or translational contexts relevant to psychosis, cognition, sleep–wake regulation, metabolic effects, or antipsychotic response. Clinical studies were considered when directly addressing mechanisms pertinent to psychotic disorders but used preclinical evidence addressing these issues as a narrative support to our data. Excluded were records not meeting these criteria and evidence published in non-peer-reviewed sources. Dissertations and theses were included only if peer reviewed or endorsed by established experts. Additional exclusion criteria comprised studies with no or incomplete data; those not including psychotic disorders (labelled “no psychosis”); those not reporting data on the orexin/hypocretin system (labelled “no orexin”); studies unrelated to the topic (“unrelated”); animal studies not providing clear translational relevance (“animal”); reviews including meta-analyses and consensus guidelines (“review”); case reports or case series (“case”); editorials, viewpoints, and letters without original data (“opinion”); duplicate publications (“duplicate”); studies based on overlapping samples (“overlap”); studies not reporting on the chosen outcomes (i.e., orexin levels or orexin antagonist effects) were labelled “unfocused”, while those reporting on heterogeneous patient populations, including psychoses, but not reporting results separately for the psychoses were termed “lumping” (i.e., they lumped the results of patients with other than psychotic diagnoses with those of patients with a psychosis), and conference abstracts that were not subsequently published in peer-reviewed journals (termed “Abstract”).

Eligibility was determined with full consensus for labelling each article, which was obtained from all authors who met in Delphi rounds to decide. No more than three were needed to reach complete consensus. Included and excluded records are shown in Supplementary Table S1, along with the specific reasons for exclusion. All authors were involved in the search strategy and in the article selection. Authors independently searched all involved databases and compared their results thereafter. The results obtained by all authors reciprocally overlapped.

**Figure 1:**
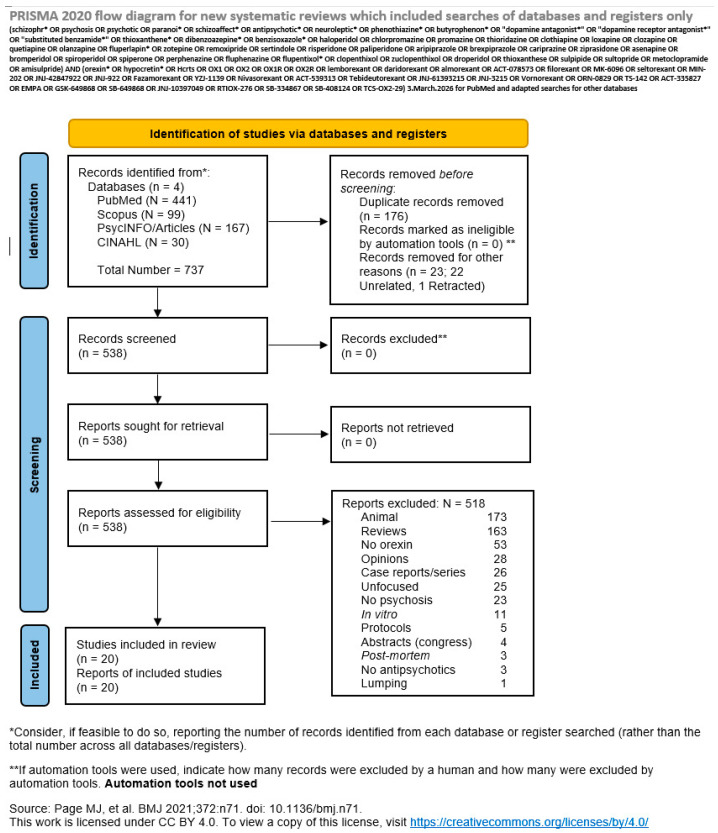
The PRISMA flow diagram of our search. After Page et al. (2021) [18].

The review adhered to the Preferred Reporting Items for Systematic reviews and Meta-Analyses (PRISMA) statement [18]; the results obtained are shown in Figure 1, while the CheckList is shown in the Supplementary Materials. The risk of bias of the included studies was assessed using the Cochrane Risk Of Bias In Non-randomised Studies-of Exposures (ROBINS-E) tool [19]. The results of the evaluation for all studies is shown in the Supplementary Materials. We registered our review on the Open Science Framework (OSF) platform with the following digital object identifier; https://doi.org/10.17605/OSF.IO/WBFCE.

## 3. Results

The above search on 3 March 2026 yielded 441 records in PubMed, 167 articles in PsycINFO/PsycARTICLES, 30 in CINAHL Plus, and 99 in Scopus for a grand total of 737 articles. The cumulative results with the reasons for exclusion are shown in Figure 1 and Supplementary Table S1. Twenty studies were eligible. Of the records resulting from our searches, 173 were animal studies, 163 were reviews, 53 did not refer to orexin or hypocretin, 28 were opinions (editorials, letters to the editor, perspectives, theoretical papers and so forth), 26 were case reports or series, 25 were unfocused (i.e., despite meeting the orexin and antipsychotic/psychoses requirement, they did not report on outcomes that were our aim to explore), 23 made no reference to psychosis, 22 were unrelated to the subject matter (i.e., they referred to neither orexins or psychoses or antipsychotic drugs), 11 were performed in vitro only, 5 were protocols with no data, 4 were congress abstracts with insufficient data, 3 were *post-mortem* studies, 3 contained no data on antipsychotics, 1 lumped the data, and 1 was retracted (an animal study). There were 176 duplicates among the databases. The first study emerging from the search was published in 1976 (and was found on PubMed but had nothing to do with orexins/hypocretins), and the last two appeared on PsycINFO/PsycARTICLES on 4 February 2026 and on PubMed on 11 February 2026. The first included paper was published on 15 May 2002, the last on 30 November 2025. The selection process is illustrated in Figure 1 and reported in detail in the Supplementary Materials, Table S1. Country details and study typology as well as population included are reported in Supplementary Table S2. Supplementary Table S3 reports the risk of bias for each included study, while Supplementary Table S4 is the PRISMA checklist [18]. In particular, the characteristics of the included studies are shown in Supplementary Table S2. Of the 20 eligible studies, 9 (45.0%) were cross-sectional, 4 (20.0%) were longitudinal, 5 (25.0%) were case–control studies (including cross-sectional case–control designs), and 2 (10.0%) were observational with a non-specified design. Most studies were single-site (*n* = 18, 90.0%), while two studies (10.0%) were multicentre. The included studies were conducted across Asia, Europe, and North America. The largest contributions came from Taiwan (25.0%), followed by China and Japan (each 20.0%), and multicentre studies (10.0%). Single-country studies were conducted in the United States, Germany, France, Turkey, and Spain (each 5.0%). Across the 20 included studies, a total of 3495 participants were examined, including 56 post-mortem samples. Of these, 2642 participants (75.6%) had a diagnosis of schizophrenia or a psychotic-spectrum disorder including 150 first-episode patients (5.7% of the psychosis sample).

The overall sample also comprised 139 neurological patients with Guillain–Barré syndrome presenting with mental status abnormalities, 55 neurological ICU controls without GBS, 80 patients with major depressive disorder, 40 patients with bipolar disorder, and 657 healthy controls (18.8% of the total sample). Regarding antipsychotic exposure, clozapine was included in 11 studies (55.0%), haloperidol in 8 studies (40.0%), and olanzapine in 6 studies (30.0%). Additional antipsychotics—including other second-generation agents, conventional neuroleptics, aripiprazole, or mixed treatment regimens—were reported in 10 studies (50.0%). Antipsychotic-free or drug-naïve samples were included in two studies (10.0%), while antipsychotic treatment was not specified in four studies (20.0%). Orexin assessment was predominantly based on peptide measurements, with orexin-A/hypocretin-1 quantified in 16 studies (80.0%), whereas orexin-B or total orexin was assessed in one study (5.0%). Investigations at the receptor level—including genetic or mRNA analyses of HcrtR1/HcrtR2—were conducted in five studies (25.0%), with four studies (20.0%) specifically examining genetic polymorphisms. Measurements were performed mainly in plasma or serum (11 studies, 55.0%) and cerebrospinal fluid (8 studies, 40.0%), while post-mortem brain tissue was examined in one study (5.0%). Radioimmunoassay (RIA) and enzyme-linked immunosorbent assay (ELISA) were the most commonly used analytical methods (35.0% and 40.0%, respectively), whereas four studies (20.0%) relied exclusively on genetic approaches without peptide quantification. With respect to the direction of reported associations, antipsychotic-related modulation of the orexin/hypocretin system was described in 12 studies (60.0%), most commonly as reduced orexin levels during treatment (7 studies, 35.0%). Increased orexin levels in specific contexts—most notably among clozapine responders—were reported in three studies (15.0%), while circadian or rhythmic modulation without changes in mean orexin levels was described in two studies (10.0%). Most studies found no significant global associations between orexin measures and psychotic symptom severity (12 studies, 60.0%), whereas favourable symptom associations emerged only in specific subgroups (5 studies, 25.0%). In contrast, metabolic and behavioural outcomes were more consistently linked to orexin measures, with associations reported for metabolic parameters in 8 studies (40.0%) and for sleep–wake regulation or physical activity in 5 studies (25.0%) (Table 1). All counts are reported in the Supplementary Materials.

## 4. Discussion

1. Synthesis of the main findings

The present review synthesised the available evidence on the involvement of the orexin/hypocretin system in psychotic disorders, with particular attention to its modulation by antipsychotic treatment. Overall, the findings indicate that the orexinergic system is altered in psychosis and is responsive to antipsychotic exposure, supporting its role at the interface between arousal regulation, metabolic homeostasis, and treatment response. Alterations of the orexin system have been reported across multiple biological levels, including peripheral measures, cerebrospinal fluid, post-mortem brain tissue, and genetic variants [14,20,21,22], suggesting system-wide dysregulation rather than compartment-specific effects. Importantly, several studies indicate that orexin alterations are dynamically modulated by antipsychotic treatment, as shown by differences between drug-naïve, antipsychotic-free, and treated patients as well as by longitudinal changes following treatment initiation [15,16,21]. Nevertheless, substantial heterogeneity characterises the literature. While some studies reported reduced orexin or hypocretin levels, particularly in relation to specific antipsychotics or central measures [14,15,21], others describe elevated peripheral orexin-A levels in subgroups of patients, often associated with more favourable clinical, cognitive, or metabolic profiles [26,30,34]. Additional findings point to alterations in circadian or state-dependent regulation rather than uniform changes in absolute orexin levels [27,35]. This variability likely reflects differences in study design, patient characteristics, antipsychotic exposure, and individual factors such as sex and metabolic status [14,26,31].

2. Orexin in psychosis: primary alteration versus pharmacological modulation

Evidence from first-episode and drug-naïve samples suggests that orexin-A levels may be reduced early in the course of psychosis, indicating a possible initial hypofunction of the orexinergic system prior to pharmacological exposure [14,15,33]. In contrast, studies in chronic or antipsychotic-treated patients more frequently report normal or increased orexin-A levels, particularly in clinically stable or treatment-responsive individuals [16,26,30,34]. This pattern supports the interpretation of orexin dysregulation as a state-dependent phenomenon, with early alterations potentially followed by adaptive changes related to illness progression and treatment. Against this background, examining the direct effects of different antipsychotic compounds on the orexinergic system becomes essential for disentangling primary pathophysiological mechanisms from secondary pharmacological modulation.

3. Effects of antipsychotics on the orexinergic system

The available evidence indicates that antipsychotic drugs directly modulate the orexinergic system, with effects that vary across compounds. Early cerebrospinal fluid studies showed reduced orexin-A levels in patients treated with haloperidol compared with unmedicated individuals, suggesting a suppressive effect associated with strong dopamine D_2_ antagonism [21]. A similar reduction in peripheral orexin-A levels has been observed following short-term olanzapine treatment in first-episode psychosis, independent of weight gain [15]. In contrast, clozapine appears to exert a distinct effect. Higher orexin-A levels have been reported in clozapine-treated patients, particularly among those showing a favourable clinical response, whereas lower levels characterise clozapine-resistant or antipsychotic-free patients [16,34]. Beyond changes in absolute levels, antipsychotics may also affect the temporal regulation of the orexin system. Evidence from chronobiological and longitudinal studies indicates that clozapine is associated with alterations in diurnal rhythms and sleep–wake-related orexin dynamics, rather than stable shifts in baseline concentrations [27,35]. Overall, these patterns support a distinction between typical and atypical antipsychotics and highlight the relevance of receptor-binding profiles. Agents with predominant D_2_ antagonism tend to suppress orexin signalling, whereas clozapine, with its broader pharmacological profile, may engage orexin pathways through non-dopaminergic mechanisms. Genetic and pharmacogenetic findings further support an interaction between orexin receptors and antipsychotic effects [17,22].

4. Orexin, sleep, and arousal: a central node

Alterations in sleep–wake regulation and diurnal rhythms are consistently reported in psychosis and appear closely linked to dysregulation of the orexinergic system. Several studies suggest that orexin signalling is more strongly associated with arousal, sleep architecture, and circadian organisation than with core psychotic symptoms, highlighting its role as a functional bridge between psychosis-related vulnerability, insomnia or sedation, and antipsychotic side effects [20,27,35]. By modulating arousal rather than psychosis per se, orexin pathways may contribute to clinically relevant dimensions often affected by antipsychotic treatment including daytime sleepiness, fatigue, and behavioural activation. In this context, targeting the orexin system—particularly through dual orexin receptor antagonists—may represent a rational add-on strategy to address sleep and arousal disturbances in psychotic disorders, without directly interfering with dopaminergic mechanisms.

5. Orexin, Metabolism, and Weight: Marker Or Mechanism?

Several studies report a positive association between higher body mass index and elevated orexin-A levels in patients with schizophrenia, suggesting a link between orexin signalling and metabolic status rather than psychotic symptom severity [30,31]. Notably, these associations often persist after controlling for metabolic syndrome, indicating that orexin alterations may not simply reflect cardiometabolic pathology [16]. This pattern raises the possibility that increased orexin activity represents a compensatory response to metabolic challenge or antipsychotic exposure, rather than a primary driver of weight gain. The relative dissociation between metabolic parameters and core psychotic symptoms further supports a modulatory role of orexin in peripheral energy balance [36].

6. Orexin, Cognition, and Treatment Response

Higher orexin-A levels have been associated with better cognitive performance and more favourable clinical profiles in patients with psychosis, particularly in domains related to executive function and working memory [26,34]. In clozapine-treated samples, elevated orexin-A levels consistently characterise treatment responders compared with non-responders or antipsychotic-free patients, suggesting a link between orexin signalling and therapeutic efficacy [16,34,37]. These findings support the view of orexin-A as a potential biomarker of treatment response and as an indicator of a biologically distinct subgroup of patients.

7. Heterogeneity and Biological Subtypes

Genetic evidence implicating orexin receptor variants, particularly HcrtR1, together with reports of bimodal distributions of circulating orexin-A levels, supports the presence of biologically distinct subgroups within psychotic disorders [22,24,26]. These findings suggest that inter-individual variability in orexin signalling may reflect differential vulnerability rather than disease severity *per se*. Conceptualising psychosis as a heterogeneous condition encompassing orexin-related subtypes may help explain variability in clinical presentation and treatment response, aligning with precision medicine approaches.

8. Clinical and Translational Implications

The emerging evidence raises the question of whether orexin-related measures could be clinically informative as biomarkers of treatment response, metabolic vulnerability, or functional outcomes in psychosis. The sensitivity of the orexinergic system to antipsychotic exposure provides a rationale for exploring selective modulation of this pathway while recognising that the current data remain insufficient to support routine clinical implementation. Given the central role of orexin in arousal regulation, the use of dual orexin receptor antagonists in psychotic disorders should be approached with caution, particularly in the context of sedation and symptom exacerbation. Potential pharmacological interactions, especially with antipsychotics such as clozapine that appear to engage orexin pathways, warrant further investigation. Addressing these translational questions will require dedicated longitudinal and interventional studies.

9. Limitations, Strengths and Future Perspectives

Most available studies are cross-sectional, limiting causal inference on the relationship between antipsychotic treatment, orexin/hypocretin alterations, and clinical outcomes. Sample sizes are often small and clinically heterogeneous, and the frequent reliance on peripheral orexin-A measures may only indirectly reflect central orexinergic function. Confounding factors such as antipsychotic type and dose, illness stage, sleep–wake disturbances, and metabolic status are not consistently controlled. A further shortcoming was the risk of bias of the eligible studies. Overall bias was low in seven out of twenty studies, but only four achieved a “clean sheet” (i.e., they were low on all domains) (Supplementary Table S3).

A strength of this literature is the convergence of clinical, genetic, and preclinical findings supporting the involvement of the orexin system in schizophrenia and antipsychotic response. Preclinical search, although not considered in this review, may help better conceptualise the issue dealt with here. In fact, the administration of antipsychotics increases the preproorexin and orexin levels in rats [38], and symmetrically, the administration of DORAs counteracts the activity of antipsychotics on the dopaminergic activity in the substantia nigra and the ventral tegmental area of the anaesthetised rat [39]. Currently, there is not much consistence in the conclusions of clinical and animal studies. Future research should adopt longitudinal and experimental designs, integrate central and peripheral biomarkers, and systematically account for metabolic and sleep-related variables. Multimodal approaches combining neuroendocrine, cognitive, and neuroimaging data may be essential to clarify the mechanistic and clinical significance of antipsychotic–orexin interactions.

## 5. Conclusions

This review highlights the orexin/hypocretin system as a key transdiagnostic pathway linking psychosis, antipsychotic treatment, and core domains of arousal, sleep–wake regulation, metabolism, cognition, and treatment response. Rather than reflecting a simple deficit, orexinergic alterations appear dynamic and state-dependent, shaped by illness stage and pharmacological modulation, particularly by antipsychotics with distinct receptor profiles. From a clinical perspective, these findings suggest that orexin signalling may represent both a biomarker of therapeutic response—especially to clozapine—and a mechanistic contributor to treatment-related benefits and adverse effects. Integrating orexin-focused biomarkers with clinical, cognitive, and metabolic assessments may advance a more nuanced, neurobiologically informed model of psychosis treatment, in which the modulation of arousal and circadian systems complements dopaminergic strategies and opens new avenues for personalised and adjunctive interventions.

## Figures and Tables

**Table 1:** Summary of studies investigating the relationship between orexins and antipsychotic treatment in chronological order.

Study	Population	Design	Instruments and Measures	Results and Conclusions
Nishino et al., 2002 [20]	25 male veterans were included: 13 psychiatric inpatients with schizophrenia spectrum disorders (11 schizophrenia, 2 schizoaffective disorder) and 12 non-psychiatric HC. Mean age was approximately 33 years in both groups. Patients were medication-free for at least two weeks (six months for depot neuroleptics) prior to assessments	Cross-sectional observational study	CSF hypocretin-1 levels were measured using radioimmunoassay following lumbar puncture. Sleep architecture was assessed with overnight polysomnography, including EEG, EOG, and EMG recordings scored according to standard criteria. Clinical diagnoses were established using structured diagnostic interviews.	CSF hypocretin-1 levels did not differ significantly between patients with schizophrenia and healthy controls. Patients showed longer sleep onset latency, reduced sleep efficiency, and altered sleep architecture compared with controls, but these sleep abnormalities were not associated with CSF hypocretin-1 concentrations. The authors conclude that, unlike narcolepsy, schizophrenia is not characterised by hypocretin deficiency, and that sleep disturbances in schizophrenia are unlikely to be directly mediated by altered hypocretin signalling.
Dalal et al., 2003 [21]	27 patients with schizophrenia diagnosed according to DSM-IV criteria: 7 treated with haloperidol, 8 treated with atypical antipsychotics (clozapine or olanzapine), and 12 unmedicated patients, of whom 6 were drug-naïve	Cross-sectional observational study	Cerebrospinal fluid orexin-A concentrations were measured using radioimmunoassay following standardised morning lumbar puncture. Demographic variables, body mass index, antipsychotic dose, duration of treatment, and duration of drug-free period were recorded at the time of sampling.	Patients with schizophrenia treated with haloperidol showed significantly lower CSF orexin A levels compared with both unmedicated patients and healthy controls. No significant differences were observed between unmedicated patients and controls. The authors conclude that reduced orexin A levels are likely related to antipsychotic treatment rather than to schizophrenia per se, suggesting a pharmacological modulation of the orexin system by dopamine D_2_ receptor blockade. These findings support a role for antipsychotic–orexin interactions in the regulation of arousal and sleep–wake processes in schizophrenia.
Meerabux et al., 2005 [22]	168 patients with DSM-IV schizophrenia were recruited and subdivided into a PHS subgroup and a non-polydipsic schizophrenia subgroup + 80 healthy controls. All participants were of Japanese ancestry. Polydipsia–hyponatremia status was defined using clinical history, water intake behaviour, and laboratory sodium measurements	Genetic association study	Genotyping of polymorphisms in the gene, prepro-orexin, *HcrtR1*, and *HcrtR2* genes using PCR-based methods and direct sequencing; clinical classification of polydipsia–hyponatremia status.	No association was found between the ACE insertion/deletion polymorphism and polydipsic schizophrenia. A missense variant in the orexin receptor 1 gene (*HcrtR1* 408Ile→Val) showed a significant genotypic and allelic association with polydipsia–hyponatremia in patients with schizophrenia, with higher prevalence in polydipsic compared with non-polydipsic patients. Functional assays suggested no major alterations in receptor localisation or calcium signalling. The findings suggest that genetic variation in the orexin system may increase vulnerability to specific clinical complications of schizophrenia, supporting a modulatory role of orexin signalling in psychosis-related phenotypes, particularly those involving dopaminergic and homeostatic dysregulation.
Cochen et al., 2005 [23]	139 consecutive patients with definite GBS admitted to a tertiary care hospital between 1990 and 2004 (age range 16–84 years). A subgroup of 55 ICU patients was assessed in detail, and 13 GBS patients plus 6 ICU neurological controls without GBS underwent polysomnographic recordings. CSF hypocretin-1 levels were measured in a subset of 20 GBS patients (9 with and 11 without mental status abnormalities)	Prospective observational cohort study	Mental status abnormalities (vivid dreams, illusions, hallucinations, delusions) were assessed through daily structured clinical interviews and bedside observation. Sleep architecture was evaluated using full polysomnography and EEG recordings. CSF hypocretin-1 concentrations were measured by radioimmunoassay. Clinical severity, autonomic dysfunction, ICU admission, and biological parameters (including CSF protein levels) were also recorded.	Mental status abnormalities occurred in 43 of 139 GBS patients (31%), significantly more frequently than in ICU controls (16%). These symptoms were strongly associated with disease severity, autonomic dysfunction, and ICU admission. Patients with mental status abnormalities showed significantly lower CSF hypocretin-1 levels compared with GBS patients without such symptoms. Polysomnography revealed profound REM sleep dysregulation, including REM sleep intrusions into wakefulness and non-REM sleep, particularly in patients with hallucinations and vivid dreams. Mental status abnormalities and sleep disturbances were reversible and resolved in parallel with clinical recovery, suggesting a transient dysfunction of orexinergic regulation of arousal rather than a primary psychiatric disorder.
Fukunaka et al., 2007 [24]	312 Japanese inpatients with schizophrenia diagnosed according to DSM-IV criteria. Of these, 65 patients met criteria for primary polydipsia with hyponatremia, while 247 patients with schizophrenia without polydipsia served as psychiatric controls. All participants were chronically ill, had been hospitalised for more than three years, and had stable laboratory follow-up. Subjects with major neurological disorders, substance abuse, or significant medical causes of hyponatremia were excluded	Case–control genetic association study	Polydipsia was defined clinically by excessive fluid intake (>3 L/day) accompanied by serum sodium levels below 135 mEq/L, confirmed through medical records and psychiatric assessment. Genomic DNA was extracted from peripheral blood samples, and the *HcrtR1* Ile408Val (rs2271933) polymorphism was genotyped using TaqMan allelic discrimination assays. Demographic variables, illness duration, antipsychotic dose (haloperidol equivalents), smoking status, and age at onset were recorded.	Genotype and allele frequencies of the HCRTR1 Ile408Val polymorphism differed significantly between polydipsic and non-polydipsic patients. The Ile allele was significantly more frequent in patients with polydipsia compared to controls, indicating an association between the HCRTR1 variant and susceptibility to polydipsia–hyponatremia in schizophrenia. No significant differences were observed in current antipsychotic dosage between groups, suggesting that the genetic association was not driven by medication exposure. The authors conclude that variation in the orexin 1 receptor gene may contribute to vulnerability to dysregulated drinking behaviour in schizophrenia, supporting a role of the orexin system in the pathophysiology of polydipsia beyond dopaminergic mechanisms alone.
Basoglu et al., 2010 [15]	20 male first-episode, drug-naïve, non-obese patients with psychosis (DSM-IV schizophrenia criteria except illness duration), aged approximately 21 years, hospitalised for 6 weeks and treated with olanzapine. 22 age-matched healthy male controls without psychiatric or medical disorders were included	Prospective longitudinal observational study	Body mass index, waist circumference, fasting glucose, lipid profile (LDL, HDL, triglycerides) measured at baseline, week 2, and week 6. Plasma leptin, ghrelin, orexin-A, cholecystokinin, agouti-related protein, and visfatin measured at baseline and week 6 using ELISA. Psychopathology assessed with the PANSS at baseline, week 2, and week 6.	After 6 weeks of olanzapine treatment, BMI, waist circumference, LDL-C, triglycerides, and leptin levels increased significantly, while ghrelin and orexin-A levels decreased significantly. BMI increased by more than 7% in 75% of patients. No significant changes were observed for cholecystokinin, agouti-related protein, visfatin, fasting glucose, or HDL-C. Changes in orexin-A were not directly correlated with BMI changes, suggesting that olanzapine-induced reductions in orexin-A may reflect central neuropeptide modulation rather than a simple consequence of weight gain. PANSS total, positive, negative, and general psychopathology scores improved significantly over the treatment period.
Huang et al., 2014 [25]	Paediatric/adolescent sample from Taiwan; with secondary schizophrenia (*n* = 10); Control groups: N–C without schizophrenia (*n* = 37) and schizophrenia without N–C (*n* = 13)	Case–control, observational, longitudinal follow-up	Clinical diagnosis of narcolepsy and schizophrenia based on standardised criteria; assessment of psychotic symptoms and clinical course. Orexin/hypocretin system evaluated indirectly through HLA typing (DQB1 alleles) and genetic analysis of orexin receptor genes (HcrtR1 and HcrtR2). No direct measurement of orexin-A/hypocretin-1 levels in CSF or plasma.	Schizophrenia developed after narcolepsy onset in a subset of patients and was characterised by persistent psychotic symptoms with limited response to antipsychotic treatment. No significant differences in orexin receptor gene variants were found between groups. The findings suggest that psychosis in narcolepsy is not explained by direct orexin receptor abnormalities, supporting an indirect or modulatory role of hypocretin system dysfunction rather than a primary orexin-deficiency mechanism in schizophrenia.
Chien et al., 2015 [26]	127 chronic, clinically stable patients with DSM-IV-diagnosed schizophrenia receiving antipsychotic treatment, and 34 age- and sex-matched healthy control subjects. Patients with neurological disorders, substance use, or major medical comorbidities were excluded	Cross-sectional observational study	Plasma orexin A levels measured by radioimmunoassay. Psychopathology assessed using the PANSS, including factor-derived subscores for positive, negative, disorganised, excitement, and anxiety/depressive symptoms. Executive function evaluated with the WCST. Antipsychotic treatment was categorised according to weight-gain liability and expressed in chlorpromazine-equivalent doses; smoking status was recorded and controlled for in analyses.	Mean plasma orexin A levels were significantly higher in patients with schizophrenia compared with healthy controls. Orexin levels in patients showed a bimodal distribution, identifying a subgroup (approximately 20%) with markedly elevated orexin A levels. This high-orexin subgroup exhibited significantly fewer negative and disorganised symptoms and trends toward better cognitive flexibility on the WCST compared with patients with normal orexin levels. Plasma orexin A levels were negatively correlated with negative and disorganised symptom severity. No significant differences in orexin levels were observed across antipsychotic groups with different weight-gain liabilities. The authors conclude that elevated orexin A may characterise a biologically distinct subgroup of patients with schizophrenia and could represent a favourable prognostic marker related to symptom profile and executive functioning, rather than a direct effect of antipsychotic medication.
Sun et al., 2016 [27]	13 male patients with first-episode schizophrenia and 15 age- and sex-matched healthy male controls were included. Patients were recruited from a military hospital in China and met DSM-IV criteria for schizophrenia. All patients were antipsychotic-naïve at baseline, with an illness duration ranging from 1 to 36 months. Inclusion required moderate to severe symptomatology. Healthy controls had no lifetime psychiatric disorders or relevant medical conditions	Prospective longitudinal observational study	Plasma samples were collected repeatedly over a 24-h period at baseline and after treatment to assess diurnal patterns. Plasma orexin, cortisol, and insulin levels were measured using radioimmunoassay. Circadian clock gene expression was assessed in peripheral blood mononuclear cells using quantitative PCR. Psychopathology was evaluated with the PANSS. Circadian rhythmicity was analysed with standard cosinor methods.	At baseline, patients showed marked disturbances in circadian regulation, including altered diurnal rhythmicity of plasma orexin and clock gene expression compared with controls. Following 8 weeks of clozapine treatment, psychotic symptoms improved and circadian rhythmicity of plasma orexin and cortisol was partially restored, although some abnormalities in clock gene expression persisted. The authors conclude that first-episode schizophrenia is associated with circadian dysregulation involving the orexin system, and that clozapine treatment can partially normalise neuroendocrine rhythms without fully correcting underlying molecular clock alterations.
Tiwari et al., 2016 [17]	218 patients with schizophrenia or schizoaffective disorder were included in the discovery sample. Patients were treated predominantly with clozapine or olanzapine for up to 14 weeks. A replication analysis was conducted in an independent sample of 122 patients from the CATIE study treated with olanzapine or risperidone for up to 190 days. Baseline weight and duration of antipsychotic exposure were recorded for all participants	Case–control	Genotyping of single-nucleotide polymorphisms (SNPs) in orexin receptor genes (*HcrtR1* and *HcrtR2*) was performed using standard molecular genetic techniques. Body weight and BMI were measured at baseline and follow-up. Antipsychotic treatment type and duration were documented. Associations between genetic variants and antipsychotic-induced weight gain were assessed using ANCOVA, controlling for baseline weight and treatment duration. Replication analyses were performed in the CATIE subsample.	Several polymorphisms in HcrtR2 were significantly associated with antipsychotic-induced weight gain in the discovery cohort. Selected variants showed consistent direction of effect in the replication sample, although not all associations reached statistical significance. The authors conclude that genetic variation in orexin receptor genes, particularly HcrtR2, is associated with individual susceptibility to antipsychotic-induced weight gain in schizophrenia, supporting a role of the orexin system in antipsychotic-related metabolic effects.
Sansa et al., 2016 [28]	366 adult patients with schizophrenia or schizoaffective disorder (DSM-IV criteria) were consecutively screened at two tertiary hospitals in Spain. Of these, 35 patients reported hypersomnia and/or cataplexy-like symptoms and entered the second phase of evaluation. 24 patients completed detailed clinical assessment, 5 were positive for HLA DQB1*06:02, and cerebrospinal fluid hypocretin-1 levels were measured in three patients	Prospective, single-centre, stepwise observational screening study	Narcolepsy-related symptoms were assessed using a structured screening questionnaire and the Epworth Sleepiness Scale. Patients with hypersomnia and/or cataplexy-like symptoms underwent expert sleep evaluation and HLA DQB1*06:02 genotyping. Cerebrospinal fluid hypocretin-1 levels were measured by radioimmunoassay in HLA-positive patients who consented to lumbar puncture. Clinical and treatment variables were recorded.	No cases of definite narcolepsy type 1 were identified in the cohort. Among the 35 patients reporting hypersomnia and/or cataplexy-like symptoms, only five were HLA DQB1*06:02 positive, and all three patients who underwent lumbar puncture showed normal CSF hypocretin-1 levels (>200 pg/mL). Excessive daytime sleepiness was infrequent, with only 5.7% of the total sample showing clinically significant sleepiness, often temporally related to antipsychotic treatment or comorbid sleep disorders. The authors conclude that narcolepsy with hypocretin deficiency is not under-recognised in adult patients with schizophrenia or schizoaffective disorder, and that orexin/hypocretin deficiency does not appear to play a major role in sleep-related symptoms in this population.
Tsuchimine et al., 2019 [29]	80 patients with schizophrenia, 80 healthy controls, and additional mood disorder groups including major depressive disorder and bipolar disorder (40). Patients with schizophrenia were chronically ill and receiving antipsychotic treatment	Cross-sectional observational study	Plasma orexin-A (hypocretin-1) levels were measured by ELISA. Psychopathology was assessed using the PANSS in schizophrenia and the HAM-D in mood disorder groups. Daily doses of typical and atypical antipsychotics, as well as concomitant psychotropic medications, were recorded.	In schizophrenia, plasma orexin-A levels did not differ from HC and were not significantly associated with PANSS symptom severity or antipsychotic dosage. In contrast, patients with BD showed significantly lower orexin-A levels compared with HC. Overall, the findings indicate that peripheral orexin-A levels are not directly related to core psychotic symptoms or antipsychotic exposure in chronic schizophrenia, while reduced orexin-A may characterise affective psychopathology.
Chen et al., 2019 [30]	219 participants were included, comprising 159 patients with schizophrenia and 60 healthy nonpsychiatric controls, aged 20–65 years. Patients had a DSM-IV-TR diagnosis of schizophrenia confirmed by two senior psychiatrists and had been receiving continuous antipsychotic treatment for at least 6 months. Patients were divided into two treatment groups: those treated with clozapine and those treated with less obesogenic antipsychotics. Healthy controls had no lifetime psychiatric disorders as assessed by structured clinical interviews	Cross-sectional observational study	Plasma orexin-A levels were measured using commercial ELISA. Metabolic parameters included body mass index, waist circumference, blood pressure, fasting plasma glucose, triglycerides, HDL cholesterol, HbA1c, insulin levels, and HOMA-IR. Metabolic syndrome was defined according to modified NCEP ATP III criteria. Psychiatric symptom severity was assessed using the BPRS-18. Demographic and clinical variables were obtained from structured interviews and medical records.	Plasma orexin-A levels differed among patient groups according to antipsychotic treatment. In patients with schizophrenia, higher orexin-A levels were significantly associated with more favourable metabolic parameters, including lower fasting glucose, triglycerides, and blood pressure, as well as higher HDL-C. Logistic regression analyses showed that higher orexin-A levels were independently associated with a lower risk of metabolic syndrome after adjustment for demographic and clinical covariates. These associations were observed in patients with schizophrenia but not in healthy controls. Higher plasma orexin-A levels are associated with a reduced risk of metabolic syndrome in patients with schizophrenia receiving antipsychotic treatment. Findings suggest that the orexin system may be involved in metabolic regulation in antipsychotic-treated patients, potentially modulating susceptibility to metabolic adverse effects.
Liu et al., 2020 [31]	324 Chinese in-patients with chronic schizophrenia (193 men, 131 women) were enrolled. All participants met DSM-IV criteria for schizophrenia, were aged 18–75 years, and had an illness duration longer than 5 years. Patients with severe medical, neurological, or substance-related conditions were excluded. All participants were receiving stable antipsychotic treatment	Cross-sectional observational study	BMI was calculated using standardised anthropometric measures. Plasma orexin-A concentrations were measured using ELISA. Psychopathology was assessed with the PANSS, including total and five-factor scores. Metabolic variables included fasting glucose, triglycerides, HDL-C, and LDL-C. Antipsychotic treatment type and chlorpromazine-equivalent doses were recorded.	Higher BMI was significantly associated with elevated plasma orexin-A levels. Orexin-A levels were positively correlated with BMI and lipid indices (TG, HDL-C, LDL-C), but not with fasting glucose. Higher BMI was associated with fewer negative symptoms, while plasma orexin-A levels showed no direct correlation with PANSS scores after adjustment. Multivariate regression identified BMI as independently associated with plasma orexin-A levels, suggesting a link between orexin signalling and metabolic status rather than core psychopathology in chronic schizophrenia.
Lu et al., 2021 [14]	61 drug-naïve FES patients (27 males, 34 females; mean age ≈ 24 years) and 82 HC matched for age and sex. In addition, 56 postmortem human brain samples were analysed, including hypothalamic, SFG, and ventricular CSF samples from schizophrenia patients and matched controls	Multimodal case-control study	Plasma and ventricular CSF hypocretin-1 levels were measured using immunoassays. Postmortem hypothalamic hypocretin-1 immunoreactivity was quantified by immunohistochemistry. mRNA expression of Hcrt-R1 and Hcrt-R2 in the SFG was assessed using quantitative PCR. Clinical symptoms were evaluated with standardised psychiatric assessments, and demographic and clinical variables were controlled in statistical analyses.	Patients with schizophrenia showed significantly reduced plasma hypocretin-1 levels compared with controls, an effect driven primarily by female patients. Postmortem analyses revealed reduced hypothalamic hypocretin-1 immunoreactivity and decreased ventricular CSF hypocretin-1 levels in schizophrenia. Female patients exhibited reduced Hcrt-R2 mRNA expression in the SFG, while male patients showed a trend toward increased receptor expression. Hypocretin alterations were not explained by illness duration or BMI and were interpreted as reflecting central hypocretin system dysregulation, partially mirrored in peripheral measures.
Chen et al., 2022 [16]	199 patients with schizophrenia: 37 APD-free and 162 clozapine-treated. Clozapine-treated patients were classified as clozapine-responsive (*n* = 100) or clozapine-resistant (*n* = 62) based on psychotic remission defined by the BPRS-18	Cross-sectional observational study	Plasma orexin-A levels. Psychopathology assessed using the BPRS-18 (total and subscale scores). Cognitive functioning assessed with the CogState Schizophrenia Battery.	Clozapine-responsive patients showed significantly higher orexin-A levels compared with clozapine-resistant and APD-free patients. Orexin-A level was the only factor independently associated with clozapine treatment response after adjustment. Orexin-A levels were negatively correlated with BPRS-18 total score and positive, negative, and general symptom subscales, and positively correlated with verbal memory, visual learning and memory, and working memory performance.
Ren et al., 2022 [32]	140 patients with chronic schizophrenia diagnosed according to DSM criteria and receiving long-term clozapine treatment were included. Among them, 63 met criteria for metabolic syndrome and 77 did not. All participants were clinically stable at the time of assessment	Cross-sectional observational study	Plasma orexin-A and BDNF levels were measured using ELISA. Metabolic syndrome was diagnosed according to standardised clinical criteria. Cognitive functioning was assessed with the RBANS, and psychopathology was evaluated using the PANSS. Anthropometric and metabolic parameters, including BMI, waist circumference, fasting glucose, and lipid profile, as well as clinical variables such as illness duration and clozapine treatment duration, were recorded.	Plasma orexin-A and BDNF levels were measured using ELISA. Metabolic syndrome was diagnosed according to standardised clinical criteria. Cognitive functioning was assessed with the RBANS, and psychopathology was evaluated using the PANSS. Anthropometric and metabolic parameters, including BMI, waist circumference, fasting glucose, and lipid profile, as well as clinical variables such as illness duration and clozapine treatment duration, were recorded.
Yu et al., 2023 [33]	195 participants were included: 54 patients with FES, 52 with BD, 35 with MDD, and 54 HC. Participants were recruited from inpatient and outpatient psychiatric facilities at West China Hospital of Sichuan University. Diagnoses were established according to DSM-IV criteria. FES patients were at an early stage of illness and were drug-naïve, whereas BD and MDD patients had variable prior exposure to psychotropic medications. Healthy controls were screened using the SCID (non-patient version) and had no lifetime psychiatric disorders. Subjects with substance abuse within the previous year or significant medical illnesses were excluded	Cross-sectional case-control study	Plasma levels of α-MSH, β-endorphin, neurotensin, orexin-A, oxytocin, and substance P measured using a quantitative multiplex immunoassay. Psychotic symptoms assessed using the BPRS and the PANSS. Manic symptoms assessed with the YMRS. Depressive symptoms assessed using the 17-item HAMD. Cognitive functioning evaluated using a comprehensive neuropsychological test battery assessing executive function and other cognitive domains.	Plasma levels of α-MSH, neurotensin, orexin-A, oxytocin, and substance P were significantly reduced in patients with first-episode schizophrenia, bipolar disorder, and major depressive disorder compared with healthy controls. β-endorphin levels were reduced only in the BD group. No neuropeptide reliably differentiated between the three patient groups, although neurotensin showed the strongest ability to distinguish patients from controls overall. Across the entire sample, higher neurotensin levels were associated with better executive functioning. In the FES and BD groups, lower oxytocin and higher substance P levels were associated with greater severity of psychotic symptoms. β-endorphin levels were associated with early morning awakening across all diagnostic groups. The authors conclude that reduced circulating neuropeptides, including orexin-A, represent potential biomarkers of severe mental illness and may be relevant targets for interventions aimed at improving clinical symptoms and cognitive function, particularly in early-stage psychiatric disorders, although causality cannot be inferred due to the cross-sectional design.
Chen et al., 2024 [34]	199 patients with schizophrenia were included, comprising 37 antipsychotic drug–free patients and 162 clozapine-treated patients with TRS. The clozapine-treated group was further subdivided into clozapine-responsive patients (*n* = 100) and clozapine-resistant patients (*n* = 62)	Cross-sectional observational study	Blood orexin-A levels measured using immunoassay techniques. Psychotic symptom severity assessed using the BPRS-18, including total score and positive, negative, and general psychopathology subscales. Treatment response to clozapine defined by achievement of psychotic remission on the BPRS-18. Cognitive functioning assessed using the CogState Schizophrenia Battery, covering verbal memory, visual learning and memory, and working memory.	Clozapine-responsive patients exhibited significantly higher circulating orexin-A levels compared with both clozapine-resistant patients and antipsychotic-free patients. After adjustment for potential confounders, orexin-A level was the only variable independently associated with clozapine treatment response. Higher orexin-A levels were negatively correlated with overall psychotic symptom severity as well as positive, negative, and general symptom subscales. In addition, orexin-A levels showed positive correlations with multiple cognitive domains, including verbal memory, visual learning and memory, and working memory. The authors conclude that elevated orexin-A levels are associated with a favourable therapeutic response to clozapine in treatment-resistant schizophrenia, supporting a role for the orexin system in antipsychotic efficacy and cognitive functioning, and highlighting orexin signalling as a potential therapeutic target.
Tanaka et al., 2025 [35]	17 long-term inpatients with DSM-5 schizophrenia were recruited from a single psychiatric hospital in Japan. Inclusion criteria included illness duration ≥ 15 years and continuous hospitalisation. The median age was 59 years, and approximately 2/3 of participants were male. All patients were receiving antipsychotic medication; none were treated with clozapine	Single-centre longitudinal observational study	Serum orexin-A levels were measured at baseline and follow-up using a commercial ELISA. Physical activity and sleep parameters were assessed using actigraphy over one week at each time point, including step count and sleep indices. Psychotic symptoms were evaluated using the PANSS. Associations between changes in variables were examined using non-parametric correlation analyses.	Changes in serum orexin-A levels over six months were positively correlated with changes in daily physical activity (step count) and negatively correlated with changes in time spent in bed. No significant longitudinal associations were observed between orexin-A changes and PANSS scores, antipsychotic dose, or body mass index. The authors conclude that in this preliminary longitudinal study of hospitalised patients with schizophrenia, changes in serum orexin-A were associated with changes in physical activity and sleep-related behaviour but not with changes in psychotic symptom severity. The findings are exploratory and warrant confirmation in larger samples.

Abbreviations: ACE, angiotensin-converting enzyme; ANCOVA, analysis of covariance; APD, antipsychotic drug(s); BD, bipolar disorder; BDNF, brain-derived neurotrophic factor; BPRS-18, 18-item Brief Psychiatric Rating Scale; BMI, body mass index; CSF, cerebrospinal fluid; DNA, deoxyribonucleic acid; DSM-IV, -5, Diagnostic and Statistical Manual of Mental Disorders, IV-5th edition, TR, text revision; EEG, electroencephalogram; ELISA, enzyme-linked immunosorbent assay; EMG, electromyogram; EOG, electro-oculogram; FES, first-episode schizophrenia; GBS, Guillain–Barré syndrome; HC, healthy control(s); HAMD, Hamilton Depression Rating Scale; Hcrt-R1, 2, hypocretin receptors, -1, -2; HDL-C, high-density lipoproteins-cholesterol; HLA, Human Leukocyte Antigens; ICU, intensive care unit; LDL-C, low-density lipoproteins-cholesterol; MDD, major depressive disorder; N–C, Narcolepsy–cataplexy; PANSS, Positive And Negative Syndrome Scale; PCR, polymerase chain reaction; PHS, polydipsia–hyponatremia; RBANS, Repeatable Battery for the Assessment of Neuropsychological Status; SCID, Structured Clinical Interview for DSM-IV; SFG, superior frontal gyrus; TRS, treatment-resistant schizophrenia; WCST, Wisconsin Card Sorting Test; YMRS, Young Mania Rating Scale; α-MSH, alpha-melanocyte stimulating hormone.

## Data Availability

No new data were generated for this review; all data are in the published material of the studies that were referred to.

## Supplementary Materials

The following supporting information can be downloaded at: https://www.mdpi.com/article/10.3390/brainsci16040361/s1, Table S1: Search strategy; Table S2: Counts of studies and samples; Table S3: Risk of bias of included studies according to the Robins-E tool; Table S4: PRISMA 2020 Checklist.

## Abbreviations

The following abbreviations are used in this manuscript:

DORAs	Dual orexin receptor antagonists
ELISA	Enzyme-linked immunosorbent assay
HcrtR1, -2	Hypocretin receptor type 1 (Orexin-A receptor), -2 (Orexin-B receptor)
NMDA	N-methyl-D-aspartate
PRISMA	Preferred Reporting Items for Systematic reviews and Meta-Analyses
ROBINS-E	Cochrane Risk Of Bias In Non-randomised Studies-of Exposures
