# Supplementary material for: Orexins, Psychosis, and Antipsychotics: A Systematic Review of Studies of Orexin Levels and the Effects of Dual Orexin Receptor Antagonists (DORAs)
Source: Brain Sci. 2026 Mar 27;16(4):361. doi: 10.3390/brainsci16040361 (PMC13115374; doi:10.3390/brainsci16040361)
Supplement: Supplementary file 1 [file brainsci-16-00361-s001.zip › brainsci-4164832-supplementary.pdf]

Table S1. Search strategy

(schizophr* OR psychosis OR psychotic OR paranoi* OR schizoaffect* OR antipsychotic* OR neuroleptic* OR phenothiazine* OR butyrophenon* OR "dopamine antagonist*" OR "dopamine receptor antagonist*" OR "substituted benzamide*" OR thioxanthene* OR dibenzoazepine* OR benzisoxazole* OR haloperidol OR chlorpromazine OR promazine OR thioridazine OR clothiapine OR loxapine OR clozapine OR quetiapine OR olanzapine OR fluperlapin* OR zotepine OR remoxipride OR sertindole OR risperidone OR paliperidone OR aripiprazole OR brexpiprazole OR cariprazine OR ziprasidone OR asenapine OR bromperidol OR spiroperidol OR spiperone OR perphenazine OR fluphenazine OR flupentixol* OR clopenthixol OR zuclopenthixol OR droperidol OR thioxanthene OR sulpiride OR sultopride OR metoclopramide OR amisulpride) AND (orexin* OR hypocretin* OR Hcrts OR OX1 OR OX2 OR OX1R OR OX2R OR suvorexant OR MK-4305 OR lemborexant OR daridorexant OR almorexant OR ACT-078573 OR filorexant OR MK-6096 OR seltorexant OR MIN-202 OR JNJ-42847922 OR JNJ-922 OR Fazamorexant OR YZJ-1139 OR Nivasorexant OR ACT-539313 OR Tebideutorexant OR JNJ-61393215 OR JNJ-3215 OR Vornorexant OR ORN-0829 OR TS-142 OR ACT-335827 OR EMPA OR GSK-649868 OR SB-649868 OR JNJ-10397049 OR RTIOX-276 OR SB-334867 OR SB-408124 OR TCS-OX2-29) 3.3.2026 PubMed 441 results

1
Boullin DJ, Grimes RP. Increased platelet aggregation in patients receiving chlorpromazine: responses to 5-hydroxytryptamine, dopamine and N-dimethyl dopamine. Br J Clin Pharmacol. 1976;3(4):649-53. doi: 10.1111/j.1365-2125.1976.tb04889.x.
No orexin
2
Iversen SD. Interactions between excitatory amino acids and dopamine systems in the forebrain: implications for schizophrenia and Parkinson's disease. Behav Pharmacol. 1995;6(5-6):478-491.
Review
3
Pedersen ME, Dorrington KL, Robbins PA. Effects of haloperidol on ventilation during isocapnic hypoxia in humans. J Appl Physiol (1985). 1997;83(4):1110-5. doi: 10.1152/jappl.1997.83.4.1110.
No orexin
4
Thornley B, Adams C. Content and quality of 2000 controlled trials in schizophrenia over 50 years. BMJ. 1998;317(7167):1181-4. doi: 10.1136/bmj.317.7167.1181.
Review
5
Pedersen ME, Dorrington KL, Robbins PA. Effects of dopamine and domperidone on ventilatory sensitivity to hypoxia after 8 h of isocapnic hypoxia. J Appl Physiol (1985). 1999;86(1):222-9. doi: 10.1152/jappl.1999.86.1.222.
No orexin
6
Stein J, Richardson A. Cognitive disorders: A question of misattribution. Curr Biol. 1999;9(10):R374-6. doi: 10.1016/s0960-9822(99)80231-0.
No orexin
7
Newberry NR, Footitt DR, Papanastassiou V, Reynolds DJ. Actions of 5-HT on human neocortical neurones in vitro. Brain Res. 1999;833(1):93-100. doi: 10.1016/s0006-8993(99)01540-1.
No orexin
8
Thornley B, Adams CE, Awad G. Chlorpromazine versus placebo for schizophrenia. Cochrane Database Syst Rev. 2000;(2):CD000284. doi: 10.1002/14651858.CD000284. Update in: Cochrane Database Syst Rev. 2003;(2):CD000284. doi: 10.1002/14651858.CD000284.
No orexin
9
Adams CE, Eisenbruch M. Depot fluphenazine for schizophrenia. Cochrane Database Syst Rev. 2000;(2):CD000307. doi: 10.1002/14651858.CD000307. Update in: Cochrane Database Syst Rev. 2005;(1):CD000307. doi: 10.1002/14651858.CD000307.
Review
10
Joy CB, Adams CE, Rice K. Crisis intervention for people with severe mental illnesses. Cochrane Database Syst Rev. 2000;(2):CD001087. doi: 10.1002/14651858.CD001087. Update in: Cochrane Database Syst Rev. 2004;(4):CD001087. doi: 10.1002/14651858.CD001087.pub2.
Review
11
Joy CB, Mumby-Croft R, Joy LA. Polyunsaturated fatty acid (fish or evening primrose oil) for schizophrenia. Cochrane Database Syst Rev. 2000;(2):CD001257. doi: 10.1002/14651858.CD001257. Update in: Cochrane Database Syst Rev. 2003;(2):CD001257. doi: 10.1002/14651858.CD001257.
Review
12
Nakamura T, Uramura K, Nambu T, Yada T, Goto K, Yanagisawa M, Sakurai T. Orexin-induced hyperlocomotion and stereotypy are mediated by the dopaminergic system. Brain Res. 2000;873(1):181-7. doi: 10.1016/s0006-8993(00)02555-5.
Animal
13
Russell SH, Kim MS, Small CJ, Abbott CR, Morgan DG, Taheri S, Murphy KG, Todd JF, Ghatei MA, Bloom SR. Central administration of orexin A suppresses basal and domperidone stimulated plasma prolactin. J Neuroendocrinol. 2000;12(12):1213-8.
Animal
14
Adams C, Wilson P, Bagnall AM. Psychosocial interventions for schizophrenia. Qual Health Care. 2000;9(4):251-6. doi: 10.1136/qhc.9.4.251.
Review
15
Canales JJ, Iversen SD. Psychomotor-activating effects mediated by dopamine $D_2$ and $D_3$ receptors in the nucleus accumbens. Pharmacol Biochem Behav. 2000;67(1):161-8. doi: 10.1016/s0091-3057(00)00311-7.
Animal
16
Howson N, Adams CE. Always search this year's disc. Health Libr Rev. 2000;17(3):171-2. doi: 10.1046/j.1365-2532.2000.00282.x.
Review
17
Greene JR, Kerkhoff JE, Guiver L, Totterdell S. Structural and functional abnormalities of the hippocampal formation in rats with environmentally induced reductions in prepulse inhibition of acoustic startle. Neuroscience. 2001;103(2):315-23. doi: 10.1016/s0306-4522(00)00560-1.
Animal
18
Carpenter S, Berk M. Clotiapine for acute psychotic illnesses. Cochrane Database Syst Rev. 2001;(1):CD002304. doi: 10.1002/14651858.CD002304. Update in: Cochrane Database Syst Rev. 2004;(4):CD002304. doi: 10.1002/14651858.CD002304.pub2.
Review

19
Wolfart J, Neuhoff H, Franz O, Roeper J. Differential expression of the small-conductance, calcium-activated potassium channel SK3 is critical for pacemaker control in dopaminergic midbrain neurons. J Neurosci. 2001;21(10):3443-56. doi: 10.1523/JNEUROSCI.21-10-03443.2001.
Animal
20
Joy CB, Adams CE, Lawrie SM. Haloperidol versus placebo for schizophrenia. Cochrane Database Syst Rev. 2001;(2):CD003082. doi: 10.1002/14651858.CD003082. Update in: Cochrane Database Syst Rev. 2006;(4):CD003082. doi: 10.1002/14651858.CD003082.pub2.
Review
21
Deacon RM, Raley JM, Perry VH, Rawlins JN. Burrowing into prion disease. Neuroreport. 2001;12(9):2053-7. doi: 10.1097/00001756-200107030-00052.
Animal
22
Shaw J, Claridge G, Clark K. Schizotypy and the shift from dextrality: a study of handedness in a large non-clinical sample. Schizophr Res. 2001;50(3):181-9. doi: 10.1016/s0920-9964(00)00167-5.
Unrelated
23
Lo D, Hilbush B, Sutcliffe JG. TOGA analysis of gene expression to accelerate target development. Eur J Pharm Sci. 2001;14(3):191-6. doi: 10.1016/s0928-0987(01)00174-9.
Unfocused
24
Law AJ, Deakin JF. Asymmetrical reductions of hippocampal NMDAR1 glutamate receptor mRNA in the psychoses. Neuroreport. 2001;12(13):2971-4. doi: 10.1097/00001756-200109170-00043.
*Post-mortem*
25
Hille CJ, Fox SH, Maneuf YP, Crossman AR, Brotchie JM. Antiparkinsonian action of a delta opioid agonist in rodent and primate models of Parkinson's disease. Exp Neurol. 2001;172(1):189-98. doi: 10.1006/exnr.2001.7763.
Animal
26
Fenton M, Coutinho ES, Campbell C. Zuclopenthixol acetate in the treatment of acute schizophrenia and similar serious mental illnesses. Cochrane Database Syst Rev. 2001;(3):CD000525. doi: 10.1002/14651858.CD000525. Update in: Cochrane Database Syst Rev. 2004;(3):CD000525. doi: 10.1002/14651858.CD000525.pub2.
Review
27
Telegdy G, Adamik A. The action of orexin A on passive avoidance learning. Involvement of transmitters. Regul Pept. 2002;104(1-3):105-10. doi: 10.1016/s0167-0115(01)00341-x.
Animal
28
Matsuzaki I, Sakurai T, Kunii K, Nakamura T, Yanagisawa M, Goto K. Involvement of the serotonergic system in orexin-induced behavioral alterations in rats. Regul Pept. 2002;104(1-3):119-23. doi: 10.1016/s0167-0115(01)00355-x.
Animal
29
Neuhoff H, Neu A, Liss B, Roeper J. I(h) channels contribute to the different functional properties of identified dopaminergic subpopulations in the midbrain. J Neurosci. 2002;22(4):1290-302. doi: 10.1523/JNEUROSCI.22-04-01290.2002.
Animal
**30**
**Nishino S, Ripley B, Mignot E, Benson KL, Zarcone VP. CSF hypocretin-1 levels in schizophrenics and controls: relationship to sleep architecture. Psychiatry Res. 2002;110(1):1-7. doi: 10.1016/s0165-1781(02)00032-x.**
**Included**
31
Fadel J, Bubser M, Deutch AY. Differential activation of orexin neurons by antipsychotic drugs associated with weight gain. J Neurosci. 2002;22(15):6742-6. doi: 10.1523/JNEUROSCI.22-15-06742.2002.
Animal
32
Katz JD, Ropper AH. Familial Kleine-Levin syndrome: two siblings with unusually long hypersomnic spells. Arch Neurol. 2002;59(12):1959-61. doi: 10.1001/archneur.59.12.1959.
Case
33
Monda M, Viggiano A, De Luca V. Haloperidol reduces the sympathetic and thermogenic activation induced by orexin A. Neurosci Res. 2003;45(1):17-23. doi: 10.1016/s0168-0102(02)00191-8.
Animal
34
Cullen TJ, Walker MA, Parkinson N, Craven R, Crow TJ, Esiri MM, Harrison PJ. A postmortem study of the mediodorsal nucleus of the thalamus in schizophrenia. Schizophr Res. 2003;60(2-3):157-66. doi: 10.1016/s0920-9964(02)00297-9.
*Post-mortem*
35
Douglass AB. Narcolepsy: differential diagnosis or etiology in some cases of bipolar disorder and schizophrenia? CNS Spectr. 2003;8(2):120-6. doi: 10.1017/s1092852900018344.
Review
36
Young WB, Piovesan EJ, Biglan KM. Restless legs syndrome and drug-induced akathisia in headache patients. CNS Spectr. 2003;8(6):450-6. doi: 10.1017/s1092852900018769.
Unfocused
37
Sasa M, Nishi A, Kobayashi K, Sano H, Momiyama T, Uramura K, Yada T, Mori N, Suzuki K, Minabe Y. ドパミンによる運動精神機能調節：新たな研究への展開 [Regulation of psychomotor functions by dopamine: integration of various approaches]. Nihon Yakurigaku Zasshi (Folia Pharmacol Jpn). 2003;122(3):215-25. Japanese. doi: 10.1254/fpj.122.215.
Animal

38
Highley JR, DeLisi LE, Roberts N, Webb JA, Relja M, Razi K, Crow TJ. Sex-dependent effects of schizophrenia: an MRI study of gyral folding, and cortical and white matter volume. Psychiatry Res. 2003;124(1):11-23. doi: 10.1016/s0925-4927(03)00076-3.
No orexin
**39**
**Dalal MA, Schuld A, Pollmächer T. Lower CSF orexin A (hypocretin-1) levels in patients with schizophrenia treated with haloperidol compared to unmedicated subjects. Mol Psychiatry. 2003;8(10):836-7. doi: 10.1038/sj.mp.4001363.**
**Included**
40
Pei Q, Zetterström TS, Sprakes M, Tordera R, Sharp T. Antidepressant drug treatment induces Arc gene expression in the rat brain. Neuroscience. 2003;121(4):975-82. doi: 10.1016/s0306-4522(03)00504-9.
Animal
41
Le Masurier M, Houston G, Cowen P, Grasby P, Sharp T, Hume S. Tyrosine-free amino acid mixture attenuates amphetamine-induced displacement of [$^{11}$C]raclopride in striatum in vivo: a rat PET study. Synapse. 2004;51(2):151-7. doi: 10.1002/syn.10285.
Animal
42
Richardson AJ, Cyhlarova E, Ross MA. Omega-3 and omega-6 fatty acid concentrations in red blood cell membranes relate to schizotypal traits in healthy adults. Prostaglandins Leukot Essent Fatty Acids. 2003;69(6):461-6. doi: 10.1016/j.plefa.2003.08.018.
No orexin
43
Sheitman BB, Knable MB, Jarskog LF, Chakos M, Boyce LH, Early J, Lieberman JA. Secretin for refractory schizophrenia. Schizophr Res. 2004;66(2-3):177-81. doi: 10.1016/S0920-9964(03)00068-9.
Unfocused
44
Benson MA, Sillitoe RV, Blake DJ. Schizophrenia genetics: dysbindin under the microscope. Trends Neurosci. 2004;27(9):516-9. doi: 10.1016/j.tins.2004.06.004.
Review
45
Chance SA, Tzotzoli PM, Vitelli A, Esiri MM, Crow TJ. The cytoarchitecture of sulcal folding in Heschl's sulcus and the temporal cortex in the normal brain and schizophrenia: lamina thickness and cell density. Neurosci Lett. 2004;367(3):384-8. doi: 10.1016/j.neulet.2004.06.041.
No orexin
46
Monda M, Viggiano A, Viggiano A, Fuccio F, De Luca V. Clozapine blocks sympathetic and thermogenic reactions induced by orexin A in rat. Physiol Res. 2004;53(5):507-13.
Animal
47
Cure S, Rathbone J, Carpenter S. Droperidol for acute psychosis. Cochrane Database Syst Rev. 2004;(4):CD002830. doi: 10.1002/14651858.CD002830.pub2. Update in: Cochrane Database Syst Rev. 2016;12:CD002830. doi: 10.1002/14651858.CD002830.pub3.
Review
48
Hardwick C, French SJ, Southam E, Totterdell S. A comparison of possible markers for chandelier cartridges in rat medial prefrontal cortex and hippocampus. Brain Res. 2005;1031(2):238-44. doi: 10.1016/j.brainres.2004.10.047.
Animal
49
Buckingham SD, Pym L, Jones AK, Brown L, Sansom MS, Sattelle DB, Biggin PC. A7DB: a relational database for mutational, physiological and pharmacological data related to the alpha7 nicotinic acetylcholine receptor. BMC Neurosci. 2005;6:2. doi: 10.1186/1471-2202-6-2.
No orexin
50
Chance SA, Esiri MM, Crow TJ. Macroscopic brain asymmetry is changed along the antero-posterior axis in schizophrenia. Schizophr Res. 2005;74(2-3):163-70. doi: 10.1016/j.schres.2004.09.001.
*Post-mortem*
51
Monda M, Viggiano A, Viggiano A, Viggiano E, De Luca V. Risperidone potentiates the sympathetic and hyperthermic reactions induced by orexin A in the rat. Physiol Res. 2006;55(1):73-78. doi: 10.33549/physiolres.930906. Epub 2005 Apr 26.
Animal
52
Denk F, Walton ME, Jennings KA, Sharp T, Rushworth MF, Bannerman DM. Differential involvement of serotonin and dopamine systems in cost-benefit decisions about delay or effort. Psychopharmacology (Berl). 2005;179(3):587-96. doi: 10.1007/s00213-004-2059-4. Epub 2004 Dec 10.
Animal
53
Walterfang M, Upjohn E, Velakoulis D. Is schizophrenia associated with narcolepsy? Cogn Behav Neurol. 2005;18(2):113-8. doi: 10.1097/01.wnn.0000160822.53577.2c.
Case
54
Bubser M, Fadel JR, Jackson LL, Meador-Woodruff JH, Jing D, Deutch AY. Dopaminergic regulation of orexin neurons. Eur J Neurosci. 2005;21(11):2993-3001. doi: 10.1111/j.1460-9568.2005.04121.x.
Animal
**55**
**Meerabux J, Iwayama Y, Sakurai T, Ohba H, Toyota T, Yamada K, Nagata R, Irukayama-Tomobe Y, Shimizu H, Yoshitsugu K, Ohta K, Yoshikawa T. Association of an orexin 1 receptor 408Val variant with polydipsia-hyponatremia in schizophrenic subjects. Biol Psychiatry. 2005;58(5):401-7. doi: 10.1016/j.biopsych.2005.04.015.**
**Included**
**56**
**Cochen V, Arnulf I, Demeret S, Neulat ML, Gourlet V, Drouot X, Moutereau S, Derenne JP, Similowski T, Willer JC, Pierrot-Deseiligny C, Bolgert F. Vivid dreams, hallucinations, psychosis and REM sleep in Guillain-Barré syndrome. Brain. 2005;128(Pt 11):2535-45. doi: 10.1093/brain/awh585. Epub 2005 Jul 6.**

**Included**
57
Rasmussen K, Benvenga MJ, Bymaster FP, Calligaro DO, Cohen IR, Falcone JF, Hemrick-Luecke SK, Martin FM, Moore NA, Nisenbaum LK, Schaus JM, Sundquist SJ, Tupper DE, Wiernicki TR, Nelson DL. Preclinical pharmacology of FMPD [6-fluoro-10-[3-(2-methoxyethyl)-4-methyl-piperazin-1-yl]-2-methyl-4H-3-thia-4,9-diaza-benzo[f]azulene]: a potential novel antipsychotic with lower histamine H1 receptor affinity than olanzapine. J Pharmacol Exp Ther. 2005;315(3):1265-77. doi: 10.1124/jpet.105.089326. Epub 2005 Sep 1.
*In vitro*
58
Cyhlarova E, Claridge G. Development of a version of the Schizotypy Traits Questionnaire (STA) for screening children. Schizophr Res. 2005;80(2-3):253-61. doi: 10.1016/j.schres.2005.07.037. Epub 2005 Sep 19.
Unrelated
59
Narita M, Nagumo Y, Hashimoto S, Narita M, Khotib J, Miyatake M, Sakurai T, Yanagisawa M, Nakamachi T, Shioda S, Suzuki T. Direct involvement of orexinergic systems in the activation of the mesolimbic dopamine pathway and related behaviors induced by morphine. J Neurosci. 2006;26(2):398-405. doi: 10.1523/JNEUROSCI.2761-05.2006.
Animal
60
Webster JP, Lamberton PH, Donnelly CA, Torrey EF. Parasites as causative agents of human affective disorders? The impact of anti-psychotic, mood-stabilizer and anti-parasite medication on *Toxoplasma gondii*'s ability to alter host behaviour. Proc Biol Sci. 2006;273(1589):1023-30. doi: 10.1098/rspb.2005.3413.
Animal
61
Steffen KJ, Roerig JL, Mitchell JE, Uppala S. Emerging drugs for eating disorder treatment. Expert Opin Emerg Drugs. 2006;11(2):315-36. doi: 10.1517/14728214.11.2.315.
Review
62
Day-Wilson KM, Jones DN, Southam E, Cilia J, Totterdell S. Medial prefrontal cortex volume loss in rats with isolation rearing-induced deficits in prepulse inhibition of acoustic startle. Neuroscience. 2006;141(3):1113-21. doi: 10.1016/j.neuroscience.2006.04.048. Epub 2006 Jun 5.
Animal
63
Kotz CM, Wang C, Teske JA, Thorpe AJ, Novak CM, Kiwaki K, Levine JA. Orexin A mediation of time spent moving in rats: neural mechanisms. Neuroscience. 2006;142(1):29-36. doi: 10.1016/j.neuroscience.2006.05.028. Epub 2006 Jun 30.
Animal
64
Hamlin AS, Blatchford KE, McNally GP. Renewal of an extinguished instrumental response: neural correlates and the role of D1 dopamine receptors. Neuroscience. 2006;143(1):25-38. doi: 10.1016/j.neuroscience.2006.07.035. Epub 2006 Sep 1.
Animal
65
Alberto CO, Trask RB, Quinlan ME, Hirasawa M. Bidirectional dopaminergic modulation of excitatory synaptic transmission in orexin neurons. J Neurosci. 2006;26(39):10043-50. doi: 10.1523/JNEUROSCI.1819-06.2006. **Retraction** in: J Neurosci. 2012;32(26):9116. doi: 10.1523/JNEUROSCI.1889-12.2012.
**Retract**
66
Monda M, Viggiano A, Viggiano A, Viggiano E, Messina G, Tafuri D, De Luca V. Quetiapine lowers sympathetic and hyperthermic reactions due to cerebral injection of orexin A. Neuropeptides. 2006;40(5):357-63. doi: 10.1016/j.npep.2006.07.003. Epub 2006 Sep 28.
Animal
67
Totterdell S. The anatomy of co-morbid neuropsychiatric disorders based on cortico-limbic synaptic interactions. Neurotox Res. 2006;10(2):65-85. doi: 10.1007/BF03033236.
Review
68
Rasmussen K, Hsu MA, Yang Y. The orexin-1 receptor antagonist SB-334867 blocks the effects of antipsychotics on the activity of A9 and A10 dopamine neurons: implications for antipsychotic therapy. Neuropsychopharmacology. 2007;32(4):786-92. doi: 10.1038/sj.npp.1301239. Epub 2006 Oct 25.
Animal
69
Jones AK, Raymond-Delpech V, Thany SH, Gauthier M, Sattelle DB. The nicotinic acetylcholine receptor gene family of the honey bee, *Apis mellifera*. Genome Res. 2006;16(11):1422-30. doi: 10.1101/gr.4549206. Epub 2006 Oct 25.
Animal
70
Monda M, Viggiano A, Viggiano A, Viggiano E, Messina G, Tafuri D, De Luca V. Sympathetic and hyperthermic reactions by orexin A: role of cerebral catecholaminergic neurons. Regul Pept. 2007;139(1-3):39-44. doi: 10.1016/j.regpep.2006.10.002. Epub 2006 Nov 28.
Animal
71
Brundin L, Petersén A, Björkqvist M, Träskman-Bendz L. Orexin and psychiatric symptoms in suicide attempters. J Affect Disord. 2007;100(1-3):259-63. doi: 10.1016/j.jad.2006.10.019. Epub 2006 Dec 4.
Unfocused
72
Brundin L, Björkqvist M, Petersén A, Träskman-Bendz L. Reduced orexin levels in the cerebrospinal fluid of suicidal patients with major depressive disorder. Eur Neuropsychopharmacol. 2007;17(9):573-9. doi: 10.1016/j.euroneuro.2007.01.005. Epub 2007 Mar 7.
No antipsychotics
73
Iversen SD, Iversen LL. Dopamine: 50 years in perspective. Trends Neurosci. 2007;30(5):188-93. doi: 10.1016/j.tins.2007.03.002. Epub 2007 Mar 26.
Review
74
Terrar DA, Wilson CM, Graham SG, Bryant SM, Heath BM. Comparison of guinea-pig ventricular myocytes and dog Purkinje fibres for in vitro assessment of drug-induced delayed repolarization. J Pharmacol Toxicol Methods. 2007;56(2):171-85. doi: 10.1016/j.vascn.2007.04.005. Epub 2007 May 26.
Animal
75

Morein-Zamir S, Turner DC, Sahakian BJ. A review of the effects of modafinil on cognition in schizophrenia. Schizophr Bull. 2007;33(6):1298-306. doi: 10.1093/schbul/sbm090. Epub 2007 Jul 18.
Review
76
Lambe EK, Liu RJ, Aghajanian GK. Schizophrenia, hypocretin (orexin), and the thalamocortical activating system. Schizophr Bull. 2007;33(6):1284-90. doi: 10.1093/schbul/sbm088. Epub 2007 Jul 26.
Review
77
Rasmussen K, Hsu MA, Noone S, Johnson BG, Thompson LK, Hemrick-Luecke SK. The orexin-1 antagonist SB-334867 blocks antipsychotic treatment emergent catalepsy: implications for the treatment of extrapyramidal symptoms. Schizophr Bull. 2007;33(6):1291-7. doi: 10.1093/schbul/sbm087. Epub 2007 Jul 28.
Animal
78
Minzenberg MJ, Carter CS. Modafinil: a review of neurochemical actions and effects on cognition. Neuropsychopharmacology. 2008;33(7):1477-502. doi: 10.1038/sj.npp.1301534. Epub 2007 Aug 22.
Review
79
Deutch AY, Bubser M. The orexins/hypocretins and schizophrenia. Schizophr Bull. 2007;33(6):1277-83. doi: 10.1093/schbul/sbm096. Epub 2007 Aug 28.
Review
80
Miskowiak K, Inkster B, O'Sullivan U, Selvaraj S, Goodwin GM, Harmer CJ. Differential effects of erythropoietin on neural and cognitive measures of executive function 3 and 7 days post-administration. Exp Brain Res. 2008;184(3):313-21. doi: 10.1007/s00221-007-1102-1. Epub 2007 Sep 8.
Unrelated
**81**
**Fukunaka Y, Shinkai T, Hwang R, Hori H, Utsunomiya K, Sakata S, Naoe Y, Shimizu K, Matsumoto C, Ohmori O, Nakamura J. The orexin 1 receptor (HCRTR1) gene as a susceptibility gene contributing to polydipsia-hyponatremia in schizophrenia. Neuromolecular Med. 2007;9(4):292-7. doi: 10.1007/s12017-007-8001-2. Epub 2007 Aug 1.**
**Included**
82
McWilliams S, Pennington N, Aziz TZ, Brophy J. Globus pallidus deep brain stimulators for a case of severe neuroleptic-related dystonia and dyskinesia. Ir J Psychol Med. 2007;24(4):159-160. doi: 10.1017/S0790966700010612.
Case
83
Monda M, Viggiano A, Viggiano A, Mondola R, Viggiano E, Messina G, Tafuri D, De Luca V. Olanzapine blocks the sympathetic and hyperthermic reactions due to cerebral injection of orexin A. Peptides. 2008;29(1):120-6. doi: 10.1016/j.peptides.2007.10.016. Epub 2007 Oct 24.
Animal
84
Wallingford NM, Sinnayah P, Bymaster FP, Gadde KM, Krishnan RK, McKinney AA, Landbloom RP, Tollefson GD, Cowley MA. Zonisamide prevents olanzapine-associated hyperphagia, weight gain, and elevated blood glucose in rats. Neuropsychopharmacology. 2008;33(12):2922-33. doi: 10.1038/npp.2008.9. Epub 2008 Mar 5.
Animal
85
Mallet N, Pogosyan A, Sharott A, Csicsvari J, Bolam JP, Brown P, Magill PJ. Disrupted dopamine transmission and the emergence of exaggerated beta oscillations in subthalamic nucleus and cerebral cortex. J Neurosci. 2008;28(18):4795-806. doi: 10.1523/JNEUROSCI.0123-08.2008.
Animal
86
Molnár Z, Hoerder-Suabedissen A, Wang WZ, DeProto J, Davies K, Lee S, Jacobs EC, Campagnoni AT, Paulsen O, Piñon MC, Cheung AF. Genes involved in the formation of the earliest cortical circuits. Novartis Found Symp. 2007;288:212-24; discussion 224-9, 276-81.
Review
87
Bloomfield C, French SJ, Jones DN, Reavill C, Southam E, Cilia J, Totterdell S. Chandelier cartridges in the prefrontal cortex are reduced in isolation reared rats. Synapse. 2008;62(8):628-31. doi: 10.1002/syn.20521.
Animal
88
Janas-Kozik M, Stachowicz M, Mazurek U, Zajdel A, Wilczok A, Krupka-Matuszczyk I, Rybakowski JK. Preliminary study of the expression of genes connected with the orexigenic and anorexigenic system using microarray technique in anorexia nervosa. Neuropsychobiology. 2008;57(3):116-20. doi: 10.1159/000138913. Epub 2008 Jun 13.
Case
89
Lamberton PH, Donnelly CA, Webster JP. Specificity of the *Toxoplasma gondii*-altered behaviour to definitive versus non-definitive host predation risk. Parasitology. 2008;135(10):1143-50. doi: 10.1017/S0031182008004666. Epub 2008 Jul 14.
Animal
90
Qu WM, Huang ZL, Xu XH, Matsumoto N, Urade Y. Dopaminergic $D_1$ and $D_2$ receptors are essential for the arousal effect of modafinil. J Neurosci. 2008;28(34):8462-9. doi: 10.1523/JNEUROSCI.1819-08.2008.
Animal
91
Davoodi N, Kalinichev M, Korneev SA, Clifton PG. Hyperphagia and increased meal size are responsible for weight gain in rats treated sub-chronically with olanzapine. Psychopharmacology (Berl). 2009;203(4):693-702. doi: 10.1007/s00213-008-1415-1. Epub 2008 Dec 4.
Animal
92
Cope MB, Li X, Jumbo-Lucioni P, DiCostanzo CA, Jamison WG, Kesterson RA, Allison DB, Nagy TR. Risperidone alters food intake, core body temperature, and locomotor activity in mice. Physiol Behav. 2009;96(3):457-63. doi: 10.1016/j.physbeh.2008.11.011. Epub 2008 Nov 27.
Animal
93
Stefanidis A, Verty AN, Allen AM, Owens NC, Cowley MA, Oldfield BJ. The role of thermogenesis in antipsychotic drug-induced weight gain. Obesity (Silver Spring). 2009;17(1):16-24. doi: 10.1038/oby.2008.468. Epub 2008 Oct 30.

Animal
94
Walton ME, Groves J, Jennings KA, Croxson PL, Sharp T, Rushworth MF, Bannerman DM. Comparing the role of the anterior cingulate cortex and 6-hydroxydopamine nucleus accumbens lesions on operant effort-based decision making. Eur J Neurosci. 2009;29(8):1678-91. doi: 10.1111/j.1460-9568.2009.06726.x.
Animal
95
Jänsch C, Harmer C, Cooper MJ. Emotional processing in women with anorexia nervosa and in healthy volunteers. Eat Behav. 2009;10(3):184-91. doi: 10.1016/j.eatbeh.2009.06.001. Epub 2009 Jun 11.
Unrelated
96
Oliver PL, Davies KE. Interaction between environmental and genetic factors modulates schizophrenic endophenotypes in the Snap-25 mouse mutant blind-drunk. Hum Mol Genet. 2009;18(23):4576-89. doi: 10.1093/hmg/ddp425. Epub 2009 Sep 3.
Animal
97
Simons G, Ellgring JH, Beck-Dossler K, Gaebel W, Wölwer W. Facial expression in male and female schizophrenia patients. Eur Arch Psychiatry Clin Neurosci. 2010;260(3):267-76. doi: 10.1007/s00406-009-0074-5. Epub 2009 Oct 9.
No orexin
98
Borgland SL, Labouèbe G. Orexin/hypocretin in psychiatric disorders: present state of knowledge and future potential. Neuropsychopharmacology. 2010;35(1):353-4. doi: 10.1038/npp.2009.119.
Review
99
Suzuki G, Satow A, Ohta H. Effect of CFMTI, an allosteric metabotropic glutamate receptor 1 antagonist with antipsychotic activity, on Fos expression in regions of the brain related to schizophrenia. Neuroscience. 2010;168(3):787-96. doi: 10.1016/j.neuroscience.2010.04.016. Epub 2010 Apr 18.
Animal
**100**
**Basoglu C, Oner O, Gunes C, Semiz UB, Ates AM, Algul A, Ebrinc S, Cetin M, Ozcan O, Ipcioglu O. Plasma orexin A, ghrelin, cholecystokinin, visfatin, leptin and agouti-related protein levels during 6-week olanzapine treatment in first-episode male patients with psychosis. Int Clin Psychopharmacol. 2010;25(3):165-71. doi: 10.1097/YIC.0b013e3283377850.**
**Included**
101
Milella MS, Passarelli F, De Carolis L, Schepisi C, Nativio P, Scaccianoce S, Nencini P. Opposite roles of dopamine and orexin in quinpirole-induced excessive drinking: a rat model of psychotic polydipsia. Psychopharmacology (Berl). 2010;211(3):355-66. doi: 10.1007/s00213-010-1909-5. Epub 2010 Jun 16.
Animal
102
Martins PJ, Haas M, Obici S. Central nervous system delivery of the antipsychotic olanzapine induces hepatic insulin resistance. Diabetes. 2010;59(10):2418-25. doi: 10.2337/db10-0449. Epub 2010 Aug 3.
Animal
103
Mori K, Kim J, Sasaki K. Electrophysiological effects of orexin-B and dopamine on rat nucleus accumbens shell neurons in vitro. Peptides. 2011;32(2):246-52. doi: 10.1016/j.peptides.2010.10.023. Epub 2010 Nov 3.
Animal
104
Burgess CR, Tse G, Gillis L, Peever JH. Dopaminergic regulation of sleep and cataplexy in a murine model of narcolepsy. Sleep. 2010;33(10):1295-304. doi: 10.1093/sleep/33.10.1295.
Animal
105
Cao J, de Lecea L, Ikemoto S. Intraventricular administration of neuropeptide S has reward-like effects. Eur J Pharmacol. 2011;658(1):16-21. doi: 10.1016/j.ejphar.2011.02.009. Epub 2011 Feb 22.
Animal
106
Ito R, Hayen A. Opposing roles of nucleus accumbens core and shell dopamine in the modulation of limbic information processing. J Neurosci. 2011;31(16):6001-7. doi: 10.1523/JNEUROSCI.6588-10.2011.
Animal
107
Sasaki K, Suzuki M, Mieda M, Tsujino N, Roth B, Sakurai T. Pharmacogenetic modulation of orexin neurons alters sleep/wakefulness states in mice. PLoS One. 2011;6(5):e20360. doi: 10.1371/journal.pone.0020360. Epub 2011 May 27.
Animal
108
Fernø J, Varela L, Skrede S, Vázquez MJ, Nogueiras R, Diéguez C, Vidal-Puig A, Steen VM, López M. Olanzapine-induced hyperphagia and weight gain associate with orexigenic hypothalamic neuropeptide signaling without concomitant AMPK phosphorylation. PLoS One. 2011;6(6):e20571. doi: 10.1371/journal.pone.0020571. Epub 2011 Jun 13.
Animal
109
Jeans A, Malins R, Padamsey Z, Reinhart M, Emptage N. Increased expression of dysbindin-1A leads to a selective deficit in NMDA receptor signaling in the hippocampus. Neuropharmacology. 2011;61(8):1345-53. doi: 10.1016/j.neuropharm.2011.08.007. Epub 2011 Aug 16.
Animal
110
Oliver PL, Sobczyk MV, Maywood ES, Edwards B, Lee S, Livieratos A, Oster H, Butler R, Godinho SI, Wulff K, Peirson SN, Fisher SP, Chesham JE, Smith JW, Hastings MH, Davies KE, Foster RG. Disrupted circadian rhythms in a mouse model of schizophrenia. Curr Biol. 2012;22(4):314-9. doi: 10.1016/j.cub.2011.12.051. Epub 2012 Jan 19.
Animal
111
Barkus C, Dawson LA, Sharp T, Bannerman DM. GluN1 hypomorph mice exhibit wide-ranging behavioral alterations. Genes Brain Behav. 2012;11(3):342-51. doi: 10.1111/j.1601-183X.2012.00767.x. Epub 2012 Feb 9.
Animal

112
Ioachimescu OC, El-Solh AA. Pharmacotherapy of insomnia. Expert Opin Pharmacother. 2012;13(9):1243-60. doi: 10.1517/14656566.2012.683860. Epub 2012 May 11.
Review
113
Ma J, Tai SK, Leung LS. Septohippocampal GABAergic neurons mediate the altered behaviors induced by n-methyl-D-aspartate receptor antagonists. Hippocampus. 2012;22(12):2208-18. doi: 10.1002/hipo.22039. Epub 2012 May 17.
Animal
114
Jiang R, Song X, Bali P, Smith A, Bayona CR, Lin L, Cameron MD, McDonald PH, Kenny PJ, Kamenecka TM. Disubstituted piperidines as potent orexin (hypocretin) receptor antagonists. Bioorg Med Chem Lett. 2012;22(12):3890-4. doi: 10.1016/j.bmcl.2012.04.122. Epub 2012 May 4.
*In vitro*
115
Bradshaw CM, Killeen PR. A theory of behaviour on progressive ratio schedules, with applications in behavioural pharmacology. Psychopharmacology (Berl). 2012;222(4):549-64. doi: 10.1007/s00213-012-2771-4. Epub 2012 Jul 3.
Review
116
Vasudevan SR, Moore JB, Schymura Y, Churchill GC. Shape-based reprofiling of FDA-approved drugs for the $H_1$ histamine receptor. J Med Chem. 2012;55(16):7054-60. doi: 10.1021/jm300671m. Epub 2012 Aug 6.
*In vitro*
117
Taslimi Z, Arezoomandan R, Omranifard A, Ghalandari-Shamami M, Riahi E, Vafaei AA, Rashidy-Pour A, Haghparast A. Orexin A in the ventral tegmental area induces conditioned place preference in a dose-dependent manner: involvement of D1/D2 receptors in the nucleus accumbens. Peptides. 2012;37(2):225-32. doi: 10.1016/j.peptides.2012.07.023. Epub 2012 Aug 3.
Animal
118
Ikeda H, Kamei J, Koshikawa N, Cools AR. Nucleus accumbens and dopamine-mediated turning behavior of the rat: role of accumbal non-dopaminergic receptors. J Pharmacol Sci. 2012;120(3):152-64. doi: 10.1254/jphs.12r02cr. Epub 2012 Oct 10.
Animal
119
Hoyer D, Bartfai T. Neuropeptides and neuropeptide receptors: drug targets, and peptide and non-peptide ligands: a tribute to Prof. Dieter Seebach. Chem Biodivers. 2012;9(11):2367-87. doi: 10.1002/cbdv.201200288.
Review
120
Hoerder-Suabedissen A, Oeschger FM, Krishnan ML, Belgard TG, Wang WZ, Lee S, Webber C, Petretto E, Edwards AD, Molnár Z. Expression profiling of mouse subplate reveals a dynamic gene network and disease association with autism and schizophrenia. Proc Natl Acad Sci U S A. 2013;110(9):3555-60. doi: 10.1073/pnas.1218510110. Epub 2013 Feb 11.
Animal
121
Li X, Johnson MS, Smith DL Jr, Li Y, Kesterson RA, Allison DB, Nagy TR. Effects of risperidone on energy balance in female C57BL/6J mice. Obesity (Silver Spring). 2013;21(9):1850-7. doi: 10.1002/oby.20350. Epub 2013 May 29.
Animal
122
LaCrosse AL, Olive MF. Neuropeptide systems and schizophrenia. CNS Neurol Disord Drug Targets. 2013;12(5):619-32. doi: 10.2174/1871527311312050010.
Review
123
Gozzi A, Lepore S, Vicentini E, Merlo-Pich E, Bifone A. Differential effect of orexin-1 and CRF-1 antagonism on stress circuits: a fMRI study in the rat with the pharmacological stressor Yohimbine. Neuropsychopharmacology. 2013;38(11):2120-30. doi: 10.1038/npp.2013.109. Epub 2013 May 8.
Animal
124
Ortega-Roldan JL, Ossa F, Schnell JR. Characterization of the human sigma-1 receptor chaperone domain structure and binding immunoglobulin protein (BiP) interactions. J Biol Chem. 2013;288(29):21448-21457. doi: 10.1074/jbc.M113.450379. Epub 2013 Jun 12.
*In vitro*
125
Girault EM, Foppen E, Ackermans MT, Fliers E, Kalsbeek A. Central administration of an orexin receptor 1 antagonist prevents the stimulatory effect of Olanzapine on endogenous glucose production. Brain Res. 2013;1527:238-45. doi: 10.1016/j.brainres.2013.06.034. Epub 2013 Jul 4.
Animal
126
Haghparast A, Omranifard A, Arezoomandan R, Ghalandari-Shamami M, Taslimi Z, Vafaei AA, Rashidy-Pour A. Involvement of dopaminergic receptors of the rat nucleus accumbens in decreasing the conditioned place preference induced by lateral hypothalamus stimulation. Neurosci Lett. 2013;556:10-4. doi: 10.1016/j.neulet.2013.09.062. Epub 2013 Oct 5.
Animal
127
Teske JA, Billington CJ, Kotz CM. Mechanisms underlying obesity resistance associated with high spontaneous physical activity. Neuroscience. 2014;256:91-100. doi: 10.1016/j.neuroscience.2013.10.028. Epub 2013 Oct 22.
Animal
128
Chen YW, Morganstern I, Barson JR, Hoebel BG, Leibowitz SF. Differential role of D1 and D2 receptors in the perifornical lateral hypothalamus in controlling ethanol drinking and food intake: possible interaction with local orexin neurons. Alcohol Clin Exp Res. 2014;38(3):777-86. doi: 10.1111/acer.12313. Epub 2013 Nov 15.
Animal
**129**
**Huang YS, Guilleminault C, Chen CH, Lai PC, Hwang FM. Narcolepsy-cataplexy and schizophrenia in adolescents. Sleep Med. 2014;15(1):15-22. doi: 10.1016/j.sleep.2013.09.018. Epub 2013 Oct 26.**
**Included**
130

Brandler WM, Paracchini S. The genetic relationship between handedness and neurodevelopmental disorders. Trends Mol Med. 2014;20(2):83-90. doi: 10.1016/j.molmed.2013.10.008. Epub 2013 Nov 23.
Review
131
Brown R, Taylor MJ, Geddes J. Aripiprazole alone or in combination for acute mania. Cochrane Database Syst Rev. 2013;2013(12):CD005000. doi: 10.1002/14651858.CD005000.pub2.
Review
132
Pizza F, Magnani M, Indrio C, Plazzi G. The hypocretin system and psychiatric disorders. Curr Psychiatry Rep. 2014;16(2):433. doi: 10.1007/s11920-013-0433-9.
Review
133
Hasegawa E, Yanagisawa M, Sakurai T, Mieda M. Orexin neurons suppress narcolepsy via 2 distinct efferent pathways. J Clin Invest. 2014;124(2):604-16. doi: 10.1172/JCI71017. Epub 2014 Jan 2.
Animal
134
Hayen A, Meese-Tamuri S, Gates A, Ito R. Opposing roles of prelimbic and infralimbic dopamine in conditioned cue and place preference. Psychopharmacology (Berl). 2014;231(12):2483-92. doi: 10.1007/s00213-013-3414-0. Epub 2014 Jan 16.
Animal
135
Ekert A, Renner R. The ultimate physical limits of privacy. Nature. 2014;507(7493):443-7. doi: 10.1038/nature13132.
Unrelated
136
Thompson MD, Xhaard H, Sakurai T, Rainero I, Kukkonen JP. OX1 and OX2 orexin/hypocretin receptor pharmacogenetics. Front Neurosci. 2014;8:57. doi: 10.3389/fnins.2014.00057.
Review
137
Palotai M, Telegdy G, Jászberényi M. Orexin A-induced anxiety-like behavior is mediated through GABA-ergic, α- and β-adrenergic neurotransmissions in mice. Peptides. 2014;57:129-34. doi: 10.1016/j.peptides.2014.05.003. Epub 2014 May 27.
Animal
138
Palotai M, Telegdy G, Ekwerike A, Jászberényi M. The action of orexin B on passive avoidance learning. Involvement of neurotransmitters. Behav Brain Res. 2014;272:1-7. doi: 10.1016/j.bbr.2014.06.016. Epub 2014 Jun 13.
Animal
139
Inutsuka A, Inui A, Tabuchi S, Tsunematsu T, Lazarus M, Yamanaka A. Concurrent and robust regulation of feeding behaviors and metabolism by orexin neurons. Neuropharmacology. 2014;85:451-60. doi: 10.1016/j.neuropharm.2014.06.015. Epub 2014 Jun 18.
Animal
140
Kolaj M, Zhang L, Renaud LP. Novel coupling between TRPC-like and KNa channels modulates low threshold spike-induced afterpotentials in rat thalamic midline neurons. Neuropharmacology. 2014;86:88-96. doi: 10.1016/j.neuropharm.2014.06.023. Epub 2014 Jul 9.
Animal
141
Schweimer JV, Coullon GS, Betts JF, Burnet PW, Engle SJ, Brandon NJ, Harrison PJ, Sharp T. Increased burst-firing of ventral tegmental area dopaminergic neurons in D-amino acid oxidase knockout mice in vivo. Eur J Neurosci. 2014;40(7):2999-3009. doi: 10.1111/ejn.12667. Epub 2014 Jul 5.
Animal
142
Yazdi-Ravandi S, Razavi Y, Haghparast A, Goudarzvand M, Haghparast A. Orexin A induced antinociception in the ventral tegmental area involves D1 and D2 receptors in the nucleus accumbens. Pharmacol Biochem Behav. 2014;126:1-6. doi: 10.1016/j.pbb.2014.08.009. Epub 2014 Aug 30.
Animal
**143**
**Chien YL, Liu CM, Shan JC, Lee HJ, Hsieh MH, Hwu HG, Chiou LC. Elevated plasma orexin A levels in a subgroup of patients with schizophrenia associated with fewer negative and disorganized symptoms. Psychoneuroendocrinology. 2015;53:1-9. doi: 10.1016/j.psyneuen.2014.12.012. Epub 2014 Dec 20.**
**Included**
144
Rojczyk E, Pałasz A, Wiaderkiewicz R. Effect of short and long-term treatment with antipsychotics on orexigenic/anorexigenic neuropeptides expression in the rat hypothalamus. Neuropeptides. 2015;51:31-42. doi: 10.1016/j.npep.2015.04.001. Epub 2015 Apr 3.
Animal
145
Pritchett D, Jagannath A, Brown LA, Tam SK, Hasan S, Gatti S, Harrison PJ, Bannerman DM, Foster RG, Peirson SN. Deletion of metabotropic glutamate receptors 2 and 3 (mGlu2 & mGlu3) in mice disrupts sleep and wheel-running activity, and increases the sensitivity of the circadian system to light. PLoS One. 2015;10(5):e0125523. doi: 10.1371/journal.pone.0125523.
Animal
146
Rocca FL, Pizza F, Ricci E, Plazzi G. Narcolepsy during childhood: An update. Neuropediatrics. 2015;46(3):181-98. doi: 10.1055/s-0035-1550152. Epub 2015 May 11.
Review
147
Li AJ, Wang Q, Elsarelli MM, Brown RL, Ritter S. Hindbrain catecholamine neurons activate orexin neurons during systemic glucoprivation in male rats. Endocrinology. 2015;156(8):2807-20. doi: 10.1210/en.2015-1138. Epub 2015 May 15.
Animal
148
Freeman D, Sheaves B, Goodwin GM, Yu LM, Harrison PJ, Emsley R, Bostock S, Foster RG, Wadekar V, Hinds C, Espie CA. Effects of cognitive behavioural therapy for insomnia on the mental health of university students: study protocol for a randomized controlled trial. Trials. 2015;16:236. doi: 10.1186/s13063-015-0756-4.
Protocol

149
Moradi M, Fatahi Z, Haghparast A. Blockade of D1-like dopamine receptors within the ventral tegmental area and nucleus accumbens attenuates antinociceptive responses induced by chemical stimulation of the lateral hypothalamus. Neurosci Lett. 2015;599:61-6. doi: 10.1016/j.neulet.2015.05.047. Epub 2015 May 26.
Animal
150
Ouhaz Z, Ba-M'hamed S, Mitchell AS, Elidrissi A, Bennis M. Behavioral and cognitive changes after early postnatal lesions of the rat mediodorsal thalamus. Behav Brain Res. 2015;292:219-32. doi: 10.1016/j.bbr.2015.06.017. Epub 2015 Jun 12.
Animal
151
Ganzetti M, Wenderoth N, Mantini D. Mapping pathological changes in brain structure by combining T1- and T2-weighted MR imaging data. Neuroradiology. 2015;57(9):917-28. doi: 10.1007/s00234-015-1550-4. Epub 2015 Jun 24.
Unrelated
152
Ma J, Domicevica L, Schnell JR, Biggin PC. Position and orientational preferences of drug-like compounds in lipid membranes: a computational and NMR approach. Phys Chem Chem Phys. 2015;17(30):19766-76. doi: 10.1039/c5cp03218k.
Unrelated
153
Moradi M, Yazdanian M, Haghparast A. Role of dopamine D2-like receptors within the ventral tegmental area and nucleus accumbens in antinociception induced by lateral hypothalamus stimulation. Behav Brain Res. 2015;292:508-14. doi: 10.1016/j.bbr.2015.07.007. Epub 2015 Jul 9.
Animal
154
Jacobs BM. A dangerous method? The use of induced pluripotent stem cells as a model for schizophrenia. Schizophr Res. 2015;168(1-2):563-8. doi: 10.1016/j.schres.2015.07.005. Epub 2015 Jul 17.
Unrelated
155
Majercikova Z, Kiss A. Effect of asenapine on the activity of hypocretin neurons in normal and unpredictable mild stress preconditioned rats. Folia Biol (Praha). 2015;61(3):110-5. doi: 10.14712/fb2015061030110.
Animal
156
Okumura T, Nozu T, Kumei S, Takakusaki K, Miyagishi S, Ohhira M. Involvement of the dopaminergic system in the central orexin-induced antinociceptive action against colonic distension in conscious rats. Neurosci Lett. 2015;605:34-8. doi: 10.1016/j.neulet.2015.08.013. Epub 2015 Aug 12.
Animal
157
Nishizawa D, Kasai S, Hasegawa J, Sato N, Yamada H, Tanioka F, Nagashima M, Katoh R, Satoh Y, Tagami M, Ujike H, Ozaki N, Inada T, Iwata N, Sora I, Iyo M, Yamada M, Kondo N, Won MJ, Naruse N, Uehara-Aoyama K, Itokawa M, Ohi K, Hashimoto R, Tanisawa K, Arai T, Mori S, Sawabe M, Naka-Mieno M, Yamada Y, Yamada M, Sato N, Muramatsu M, Tanaka M, Irukayama-Tomobe Y, Saito YC, Sakurai T, Hayashida M, Sugimura H, Ikeda K. Associations between the orexin (hypocretin) receptor 2 gene polymorphism Val308Ile and nicotine dependence in genome-wide and subsequent association studies. Mol Brain. 2015;8:50. doi: 10.1186/s13041-015-0142-x.
No psychosis
158
Krystal AD. Current, emerging, and newly available insomnia medications. J Clin Psychiatry. 2015;76(8):e1045. doi: 10.4088/JCP.14046tx2c.
Review
159
Parrott M, Koralus P. The erotetic theory of delusional thinking. Cogn Neuropsychiatry. 2015;20(5):398-415. doi: 10.1080/13546805.2015.1067601. Epub 2015 Sep 14. Erratum in: Cogn Neuropsychiatry. 2015;20(6):555. doi: 10.1080/13546805.2015.1126136.
Unrelated
**160**
**Tiwari AK, Brandl EJ, Zai CC, Goncalves VF, Chowdhury NI, Freeman N, Lieberman JA, Meltzer HY, Kennedy JL, Müller DJ. Association of orexin receptor polymorphisms with antipsychotic-induced weight gain. World J Biol Psychiatry. 2016;17(3):221-9. doi: 10.3109/15622975.2015.1076173. Epub 2015 Oct 8.**
**Included**
161
Barandas R, Landgraf D, McCarthy MJ, Welsh DK. Circadian clocks as modulators of metabolic comorbidity in psychiatric disorders. Curr Psychiatry Rep. 2015;17(12):98. doi: 10.1007/s11920-015-0637-2.
Review
162
Vickers SP, Hackett D, Murray F, Hutson PH, Heal DJ. Effects of lisdexamfetamine in a rat model of binge-eating. J Psychopharmacol. 2015;29(12):1290-307. doi: 10.1177/0269881115615107. Epub 2015 Nov 20.
Animal
163
Boss C, Roch C. Substituted cyclopentanes, tetrahydrofurans and pyrrolidines as orexin-1-receptor antagonists for treatment of various CNS disorders (WO2015/055994; WO2015/124932; WO2015/124934). Expert Opin Ther Pat. 2016;26(3):409-15. doi: 10.1517/13543776.2016.1124087. Epub 2015 Dec 19.
*In vitro*
164
Krystal AD. New developments in insomnia medications of relevance to mental health disorders. Psychiatr Clin North Am. 2015;38(4):843-60. doi: 10.1016/j.psc.2015.08.001. Epub 2015 Sep 11.
Review
165
Hayward A, Tomlinson A, Neill JC. Low attentive and high impulsive rats: A translational animal model of ADHD and disorders of attention and impulse control. Pharmacol Ther. 2016;158:41-51. doi: 10.1016/j.pharmthera.2015.11.010. Epub 2015 Nov 23.
Review
166
**Sun HQ, Li SX, Chen FB, Zhang Y, Li P, Jin M, Sun Y, Wang F, Mi WF, Shi L, Yue JL, Yang FD, Lu L. Diurnal neurobiological alterations after exposure to clozapine in first-episode schizophrenia patients. Psychoneuroendocrinology. 2016;64:108-16. doi: 10.1016/j.psyneuen.2015.11.013. Epub 2015 Nov 26.**

**Included**
167
Pritchett D, Taylor AM, Barkus C, Engle SJ, Brandon NJ, Sharp T, Foster RG, Harrison PJ, Peirson SN, Bannerman DM. Searching for cognitive enhancement in the Morris water maze: better and worse performance in D-amino acid oxidase knockout (*Dao*$^{-/-}$) mice. Eur J Neurosci. 2016;43(7):979-89. doi: 10.1111/ejn.13192. Epub 2016 Mar 23.
Animal
168
Okumura T, Nozu T, Kumei S, Takakusaki K, Miyagishi S, Ohhira M. Levodopa acts centrally to induce an antinociceptive action against colonic distension through activation of D2 dopamine receptors and the orexinergic system in the brain in conscious rats. J Pharmacol Sci. 2016;130(2):123-7. doi: 10.1016/j.jphs.2016.01.007. Epub 2016 Jan 29.
Animal
169
Majercikova Z, Kiss A. Stress alters asenapine-induced Fos expression in the Meynert's nucleus: response of adjacent hypocretin and melanin-concentrating hormone neurons in rat. Neurol Res. 2016;38(1):32-9. doi: 10.1080/01616412.2015.1105585. Epub 2016 Feb 19.
Animal
170
Gilmour G, Gastambide F, Marston HM, Walton ME. Using intermediate cognitive endpoints to facilitate translational research in psychosis. Curr Opin Behav Sci. 2015;4:128-135. doi: 10.1016/j.cobeha.2015.04.011.
Review
171
Wagner L, Kaestner F, Wolf R, Stiller H, Heiser U, Manhart S, Hoffmann T, Rahfeld JU, Demuth HU, Rothermundt M, von Hörsten S. Identifying neuropeptide Y (NPY) as the main stress-related substrate of dipeptidyl peptidase 4 (DPP4) in blood circulation. Neuropeptides. 2016;57:21-34. doi: 10.1016/j.npep.2016.02.007. Epub 2016 Feb 27.
No psychosis
172
Sum-Ping O, Guilleminault C. Kleine-Levin Syndrome. Curr Treat Options Neurol. 2016;18(6):24. doi: 10.1007/s11940-016-0409-2.
Review
173
Sheaves B, Bebbington PE, Goodwin GM, Harrison PJ, Espie CA, Foster RG, Freeman D. Insomnia and hallucinations in the general population: Findings from the 2000 and 2007 British Psychiatric Morbidity Surveys. Psychiatry Res. 2016;241:141-6. doi: 10.1016/j.psychres.2016.03.055. Epub 2016 Apr 26.
No orexin
174
Okbay A, Beauchamp JP, Fontana MA, Lee JJ, Pers TH, Rietveld CA, Turley P, Chen GB, Emilsson V, Meddens SF, Oskarsson S, Pickrell JK, Thom K, Timshel P, de Vlaming R, Abdellaoui A, Ahluwalia TS, Bacelis J, Baumbach C, Bjornsdottir G, Brandsma JH, Pina Concas M, Derringer J, Furlotte NA, Galesloot TE, Girotto G, Gupta R, Hall LM, Harris SE, Hofer E, Horikoshi M, Huffman JE, Kaasik K, Kalafati IP, Karlsson R, Kong A, Lahti J, van der Lee SJ, deLeeuw C, Lind PA, Lindgren KO, Liu T, Mangino M, Marten J, Mihailov E, Miller MB, van der Most PJ, Oldmeadow C, Payton A, Pervjakova N, Peyrot WJ, Qian Y, Raitakari O, Rueedi R, Salvi E, Schmidt B, Schraut KE, Shi J, Smith AV, Poot RA, St Pourcain B, Teumer A, Thorleifsson G, Verweij N, Vuckovic D, Wellmann J, Westra HJ, Yang J, Zhao W, Zhu Z, Alizadeh BZ, Amin N, Bakshi A, Baumeister SE, Biino G, Bønnelykke K, Boyle PA, Campbell H, Cappuccio FP, Davies G, De Neve JE, Deloukas P, Demuth I, Ding J, Eibich P, Eisele L, Eklund N, Evans DM, Faul JD, Feitosa MF, Forstner AJ, Gandin I, Gunnarsson B, Halldórsson BV, Harris TB, Heath AC, Hocking LJ, Holliday EG, Homuth G, Horan MA, Hottenga JJ, de Jager PL, Joshi PK, Jugessur A, Kaakinen MA, Kähönen M, Kanoni S, Keltigangas-Järvinen L, Kiemeney LA, Kolcic I, Koskinen S, Kraja AT, Kroh M, Kutalik Z, Latvala A, Launer LJ, Lebreton MP, Levinson DF, Lichtenstein P, Lichtner P, Liewald DC; LifeLines Cohort Study; Loukola A, Madden PA, Mägi R, Mäki-Opas T, Marioni RE, Marques-Vidal P, Meddens GA, McMahon G, Meisinger C, Meitinger T, Milaneschi Y, Milani L, Montgomery GW, Myhre R, Nelson CP, Nyholt DR, Ollier WE, Palotie A, Paternoster L, Pedersen NL, Petrovic KE, Porteous DJ, Räikkönen K, Ring SM, Robino A, Rostapshova O, Rudan I, Rustichini A, Salomaa V, Sanders AR, Sarin AP, Schmidt H, Scott RJ, Smith BH, Smith JA, Staessen JA, Steinhagen-Thiessen E, Strauch K, Terracciano A, Tobin MD, Ulivi S, Vaccargiu S, Quaye L, van Rooij FJ, Venturini C, Vinkhuyzen AA, Völker U, Völzke H, Vonk JM, Vozzi D, Waage J, Ware EB, Willemsen G, Attia JR, Bennett DA, Berger K, Bertram L, Bisgaard H, Boomsma DI, Borecki IB, Bültmann U, Chabris CF, Cucca F, Cusi D, Deary IJ, Dedoussis GV, van Duijn CM, Eriksson JG, Franke B, Franke L, Gasparini P, Gejman PV, Gieger C, Grabe HJ, Gratten J, Groenen PJ, Gudnason V, van der Harst P, Hayward C, Hinds DA, Hoffmann W, Hyppönen E, Iacono WG, Jacobsson B, Järvelin MR, Jöckel KH, Kaprio J, Kardia SL, Lehtimäki T, Lehrer SF, Magnusson PK, Martin NG, McGue M, Metspalu A, Pendleton N, Penninx BW, Perola M, Pirastu N, Pirastu M, Polasek O, Posthuma D, Power C, Province MA, Samani NJ, Schlessinger D, Schmidt R, Sørensen TI, Spector TD, Stefansson K, Thorsteinsdottir U, Thurik AR, Timpson NJ, Tiemeier H, Tung JY, Uitterlinden AG, Vitart V, Vollenweider P, Weir DR, Wilson JF, Wright AF, Conley DC, Krueger RF, Davey Smith G, Hofman A, Laibson DI, Medland SE, Meyer MN, Yang J, Johannesson M, Visscher PM, Esko T, Koellinger PD, Cesarini D, Benjamin DJ. Genome-wide association study identifies 74 loci associated with educational attainment. Nature. 2016;533(7604):539-42. doi: 10.1038/nature17671. Epub 2016 May 11.
No orexin
**175**
**Sansa G, Gavaldà A, Gaig C, Monreal J, Ercilla G, Casamitjana R, Ribera G, Iranzo A, Santamaria J. Exploring the presence of narcolepsy in patients with schizophrenia. BMC Psychiatry. 2016;16:177. doi: 10.1186/s12888-016-0859-9.**
**Included**
176
Xue Y, Yang YT, Liu HY, Chen WF, Chen AQ, Sheng Q, Chen XY, Wang Y, Chen H, Liu HX, Pang YY, Chen L. Orexin-A increases the activity of globus pallidus neurons in both normal and parkinsonian rats. Eur J Neurosci. 2016;44(5):2247-57. doi: 10.1111/ejn.13323. Epub 2016 Jul 13.
Animal
177
Bakken TE, Miller JA, Ding SL, Sunkin SM, Smith KA, Ng L, Szafer A, Dalley RA, Royall JJ, Lemon T, Shapouri S, Aiona K, Arnold J, Bennett JL, Bertagnolli D, Bickley K, Boe A, Brouner K, Butler S, Byrnes E, Caldejon S, Carey A, Cate S, Chapin M, Chen J, Dee N, Desta T, Dolbeare TA, Dotson N, Ebbert A, Fulfs E, Gee G, Gilbert TL, Goldy J, Gourley L, Gregor B, Gu G, Hall J, Haradon Z, Haynor DR, Hejazinia N, Hoerder-Suabedissen A, Howard R, Jochim J, Kinnunen M, Kriedberg A, Kuan CL, Lau C, Lee CK, Lee F, Luong L, Mastan N, May R, Melchor J, Mosqueda N, Mott E, Ngo K, Nyhus J, Oldre A, Olson E, Parente J, Parker PD, Parry S, Pendergraft J, Potekhina L, Reding M, Riley ZL, Roberts T, Rogers B, Roll K, Rosen D, Sandman D, Sarreal M, Shapovalova N, Shi S, Sjoquist N, Sodt AJ, Townsend R, Velasquez L, Wagley U, Wakeman WB, White C, Bennett C, Wu J, Young R, Youngstrom BL, Wohnoutka P, Gibbs RA, Rogers J, Hohmann JG, Hawrylycz MJ, Hevner RF, Molnár Z, Phillips JW, Dang C, Jones AR, Amaral DG, Bernard A, Lein ES. A comprehensive transcriptional map of primate brain development. Nature. 2016;535(7612):367-75. doi: 10.1038/nature18637. Epub 2016 Jul 13.
Animal
178
Sharpee TO, Destexhe A, Kawato M, Sekulić V, Skinner FK, Wójcik DK, Chintaluri C, Cserpán D, Somogyvári Z, Kim JK, Kilpatrick ZP, Bennett MR, Josić K, Elices I, Arroyo D, Levi R, Rodriguez FB, Varona P, Hwang E, Kim B, Han HB, Kim T, McKenna JT, Brown RE, McCarley RW, Choi JH, Rankin J, Popp PO, Rinzel J, Tabas A, Rupp A, Balaguer-Ballester E, Maturana MI, Grayden DB, Cloherty SL, Kameneva T, Ibbotson MR, Meffin H, Koren V,

Lochmann T, Dragoi V, Obermayer K, Psarrou M, Schilstra M, Davey N, Torben-Nielsen B, Steuber V, Ju H, Yu J, Hines ML, Chen L, Yu Y, Kim J, Leahy W, Shlizerman E, Birgiolas J, Gerkin RC, Crook SM, Viriyopase A, Memmesheimer RM, Gielen S, Dabaghian Y, DeVito J, Perotti L, Kim AJ, Fenk LM, Cheng C, Maimon G, Zhao C, Widmer Y, Sprecher S, Senn W, Halnes G, Mäki-Marttunen T, Keller D, Pettersen KH, Andreassen OA, Einevoll GT, Yamada Y, Steyn-Ross ML, Alistair Steyn-Ross D, Mejias JF, Murray JD, Kennedy H, Wang XJ, Kruscha A, Grewe J, Benda J, Lindner B, Badel L, Ohta K, Tsuchimoto Y, Kazama H, Kahng B, Tam ND, Pollonini L, Zouridakis G, Soh J, Kim D, Yoo M, Palmer SE, Culmone V, Bojak I, Ferrario A, Merrison-Hort R, Borisyuk R, Kim CS, Tezuka T, Joo P, Rho YA, Burton SD, Bard Ermentrout G, Jeong J, Urban NN, Marsalek P, Kim HH, Moon SH, Lee DW, Lee SB, Lee JY, Molkov YI, Hamade K, Teka W, Barnett WH, Kim T, Markin S, Rybak IA, Forro C, Dermutz H, Demkó L, Vörös J, Babichev A, Huang H, Verduzco-Flores S, Dos Santos F, Andras P, Metzner C, Schweikard A, Zurowski B, Roach JP, Sander LM, Zochowski MR, Skilling QM, Ognjanovski N, Aton SJ, Zochowski M, Wang SJ, Ouyang G, Guang J, Zhang M, Michael Wong KY, Zhou C, Robinson PA, Sanz-Leon P, Drysdale PM, Fung F, Abeysuriya RG, Rennie CJ, Zhao X, Choe Y, Yang HF, Mi Y, Lin X, Wu S, Liedtke J, Schottdorf M, Wolf F, Yamamura Y, Wickens JR, Rumbell T, Ramsey J, Reyes A, Draguljić D, Hof PR, Luebke J, Weaver CM, He H, Yang X, Ma H, Xu Z, Wang Y, Baek K, Morris LS, Kundu P, Voon V, Agnes EJ, Vogels TP, Podlaski WF, Giese M, Kuravi P, Vogels R, Seeholzer A, Podlaski W, Ranjan R, Vogels T, Torres JJ, Baroni F, Latorre R, Gips B, Lowet E, Roberts MJ, de Weerd P, Jensen O, van der Eerden J, Goodarzinick A, Niry MD, Valizadeh A, Pariz A, Parsi SS, Warburton JM, Marucci L, Tamagnini F, Brown J, Tsaneva-Atanasova K, Kleberg FI, Triesch J, Moezzi B, Iannella N, Schaworonkow N, Plogmacher L, Goldsworthy MR, Hordacre B, McDonnell MD, Ridding MC, Zapotocky M, Smit D, Fouquet C, Trembleau A, Dasgupta S, Nishikawa I, Aihara K, Toyoizumi T, Robb DT, Mellen N, Toporikova N, Tang R, Tang YY, Liang G, Kiser SA, Howard JH Jr, Goncharenko J, Voronenko SO, Ahamed T, Stephens G, Yger P, Lefebvre B, Spampinato GLB, Esposito E, et Olivier Marre MS, Choi H, Song MH, Chung S, Lee DD, Sompolinsky H, Phillips RS, Smith J, Chatzikalymniou AP, Ferguson K, Alex Cayco Gajic N, Clopath C, Angus Silver R, Gleeson P, Marin B, Sadeh S, Quintana A, Cantarelli M, Dura-Bernal S, Lytton WW, Davison A, Li L, Zhang W, Wang D, Song Y, Park S, Choi I, Shin HS, Choi H, Pasupathy A, Shea-Brown E, Huh D, Sejnowski TJ, Vogt SM, Kumar A, Schmidt R, Van Wert S, Schiff SJ, Veale R, Scheutz M, Lee SW, Gallinaro J, Rotter S, Rubchinsky LL, Cheung CC, Ratnadurai-Giridharan S, Shomali SR, Ahmadabadi MN, Shimazaki H, Nader Rasuli S, Zhao X, Rasch MJ, Wilting J, Priesemann V, Levina A, Rudelt L, Lizier JT, Spinney RE, Rubinov M, Wibral M, Bak JH, Pillow J, Zaho Y, Park IM, Kang J, Park HJ, Jang J, Paik SB, Choi W, Lee C, Song M, Lee H, Park Y, Yilmaz E, Baysal V, Ozer M, Saska D, Nowotny T, Chan HK, Diamond A, Herrmann CS, Murray MM, Ionta S, Hutt A, Lefebvre J, Weidel P, Duarte R, Morrison A, Lee JH, Iyer R, Mihalas S, Koch C, Petrovici MA, Leng L, Breitwieser O, Stöckel D, Bytschok I, Martel R, Bill J, Schemmel J, Meier K, Esler TB, Burkitt AN, Kerr RR, Tahayori B, Nolte M, Reimann MW, Muller E, Markram H, Parziale A, Senatore R, Marcelli A, Skiker K, Maouene M, Neymotin SA, Seidenstein A, Lakatos P, Sanger TD, Menzies RJ, McLauchlan C, van Albada SJ, Kedziora DJ, Neymotin S, Kerr CC, Suter BA, Shepherd GMG, Ryu J, Lee SH, Lee J, Lee HJ, Lim D, Wang J, Lee H, Jung N, Anh Quang L, Maeng SE, Lee TH, Lee JW, Park CH, Ahn S, Moon J, Choi YS, Kim J, Jun SB, Lee S, Lee HW, Jo S, Jun E, Yu S, Goetze F, Lai PY, Kim S, Kwag J, Jang HJ, Filipović M, Reig R, Aertsen A, Silberberg G, Bachmann C, Buttler S, Jacobs H, Dillen K, Fink GR, Kukolja J, Kepple D, Giaffar H, Rinberg D, Shea S, Koulakov A, Bahuguna J, Tetzlaff T, Kotaleski JH, Kunze T, Peterson A, Knösche T, Kim M, Kim H, Park JS, Yeon JW, Kim SP, Kang JH, Lee C, Spiegler A, Petkoski S, Palva MJ, Jirsa VK, Saggio ML, Siep SF, Stacey WC, Bernar C, Choung OH, Jeong Y, Lee YI, Kim SH, Jeong M, Lee J, Kwon J, Kralik JD, Jahng J, Hwang DU, Kwon JH, Park SM, Kim S, Kim H, Kim PS, Yoon S, Lim S, Park C, Miller T, Clements K, Ahn S, Ji EH, Issa FA, Baek J, Oba S, Yoshimoto J, Doya K, Ishii S, Mosqueiro TS, Strube- Bloss MF, Smith B, Huerta R, Hadrava M, Hlinka J, Bos H, Helias M, Welzig CM, Harper ZJ, Kim WS, Shin IS, Baek HM, Han SK, Richter R, Vitay J, Beuth F, Hamker FH, Toppin K, Guo Y, Graham BP, Kale PJ, Gollo LL, Stern M, Abbott LF, Fedorov LA, Giese MA, Ardestani MH, Faraji MJ, Preuschoff K, Gerstner W, van Gendt MJ, Briaire JJ, Kalkman RK, Frijns JHM, Lee WH, Frangou S, Fulcher BD, Tran PHP, Fornito A, Gliske SV, Lim E, Holman KA, Fink CG, Kim JS, Mu S, Briggman KL, Sebastian Seung H; the EyeWirers; Wegener D, Bohnenkamp L, Ernst UA, Devor A, Dale AM, Lines GT, Edwards A, Tveito A, Hagen E, Senk J, Diesmann M, Schmidt M, Bakker R, Shen K, Bezgin G, Hilgetag CC, van Albada SJ, Sun H, Sourina O, Huang GB, Klanner F, Denk C, Glomb K, Ponce-Alvarez A, Gilson M, Ritter P, Deco G, Witek MAG, Clarke EF, Hansen M, Wallentin M, Kringelbach ML, Vuust P, Klingbeil G, De Schutter E, Chen W, Zang Y, Hong S, Takashima A, Zamora C, Gallimore AR, Goldschmidt D, Manoonpong P, Karoly PJ, Freestone DR, Soundry D, Kuhlmann L, Paninski L, Cook M, Lee J, Fishman YI, Cohen YE, Roberts JA, Cocchi L, Sweeney Y, Lee S, Jung WS, Kim Y, Jung Y, Song YK, Chavane F, Soman K, Muralidharan V, Srinivasa Chakravarthy V, Shivkumar S, Mandali A, Pragathi Priyadharsini B, Mehta H, Davey CE, Brinkman BAW, Kekona T, Rieke F, Buice M, De Pittà M, Berry H, Brunel N, Breakspear M, Marsat G, Drew J, Chapman PD, Daly KC, Bradle SP, Seo SB, Su J, Kavalali ET, Blackwell J, Shiau L, Buhry L, Basnayake K, Lee SH, Levy BA, Baker CI, Leleu T, Philips RT, Chhabria K. 25th Annual Computational Neuroscience Meeting: CNS-2016. BMC Neurosci. 2016;17 Suppl 1(Suppl 1):54. doi: 10.1186/s12868-016-0283-6.
Abstr

179

Sarkanen T, Alén R, Partinen M. Transient impact of rituximab in H1N1 vaccination-associated narcolepsy with severe psychiatric symptoms. Neurologist. 2016;21(5):85-6. doi: 10.1097/NRL.0000000000000099.
Case

180

Astle WJ, Elding H, Jiang T, Allen D, Ruklisa D, Mann AL, Mead D, Bouman H, Riveros-Mckay F, Kostadima MA, Lambourne JJ, Sivapalaratnam S, Downes K, Kundu K, Bomba L, Berentsen K, Bradley JR, Daugherty LC, Delaneau O, Freson K, Garner SF, Grassi L, Guerrero J, Haimel M, Janssen-Megens EM, Kaan A, Kamat M, Kim B, Mandoli A, Marchini J, Martens JHA, Meacham S, Megy K, O'Connell J, Petersen R, Sharifi N, Sheard SM, Staley JR, Tuna S, van der Ent M, Walter K, Wang SY, Wheeler E, Wilder SP, Iotchkova V, Moore C, Sambrook J, Stunnenberg HG, Di Angelantonio E, Kaptoge S, Kuijpers TW, Carrillo-de-Santa-Pau E, Juan D, Rico D, Valencia A, Chen L, Ge B, Vasquez L, Kwan T, Garrido-Martín D, Watt S, Yang Y, Guigo R, Beck S, Paul DS, Pastinen T, Bujold D, Bourque G, Frontini M, Danesh J, Roberts DJ, Ouwehand WH, Butterworth AS, Soranzo N. The allelic landscape of human blood cell trait variation and links to common complex disease. Cell. 2016;167(5):1415-1429.e19. doi: 10.1016/j.cell.2016.10.042.
No orexin

181

Boerner T, Bygrave AM, Chen J, Fernando A, Jackson S, Barkus C, Sprengel R, Seeburg PH, Harrison PJ, Gilmour G, Bannerman DM, Sanderson DJ. The group II metabotropic glutamate receptor agonist LY354740 and the D2 receptor antagonist haloperidol reduce locomotor hyperactivity but fail to rescue spatial working memory in GluA1 knockout mice. Eur J Neurosci. 2017;45(7):912-921. doi: 10.1111/ejn.13539. Epub 2017 Mar 4.
Animal

182

Mahoney CE, Agostinelli LJ, Brooks JN, Lowell BB, Scammell TE. GABAergic neurons of the central amygdala promote cataplexy. J Neurosci. 2017;37(15):3995-4006. doi: 10.1523/JNEUROSCI.4065-15.2017. Epub 2017 Feb 24.
Animal

183

Webber C. Epistasis in neuropsychiatric disorders. Trends Genet. 2017;33(4):256-265. doi: 10.1016/j.tig.2017.01.009. Epub 2017 Mar 6.
Review

184

Bunney PE, Zink AN, Holm AA, Billington CJ, Kotz CM. Orexin activation counteracts decreases in nonexercise activity thermogenesis (NEAT) caused by high-fat diet. Physiol Behav. 2017;176:139-148. doi: 10.1016/j.physbeh.2017.03.040. Epub 2017 Mar 28.
Animal

185

Giannoccaro MP, Waters P, Pizza F, Liguori R, Plazzi G, Vincent A. Antibodies against hypocretin receptor 2 are rare in narcolepsy. Sleep. 2017;40(2):zsw056. doi: 10.1093/sleep/zsw056.
No antipsychotics

186
Sanderson DJ, Lee A, Sprengel R, Seeburg PH, Harrison PJ, Bannerman DM. Altered balance of excitatory and inhibitory learning in a genetically modified mouse model of glutamatergic dysfunction relevant to schizophrenia. Sci Rep. 2017;7(1):1765. doi: 10.1038/s41598-017-01925-8.
Animal
187
Barron HC, Vogels TP, Behrens TE, Ramaswami M. Inhibitory engrams in perception and memory. Proc Natl Acad Sci U S A. 2017;114(26):6666-6674. doi: 10.1073/pnas.1701812114. Epub 2017 Jun 13.
Opinion
188
Matheson E, Hainer BL. Insomnia: Pharmacologic therapy. Am Fam Physician. 2017;96(1):29-35.
Review
189
Cañellas-Dols F, Delgado C, Arango-Lopez C, Peraita-Adrados R. Narcolepsy-cataplexy and psychosis: a case study. Rev Neurol. 2017;65(2):70-74. Spanish, English.
Case
190
Campbell EJ, Mitchell CS, Adams CD, Yeoh JW, Hodgson DM, Graham BA, Dayas CV. Chemogenetic activation of the lateral hypothalamus reverses early life stress-induced deficits in motivational drive. Eur J Neurosci. 2017;46(7):2285-2296. doi: 10.1111/ejn.13674. Epub 2017 Sep 22.
Animal
191
Thompson MD, Sakurai T, Rainero I, Maj MC, Kukkonen JP. Orexin receptor multimerization versus functional interactions: Neuropharmacological implications for opioid and cannabinoid signalling and pharmacogenetics. Pharmaceuticals (Basel). 2017;10(4):79. doi: 10.3390/ph10040079.
Review
192
Keks NA, Hope J, Keogh S. Suvorexant: scientifically interesting, utility uncertain. Australas Psychiatry. 2017;25(6):622-624. doi: 10.1177/1039856217734677. Epub 2017 Oct 10.
Opinion
193
Alderson-Day B, Lima CF, Evans S, Krishnan S, Shanmugalingam P, Fernyhough C, Scott SK. Distinct processing of ambiguous speech in people with non-clinical auditory verbal hallucinations. Brain. 2017;140(9):2475-2489. doi: 10.1093/brain/awx206.
No orexin
194
Sheaves B, Freeman D, Isham L, McInerney J, Nickless A, Yu LM, Rek S, Bradley J, Reeve S, Attard C, Espie CA, Foster R, Wirz-Justice A, Chadwick E, Barrera A. Stabilising sleep for patients admitted at acute crisis to a psychiatric hospital (OWLS): an assessor-blind pilot randomised controlled trial. Psychol Med. 2018;48(10):1694-1704. doi: 10.1017/S0033291717003191. Epub 2017 Nov 7.
No orexin
195
Zink AN, Bunney PE, Holm AA, Billington CJ, Kotz CM. Neuromodulation of orexin neurons reduces diet-induced adiposity. Int J Obes (Lond). 2018;42(4):737-745. doi: 10.1038/ijo.2017.276. Epub 2017 Nov 28.
Animal
196
Öz P, Gökalp HK, Göver T, Uzbay T. Dose-dependent and opposite effects of orexin A on prepulse inhibition response in sleep-deprived and non-sleep-deprived rats. Behav Brain Res. 2018;346:73-79. doi: 10.1016/j.bbr.2017.12.002. Epub 2017 Dec 10.
Animal
197
Vergunst F, Jenkinson C, Burns T, Anand P, Gray A, Rugkåsa J, Simon J. Psychometric validation of a multi-dimensional capability instrument for outcome measurement in mental health research (OxCAP-MH). Health Qual Life Outcomes. 2017;15(1):250. doi: 10.1186/s12955-017-0825-3.
No orexin
198
Ross RM, McKay R. Shamanism and the psychosis continuum. Behav Brain Sci. 2018;41:e84. doi: 10.1017/S0140525X17002151.
Opinion
199
Monda V, Salerno M, Sessa F, Bernardini R, Valenzano A, Marsala G, Zammit C, Avola R, Carotenuto M, Messina G, Messina A. Functional changes of orexinergic reaction to psychoactive substances. Mol Neurobiol. 2018;55(8):6362-6368. doi: 10.1007/s12035-017-0865-z. Epub 2018 Jan 6.
Animal
200
Vann Jones S, Banerjee S, Smith AD, Refsum H, Lennox B. Elevated homocysteine and *N*-methyl-D-aspartate-receptor antibodies as a cause of behavioural and cognitive decline in 22q11.2 deletion syndrome. Oxf Med Case Reports. 2017;2017(12):omx076. doi: 10.1093/omcr/omx076.
Case
201
Green AR, Haddad PM, Aronson JK. Marketing medicines: charting the rise of modern therapeutics through a systematic review of adverts in UK medical journals (1950-1980). Br J Clin Pharmacol. 2018;84(8):1668-1685. doi: 10.1111/bcp.13549. Epub 2018 Mar 25.
Review
202
Barton J, Kyle SD, Varese F, Jones SH, Haddock G. Are sleep disturbances causally linked to the presence and severity of psychotic-like, dissociative and hypomanic experiences in non-clinical populations? A systematic review. Neurosci Biobehav Rev. 2018;89:119-131. doi: 10.1016/j.neubiorev.2018.02.008. Epub 2018 Feb 13.
Review
203
Okumura T, Nozu T, Kumei S, Takakusaki K, Ohhira M. Ghrelin acts centrally to induce an antinociceptive action during colonic distension through the orexinergic, dopaminergic and opioid systems in conscious rats. Brain Res. 2018;1686:48-54. doi: 10.1016/j.brainres.2018.02.024. Epub 2018 Feb 21.
Animal
204
Atkin T, Comai S, Gobbi G. Drugs for insomnia beyond benzodiazepines: Pharmacology, clinical applications, and discovery. Pharmacol Rev. 2018;70(2):197-245. doi: 10.1124/pr.117.014381.
Review
205

Kao AC, Spitzer S, Anthony DC, Lennox B, Burnet PWJ. Prebiotic attenuation of olanzapine-induced weight gain in rats: analysis of central and peripheral biomarkers and gut microbiota. Transl Psychiatry. 2018;8(1):66. doi: 10.1038/s41398-018-0116-8.
Animal
206
Wykes T, Joyce E, Velikonja T, Watson A, Aarons G, Birchwood M, Cella M, Dopson S, Fowler D, Greenwood K, Johnson S, McCrone P, Perez J, Pickles A, Reeder C, Rose D, Singh S, Stringer D, Taylor M, Taylor R, Upthegrove R. The CIRCuiTS study (Implementation of cognitive remediation in early intervention services): protocol for a randomised controlled trial. Trials. 2018;19(1):183. doi: 10.1186/s13063-018-2553-3.
Protocol
207
Luo YJ, Li YD, Wang L, Yang SR, Yuan XS, Wang J, Cherasse Y, Lazarus M, Chen JF, Qu WM, Huang ZL. Nucleus accumbens controls wakefulness by a subpopulation of neurons expressing dopamine $D_1$ receptors. Nat Commun. 2018;9(1):1576. doi: 10.1038/s41467-018-03889-3.
Animal
208
Risco S, Mediavilla C. Orexin A in the ventral tegmental area enhances saccharin-induced conditioned flavor preference: The role of D1 receptors in central nucleus of amygdala. Behav Brain Res. 2018;348:192-200. doi: 10.1016/j.bbr.2018.04.010. Epub 2018 Apr 21.
Animal
209
Kotzadimitriou D, Nissen W, Paizs M, Newton K, Harrison PJ, Paulsen O, Lamsa K. Neuregulin 1 type I overexpression is associated with reduced NMDA receptor-mediated synaptic signaling in hippocampal interneurons expressing PV or CCK. eNeuro. 2018;5(2):ENEURO.0418-17.2018. doi: 10.1523/ENEURO.0418-17.2018.
Animal
210
Dujardin S, Pijpers A, Pevernagie D. Prescription drugs used in insomnia. Sleep Med Clin. 2018;13(2):169-182. doi: 10.1016/j.jsmc.2018.03.001.
Review
211
Bolton JL, Ruiz CM, Rismanchi N, Sanchez GA, Castillo E, Huang J, Cross C, Baram TZ, Mahler SV. Early-life adversity facilitates acquisition of cocaine self-administration and induces persistent anhedonia. Neurobiol Stress. 2018;8:57-67. doi: 10.1016/j.ynstr.2018.01.002.
Animal
212
Isham L, Grafahrend H, Nickless A, Pugh K, Pleasants S, Smedley N, Freeman D, Mulligan A. Group-based worry intervention for persecutory delusions: an initial feasibility study. Behav Cogn Psychother. 2018;46(5):619-625. doi: 10.1017/S1352465818000383. Epub 2018 Jun 20.
Unrelated
213
Miskoff JA, Chaudhri M. Off-label sodium oxybate in childhood narcolepsy: A comprehensive report. Cureus. 2018;10(4):e2526. doi: 10.7759/cureus.2526.
Case
214
Juvodden HT, Alnæs D, Lund MJ, Agartz I, Andreassen OA, Dietrichs E, Thorsby PM, Westlye LT, Knudsen S. Widespread white matter changes in post-H1N1 patients with narcolepsy type 1 and first-degree relatives. Sleep. 2018;41(10):zsy145. doi: 10.1093/sleep/zsy145.
No psychosis
215
Bobrovitz N, Heneghan C, Onakpoya I, Fletcher B, Collins D, Tompson A, Lee J, Nunan D, Fisher R, Scott B, O'Sullivan J, Van Hecke O, Nicholson BD, Stevens S, Roberts N, Mahtani KR. Medications that reduce emergency hospital admissions: an overview of systematic reviews and prioritisation of treatments. BMC Med. 2018;16(1):115. doi: 10.1186/s12916-018-1104-9.
Review
216
Palman AD. Сон и его нарушения при хронической обструктивной болезни легких [Sleep and its' disturbanses in chronic obstructive pulmonary disease]. Zh Nevrol Psikhiatr Im S S Korsakova. 2018;118(4. Vyp. 2):113-118. Russian. doi: 10.17116/jnevro201811842113.
Review
217
Liu C, Xue Y, Liu MF, Wang Y, Liu ZR, Diao HL, Chen L. Orexins increase the firing activity of nigral dopaminergic neurons and participate in motor control in rats. J Neurochem. 2018;147(3):380-394. doi: 10.1111/jnc.14568. Epub 2018 Oct 15.
Animal
218
López-Jury L, Meza RC, Brown MTC, Henny P, Canavier CC. Morphological and biophysical determinants of the intracellular and extracellular waveforms in nigral dopaminergic neurons: A computational study. J Neurosci. 2018;38(38):8295-8310. doi: 10.1523/JNEUROSCI.0651-18.2018. Epub 2018 Aug 13.
*In vitro*
219
Ang G, McKillop LE, Purple R, Blanco-Duque C, Peirson SN, Foster RG, Harrison PJ, Sprengel R, Davies KE, Oliver PL, Bannerman DM, Vyazovskiy VV. Absent sleep EEG spindle activity in GluA1 (Gria1) knockout mice: relevance to neuropsychiatric disorders. Transl Psychiatry. 2018;8(1):154. doi: 10.1038/s41398-018-0199-2.
Animal
220
Naganuma F, Bandaru SS, Absi G, Mahoney CE, Scammell TE, Vetrivelan R. Melanin-concentrating hormone neurons contribute to dysregulation of rapid eye movement sleep in narcolepsy. Neurobiol Dis. 2018;120:12-20. doi: 10.1016/j.nbd.2018.08.012. Epub 2018 Aug 24.
Animal
221
Frase L, Nissen C, Riemann D, Spiegelhalder K. Making sleep easier: pharmacological interventions for insomnia. Expert Opin Pharmacother. 2018;19(13):1465-1473. doi: 10.1080/14656566.2018.1511705. Epub 2018 Sep 3.
Review
**222**
**Chen P-Y, Chen C-H, Chang C-K, Kao C-F, Lu M-L, Lin S-K, Huang M-C, Hwang L-L, Mondelli V. Orexin-A levels in relation to the risk of metabolic syndrome in patients with schizophrenia taking antipsychotics. Int J Neuropsychopharmacol. 2019;22(1):28-36. doi: 10.1093/ijnp/pyy075.**
**Included**
223
Prihodova I, Dudova I, Mohaplova M, Hrdlicka M, Nevsimalova S. Childhood narcolepsy and autism spectrum disorders: four case reports. Sleep Med. 2018;51:167-170. doi: 10.1016/j.sleep.2018.07.017. Epub 2018 Aug 9.
Case

224
Heiss JE, Yamanaka A, Kilduff TS. Parallel arousal pathways in the lateral hypothalamus. eNeuro. 2018;5(4):ENEURO.0228-18.2018. doi: 10.1523/ENEURO.0228-18.2018.
Animal
225
Lu G-L, Lee MT, Chiou L-C. Orexin-mediated restoration of hippocampal synaptic potentiation in mice with established cocaine-conditioned place preference. Addict Biol. 2019;24(6):1153-1166. doi: 10.1111/adb.12672. Epub 2018 Oct 1.
Animal
226
Okumura T, Nozu T, Kumei S, Ohhira M. Central oxytocin signaling mediates the central orexin-induced visceral antinociception through the opioid system in conscious rats. Physiol Behav. 2019;198:96-101. doi: 10.1016/j.physbeh.2018.10.007. Epub 2018 Oct 18.
Animal
227
Zhou W, Cheung K, Kyu S, Wang L, Guan Z, Kurien PA, Bickler PE, Jan LY. Activation of orexin system facilitates anesthesia emergence and pain control. Proc Natl Acad Sci U S A. 2018;115(45):E10740-E10747. doi: 10.1073/pnas.1808622115. Epub 2018 Oct 22.
Animal
228
Gerry CJ. The economic case for deinstitutionalisation in post-communist Europe. Lancet Psychiatry. 2018;5(12):950-952. doi: 10.1016/S2215-0366(18)30435-8. Epub 2018 Nov 8.
Unrelated
229
Linehan V, Rowe TM, Hirasawa M. Dopamine modulates excitatory transmission to orexin neurons in a receptor subtype-specific manner. Am J Physiol Regul Integr Comp Physiol. 2019;316(1):R68-R75. doi: 10.1152/ajpregu.00150.2018. Epub 2018 Nov 21.
Animal
230
Singh R, Bansal Y, Medhi B, Kuhad A. Antipsychotics-induced metabolic alterations: Recounting the mechanistic insights, therapeutic targets and pharmacological alternatives. Eur J Pharmacol. 2019;844:231-240. doi: 10.1016/j.ejphar.2018.12.003. Epub 2018 Dec 7.
Review
231
McElroy SL, Guerdjikova AI, Mori N, Romo-Nava F. Progress in developing pharmacologic agents to treat bulimia nervosa. CNS Drugs. 2019;33(1):31-46. doi: 10.1007/s40263-018-0594-5.
Review
232
Magdaleno-Madrigal VM, Morales-Mulia S, Nicolini H, Genis-Mendoza A, Cázares-Martínez Claudia E, Pérez-Luna José M, Morales-Mulia M. Orexin-A promotes EEG changes but fails to induce anxiety in rats. Behav Brain Res. 2019;361:26-31. doi: 10.1016/j.bbr.2018.12.037. Epub 2018 Dec 21.
Animal
233
Drugs for chronic insomnia. Med Lett Drugs Ther. 2018;60(1562):201-205.
Opinion
234
Kao AC, Chan KW, Anthony DC, Lennox BR, Burnet PW. Prebiotic reduction of brain histone deacetylase (HDAC) activity and olanzapine-mediated weight gain in rats, are acetate independent. Neuropharmacology. 2019;150:184-191. doi: 10.1016/j.neuropharm.2019.02.014. Epub 2019 Feb 11.
Animal
235
Akinnusi M, El Solh AA. Drug treatment strategies for insomnia in patients with post-traumatic stress disorder. Expert Opin Pharmacother. 2019;20(6):691-699. doi: 10.1080/14656566.2019.1574745. Epub 2019 Feb 22.
Review
236
Juvodden HT, Alnæs D, Lund MJ, Dietrichs E, Thorsby PM, Westlye LT, Knudsen S. Hypocretin-deficient narcolepsy patients have abnormal brain activation during humor processing. Sleep. 2019;42(7):zsz082. doi: 10.1093/sleep/zsz082.
No psychosis
237
Naghavi FS, Namvar P, Sadeghzadeh F, Haghparast A. The involvement of intra-hippocampal dopamine receptors in the conditioned place preference induced by orexin administration into the rat ventral tegmental area. Iran J Pharm Res. 2019;18(1):328-338.
Animal
238
Misiak B, Bartoli F, Stramecki F, Samochowiec J, Lis M, Kasznia J, Jarosz K, Stańczykiewicz B. Appetite regulating hormones in first-episode psychosis: A systematic review and meta-analysis. Neurosci Biobehav Rev. 2019;102:362-370. doi: 10.1016/j.neubiorev.2019.05.018. Epub 2019 May 20.
Review
239
Soneson E, Russo D, Knight C, Lafortune L, Heslin M, Stochl J, Georgiadis A, Galante J, Duschinsky R, Grey N, Gonzalez-Blanco L, Couche J, Griffiths M, Murray H, Reeve N, Hodgekins J, French P, Fowler D, Byford S, Dixon-Woods M, Jones PB, Perez J. Psychological interventions for people with psychotic experiences: protocol for a systematic review and meta-analysis. Syst Rev. 2019;8(1):124. doi: 10.1186/s13643-019-1041-5.
Protocol
240
Lennox B, Yeeles K, Jones PB, Zandi M, Joyce E, Yu LM, Tomei G, Pollard R, Vincent SA, Shimazaki M, Cairns I, Dowling F, Kabir T, Barnes TRE, Lingford Hughes A, Hosseini AA, Harrower T, Buckley C, Coles A. Intravenous immunoglobulin and rituximab versus placebo treatment of antibody-associated psychosis: study protocol of a randomised phase IIa double-blinded placebo- controlled trial (SINAPPS2). Trials. 2019;20(1):331. doi: 10.1186/s13063-019-3336-1.
Protocol
241
Rolls ET, Cheng W, Gilson M, Gong W, Deco G, Lo CZ, Yang AC, Tsai SJ, Liu ME, Lin CP, Feng J. Beyond the disconnectivity hypothesis of schizophrenia. Cereb Cortex. 2020;30(3):1213-1233. doi: 10.1093/cercor/bhz161.
No orexin
242
Ni P, Tian Y, Gu X, Yang L, Wei J, Wang Y, Zhao L, Zhang Y, Zhang C, Li L, Tang X, Ma X, Hu X, Li T. Plasma neuropeptides as circulating biomarkers of multifactorial schizophrenia. Compr Psychiatry. 2019;94:152114. doi: 10.1016/j.comppsych.2019.152114. Epub 2019 Aug 5.

Animal
243
Vanda D, Zajdel P, Soural M. Imidazopyridine-based selective and multifunctional ligands of biological targets associated with psychiatric and neurodegenerative diseases. Eur J Med Chem. 2019;181:111569. doi: 10.1016/j.ejmech.2019.111569. Epub 2019 Jul 31.
Review
244
Krystal AD, Prather AA, Ashbrook LH. The assessment and management of insomnia: an update. World Psychiatry. 2019;18(3):337-352. doi: 10.1002/wps.20674.
Review
**245**
**Tsuchimine S, Hattori K, Ota M, Hidese S, Teraishi T, Sasayama D, Hori H, Noda T, Yoshida S, Yoshida F, Kunugi H. Reduced plasma orexin-A levels in patients with bipolar disorder. Neuropsychiatr Dis Treat. 2019;15:2221-2230. doi: 10.2147/NDT.S209023.**
**Included**
246
Bruni O, Angriman M, Melegari MG, Ferri R. Pharmacotherapeutic management of sleep disorders in children with neurodevelopmental disorders. Expert Opin Pharmacother. 2019;20(18):2257-2271. doi: 10.1080/14656566.2019.1674283. Epub 2019 Oct 22.
Review
247
Stanojlovic M, Pallais JP, Lee MK, Kotz CM. Pharmacological and chemogenetic orexin/hypocretin intervention ameliorates Hipp-dependent memory impairment in the A53T mice model of Parkinson's disease. Mol Brain. 2019;12(1):87. doi: 10.1186/s13041-019-0514-8.
Animal
248
Hanazawa T, Kamijo Y. Effect of suvorexant on nocturnal delirium in elderly patients with Alzheimer's disease: A case-series study. Clin Psychopharmacol Neurosci. 2019;17(4):547-550. doi: 10.9758/cpn.2019.17.4.547.
Case
249
Chamera K, Trojan E, Szuster-Głuszczak M, Basta-Kaim A. The potential role of dysfunctions in neuron-microglia communication in the pathogenesis of brain disorders. Curr Neuropharmacol. 2020;18(5):408-430. doi: 10.2174/1570159X17666191113101629.
Review
250
Sarker G, Litwan K, Kastli R, Peleg-Raibstein D. Maternal overnutrition during critical developmental periods leads to different health adversities in the offspring: relevance of obesity, addiction and schizophrenia. Sci Rep. 2019;9(1):17322. doi: 10.1038/s41598-019-53652-x.
Animal
251
Chen XY, Xue Y, Chen H, Chen L. The globus pallidus as a target for neuropeptides and endocannabinoids participating in central activities. Peptides. 2020;124:170210. doi: 10.1016/j.peptides.2019.170210. Epub 2019 Nov 26.
Review
252
Bada Juarez JF, Muñoz-García JC, Inácio Dos Reis R, Henry A, McMillan D, Kriek M, Wood M, Vandenplas C, Sands Z, Castro L, Taylor R, Watts A. Detergent- free extraction of a functional low-expressing GPCR from a human cell line. Biochim Biophys Acta Biomembr. 2020;1862(3):183152. doi: 10.1016/j.bbamem.2019.183152. Epub 2019 Dec 13.
*In vitro*
253
Murai H, Suzuki H, Tanji H, Kimura T, Iba Y. A simple method using anesthetics to test effects of sleep-inducing substances in mice. J Pharmacol Sci. 2020;142(2):79-82. doi: 10.1016/j.jphs.2019.12.003. Epub 2019 Dec 6.
Animal
254
Feketeova E, Tormasiova M, Klobučníková K, Durdik P, Jarcuskova D, Benca M, Vitkova M. Narcolepsy in Slovakia - Epidemiology, clinical and polysomnographic features, comorbid diagnoses: a case-control study. Sleep Med. 2020;67:15-22. doi: 10.1016/j.sleep.2019.10.012. Epub 2019 Nov 11.
No psychosis
255
Rezaee L, Alizadeh AM, Haghparast A. Role of hippocampal dopamine receptors in the antinociceptive responses induced by chemical stimulation of the lateral hypothalamus in animal model of acute pain. Brain Res. 2020;1734:146759. doi: 10.1016/j.brainres.2020.146759. Epub 2020 Mar 2.
Animal
256
Hou Y, Liu Y, Liu C, Yan Z, Ma Q, Chen J, Zhang M, Yan Q, Li X, Chen J. Xiaoyaosan regulates depression-related behaviors with physical symptoms by modulating Orexin A/OxR1 in the hypothalamus. Anat Rec (Hoboken). 2020;303(8):2144-2153. doi: 10.1002/ar.24386. Epub 2020 Mar 16.
Animal
257
Su J, Li Z, Yamashita A, Kusumoto-Yoshida I, Isomichi T, Hao L, Kuwaki T. Involvement of the nucleus accumbens in chocolate-induced cataplexy. Sci Rep. 2020;10(1):4958. doi: 10.1038/s41598-020-61823-4.
Animal
**258**
**Liu Z, Zhang Y, Zhao T, Wang J, Xia L, Zhong Y, Yang Y, Ning X, Zhang Y, Ren Z, Liu H. A higher body mass index in Chinese inpatients with chronic schizophrenia is associated with elevated plasma orexin-A levels and fewer negative symptoms. Nord J Psychiatry. 2020;74(7):525-532. doi: 10.1080/08039488.2020.1755995. Epub 2020 May 4.**
**Included**
259
Dujardin S, Pijpers A, Pevernagie D. Prescription drugs used in insomnia. Sleep Med Clin. 2020;15(2):133-145. doi: 10.1016/j.jsmc.2020.02.002.
Review
260
BaHammam AS, Alnakshabandi K, Pandi-Perumal SR. Neuropsychiatric correlates of narcolepsy. Curr Psychiatry Rep. 2020;22(8):36. doi: 10.1007/s11920-020-01159-y.
Review
261
Lis M, Stańczykiewicz B, Liśkiewicz P, Misiak B. Impaired hormonal regulation of appetite in schizophrenia: A narrative review dissecting intrinsic mechanisms and the effects of antipsychotics. Psychoneuroendocrinology. 2020;119:104744. doi: 10.1016/j.psyneuen.2020.104744. Epub 2020 Jun 5.

Review
262
Matini T, Haghparast A, Rezaee L, Salehi S, Tehranchi A, Haghparast A. Role of dopaminergic receptors within the ventral tegmental area in antinociception induced by chemical stimulation of the lateral hypothalamus in an animal model of orofacial pain. J Pain Res. 2020;13:1449-1460. doi: 10.2147/JPR.S255250.
Animal
263
Purple RJ, Cosgrave J, Vyazovskiy V, Foster RG, Porcheret K, Wulff K. Sleep-related memory consolidation in the psychosis spectrum phenotype. Neurobiol Learn Mem. 2020;174:107273. doi: 10.1016/j.nlm.2020.107273. Epub 2020 Jul 10.
No orexin
264
Chan SY, Probert F, Radford-Smith DE, Hebert JC, Claridge TDW, Anthony DC, Burnet PWJ. Post-inflammatory behavioural despair in male mice is associated with reduced cortical glutamate-glutamine ratios, and circulating lipid and energy metabolites. Sci Rep. 2020;10(1):16857. doi: 10.1038/s41598-020-74008-w.
Animal
265
Al-Kuraishy HM, Abdulhadi MH, Hussien NR, Al-Niemi MS, Rasheed HA, Al- Gareeb AI. Involvement of orexinergic system in psychiatric and neurodegenerative disorders: A scoping review. Brain Circ. 2020;6(2):70-80. doi: 10.4103/bc.bc_42_19.
Review
266
Molnár Z, Luhmann HJ, Kanold PO. Transient cortical circuits match spontaneous and sensory-driven activity during development. Science. 2020;370(6514):eabb2153. doi: 10.1126/science.abb2153.
Review
267
Durairaja A, Fendt M. Orexin deficiency modulates cognitive flexibility in a sex-dependent manner. Genes Brain Behav. 2021;20(3):e12707. doi: 10.1111/gbb.12707. Epub 2020 Nov 3.
Animal
268
Khaleghzadeh-Ahangar H, Rashvand M, Haghparast A. Role of D1- and D2-like dopamine receptors within the dentate gyrus in antinociception induced by chemical stimulation of the lateral hypothalamus in an animal model of acute pain. Physiol Behav. 2021;229:113214. doi: 10.1016/j.physbeh.2020.113214. Epub 2020 Oct 20.
Animal
269
Černis E, Evans R, Ehlers A, Freeman D. Dissociation in relation to other mental health conditions: An exploration using network analysis. J Psychiatr Res. 2021;136:460-467. doi: 10.1016/j.jpsychires.2020.08.023. Epub 2020 Aug 20.
No orexin
270
Kenaan K, Zafar M, Bond R, Gracious BL. Perampanel-induced cataplexy in a young male with generalized epilepsy. HCA Healthc J Med. 2020;1(5):283-288. doi: 10.36518/2689-0216.1040.
Case
271
Chén OY, Cao H, Phan H, Nagels G, Reinen JM, Gou J, Qian T, Di J, Prince J, Cannon TD, de Vos M. Identifying neural signatures mediating behavioral symptoms and psychosis onset: High-dimensional whole brain functional mediation analysis. Neuroimage. 2021;226:117508. doi: 10.1016/j.neuroimage.2020.117508. Epub 2020 Nov 4.
No orexin
272
Ashraf GM, Alghamdi BS, Alshehri FS, Alam MZ, Tayeb HO, Tarazi FI. Standardizing the effective correlated dosage of olanzapine and empagliflozin in female Wistar rats. Curr Gene Ther. 2021;21(1):53-59. doi: 10.2174/1566523220999201111195047.
Animal
273
Nigam M, Leu-Semenescu S, Arnulf I. Successful treatment of drug-resistant cataplexy with the anticholinergic drug tropatepine. J Clin Sleep Med. 2021;17(4):849-851. doi: 10.5664/jcsm.9030.
Case
274
Geraghty Z, Barnard C, Uluocak P, Gruneberg U. The association of Plk1 with the astrin-kinastrin complex promotes formation and maintenance of a metaphase plate. J Cell Sci. 2021;134(1):jcs251025. doi: 10.1242/jcs.251025.
Unrelated
275
Martins D, Rademacher L, Gabay AS, Taylor R, Richey JA, Smith DV, Goerlich KS, Nawijn L, Cremers HR, Wilson R, Bhattacharyya S, Paloyelis Y. Mapping social reward and punishment processing in the human brain: A voxel-based meta-analysis of neuroimaging findings using the social incentive delay task. Neurosci Biobehav Rev. 2021;122:1-17. doi: 10.1016/j.neubiorev.2020.12.034. Epub 2021 Jan 6.
Review
276
Elam HB, Perez SM, Donegan JJ, Lodge DJ. Orexin receptor antagonists reverse aberrant dopamine neuron activity and related behaviors in a rodent model of stress-induced psychosis. Transl Psychiatry. 2021;11(1):114. doi: 10.1038/s41398-021-01235-8.
Animal
277
Crawford K, Oliver PL, Agnew T, Hunn BHM, Ahel I. Behavioural characterisation of *Macrod1* and *Macrod2* knockout mice. Cells. 2021;10(2):368. doi: 10.3390/cells10020368.
Animal
278
Perez SM, Lodge DJ. Orexin modulation of VTA dopamine neuron activity: Relevance to schizophrenia. Int J Neuropsychopharmacol. 2021;24(4):344-353. doi: 10.1093/ijnp/pyaa080.
Animal
279
Veeraraghavan V. Obesogenic behavior and binge eating disorder in an elderly female with schizophrenia. J Obes Metab Syndr. 2021;30(2):184-187. doi: 10.7570/jomes20096.
Case

280
Ashraf GM, Alghamdi BS, Alshehri FS, Alam MZ, Tayeb HO, Tarazi FI. Empagliflozin effectively attenuates olanzapine-induced body weight gain in female Wistar rats. Front Pharmacol. 2021;12:578716. doi: 10.3389/fphar.2021.578716.
Animal
**281**
**Lu J, Huang ML, Li JH, Jin KY, Li HM, Mou TT, Fronczek R, Duan JF, Xu WJ, Swaab D, Bao AM. Changes of hypocretin (Orexin) system in schizophrenia: From plasma to brain. Schizophr Bull. 2021;47(5):1310-1319. doi: 10.1093/schbul/sbab042.**
**Included**
282
Saatci D, van Nieuwenhuizen A, Handunnetthi L. Maternal infection in gestation increases the risk of non-affective psychosis in offspring: a meta-analysis. J Psychiatr Res. 2021;139:125-131. doi: 10.1016/j.jpsychires.2021.05.039. Epub 2021 May 23.
No orexin
283
Perry BAL, Lomi E, Mitchell AS. Thalamocortical interactions in cognition and disease: The mediodorsal and anterior thalamic nuclei. Neurosci Biobehav Rev. 2021;130:162-177. doi: 10.1016/j.neubiorev.2021.05.032. Epub 2021 Jun 30.
Review
284
Nasrollahi S, Karimi S, Hamidi G, Naderitehrani M, Abed A. Blockade of the orexin 1 receptors in the nucleus accumbens' shell reversed the reduction effect of olanzapine on motivation for positive reinforcers. Neurosci Lett. 2021;762:136137. doi: 10.1016/j.neulet.2021.136137. Epub 2021 Jul 24.
Animal
285
Molina JD, Avila S, Rubio G, López-Muñoz F. Metabolomic connections between schizophrenia, antipsychotic drugs and metabolic syndrome: A variety of players. Curr Pharm Des. 2021;27(39):4049-4061. doi: 10.2174/1381612827666210804110139.
Review
286
Grünwald LM, Duddy C, Byng R, Crellin N, Moncrieff J. The role of trust and hope in antipsychotic medication reviews between GPs and service users a realist review. BMC Psychiatry. 2021;21(1):390. doi: 10.1186/s12888-021-03355-3.
No orexin
287
Pisani S, Murphy J, Conway J, Millgate E, Catmur C, Bird G. The relationship between alexithymia and theory of mind: A systematic review. Neurosci Biobehav Rev. 2021;131:497-524. doi: 10.1016/j.neubiorev.2021.09.036. Epub 2021 Sep 29.
Review
288
Porwal A, Yadav YC, Pathak K, Yadav R. An update on assessment, therapeutic management, and patents on insomnia. Biomed Res Int. 2021;2021:6068952. doi: 10.1155/2021/6068952.
Review
289
Černis E, Molodynski A, Ehlers A, Freeman D. Dissociation in patients with non-affective psychosis: Prevalence, symptom associations, and maintenance factors. Schizophr Res. 2022;239:11-18. doi: 10.1016/j.schres.2021.11.008. Epub 2021 Nov 17.
No orexin
290
Handunnetthi L, Saatci D, Hamley JC, Knight JC. Maternal immune activation downregulates schizophrenia genes in the foetal mouse brain. Brain Commun. 2021;3(4):fcab275. doi: 10.1093/braincomms/fcab275.
Animal
291
Iakovleva OV, Levin OS (Яковлева О.В., Левин О.С.). Речевые и поведенческие контаминации как неэпилептические автоматизмы при болезни Паркинсона [Speech and behavioral contaminations as non-epileptic automatic behavior in Parkinson's disease]. Zh Nevrol Psikhiatr Im S S Korsakova. 2021;121(10. Vyp. 2):58-63 (Журнал неврологии и психиатрии им. С.С. Корсакова 2021;т. 121(№10, вып. 2)с. 58-63). Russian. doi: 10.17116/jnevro202112110258.
Review
292
Terada T, Hirayama T, Sadahiro R, Wada S, Nakahara R, Matsuoka H. Pilot study of lemborexant for insomnia in cancer patients with delirium. J Palliat Med. 2022;25(5):797-801. doi: 10.1089/jpm.2021.0509. Epub 2022 Jan 28.
Unfocused
293
Demidova A, Kahl E, Fendt M. Orexin deficiency affects sensorimotor gating and its amphetamine-induced impairment. Prog Neuropsychopharmacol Biol Psychiatry. 2022;116:110517. doi: 10.1016/j.pnpbp.2022.110517. Epub 2022 Jan 29.
Animal
294
Chyr J, Gong H, Zhou X. DOTA: Deep Learning Optimal Transport Approach to advance drug repositioning for Alzheimer's disease. Biomolecules. 2022;12(2):196. doi: 10.3390/biom12020196.
Unfocused
295
Cope TE, Hughes LE, Phillips HN, Adams NE, Jafarian A, Nesbitt D, Assem M, Woolgar A, Duncan J, Rowe JB. Causal evidence for the multiple demand network in change detection: Auditory mismatch magnetoencephalography across focal neurodegenerative diseases. J Neurosci. 2022;42(15):3197-3215. doi: 10.1523/JNEUROSCI.1622-21.2022. Epub 2022 Mar 8.
No orexin
296
Maidment ID, Wong G, Duddy C, Upthegrove R, Oduola S, Robotham D, Higgs S, Ahern A, Birdi G. REalist Synthesis Of non-pharmacologicaL interVEntions for antipsychotic-induced weight gain (RESOLVE) in people living with severe mental illness (SMI). Syst Rev. 2022;11(1):42. doi: 10.1186/s13643-022-01912-9.
Protocol
297
Guma E, Bordeleau M, González Ibáñez F, Picard K, Snook E, Desrosiers-Grégoire G, Spring S, Lerch JP, Nieman BJ, Devenyi GA, Tremblay ME, Chakravarty MM. Differential effects of early or late exposure to prenatal maternal immune activation on mouse embryonic neurodevelopment. Proc Natl Acad Sci U S A. 2022;119(12):e2114545119. doi: 10.1073/pnas.2114545119. Epub 2022 Mar 14.
Animal

298
Phiri P, Engelthaler T, Carr H, Delanerolle G, Holmes C, Rathod S. Associated mortality risk of atypical antipsychotic medication in individuals with dementia. World J Psychiatry. 2022;12(2):298-307. doi: 10.5498/wjp.v12.i2.298.
No orexin
**299**
**Chen PY, Chang CK, Chen CH, Fang SC, Mondelli V, Chiu CC, Lu ML, Hwang LL, Huang MC. Orexin-a elevation in antipsychotic-treated compared to drug-free patients with schizophrenia: A medication effect independent of metabolic syndrome. J Formos Med Assoc. 2022;121(11):2172-2181. doi: 10.1016/j.jfma.2022.03.008. Epub 2022 Apr 6.**
**Included**
300
Lammas F, Phillips A, Dopson S, Joyce E, Csipke E, Wykes T. The organisational climate of NHS Early Intervention Services (EIS) for psychosis: a qualitative analysis. BMC Health Serv Res. 2022;22(1):509. doi: 10.1186/s12913-022-07790-0.
No orexin
301
Radley J, Barlow J, Johns LC. Sociodemographic characteristics associated with parenthood amongst patients with a psychotic diagnosis: a cross-sectional study using patient clinical records. Soc Psychiatry Psychiatr Epidemiol. 2022;57(9):1897-1906. doi: 10.1007/s00127-022-02279-x. Epub 2022 Apr 21.
No orexin
302
Palagini L, Hertenstein E, Riemann D, Nissen C. Sleep, insomnia and mental health. J Sleep Res. 2022;31(4):e13628. doi: 10.1111/jsr.13628. Epub 2022 May 4.
Review
303
Brown RE, Spratt TJ, Kaplan GB. Translational approaches to influence sleep and arousal. Brain Res Bull. 2022;185:140-161. doi: 10.1016/j.brainresbull.2022.05.002. Epub 2022 May 10.
Review
**304**
**Ren J, Chen Y, Fang X, Wang D, Wang Y, Yu L, Wu Z, Liu R, Zhang C. Correlation of Orexin-A and brain-derived neurotrophic factor levels in metabolic syndrome and cognitive impairment in schizophrenia treated with clozapine. Neurosci Lett. 2022;782:136695. doi: 10.1016/j.neulet.2022.136695. Epub 2022 May 24.**
**Included**
305
Delanerolle G, Zeng Y, Shi JQ, Yeng X, Goodison W, Shetty A, Shetty S, Haque N, Elliot K, Ranaweera S, Ramakrishnan R, Raymont V, Rathod S, Phiri P. Mental health impact of the Middle East respiratory syndrome, SARS, and COVID-19: A comparative systematic review and meta-analysis. World J Psychiatry. 2022;12(5):739-765. doi: 10.5498/wjp.v12.i5.739.
Review
306
Li S, Zhang R, Hu S, Lai J. Plasma orexin-A levels in patients with schizophrenia: A systematic review and meta-analysis. Front Psychiatry. 2022;13:879414. doi: 10.3389/fpsyt.2022.879414.
Review
307
Vasiliu O. Investigational drugs for the treatment of depression (Part 1): Monoaminergic, orexinergic, GABA-ergic, and anti-inflammatory agents. Front Pharmacol. 2022;13:884143. doi: 10.3389/fphar.2022.884143.
Review
308
Daridorexant (Quviviq) for insomnia. Med Lett Drugs Ther. 2022;64(1654):107-110.
Opinion
309
Gool JK, Fronczek R, Bosma P, van der Meer JN, van der Werf YD, Lammers GJ. Enhanced visual cortex activation in people with Narcolepsy Type 1 during active sleep resistance: An fMRI-EEG study. Front Neurosci. 2022;16:904820. doi: 10.3389/fnins.2022.904820.
No psychosis
310
Musa A, Khan S, Mujahid M, El-Gaby M. The shallow cognitive map hypothesis: A hippocampal framework for thought disorder in schizophrenia. Schizophrenia (Heidelb). 2022;8(1):34. doi: 10.1038/s41537-022-00247-7.
Review
311
Saatci D, Johnson T, Smee M, van Nieuwenhuizen A, Handunnetthi L. The role of latitude and infections in the month-of-birth effect linked to schizophrenia. Brain Behav Immun Health. 2022;24:100486. doi: 10.1016/j.bbih.2022.100486.
Review
312
Lin CC, Huang TL. Orexin/hypocretin and major psychiatric disorders. Adv Clin Chem. 2022;109:185-212. doi: 10.1016/bs.acc.2022.03.006. Epub 2022 Apr 18.
Review
313
Osipov EM, Munawar AH, Beelen S, Fearon D, Douangamath A, Wild C, Weeks SD, Van Aerschot A, von Delft F, Strelkov SV. Discovery of novel druggable pockets on polyomavirus VP1 through crystallographic fragment-based screening to develop capsid assembly inhibitors. RSC Chem Biol. 2022;3(8):1013-1027. doi: 10.1039/d2cb00052k.
*In vitro*
314
Zhu Z, Gu Y, Zeng C, Yang M, Yu H, Chen H, Zhang B, Cai H. Olanzapine-induced lipid disturbances: A potential mechanism through the gut microbiota-brain axis. Front Pharmacol. 2022;13:897926. doi: 10.3389/fphar.2022.897926.
Animal
315
Hong J, Vernon D, Kunovac J, Stahl S. Emerging drugs for the treatment of major depressive disorder. Expert Opin Emerg Drugs. 2022;27(3):263-275. doi: 10.1080/14728214.2022.2117297. Epub 2022 Sep 20.
Review
316

Mikutta CA, Pervilhac C, Znoj H, Federspiel A, Müller TJ. The impact of foehn wind on mental distress among patients in a Swiss psychiatric hospital. Int J Environ Res Public Health. 2022;19(17):10831. doi: 10.3390/ijerph191710831.
No orexin
317
Hintze JP, Edinger JD. Hypnotic discontinuation in chronic insomnia. Sleep Med Clin. 2022;17(3):523-530. doi: 10.1016/j.jsmc.2022.06.014.
Review
318
Kalra S, Bathla M, Verma S. Lemborexant: An adjuvant in difficult-to-control diabetes? J Pak Med Assoc. 2022;72(9):1874-1875. doi: 10.47391/JPMA.22-93.
Opinion
319
Karimi S, Zibaii MI, Hamidi GA, Haghparast A. Differential effects of the lateral hypothalamus lesion as an origin of orexin and blockade of orexin-1 receptor in the orbitofrontal cortex and anterior cingulate cortex on their neuronal activity. Basic Clin Neurosci. 2022;13(3):407-420. doi: 10.32598/bcn.2022.2029.1. Epub 2022 May 1.
Animal
320
Panayi MC, Boerner T, Jahans-Price T, Huber A, Sprengel R, Gilmour G, Sanderson DJ, Harrison PJ, Walton ME, Bannerman DM. Glutamatergic dysfunction leads to a hyper-dopaminergic phenotype through deficits in short-term habituation: a mechanism for aberrant salience. Mol Psychiatry. 2023;28(2):579-587. doi: 10.1038/s41380-022-01861-8. Epub 2022 Dec 2.
Animal
321
Tang PY, Tee SF, Su KP. Editorial: The link between nutrition and schizophrenia. Front Psychiatry. 2022;13:1074120. doi: 10.3389/fpsyt.2022.1074120.
Opinion
322
Bergamini G, Coloma P, Massinet H, Steiner MA. What evidence is there for implicating the brain orexin system in neuropsychiatric symptoms in dementia? Front Psychiatry. 2022;13:1052233. doi: 10.3389/fpsyt.2022.1052233.
Review
323
Drugs for chronic insomnia. Med Lett Drugs Ther. 2023;65(1667):1-6. doi: 10.58347/tml.2023.1667a.
Opinion
324
Feketeová E, Dragašek J, Klobučníková K, Ďurdík P, Čarnakovič S, Slavkovská M, Chylová M. Psychotic episode and schizophrenia in Slovakian Narcolepsy Database. Brain Sci. 2022;13(1):43. doi: 10.3390/brainsci13010043.
No psychosis
325
Malik JA, Yaseen Z, Thotapalli L, Ahmed S, Shaikh MF, Anwar S. Understanding translational research in schizophrenia: A novel insight into animal models. Mol Biol Rep. 2023;50(4):3767-3785. doi: 10.1007/s11033-023-08241-7. Epub 2023 Jan 24. Erratum in: Mol Biol Rep. 2023;50(5):4755. doi: 10.1007/s11033-023-08352-1.
Review
326
Han AH, Burroughs CR, Falgoust EP, Hasoon J, Hunt G, Kakazu J, Lee T, Kaye AM, Kaye AD, Ganti L. Suvorexant, a novel dual orexin receptor antagonist, for the management of insomnia. Health Psychol Res. 2023;10(5):67898. doi: 10.52965/001c.67898.
Review
327
Maness EB, Blumenthal SA, Burk JA. Dual orexin/hypocretin receptor antagonism attenuates attentional impairments in an NMDA receptor hypofunction model of schizophrenia. bioRxiv [Preprint]. 2023 Feb 5:2023.02.05.527043. doi: 10.1101/2023.02.05.527043. Update in: Behav Brain Res. 2023;450:114497. doi: 10.1016/j.bbr.2023.114497.
Dupl 327PM
328
Lee C, Waite F, Piernas C, Aveyard P. Development and initial evaluation of a behavioural intervention to support weight management for people with serious mental illness: an uncontrolled feasibility and acceptability study. BMC Psychiatry. 2023;23(1):130. doi: 10.1186/s12888-023-04517-1.
No orexin
329
Okuda S, Qureshi ZP, Yanagida Y, Ito C, Homma Y, Tokita S. Factors associated with prescriptions for an orexin receptor antagonist among Japanese patients with insomnia: Analysis of a nationwide Japanese claims database. Drugs Real World Outcomes. 2023;10(2):271-281. doi: 10.1007/s40801-023-00356-4. Epub 2023 Mar 3.
No psychosis
330
Mana L, Vila-Vidal M, Köckeritz C, Aquino K, Fornito A, Kringelbach ML, Deco G. Using in silico perturbational approach to identify critical areas in schizophrenia. Cereb Cortex. 2023;33(12):7642-7658. doi: 10.1093/cercor/bhad067.
No orexin
331
Wang MO, Ma J, Li SX, Zhang L. 王美鸥，马 菁，李思迅，张 岚. 1型发作性睡病共病精神分裂症1例报告 [Narcolepsy Type 1 With Comorbid Schizophrenia: A Case Report]. Sichuan Da Xue Xue Bao Yi Xue Ban 四川大学学报(医学版). 2023;54(2):444-446. Chinese. doi: 10.12182/20230360104.
Case
332
Sariaslan A, Fanshawe T, Pitkänen J, Cipriani A, Martikainen P, Fazel S. Predicting suicide risk in 137,112 people with severe mental illness in Finland: external validation of the Oxford Mental Illness and Suicide tool (OxMIS). Transl Psychiatry. 2023;13(1):126. doi: 10.1038/s41398-023-02422-5.
No orexin
333
Lin Y, Roy K, Ioka S, Otani R, Amezawa M, Ishikawa Y, Cherasse Y, Kaushik MK, Klewe-Nebenius D, Zhou L, Yanagisawa M, Oishi Y, Saitoh T, Lazarus M. Positive allosteric adenosine $A_{2A}$ receptor modulation suppresses insomnia associated with mania- and schizophrenia-like behaviors in mice. Front Pharmacol. 2023;14:1138666. doi: 10.3389/fphar.2023.1138666.
Animal
334

Leonardsen EH, Vidal-Piñeiro D, Roe JM, Frei O, Shadrin AA, Iakunchykova O, de Lange AG, Kaufmann T, Taschler B, Smith SM, Andreassen OA, Wolfers T, Westlye LT, Wang Y. Genetic architecture of brain age and its causal relations with brain and mental disorders. Mol Psychiatry. 2023;28(7):3111-3120. doi: 10.1038/s41380-023-02087-y. Epub 2023 May 10.
No orexin
335
Williams SG, Rodriguez-Cué D. Use of daridorexant among patients with chronic insomnia: A retrospective observational analysis. J Clin Med. 2023;12(9):3240. doi: 10.3390/jcm12093240.
No psychosis
336
Fagan HA, Baldwin DS. Pharmacological treatment of generalised anxiety disorder: Current practice and future directions. Expert Rev Neurother. 2023;23(6):535-548. doi: 10.1080/14737175.2023.2211767. Epub 2023 May 15.
Review
337
Maness EB, Blumenthal SA, Burk JA. Dual orexin/hypocretin receptor antagonism attenuates NMDA receptor hypofunction-induced attentional impairments in a rat model of schizophrenia. Behav Brain Res. 2023;450:114497. doi: 10.1016/j.bbr.2023.114497. Epub 2023 May 16.
Animal
338
Horikoshi S, Miura I, Suzuki Y, Kobayashi Y, Hirata Y, Goto M, Ichinose M, Yamamoto S, Kanno-Nozaki K, Watanabe K, Yabe H. Switching to lemborexant for the management of insomnia in mental disorders: the SLIM study. J Clin Sleep Med. 2023;19(10):1753-1758. doi: 10.5664/jcsm.10668.
Unfocused
339
Abdelzaher WY, De Waard M, Abdelmonaem AA, Ali DM, El-Tahawy NFG, Rifaai RA, Mohamed HA, Shaheen K, Zeen El-Din MA, Welson NN, Tawfeek SE, Batiha GE, Abdel-Aziz AM. Empagliflozin protects against haloperidol experimentally-induced ovarian toxicity. Pharmaceuticals (Basel). 2023;16(2):168. doi: 10.3390/ph16020168.
Animal
**340**
**Yu H, Ni P, Zhao L, Tian Y, Li M, Li X, Wei W, Wei J, Deng W, Du X, Wang Q, Guo W, Ma X, Coid J, Li T. Decreased plasma neuropeptides in first-episode schizophrenia, bipolar disorder, major depressive disorder: associations with clinical symptoms and cognitive function. Front Psychiatry. 2023;14:1180720. doi: 10.3389/fpsyt.2023.1180720.**
**Included**
341
Nakamura T, Yoshizawa T, Toya R, Terasawa M, Takahashi K, Kitazawa K, Suzuki K, Sasayama D, Washizuka S. Orexin receptor antagonists versus antipsychotics for the management of delirium in intensive care unit patients with cardiovascular disease: A retrospective observational study. Gen Hosp Psychiatry. 2023;84:96-101. doi: 10.1016/j.genhosppsych.2023.06.019. Epub 2023 Jul 1.
Unfocused
342
Hansen BH, Andresen HN, Gjesvik J, Thorsby PM, Naerland T, Knudsen-Heier S. Associations between psychiatric comorbid disorders and executive dysfunctions in hypocretin-1 deficient pediatric narcolepsy type1. Sleep Med. 2023;109:149-157. doi: 10.1016/j.sleep.2023.06.021. Epub 2023 Jul 1.
No psychosis
343
Yao Y, Baronio D, Chen YC, Jin C, Panula P. The roles of histamine receptor 1 (hrh1) in neurotransmitter system regulation, behavior, and neurogenesis in zebrafish. Mol Neurobiol. 2023;60(11):6660-6675. doi: 10.1007/s12035-023-03447-z. Epub 2023 Jul 20.
Animal
344
Watanabe K, Misaka S, Kanno-Nozaki K, Chiyoda T, Suzuki Y, Sato A, Suto T, Kuroda J, Shimomura K, Miura I, Yabe H. Effect of lemborexant on pharmacokinetics of clozapine: A potential drug-drug interaction mediated by time-dependent inhibition of CYP3A4. Br J Clin Pharmacol. 2024;90(1):354-359. doi: 10.1111/bcp.15889. Epub 2023 Sep 4.
Case
345
Harrison PJ, Bannerman DM. GRIN2A (NR2A): a gene contributing to glutamatergic involvement in schizophrenia. Mol Psychiatry. 2023;28(9):3568-3572. doi: 10.1038/s41380-023-02265-y. Epub 2023 Sep 22.
Opinion
346
Tong Z, Smith PJ, Pickford HD, Christensen KE, Anderson EA. Gold-catalyzed cyclization of yndiamides with isoxazoles via α-imino gold Fischer carbenes. Chemistry. 2023;29(70):e202302821. doi: 10.1002/chem.202302821. Epub 2023 Oct 25.
*In vitro*
347
Diepenbroek C, Rijnsburger M, van Irsen AAS, Eggels L, Kisner A, Foppen E, Unmehopa UA, Berland C, Dólleman S, Hardonk M, Cruciani-Guglielmacci C, Faust RP, Wenning R, Maya-Monteiro CM, Kalsbeek A, Aponte Y, Luquet S, Serlie MJM, la Fleur SE. Dopamine in the nucleus accumbens shell controls systemic glucose metabolism via the lateral hypothalamus and hepatic vagal innervation in rodents. Metabolism. 2024;150:155696. doi: 10.1016/j.metabol.2023.155696. Epub 2023 Oct 5.
Animal
348
Uematsu T, Tomita T, Obara R, Gonai T, Hattori K, Aonuma T, Usui K, Tanifuji H, Ishizawa F, Ishii H, Suzuki E. Reducing the use of psychotropics in a convalescent rehabilitation ward. Neuropsychopharmacol Rep. 2024;44(1):227-233. doi: 10.1002/npr2.12388. Epub 2023 Oct 26.
Unfocused
349
Montastruc F, Taillefer de Laportaliere T. Drug-induced psychiatric disorders: A pharmacovigilance update. Thérapie. 2024;79(2):173-179. doi: 10.1016/j.therap.2023.09.007. Epub 2023 Oct 31.
Review
350
Guaiana G, Meader N, Barbui C, Davies SJ, Furukawa TA, Imai H, Dias S, Caldwell DM, Koesters M, Tajika A, Bighelli I, Pompoli A, Cipriani A, Dawson S, Robertson L. Pharmacological treatments in panic disorder in adults: a network meta-analysis. Cochrane Database Syst Rev. 2023;11(11):CD012729. doi: 10.1002/14651858.CD012729.pub3.
Review
351

Riemann D, Espie CA, Altena E, Arnardottir ES, Baglioni C, Bassetti CLA, Bastien C, Berzina N, Bjorvatn B, Dikeos D, Dolenc Groselj L, Ellis JG, Garcia-Borreguero D, Geoffroy PA, Gjerstad M, Gonçalves M, Hertenstein E, Hoedlmoser K, Hion T, Holzinger B, Janku K, Jansson-Fröjmark M, Järnefelt H, Jernelöv S, Jennum PJ, Khachatryan S, Krone L, Kyle SD, Lancee J, Leger D, Lupusor A, Marques DR, Nissen C, Palagini L, Paunio T, Perogamvros L, Pevernagie D, Schabus M, Shochat T, Szentkiralyi A, Van Someren E, van Straten A, Wichniak A, Verbraecken J, Spiegelhalder K. The European Insomnia Guideline: An update on the diagnosis and treatment of insomnia 2023. J Sleep Res. 2023;32(6):e14035. doi: 10.1111/jsr.14035.
Review
352
Varadharajan A, Davis AD, Ghosh A, Jagtap T, Xavier A, Menon AJ, Roy D, Gandhi S, Gregor T. Guidelines for pharmacotherapy in Alzheimer's disease - A primer on FDA-approved drugs. J Neurosci Rural Pract. 2023;14(4):566-573. doi: 10.25259/JNRP_356_2023. Epub 2023 Oct 7.
Review
353
Cipriani A, Agunbiade A, Salanti G. Muscarinic drug shows efficacy in schizophrenia but much is left to be discovered. Lancet. 2024;403(10422):120-122. doi: 10.1016/S0140-6736(23)02415-7. Epub 2023 Dec 14.
Opinion
354
Hu J, Wei SS, Jiang HZ, Luo JY, Yang W, Zhang YM, Wang XB, Wen CN (胡金;韦姗姗;姜海洲;罗静怡;杨薇;张云敏;王欣波;文朝楠;来源). 失眠的药物治疗研究进展 [Research progress in pharmacotherapy of insomnia]. Zhongguo Zhong Yao Za Zhi (中国中药杂志). 2023;48(19):5122-5130. Chinese. doi: 10.19540/j.cnki.cjcmm.20230721.601.
Review
355
Efthimiou O, Taipale H, Radua J, Schneider-Thoma J, Pinzón-Espinosa J, Ortuño M, Vinkers CH, Mittendorfer-Rutz E, Cardoner N, Tanskanen A, Fusar-Poli P, Cipriani A, Vieta E, Leucht S, Tiihonen J, Luykx JJ. Efficacy and effectiveness of antipsychotics in schizophrenia: network meta-analyses combining evidence from randomised controlled trials and real-world data. Lancet Psychiatry. 2024;11(2):102-111. doi: 10.1016/S2215-0366(23)00366-8. Epub 2024 Jan 9.
Review
356
Piri F, Salmani ME, Sepehri H. Improvement of autistic-like behaviors in adult rats prenatally exposed to valproic acid through early suppression of orexin receptor. Ann Med Surg (Lond). 2023;86(1):166-171. doi: 10.1097/MS9.0000000000000788.
Animal
**357**
**Chen PY, Chiu CC, Chang CK, Lu ML, Huang CY, Chen CH, Huang MC. Higher orexin-A levels are associated with treatment response to clozapine in patients with schizophrenia: A cross-sectional study. J Psychopharmacol. 2024;38(3):258-267. doi: 10.1177/02698811231225610. Epub 2024 Jan 27.**
**Included**
358
Glen A, Bürli RW, Livermore D, Buffham W, Merison S, Rowland AE, Newman R, Fieldhouse C, Miller DJ, Dawson LA, Matthews K, Carlton MB, Brice NL. Discovery and first-time disclosure of CVN766, an exquisitely selective orexin 1 receptor antagonist. Bioorg Med Chem Lett. 2024;100:129629. doi: 10.1016/j.bmcl.2024.129629. Epub 2024 Jan 30.
*In vitro*
359
Mori Y, Watanabe K, Suzuki Y, Ono H, Tojo M, Kawasaki Y, Kanno-Nozaki K, Nozaki M, Miura I. Clozapine-associated myocarditis in a patient with schizophrenia taking lemborexant: A case report. J Clin Psychopharmacol. 2024;44(2):193-195. doi: 10.1097/JCP.0000000000001817. Epub 2024 Feb 7.
Case
360
Meyer N, Lok R, Schmidt C, Kyle SD, McClung CA, Cajochen C, Scheer FAJL, Jones MW, Chellappa SL. The sleep-circadian interface: A window into mental disorders. Proc Natl Acad Sci U S A. 2024;121(9):e2214756121. doi: 10.1073/pnas.2214756121. Epub 2024 Feb 23.
Review
361
Fornaro M, Caiazza C, Rossano F, Cilmi F, De Prisco M, Vieta E, Thompson T, Solmi M, Carvalho AF, Iasevoli F, de Bartolomeis A. Residual effects of medications for sleep disorders on driving performance: A systematic review and network meta-analysis of randomized controlled trials: NMA driving and hypnotics. Eur Neuropsychopharmacol. 2024;81:53-63. doi: 10.1016/j.euroneuro.2024.01.011. Epub 2024 Feb 23.
Review
362
Öz P, Kamalı O, Saka HB, Gör C, Uzbay İT. Baseline prepulse inhibition dependency of orexin A and REM sleep deprivation. Psychopharmacology (Berl). 2024;241(6):1213-1225. doi: 10.1007/s00213-024-06555-3. Epub 2024 Mar 1.
Animal
363
Mutz J, Wong WLE, Powell TR, Young AH, Dawe GS, Lewis CM. The duration of lithium use and biological ageing: telomere length, frailty, metabolomic age and all-cause mortality. Geroscience. 2024;46(6):5981-5994. doi: 10.1007/s11357-024-01142-y. Epub 2024 Mar 28.
No orexin
364
Aubin H-J. Repurposing drugs for treatment of alcohol use disorder. Int Rev Neurobiol. 2024;175:153-185. doi: 10.1016/bs.irn.2024.02.002. Epub 2024 Mar 12.
Review
365
Haniff ZR, Bocharova M, Mantingh T, Rucker JJ, Velayudhan L, Taylor DM, Young AH, Aarsland D, Vernon AC, Thuret S. Psilocybin for dementia prevention? The potential role of psilocybin to alter mechanisms associated with major depression and neurodegenerative diseases. Pharmacol Ther. 2024;258:108641. doi: 10.1016/j.pharmthera.2024.108641. Epub 2024 Apr 6.
Review
366
Álamo C, Sáiz Ruiz J, Zaragozá Arnáez C. Orexinergic receptor antagonists as a new therapeutic target to overcome limitations of current pharmacological treatment of insomnia disorder. Actas Esp Psiquiatr. 2024;52(2):172-182. doi: 10.62641/aep.v52i2.1659.
Review
367

Dolz M, Tor J, Puig O, de la Serna E, Muñoz-Samons D, Pardo M, Alvarez-Subiela X, Rodriguez-Pascual M, Sugranyes G, Ilzarbe D, Baeza I. Clinical and neurodevelopmental predictors of psychotic disorders in children and adolescents at clinical high risk for psychosis: the CAPRIS study. Eur Child Adolesc Psychiatry. 2024;33(11):3925-3935. doi: 10.1007/s00787-024-02436-4. Epub 2024 Apr 20.
No orexin
368
Jelen LA, McShane R, Young AH. Guidelines for ketamine use in clinical psychiatry practice. BJPsych Open. 2024;10(3):e107. doi: 10.1192/bjo.2024.62.
Opinion
369
Thangwaritorn S, Lee C, Metchikoff E, Razdan V, Ghafary S, Rivera D, Pinto A, Pemminati S. A review of recent advances in the management of Alzheimer's disease. Cureus. 2024;16(4):e58416. doi: 10.7759/cureus.58416.
Review
370
Lodge P. "Pyrrhonism" as a therapeutic response to the allure of mania. Schizophr Bull. 2024:sbae068. doi: 10.1093/schbul/sbae068. Epub ahead of Print 2024 May 24.
Opinion
371
Nakamura T, Furihata R, Hasegawa N, Kodaka F, Muraoka H, Ichihashi K, Ochi S, Numata S, Tsuboi T, Makinodan M, Iida H, Onitsuka T, Kashiwagi H, Takeshima M, Hashimoto N, Nagasawa T, Usami M, Yamagata H, Takaesu Y, Miura K, Matsumoto J, Ohi K, Yamada H, Hori H, Inada K, Watanabe K, Hashimoto R, Yasui-Furukori N. The effect of education regarding treatment guidelines for schizophrenia and major depressive disorders on psychiatrists' hypnotic medication prescribing behavior: a multicenter study. BMC Psychiatry. 2024;24(1):399. doi: 10.1186/s12888-024-05816-x.
Unfocused
372
Jia R, Coupland C, Vinogradova Y, Qureshi N, Turner E, Vedhara K. Mental health conditions and COVID-19 vaccine outcomes: A scoping review. J Psychosom Res. 2024;183:111826. doi: 10.1016/j.jpsychores.2024.111826. Epub 2024 Jun 8.
Review
373
Rogdaki M, McCutcheon RA, D'Ambrosio E, Mancini V, Watson CJ, Fanshawe JB, Carr R, Telesia L, Martini MG, Philip A, Gilbert BJ, Salazar-de-Pablo G, Kyriakopoulos M, Siskind D, Correll CU, Cipriani A, Efthimiou O, Howes OD, Pillinger T. Comparative physiological effects of antipsychotic drugs in children and young people: a network meta-analysis. Lancet Child Adolesc Health. 2024;8(7):510-521. doi: 10.1016/S2352-4642(24)00098-1.
Review
374
Kukkonen JP, Jacobson LH, Hoyer D, Rinne MK, Borgland SL. International Union of Basic and Clinical Pharmacology CXIV: Orexin receptor function, nomenclature and pharmacology. Pharmacol Rev. 2024;76(5):625-688. doi: 10.1124/pharmrev.123.000953.
Review
375
Malafouris L, Röhricht F. Re-*thinging* embodied and enactive psychiatry: A material engagement approach. Cult Med Psychiatry. 2024;48(4):816-839. doi: 10.1007/s11013-024-09872-6. Epub 2024 Jul 19.
Opinion
376
Wei S, Freeman D, Harris V, Rovira A. A randomised controlled test in virtual reality of the effects on paranoid thoughts of virtual humans' facial animation and expression. Sci Rep. 2024;14(1):17102. doi: 10.1038/s41598-024-67534-4.
No orexin
377
Hong JSW, Ostinelli EG, Kamvar R, Smith KA, Walsh AEL, Kabir T, Tomlinson A, Cipriani A. An online evidence-based dictionary of common adverse events of antidepressants: a new tool to empower patients and clinicians in their shared decision-making process. BMC Psychiatry. 2024;24(1):532. doi: 10.1186/s12888-024-05950-6.
Unrelated
378
Kikuchi Y, Kurosawa M, Sakata M, Takahashi Y, Yamamoto K, Tomita H, Yoshio T, Yasui-Furukori N. Effects of titration speed, gender, obesity and concomitant medications on the risk and onset time of clozapine-associated fever among Japanese patients with schizophrenia: retrospective review of charts from 21 hospitals. Br J Psychiatry. 2024;225(5):492-498. doi: 10.1192/bjp.2024.113.
Unfocused
379
Cao F, Guo Z, Ma X, Li X, Wang Q. Regulation of neuronal plasticity associated with neuropsychiatric disorders by the orexinergic system. Heliyon. 2024;10(14):e34182. doi: 10.1016/j.heliyon.2024.e34182.
Review
380
Ghazi-Noori AR, Woodham RD, Rezaei H, Sharif MS, Bramon E, Ritter P, Bauer M, Young AH, Fu CHY. Home-based transcranial direct current stimulation in bipolar depression: an open-label treatment study of clinical outcomes, acceptability and adverse events. Int J Bipolar Disord. 2024;12(1):30. doi: 10.1186/s40345-024-00352-9.
No orexin
381
Dumont S, Bloch V, Lillo-Lelouet A, Le Beller C, Geoffroy PA, Veyrier M. Parasomnias and sleep-related movement disorders induced by drugs in the adult population: a review about iatrogenic medication effects. J Sleep Res. 2025;34(2):e14306. doi: 10.1111/jsr.14306. Epub 2024 Sep 7.
Review
382
Xiao W, Moncy JC, Ghazi-Noori AR, Woodham RD, Rezaei H, Bramon E, Ritter P, Bauer M, Young AH, Fu CHY. Enhanced network synchronization connectivity following transcranial direct current stimulation (tDCS) in bipolar depression: Effects on EEG oscillations and deep learning-based predictors of clinical remission. J Affect Disord. 2025;369:576-587. doi: 10.1016/j.jad.2024.09.054. Epub 2024 Sep 16.
Unrelated
383
Nozu T, Miyagishi S, Ishioh M, Takakusaki K, Okumura T. The neurotensin receptor 1 agonist PD149163 alleviates visceral hypersensitivity and colonic hyperpermeability in rat irritable bowel syndrome model. Neurogastroenterol Motil. 2024;36(12):e14925. doi: 10.1111/nmo.14925. Epub 2024 Sep 24.
Animal
384
Guo J, Guo J, Rao X, Zhang R, Li Q, Zhang K, Ma S, Zhao J, Ji C. Exploring the pathogenesis of insomnia and acupuncture intervention strategies based on the microbiota-gut-brain axis. Front Microbiol. 2024;15:1456848. doi: 10.3389/fmicb.2024.1456848.

Review
385
Vadisiute A, Meijer E, Therpurakal RN, Mueller M, Szabó F, Messore F, Jursenas A, Bredemeyer O, Krone LB, Mann E, Vyazovskiy V, Hoerder-Suabedissen A, Molnár Z. Glial cells undergo rapid changes following acute chemogenetic manipulation of cortical layer 5 projection neurons. Commun Biol. 2024;7(1):1286. doi: 10.1038/s42003-024-06994-w.
Animal
386
Haubjerg Østerby NC, Baandrup L, Jennum PJ. Psychiatric comorbidity in Danish patients with narcolepsy type 1, narcolepsy type 2, and idiopathic hypersomnia: a case-control study. Sleep Adv. 2024;5(1):zpae073. doi: 10.1093/sleepadvances/zpae073.
No antipsychotic
387
Woodham RD, Selvaraj S, Lajmi N, Hobday H, Sheehan G, Ghazi-Noori AR, Lagerberg PJ, Rizvi M, Kwon SS, Orhii P, Maislin D, Hernandez L, Machado-Vieira R, Soares JC, Young AH, Fu CHY. Home-based transcranial direct current stimulation treatment for major depressive disorder: a fully remote phase 2 randomized sham-controlled trial. Nat Med. 2025;31(1):87-95. doi: 10.1038/s41591-024-03305-y. Epub 2024 Oct 21.
No orexin
388
Mori K, Kimura M, Usami E. Short-term efficacy and safety of suvorexant and lemborexant: A retrospective study. Cureus. 2024;16(10):e71049. doi: 10.7759/cureus.71049.
Unfocused
389
Vringer M, Bijlenga D, Zhou J, Meijer OC, Vinkers CH, Lammers GJ, Fronczek R. Physiological and psychological stress reactivity in narcolepsy type 1. Sleep. 2025;48(3):zsae265. doi: 10.1093/sleep/zsae265.
No psychosis
390
Geldmacher DS. Treatment of Alzheimer disease. Continuum (Minneap Minn). 2024;30(6):1823-1844. doi: 10.1212/CON.0000000000001503.
Review
391
Rezaei H, Woodham RD, Ghazi-Noori AR, Ritter P, Bramon E, Bauer M, Young AH, Fu CHY. Effect of home-based transcranial direct current stimulation (tDCS) on cognitive functioning in bipolar depression. Res Sq [Preprint]. 2024:rs.3.rs-5396838. doi: 10.21203/rs.3.rs-5396838/v1. Update in: Int J Bipolar Disord. 2025;13(1):11. doi: 10.1186/s40345-025-00376-9.
Dupl 386PM
392
Chekani F, Mirchandani K, Zaki S, Goswami S, Sharma M. Utilization of potentially inappropriate sedative-hypnotic and atypical antipsychotic medications among elderly individuals with insomnia and Alzheimer's disease. Sleep. 2025;48(4):zsaf003. doi: 10.1093/sleep/zsaf003.
Unfocused
393
Xie X, Xu H, Shu R, Du S, Fan H, Zhang M, Sun L, Zhou J, Wang L, Li Z, Anthony DC. Period3 modulates the $NAD^+$-SIRT3 axis to alleviate depression-like behaviour by enhancing NAMPT activity in mice. J Adv Res. 2025;77:309-320. doi: 10.1016/j.jare.2025.01.043. Epub 2025 Feb 1.
Animal
394
Ferini-Strambi L. Insomnia disorder. Minerva Med. 2025;116(4):309-322. doi: 10.23736/S0026-4806.25.09690-9. Epub 2025 Feb 11.
Review
395
Arnone D, Ramaraj R, Östlundh L, Arora T, Javaid S, Govender RD, Stip E, Young AH. Assessment of cognitive domains in major depressive disorders using the Cambridge Neuropsychological Test Automated Battery (CANTAB): Systematic review and meta-analysis of cross-sectional and longitudinal studies. Prog Neuropsychopharmacol Biol Psychiatry. 2025;138:111301. doi: 10.1016/j.pnpbp.2025.111301. Epub 2025 Feb 24.
No orexin
396
Pozuelo Moyano B, Gomez Bautista D, Porras Ibarra KJ, Mueller C, von Gunten A, Vandel P, Ranjbar S, Howard R, Young AH, Stewart R, Reeves S, Orgeta V; European Task Force for treatment resistant depression in older people. Systematic review of clinical effectiveness of interventions for treatment resistant late-life depression. Ageing Res Rev. 2025;107:102710. doi: 10.1016/j.arr.2025.102710. Epub 2025 Feb 28.
Review
397
Rezaei H, Woodham RD, Ghazi-Noori AR, Ritter P, Bramon E, Bauer M, Young AH, Fu CHY. Effect of home-based transcranial direct current stimulation (tDCS) on cognitive functioning in bipolar depression: an open-label, single-arm acceptability and feasibility study. Int J Bipolar Disord. 2025;13(1):11. doi: 10.1186/s40345-025-00376-9.
No orexin
398
Nozu T, Miyagishi S, Ishioh M, Takakusaki K, Okumura T. Irisin prevents visceral hypersensitivity and colonic hyperpermeability in a rat model of irritable bowel syndrome. Peptides. 2025;188:171394. doi: 10.1016/j.peptides.2025.171394. Epub 2025 Mar 26.
Animal
399
Woodham RD, Selvaraj S, Lajmi N, Hobday H, Sheehan G, Ghazi-Noori AR, Lagerberg PJ, Machado-Vieira R, Soares JC, Young AH, Fu CHY. Home-based transcranial direct current stimulation for major depressive disorder: 6-month follow-up from randomised sham-controlled trial and open-label treatment phases. J Psychiatr Res. 2025;186:23-32. doi: 10.1016/j.jpsychires.2025.03.047. Epub 2025 Mar 26.
No orexin
400
Rezaei H, Woodham RD, Ghazi-Noori AR, Ritter P, Bauer M, Young AH, Bramon E, Fu CHY. Acceptability of Home-Based Transcranial Direct Current Stimulation (tDCS) in Bipolar Depression: Thematic Analysis of Individual Views. Res Sq [Preprint]. 2025 Apr 15:rs.3.rs-5967699. doi: 10.21203/rs.3.rs-5967699/v1. Update in: BMC Psychiatry. 2025;25(1):549. doi: 10.1186/s12888-025-06948-4.
Dupl 391PM
401
Attaallah B, Petitet P, Husain M. Active information sampling in health and disease. Neurosci Biobehav Rev. 2025;175:106197. doi: 10.1016/j.neubiorev.2025.106197. Epub 2025 May 3.
Review
402

Beech MJ, Toma EC, Smith HG, Trush MM, Ang JHJ, Wong MY, Wong CHJ, Ali HS, Butt Z, Goel V, Duarte F, Farley AJM, Walsh TR, Schofield CJ. Binding assays enable discovery of Tet(X) inhibitors that combat tetracycline destructase resistance. Chem Sci. 2025;16(22):9691-9704. doi: 10.1039/d5sc00964b.

*In vitro*

403

Rezaei H, Woodham RD, Ghazi-Noori AR, Ritter P, Bauer M, Young AH, Bramon E, Fu CHY. Acceptability of home-based transcranial direct current stimulation (tDCS) in bipolar depression: thematic analysis of individual views. BMC Psychiatry. 2025;25(1):549. doi: 10.1186/s12888-025-06948-4.

No orexin

404

Beckley A, Glogowska M, Waite F, Bee P, Freeman D. Research assistants' experiences recruiting patients with psychosis into clinical trials: a qualitative study. Trials. 2025;26(1):180. doi: 10.1186/s13063-025-08882-y.

No orexin

405

Jenner L, Payne M, Waite F, Beckwith H, Diamond R, Isham L, Collett N, Emsley R, Freeman D. Learning how to improve the treatment of persecutory delusions: Using a principal trajectories analysis to examine differential effects of two psychological interventions (Feeling Safe, Befriending) in distinct groups of patients. Schizophr Bull. 2026;52:sbaf083. doi: 10.1093/schbul/sbaf083. Epub ahead of print 2025 Jun 17.

No orexin

406

Mori K, Ohashi K, Kimura M, Yoshida M, Tomida K, Usami E (森 光輝，大橋健吾，木村美智男，吉田光代，富田顕旨，宇佐美英績). オレキシン受容体拮抗薬推進活動が睡眠薬・抗精神病薬の処方動向に与える影響：分割時系列解析研究 [Impact of promotional activities on orexin receptor antagonists prescription rates and usage of sleep and antipsychotic medications: An interrupted time-series analysis study]. Yakugaku Zasshi. 2025;145(7):629-637. Japanese. doi: 10.1248/yakushi.24-00170.

Unfocused

407

Takahashi K, Kiryu K, Harada H, Kato T, Tamune H. Management of subsyndromal delirium with daridorexant and quetiapine: A case report. Cureus. 2025;17(6):e85232. doi: 10.7759/cureus.85232.

Case

408

Liaskopoulos A, Kakouris V, Liaskopoulos N, Lappas AS, Christodoulou N, Samara M. Medical professionals and pharmacological intervention for the treatment of insomnia: A cross-sectional study. Sleep Sci. 2024;18(2):e155-e164. doi: 10.1055/s-0044-1791238.

Unfocused

409

Berger M, Helter T, Azim L, Chadwick T, Courtney P, Fouweather T, Geddes J, Hindmarch P, Morriss R, Stokes PRA, Watson S, Weetman C, Young AH, McAllister-Williams RH, Simon J. Cost-effectiveness of pramipexole in addition to mood stabilisers for patients with treatment-resistant bipolar depression: Economic evaluation of the PAX-BD randomised controlled trial. J Affect Disord. 2025;391:119937. doi: 10.1016/j.jad.2025.119937. Epub 2025 Jul 18.

No orexin

410

Jelen LA, Lythgoe DJ, Stone JM, Young AH, Mehta MA. Effect of naltrexone pretreatment on ketamine-induced glutamatergic activity and symptoms of depression: a randomized crossover study. Nat Med. 2025;31(9):2958-2966. doi: 10.1038/s41591-025-03800-w. Epub 2025 Jul 24.

No orexin

411

Kim TH, Lee K, Hwang J, Lee S, Jo H, Cho H, Lee H, Baek HJ, Kang J, Nehs CJ, Fond G, Boyer L, Chung EK, Yon DK. Adverse event reports of seizure for insomnia medication from 1967 to 2023. Sci Rep. 2025;15(1):27308. doi: 10.1038/s41598-025-11314-1.

Unfocused

412

Rezaei H, Woodham RD, Ghazi-Noori AR, Bramon E, Bauer M, Young AH, Fu CHY, Ritter P. Home-based transcranial direct current stimulation (tDCS) for bipolar depression: Effects on quality of life and functioning: an open-label study. Res Sq [Preprint]. 2025:rs.3.rs-7186400. doi: 10.21203/rs.3.rs-7186400/v1.

Dupl 420PM

413

Imran R, Kenny A, Wong G, Lee C. The role of link workers in weight management for people with severe mental illness: a qualitative study. BMC Prim Care. 2025;26(1):251. doi: 10.1186/s12875-025-02929-4.

Unrelated

414

Jauhar S, McCutcheon RA, Nour MM, Veronese M, Rogdaki M, Bonoldi I, Azis M, Whitehurst T, Arumuham A, Onwordi E, Turkheimer F, McGuire P, Young AH, Howes OD. Dopamine and mood in psychotic disorders: An $^{18}$F-DOPA PET study. JAMA Psychiatry. 2025;82(10):1009-1014. doi: 10.1001/jamapsychiatry.2025.1811. Epub ahead of print 2025 Aug 13.

No orexin

415

Todd KL, Cramb KML, Brimblecombe KR, Cragg SJ. New insights into axonal regulators of dopamine transmission in health and disease. Curr Opin Neurobiol. 2025;94:103093. doi: 10.1016/j.conb.2025.103093. Epub 2025 Aug 16.

Review

416

Nedeljkovic-Kurepa A, Abraham MN, Fernandes TD, Yaipen O, Brewer MR, Taylor MD, Pavlov VA, Deutschman CS. Loss of M1 Acetylcholine receptor-mediated orexinergic activity contributes to immune dysfunction in experimental sepsis. Res Sq [Preprint]. 2025:rs.3.rs-7329263. doi: 10.21203/rs.3.rs-7329263/v1.

Animal

417

Yamaguchi J, Sadahiro R, Wada S, Nishikawa E, Terada T, Nakahara R, Matsuoka H. Delirium derived from dementia with Lewy bodies in the cancer perioperative period: a case report. Ann Palliat Med. 2025;14(5):508-513. doi: 10.21037/apm-25-48.

Case

418

Chinoy J, Meller C, de Wet H. An emerging paradigm for ABCC5/MRP5 function in human physiology. Int J Mol Sci. 2025;26(18):9211. doi: 10.3390/ijms26189211.

Review

419

Volgin AD, Cheresiz SV, Chizhova ND, Smirnova KV, Doroshkov AV, Galstya DS, Abreu MS, Strekalova T, Lipina T, Pletnikov M, Yang L, Lim LW, Stewart AM, Amstislavskaya TG, Kalueff AV. Rethinking the role of *DISC1* in CNS function: Translational cross-taxon insights from rodent and zebrafish models. J Integr Neurosci. 2025;24(9):43162. doi: 10.31083/JIN43162.
Review
420
Nutt DJ, Morgan C, Erritzoe D, Greenway KT, Young AH. A long, strange trip: Ketamine treatment in psychiatry. J Psychopharmacol. 2025;39(10):1039-1044. doi: 10.1177/02698811251379393. Epub 2025 Oct 27.
Opinion
421
Arnone D, Östlundh L, Mosa M, MacDonald B, Oldershaw J, Qassem T, Young AH. Efficacy of lamotrigine in the treatment of unipolar and bipolar depression: Meta-analysis of acute and maintenance randomised controlled trials. Pharmaceuticals (Basel). 2025;18(10):1590. doi: 10.3390/ph18101590.
Review
422
Højlund M, Kafali HY, Kırmızı B, Fusar-Poli P, Correll CU, Cortese S, Sabé M, Fiedorowicz J, Saraf G, Zein J, Berk M, Husain MI, Rosenblat JD, Rubaiyat R, Corace K, Wong S, Hatcher S, Kaluzienski M, Yatham LN, Cipriani A, Gosling CJ, Carhart-Harris R, Tanuseputro P, Myran DT, Fabiano N, Moher D, Mayo LM, Nicholls SG, White T, Prisco M, Radua J, Vieta E, Ladha KS, Katz J, Veroniki AA, Solmi M. Efficacy, all-cause discontinuation, and safety of serotonergic psychedelics and MDMA to treat mental disorders: A living systematic review with meta-analysis. Eur Neuropsychopharmacol. 2025;101:41-55. doi: 10.1016/j.euroneuro.2025.09.011. Epub 2025 Nov 7.
Review
423
Ma W, Warnhoff I, Stephan M, Ma X, Dehne K, Volkmann P, Kannaiyan N, Brankatschk B, Jensen N, Rossner MJ, Scheuss V, Wehr MC. TAOK2 controls synaptic plasticity and anxiety via ERK and calcium signaling. iScience. 2025;28(11):113712. doi: 10.1016/j.isci.2025.113712.
Animal
424
Kono S, Nishiyama T, Nakano S, Ishimoto Y, Horikoshi S. Real-world effectiveness and safety of daridorexant in Japanese patients with insomnia: A multicenter observational study evaluating four-week changes in Athens Insomnia Scale among 54 participants. Cureus. 2025;17(11):e97174. doi: 10.7759/cureus.97174.
No psychosis
425
Ryan S, Munir S, Davies A. Pharmacological interventions for sleep disturbance ("insomnia") in patients with advanced cancer receiving specialist palliative care: a scoping review. Support Care Cancer. 2025;33(12):1124. doi: 10.1007/s00520-025-10202-8.
Review
426
Dong X, Aveyard P, Yang X, Kivimaki M, Chen S, Firth J, Drakesmith CW, Gao M. Adiposity and the first-onset of diagnosed mental illnesses: a population-based cohort study of 10 million UK adults. BMC Med. 2025. doi: 10.1186/s12916-025-04514-z. Epub ahead of print 2025 Nov 29.
No orexin
**427**
**Tanaka A, Arai Y, Yasaki T, Saito K, Nakashizuka E, Yoshida S, Nakajima Y, Koido M, Suzuki K, Nakamura T, Sasayama D, Washizuka S. Longitudinal associations of serum orexin-A with physical activity and sleep in schizophrenia: A preliminary study. Neuropsychopharmacol Rep. 2025;45(4):e70079. doi: 10.1002/npr2.70079.**
**Included**
428
Lagerberg PJ, Woodham RD, Selvaraj S, Lajmi N, Hobday H, Sheehan G, Ghazi-Noori AR, Rizvi M, Kwon SS, Orhii P, Machado-Vieira R, Soares JC, Young AH, Fidalgo AR, Rezaei H, Fu CHY. Acceptability of active and sham home-based transcranial direct current stimulation in major depression: mixed methods qualitative analysis in a randomised controlled trial. Ann Gen Psychiatry. 2026;25(1):1. doi: 10.1186/s12991-025-00607-4. Epub ahead of print 2025 Dec 4.
No orexin
429
Willard J, Velez D, Baez CS, Palladino C, Pavkovic I, Varughese RT, Kothare SV. Tactile hallucinations in pediatric and young adults with narcolepsy: A case series and review of the literature. Pediatr Neurol. 2025;175:151-155. doi: 10.1016/j.pediatrneurol.2025.11.009. Epub ahead of print 2025 Nov 14.
Case
430
Diamond R, Waite F, Boylan AM, Hicks A, Kabir T, Freeman D. Supporting patients with psychosis in the community to stand up and move more: Perspectives of community mental health staff. Community Ment Health J. 2025. doi: 10.1007/s10597-025-01563-9. Epub ahead of print 2025 Dec 27.
Unrelated
431
Moncy JC, Xiao W, Woodham RD, Ghazi-Noori AR, Rezaei H, Bramon E, Ritter P, Bauer M, Young AH, Fan Y, Fu CHY. Deep learning based treatment remission prediction to transcranial direct current stimulation in bipolar depression using EEG power spectral density. Psychiatry Res Neuroimaging. 2026;357:112115. doi: 10.1016/j.pscychresns.2025.112115. Epub ahead of print 2025 Dec 22.
No orexin
432
Rezaei H, Woodham RD, Ghazi-Noori AR, Bramon E, Bauer M, Young AH, Fu CHY, Ritter P. Home-based transcranial direct current stimulation (tDCS) for bipolar depression: effects on quality of life and functioning-an open-label study. Qual Life Res. 2026;35(2):33. doi: 10.1007/s11136-025-04135-2.
Unrelated
433
Hampsey E, Kalfas M, Carter L, Bloomfield M, Rezaei H, Young AH, Fu CHY. A systematic review of transcranial electrical stimulation and meta-analysis of transcranial direct current stimulation RCTs in unipolar and bipolar depression. J Affect Disord. 2026;400:121009. doi: 10.1016/j.jad.2025.121009. Epub ahead of print 2026 Jan 9.
Review
434
Almahwzi S, Ghabashi S, Alyami W, Alsolami J, Juma R, Alghamdi S, Althobyane L, Alqahtani M, Alotaibi M. Circulating orexin-A levels in patients with schizophrenia, bipolar disorder, and major depressive disorder: A systematic review and meta-analysis. Cureus. 2026;18(1):e101525. doi: 10.7759/cureus.101525.
Review
435
Kas MJH, Do KQ, Sand MS, Kozak R, Tunbridge EM, Oquendo MA, Tamminga C, Koutsouleris N, Knudsen GM, Penninx BWJH, Padberg FJ, Drevets WC, Falkai P, Buhl DL, Reif A. Biomarker innovations in precision psychiatry diagnostics and treatment strategies. Eur Neuropsychopharmacol. 2026;105:112762. doi: 10.1016/j.euroneuro.2026.112762. Epub ahead of print 2026 Jan 15.

Opinion
436
De Zoysa AI, Govinnage J, Giorlando F, Dodd S, Narayanaswamy JC, Berk M. Emerging neurobiological targets in psychiatric treatment. Eur Neuropsychopharmacol. 2026;105:112767. doi: 10.1016/j.euroneuro.2026.112767. Epub ahead of print 2026 Jan 27.
Review
437
Burckhardt A, Rakowsky A, Kahl E, Morchhale S, Mayer D, Panagiotou N, Permien L, Faesel N, Fendt M. Differential effects of orexin system activation on dizocilpine-induced schizophrenia-like behaviors in mice. Neuropeptides. 2026;116:102587. doi: 10.1016/j.npep.2026.102587. Epub ahead of print 2026 Jan 28.
Animal
438
Shimohara Y, Matsuda T, Iwata K. Effects of dual orexin receptor antagonists on seizure quality in modified electroconvulsive therapy: A pilot study. J ECT. 2026. doi: 10.1097/YCT.0000000000001223. Epub ahead of print 2026 Feb 4.
Unfocused
439
Hyndych A, Koval K, Dzeruzhynska N, Mader EC. Sleep and psychiatric disorders: Bidirectional interactions and shared neurobiological mechanisms. PLoS Ment Health. 2025;2(12):e0000531. doi: 10.1371/journal.pmen.0000531.
Review
440
Gnazzo M, Pisanò G, Baldini V, Citeroni F, Canulli F, De Ronchi D, Pizza F, Plazzi G. Unravelling narcolepsy: A series of complex pediatric cases. Neurol Clin Pract. 2026;16(2):e200583. doi: 10.1212/CPJ.0000000000200583. Epub 2026 Jan 28.
Case
441
von Fabeck K, Boudsocq JP, Boye M; French PCC Research Group; Simon N. Clinical toxicity of daridorexant: a retrospective analysis of poison centre data. Clin Toxicol (Phila). 2026:1-6. doi: 10.1080/15563650.2026.2617481. Epub ahead of print Feb 17.
No psychosis

( TITLE-ABS-KEY ( schizophrenia OR schizophreniform OR psychosis OR psychotic OR paranoid OR schizoaffective OR antipsychotic OR neuroleptic OR phenothiazine OR butyrophenone OR dopamine antagonist OR dopamine receptor antagonist OR substituted benzamide OR thioxanthene OR dibenzoazepine OR benzisoxazole OR haloperidol OR chlorpromazine OR promazine OR thioridazine OR clothiapine OR loxapine OR clozapine OR quetiapine OR olanzapine OR fluperlapine OR zotepine OR remoxipride OR sertindole OR risperidone OR paliperidone OR aripiprazole OR brexpiprazole OR cariprazine OR ziprasidone OR asenapine OR bromperidol OR spiroperidol OR spiperone OR perphenazine OR fluphenazine OR flupentixol OR clopenthixol OR zuclopenthixol OR droperidol OR thioxanthene OR sulpiride OR sultopride OR metoclopramide OR amisulpride ) AND TITLE-ABS-KEY ( orexin OR orexins OR hypocretin OR hypocretins OR Hcrts OR OX1 OR OX2 OR OX1R OR OX2R OR suvorexant OR MK-4305 OR lemborexant OR daridorexant OR almorexant OR ACT-078573 OR filorexant OR MK-6096 OR seltorexant OR MIN-202 OR JNJ-42847922 OR JNJ-922 OR Fazamorexant OR YZJ-1139 OR Nivasorexant OR ACT-539313 OR Tebideutorexant OR JNJ-61393215 OR JNJ-3215 OR Vornorexant OR ORN-0829 OR TS-142 OR ACT-335827 OR EMPA OR GSK-649868 OR SB-649868 OR JNJ-10397049 OR RTIOX-276 OR SB-334867 OR SB-408124 OR TCS-OX2-29 ) ) Scopus 3.3.2026 → 99 results

1
Telegdy G, Adamik A. The action of orexin A on passive avoidance learning. Involvement of transmitters. Regul Pept. 2002;104(1-3):105-10. doi: 10.1016/s0167-0115(01)00341-x.
Dupl 27 PM
2
Monda M, Viggiano A, De Luca V. Haloperidol reduces the sympathetic and thermogenic activation induced by orexin A. Neurosci Res. 2003;45(1):17-23. doi: 10.1016/s0168-0102(02)00191-8.
Dupl 33 PM
3
Winsky-Sommerer R, Boutrel B, De Lecea L. The role of the hypocretinergic system in the integration of networks that dictate the states of arousal. Drug News Perspect. 2003;16(8):504-12. doi: 10.1358/dnp.2003.16.8.829349.
Review
4
Jandacek RJ, Woods SC. Pharmaceutical approaches to the treatment of obesity. Drug Discov Today. 2004;9(20):874-80. doi: 10.1016/S1359-6446(04)03244-1.
Review
5
Rasmussen K, Benvenga MJ, Bymaster FP, Calligaro DO, Cohen IR, Falcone JF, Hemrick-Luecke SK, Martin FM, Moore NA, Nisenbaum LK, Schaus JM, Sundquist SJ, Tupper DE, Wiernicki TR, Nelson DL. Preclinical pharmacology of FMPD [6-fluoro-10-[3-(2-methoxyethyl)-4-methyl-piperazin-1-yl]-2-methyl-4H-3-thia-4,9-diaza-benzo[f]azulene]: a potential novel antipsychotic with lower histamine H1 receptor affinity than olanzapine. J Pharmacol Exp Ther. 2005;315(3):1265-77. doi: 10.1124/jpet.105.089326. Epub 2005 Sep 1.
Dupl 57PM
6
Alexander SP, Mathie A, Peters JA. Guide to receptors and channels, 1st edition (2005 revision). Br J Pharmacol. 2005;144(Suppl 1):S1-128. doi: 10.1038/sj.bjp.0706158.
Review
7
Bubser M, Fadel JR, Jackson LL, Meador-Woodruff JH, Jing D, Deutch AY. Dopaminergic regulation of orexin neurons. Eur J Neurosci. 2005;21(11):2993-3001. doi: 10.1111/j.1460-9568.2005.04121.x.
Dupl 54PM
8
Narita M, Nagumo Y, Hashimoto S, Narita M, Khotib J, Miyatake M, Sakurai T, Yanagisawa M, Nakamachi T, Shioda S, Suzuki T. Direct involvement of orexinergic systems in the activation of the mesolimbic dopamine pathway and related behaviors induced by morphine. J Neurosci. 2006;26(2):398-405. doi: 10.1523/JNEUROSCI.2761-05.2006.
Dupl 59 PM
9
Steffen KJ, Roerig JL, Mitchell JE, Uppala S. Emerging drugs for eating disorder treatment. Expert Opin Emerg Drugs. 2006;11(2):315-36. doi: 10.1517/14728214.11.2.315.
Dupl 61PM
10
Strachan RT, Ferrara G, Roth BL. Screening the receptorome: an efficient approach for drug discovery and target validation. Drug Discov Today. 2006;11(15-16):708-16. doi: 10.1016/j.drudis.2006.06.012.
Review

11
Alberto CO, Trask RB, Quinlan ME, Hirasawa M. Bidirectional dopaminergic modulation of excitatory synaptic transmission in orexin neurons. J Neurosci. 2006;26(39):10043-50. doi: 10.1523/JNEUROSCI.1819-06.2006. Retraction in: J Neurosci. 2012;32(26):9116. doi: 10.1523/JNEUROSCI.1889-12.2012.
Dupl 65PM
12
Rasmussen K, Hsu MA, Yang Y. The orexin-1 receptor antagonist SB-334867 blocks the effects of antipsychotics on the activity of A9 and A10 dopamine neurons: implications for antipsychotic therapy. Neuropsychopharmacology. 2007;32(4):786-92. doi: 10.1038/sj.npp.1301239. Epub 2006 Oct 25.
Dupl 68PM
13
Rasmussen K, Hsu MA, Noone S, Johnson BG, Thompson LK, Hemrick-Luecke SK. The orexin-1 antagonist SB-334867 blocks antipsychotic treatment emergent catalepsy: implications for the treatment of extrapyramidal symptoms. Schizophr Bull. 2007;33(6):1291-7. doi: 10.1093/schbul/sbm087. Epub 2007 Jul 28.
Dupl 77PM
14
Deutch AY, Bubser M. The orexins/hypocretins and schizophrenia. Schizophr Bull. 2007;33(6):1277-83. doi: 10.1093/schbul/sbm096. Epub 2007 Aug 28.
Dupl 79PM
15
Sullivan SS, Guilleminault C. Emerging drugs for insomnia: new frontiers for old and novel targets. Expert Opin Emerg Drugs. 2009;14(3):411-22. doi: 10.1517/14728210903171948.
Review
16
Eriksson KS, Sergeeva OA, Haas HL, Selbach O. Orexins/hypocretins and aminergic systems. Acta Physiol (Oxf). 2010;198(3):263-75. doi: 10.1111/j.1748-1716.2009.02015.x. Epub 2009 Jun 27.
Review
17
Suzuki G, Satow A, Ohta H. Effect of CFMTI, an allosteric metabotropic glutamate receptor 1 antagonist with antipsychotic activity, on Fos expression in regions of the brain related to schizophrenia. Neuroscience. 2010;168(3):787-96. doi: 10.1016/j.neuroscience.2010.04.016. Epub 2010 Apr 18.
Dupl 99PM
18
Milella MS, Passarelli F, De Carolis L, Schepisi C, Nativio P, Scaccianoce S, Nencini P. Opposite roles of dopamine and orexin in quinpirole-induced excessive drinking: a rat model of psychotic polydipsia. Psychopharmacology (Berl). 2010;211(3):355-66. doi: 10.1007/s00213-010-1909-5. Epub 2010 Jun 16.
Dupl 101PM
19
Perna G, Guerriero G, Caldirola D. Emerging drugs for panic disorder. Expert Opin Emerg Drugs. 2011;16(4):631-45. doi: 10.1517/14728214.2011.628313. Epub 2011 Oct 17.
Review
20
Ioachimescu OC, El-Solh AA. Pharmacotherapy of insomnia. Expert Opin Pharmacother. 2012;13(9):1243-60. doi: 10.1517/14656566.2012.683860. Epub 2012 May 11.
Dupl 111PM
21
Sullivan S. Update on emerging drugs for insomnia. Expert Opin Emerg Drugs. 2012;17(3):295-8. doi: 10.1517/14728214.2012.693158.
Opinion
22
Taslimi Z, Arezoomandan R, Omranifard A, Ghalandari-Shamami M, Riahi E, Vafaei AA, Rashidy-Pour A, Haghparast A. Orexin A in the ventral tegmental area induces conditioned place preference in a dose-dependent manner: involvement of D1/D2 receptors in the nucleus accumbens. Peptides. 2012;37(2):225-32. doi: 10.1016/j.peptides.2012.07.023. Epub 2012 Aug 3.
Dupl 117PM
23
Katwala J, Kumar AK, Sejpal JJ, Terrence M, Mishra M. Therapeutic rationale for low dose doxepin in insomnia patients. Asian Pac J Trop Dis. 2013;3(4):331-6. doi: 10.1016/S2222-1808(13)60080-8.
Review
24
Girault EM, Foppen E, Ackermans MT, Fliers E, Kalsbeek A. Central administration of an orexin receptor 1 antagonist prevents the stimulatory effect of Olanzapine on endogenous glucose production. Brain Res. 2013;1527:238-45. doi: 10.1016/j.brainres.2013.06.034. Epub 2013 Jul 4.
Dupl 122PM
25
LaCrosse AL, Olive MF. Neuropeptide systems and schizophrenia. CNS Neurol Disord Drug Targets. 2013;12(5):619-32. doi: 10.2174/1871527311312050010.
Dupl 119PM
26
Haghparast A, Omranifard A, Arezoomandan R, Ghalandari-Shamami M, Taslimi Z, Vafaei AA, Rashidy-Pour A. Involvement of dopaminergic receptors of the rat nucleus accumbens in decreasing the conditioned place preference induced by lateral hypothalamus stimulation. Neurosci Lett. 2013;556:10-4. doi: 10.1016/j.neulet.2013.09.062. Epub 2013 Oct 5.
Dupl 126PM
27
Palotai M, Telegdy G, Jászberényi M. Orexin A-induced anxiety-like behavior is mediated through GABA-ergic, α- and β-adrenergic neurotransmissions in mice. Peptides. 2014;57:129-34. doi: 10.1016/j.peptides.2014.05.003. Epub 2014 May 27.
Dupl 132PM
28
Chen YW, Morganstern I, Barson JR, Hoebel BG, Leibowitz SF. Differential role of D1 and D2 receptors in the perifornical lateral hypothalamus in controlling ethanol drinking and food intake: possible interaction with local orexin neurons. Alcohol Clin Exp Res. 2014;38(3):777-86. doi: 10.1111/acer.12313. Epub 2013 Nov 15.
Dupl 128PM
29
Palotai M, Telegdy G, Ekwerike A, Jászberényi M. The action of orexin B on passive avoidance learning. Involvement of neurotransmitters. Behav Brain Res. 2014;272:1-7. doi: 10.1016/j.bbr.2014.06.016. Epub 2014 Jun 13.

Dupl 133PM
30
Yazdi-Ravandi S, Razavi Y, Haghparast A, Goudarzvand M, Haghparast A. Orexin A induced antinociception in the ventral tegmental area involves D1 and D2 receptors in the nucleus accumbens. Pharmacol Biochem Behav. 2014;126:1-6. doi: 10.1016/j.pbb.2014.08.009. Epub 2014 Aug 30.
Dupl 142PM
31
Szabo ST, Kinon BJ, Brannan SK, Krystal AK, van Gerven JM, Mahableshwarkar A, Sachs GS. Lessons learned and potentials for improvement in CNS drug development: ISCTM Section on Designing the right series of experiments. Innov Clin Neurosci. 2015;12(3Suppl A):11S-25S.
Opinion
32
Uto Y. 1,2-Benzisoxazole compounds: a patent review (2009 - 2014). Expert Opin Ther Pat. 2015;25(6):643-62. doi: 10.1517/13543776.2015.1027192. Epub 2015 Mar 23.
Review
33
Shan L, Dauvilliers Y, Siegel JM. Interactions of the histamine and hypocretin systems in CNS disorders. Nat Rev Neurol. 2015;11(7):401-13. doi: 10.1038/nrneurol.2015.99. Epub 2015 Jun 23.
Review
34
Li A-J, Wang Q, Elsarelli MM, Brown RL, Ritter S. Hindbrain catecholamine neurons activate orexin neurons during systemic glucoprivation in male rats. Endocrinology. 2015;156(8):2807-20. doi: 10.1210/en.2015-1138. Epub 2015 May 15.
Dupl 141PM
35
Okumura T, Nozu T, Kumei S, Takakusaki K, Miyagishi S, Ohhira M. Involvement of the dopaminergic system in the central orexin-induced antinociceptive action against colonic distension in conscious rats. Neurosci Lett. 2015;605:34-8. doi: 10.1016/j.neulet.2015.08.013. Epub 2015 Aug 12.
Dupl 156PM
36
Vickers SP, Hackett D, Murray F, Hutson PH, Heal DJ. Effects of lisdexamfetamine in a rat model of binge-eating. J Psychopharmacol. 2015;29(12):1290-307. doi: 10.1177/0269881115615107. Epub 2015 Nov 20.
Dupl 156PM
37
Krystal AD. New developments in insomnia medications of relevance to mental health disorders. Psychiatr Clin North Am. 2015;38(4):843-60. doi: 10.1016/j.psc.2015.08.001. Epub 2015 Sep 11.
Dupl 158PM
38
Okumura T, Nozu T, Kumei S, Takakusaki K, Miyagishi S, Ohhira M. Levodopa acts centrally to induce an antinociceptive action against colonic distension through activation of D2 dopamine receptors and the orexinergic system in the brain in conscious rats. J Pharmacol Sci. 2016;130(2):123-7. doi: 10.1016/j.jphs.2016.01.007. Epub 2016 Jan 29.
Dupl 168PM
39
Clifton PG. Neural circuits of eating behaviour: Opportunities for therapeutic development. J Psychopharmacol. 2017;31(11):1388-1402. doi: 10.1177/0269881117738629. Epub 2017 Nov 14.
Review
40
Atkin T, Comai S, Gobbi G. Drugs for insomnia beyond benzodiazepines: Pharmacology, clinical applications, and discovery. Pharmacol Rev. 2018;70(2):197-245. doi: 10.1124/pr.117.014381.
Dupl 197PM
41
Okumura T, Nozu T, Kumei S, Takakusaki K, Ohhira M. Ghrelin acts centrally to induce an antinociceptive action during colonic distension through the orexinergic, dopaminergic and opioid systems in conscious rats. Brain Res. 2018;1686:48-54. doi: 10.1016/j.brainres.2018.02.024. Epub 2018 Feb 21.
Dupl 203PM
42
Heiss JE, Yamanaka A, Kilduff TS. Parallel arousal pathways in the lateral hypothalamus. eNeuro. 2018;5(4):ENEURO.0228-18.2018. doi: 10.1523/ENEURO.0228-18.2018.
Dupl 217PM
43
Liu C, Xue Y, Liu MF, Wang Y, Liu ZR, Diao HL, Chen L. Orexins increase the firing activity of nigral dopaminergic neurons and participate in motor control in rats. J Neurochem. 2018;147(3):380-394. doi: 10.1111/jnc.14568. Epub 2018 Oct 15.
Dupl 210PM
44
Linehan V, Rowe TM, Hirasawa M. Dopamine modulates excitatory transmission to orexin neurons in a receptor subtype-specific manner. Am J Physiol Regul Integr Comp Physiol. 2019;316(1):R68-R75. doi: 10.1152/ajpregu.00150.2018. Epub 2018 Nov 21.
Dupl 229PM
45
Okumura T, Nozu T, Kumei S, Ohhira M. Central oxytocin signaling mediates the central orexin-induced visceral antinociception through the opioid system in conscious rats. Physiol Behav. 2019;198:96-101. doi: 10.1016/j.physbeh.2018.10.007. Epub 2018 Oct 18.
Dupl 226PM
46
Hanazawa T, Kamijo Y. Effect of suvorexant on nocturnal delirium in elderly patients with Alzheimer's disease: A case-series study. Clin Psychopharmacol Neurosci. 2019;17(4):547-550. doi: 10.9758/cpn.2019.17.4.547.
Dupl 238PM
47
Dunn KE, Huhn AS, Bergeria CL, Gipson CD, Weerts EM. Non-opioid neurotransmitter systems that contribute to the opioid withdrawal syndrome: A review of preclinical and human evidence. J Pharmacol Exp Ther. 2019;371(2):422-452. doi: 10.1124/jpet.119.258004. Epub 2019 Aug 7.
Review
48
Magdaleno-Madrigal VM, Morales-Mulia S, Nicolini H, Genis-Mendoza A, Cázares-Martínez Claudia E, Pérez-Luna José M, Morales-Mulia M. Orexin-A promotes EEG changes but fails to induce anxiety in rats. Behav Brain Res. 2019;361:26-31. doi: 10.1016/j.bbr.2018.12.037. Epub 2018 Dec 21.

Dupl 232PM
49
Naghavi FS, Namvar P, Sadeghzadeh F, Haghparast A. The involvement of intra-hippocampal dopamine receptors in the conditioned place preference induced by orexin administration into the rat ventral tegmental area. Iran J Pharm Res. 2019;18(1):328-338.
Dupl 237PM
50
Lebedev AA, Bessolova YN, Efimov NS, Bychkov ER, Droblenkov AV, Shabanov PD. Role of orexin peptide system in emotional overeating induced by brain reward stimulation in fed rats. Res Results Pharmacol. 2020;6(1):81-91. doi: 10.3897/rrpharmacology.6.52180.
Animal
51
Matini T, Haghparast A, Rezaee L, Salehi S, Tehranchi A, Haghparast A. Role of dopaminergic receptors within the ventral tegmental area in antinociception induced by chemical stimulation of the lateral hypothalamus in an animal model of orofacial pain. J Pain Res. 2020;13:1449-1460. doi: 10.2147/JPR.S255250.
Dupl 262PM
52
Dujardin S, Pijpers A, Pevernagie D. Prescription drugs used in insomnia. Sleep Med Clin. 2020;15(2):133-145. doi: 10.1016/j.jsmc.2020.02.002.
Dupl 249PM
53
Lebedev AA, Bessolova YN, Efimov NS, Rusanovskii VV, Shabanov PD. Lateral hypothalamic self-stimulation with threshold current intensity induces emotional overeating in self-deprivation paradigm in well-fed rats: Role of orexin and dopaminergic systems of the brain. Reviews on Clinical Pharmacology and Drug Therapy, 19(4):421-429.
Animal
54
Pennington S, Stutzman D, Sannar E. Pitolisant in an adolescent with Prader-Willi syndrome. J Pediatr Pharmacol Ther. 2021;26(4):405-410. doi: 10.5863/1551-6776-26.4.405. Epub 2021 May 19.
Case
55
Borgland SL. Can treatment of obesity reduce depression or vice versa? J Psychiatry Neurosci. 2021;46(2):E313-E318. doi: 10.1503/jpn.210036.
Opinion
56
Joshi R, Bansal S, Malik D, Singla R, Mishra A, Prakash A, Medhi B. Computational modeling of ACE2 inhibitors for development of drugs against coronaviruses. In Roy K (Ed.) In Silico Modeling of Drugs Against Coronaviruses: Computational Tools and Protocols. Methods in Pharmacology and Toxicology, Springer Protocols. Totowa, New Jersey: Humana Press, 2021; pp. 615–629. doi: 10.1007/7653_2020_71. ISBN: 1071613677, 9781071613672.
Unrelated
57
Nigam M, Leu-Semenescu S, Arnulf I. Successful treatment of drug-resistant cataplexy with the anticholinergic drug tropatepine. J Clin Sleep Med. 2021;17(4):849-851. doi: 10.5664/jcsm.9030.
Dupl 263PM
58
Greenwald MK, Moses TEH, Roehrs TA. At the intersection of sleep deficiency and opioid use: mechanisms and therapeutic opportunities. Transl Res. 2021;234:58-73. doi: 10.1016/j.trsl.2021.03.006. Epub 2021 Mar 9.
Review
59
Nasrollahi S, Karimi S, Hamidi G, Naderitehrani M, Abed A. Blockade of the orexin 1 receptors in the nucleus accumbens' shell reversed the reduction effect of olanzapine on motivation for positive reinforcers. Neurosci Lett. 2021;762:136137. doi: 10.1016/j.neulet.2021.136137. Epub 2021 Jul 24.
Dupl 274PM
60
Hong J, Vernon D, Kunovac J, Stahl S. Emerging drugs for the treatment of major depressive disorder. Expert Opin Emerg Drugs. 2022;27(3):263-275. doi: 10.1080/14728214.2022.2117297. Epub 2022 Sep 20.
Dupl 305PM
61
Berger AA, Sottosanti ER, Winnick A, Keefe J, Gilbert E, Hasoon J, Thase ME, Kaye AD, Viswanath O, Urits I. Suvorexant in the treatment of difficulty falling and staying asleep (insomnia). Psychopharmacol Bull. 2022;52(1):68-90. doi: 10.64719/pb.4429.
Review
62
Koob GF. Anhedonia, hyperkatifeia, and negative reinforcement in substance use disorders. Curr Top Behav Neurosci. 2022;58:147-165. doi: 10.1007/7854_2021_288.
Review
63
Benca R, Herring WJ, Khandker R, Qureshi ZP. Burden of Insomnia and Sleep Disturbances and the Impact of Sleep Treatments in Patients with Probable or Possible Alzheimer's Disease: A Structured Literature Review. J Alzheimers Dis. 2022;86(1):83-109. doi: 10.3233/JAD-215324.
Review
64
Terada T, Hirayama T, Sadahiro R, Wada S, Nakahara R, Matsuoka H. Pilot study of lemborexant for insomnia in cancer patients with delirium. J Palliat Med. 2022;25(5):797-801. doi: 10.1089/jpm.2021.0509. Epub 2022 Jan 28.
Dupl 282PM
65
Heal DJ, Smith SL. Prospects for new drugs to treat binge-eating disorder: Insights from psychopathology and neuropharmacology. J Psychopharmacol. 2022;36(6):680-703. doi: 10.1177/02698811211032475. Epub 2021 Jul 28.
Review
66
Vasiliu O. Investigational drugs for the treatment of depression (Part 1): Monoaminergic, orexinergic, GABA-ergic, and anti-inflammatory agents. Front Pharmacol. 2022;13:884143. doi: 10.3389/fphar.2022.884143.
Dupl 297PM
67
Ishioh M, Nozu T, Miyagishi S, Igarashi S, Funayama T, Ohhira M, Okumura T. Activation of basal forebrain cholinergic neurons improves colonic hyperpermeability through the vagus nerve and adenosine A2B receptors in rats. Biochem Pharmacol. 2022;206:115331. doi: 10.1016/j.bcp.2022.115331. Epub 2022 Oct 29.

Animal
68
Serretti A. Fine-tuning of psychopharmacological treatments. Int Clin Psychopharmacol. 2023 Jan 1;38(1):1-3. doi: 10.1097/YIC.0000000000000447. Epub 2022 Nov 18.
Opinion
69
Rosenberg RP, Benca R, Doghramji P, Roth T. A 2023 Update on managing insomnia in primary care: Insights from an expert consensus group. Prim Care Companion CNS Disord. 2023;25(1):22nr03385. doi: 10.4088/PCC.22nr03385.
Review
70
Javed B, Javed A, Kow CS, Hasan SS. Pharmacological and non-pharmacological treatment options for sleep disturbances in Alzheimer's disease. Expert Rev Neurother. 2023;23(6):501-514. doi: 10.1080/14737175.2023.2214316. Epub 2023 Jun 2.
Review
71
Fagan HA, Baldwin DS. Pharmacological treatment of generalised anxiety disorder: Current practice and future directions. Expert Rev Neurother. 2023;23(6):535-548. doi: 10.1080/14737175.2023.2211767. Epub 2023 May 15.
Dupl 326PM
72
Hori H. Successful treatment of switching from benzodiazepine to orexin receptor antagonists improves cognitive function in psychiatric disorders: four case reports. Int Clin Psychopharmacol. 2023;38(3):192-194. doi: 10.1097/YIC.0000000000000450. Epub 2022 Dec 16.
Case
73
Pan B, Ge L, Lai H, Hou L, Tian C, Wang Q, Yang K, Lu Y, Zhu H, Li M, Wang D, Li X, Zhang Y, Gao Y, Liu M, Ding G, Tian J, Yang K. The comparative effectiveness and safety of insomnia drugs: A systematic review and network meta-analysis of 153 randomized trials. Drugs. 2023;83(7):587-619. doi: 10.1007/s40265-023-01859-8. Epub 2023 Mar 22.
Review
74
Dabrowska J. From recent advances in underlying neurocircuitry of fear and anxiety to promising pharmacotherapies for PTSD: The saga of heart, sex and the developing brain. Neuropharmacology. 2023;232:109529. doi: 10.1016/j.neuropharm.2023.109529. Epub 2023 Mar 31.
Opinion
75
Wu A. Updates and confounding factors in delayed sleep-wake phase disorder. Sleep Biol Rhythms. 2023;21(3):279-287. doi: 10.1007/s41105-023-00454-4.
Review
76
Nakamura T, Yoshizawa T, Toya R, Terasawa M, Takahashi K, Kitazawa K, Suzuki K, Sasayama D, Washizuka S. Orexin receptor antagonists versus antipsychotics for the management of delirium in intensive care unit patients with cardiovascular disease: A retrospective observational study. Gen Hosp Psychiatry. 2023;84:96-101. doi: 10.1016/j.genhosppsych.2023.06.019. Epub 2023 Jul 1.
Dupl 331PM
77
Bonifazi A, Del Bello F, Giorgioni G, Piergentili A, Saab E, Botticelli L, Cifani C, Micioni Di Bonaventura E, Micioni Di Bonaventura MV, Quaglia W. Targeting orexin receptors: Recent advances in the development of subtype selective or dual ligands for the treatment of neuropsychiatric disorders. Med Res Rev. 2023;43(5):1607-1667. doi: 10.1002/med.21959. Epub 2023 Apr 10.
Review
78
Arai Y, Sasayama D, Kuraishi A, Sahara R, Murata S, Tanaka A, Amemiya K, Usuda N, Kuraishi K, Washizuka S. Sodium valproate use in Japanese patients with schizophrenia and Coronavirus disease is associated with an increased risk of pneumonia. J Clin Med. 2023;12(18):5953. doi: 10.3390/jcm12185953.
Unfocused
79
Varadharajan A, Davis AD, Ghosh A, Jagtap T, Xavier A, Menon AJ, Roy D, Gandhi S, Gregor T. Guidelines for pharmacotherapy in Alzheimer's disease - A primer on FDA-approved drugs. J Neurosci Rural Pract. 2023;14(4):566-573. doi: 10.25259/JNRP_356_2023. Epub 2023 Oct 7.
Dupl 342PM
80
Klugherz LJ, Kolla BP, Mansukhani MP. Pharmacological treatments for insomnia. Curr Sleep Medicine Rep. 2023;9(4):265-273. doi: 10.1007/s40675-023-00265-6
Review
81
Watanabe K, Misaka S, Kanno-Nozaki K, Chiyoda T, Suzuki Y, Sato A, Suto T, Kuroda J, Shimomura K, Miura I, Yabe H. Effect of lemborexant on pharmacokinetics of clozapine: A potential drug-drug interaction mediated by time-dependent inhibition of CYP3A4. Br J Clin Pharmacol. 2024;90(1):354-359. doi: 10.1111/bcp.15889. Epub 2023 Sep 4.
Dupl 334PM
82
Aubin H-J. Repurposing drugs for treatment of alcohol use disorder. Int Rev Neurobiol. 2024;175:153-185. doi: 10.1016/bs.irn.2024.02.002. Epub 2024 Mar 12.
Dupl 354PM
83
Tachibana M, Kanahara N, Oda Y, Hasegawa T, Kimura A, Iyo M. A retrospective clinical practice study comparing the usefulness of dual-orexin receptor antagonists and a melatonin receptor agonist in patients switching from long-term benzodiazepine receptor agonists. J Clin Sleep Med. 2024;20(4):603-613. doi: 10.5664/jcsm.10946.
Unfocused
84
Kamata Y, Takashio O, Sato R, Kawai H, Ishii H, Aoyagi K, Tomita A, Toda S, Iwanami A. Relationship between insomnia and continued outpatient treatment in psychiatric patients. Neuropsychiatr Dis Treat. 2024;20:697-723. doi: 10.2147/NDT.S454757.
Unfocused
85
Mori Y, Watanabe K, Suzuki Y, Ono H, Tojo M, Kawasaki Y, Kanno-Nozaki K, Nozaki M, Miura I. Clozapine-associated myocarditis in a patient with schizophrenia taking lemborexant: A case report. J Clin Psychopharmacol. 2024;44(2):193-195. doi: 10.1097/JCP.0000000000001817. Epub 2024 Feb 7.
Dupl 349PM

86

Devlin JW. Pharmacologic treatment strategies for delirium in hospitalized adults: Past, present, and future. Semin Neurol. 2024;44(6):762-776. doi: 10.1055/s-0044-1791246. Epub 2024 Sep 23.

Review

87

Liaskopoulos A, Kakouris V, Liaskopoulos N, Lappas AS, Christodoulou N, Samara M. Medical professionals and pharmacological intervention for the treatment of insomnia: A cross-sectional study. Sleep Sci. 2024;18(2):e155-e164. doi: 10.1055/s-0044-1791238.

Dupl 396PM

88

Kikuchi Y, Kurosawa M, Sakata M, Takahashi Y, Yamamoto K, Tomita H, Yoshio T, Yasui-Furukori N. Effects of titration speed, gender, obesity and concomitant medications on the risk and onset time of clozapine-associated fever among Japanese patients with schizophrenia: retrospective review of charts from 21 hospitals. Br J Psychiatry. 2024;225(5):492-498. doi: 10.1192/bjp.2024.113.

Dupl 368PM

89

Nozu T, Miyagishi S, Ishioh M, Takakusaki K, Okumura T. The neurotensin receptor 1 agonist PD149163 alleviates visceral hypersensitivity and colonic hyperpermeability in rat irritable bowel syndrome model. Neurogastroenterol Motil. 2024;36(12):e14925. doi: 10.1111/nmo.14925. Epub 2024 Sep 24.

Dupl 383PM

90

Nakamura T, Furihata R, Hasegawa N, Kodaka F, Muraoka H, Ichihashi K, Ochi S, Numata S, Tsuboi T, Makinodan M, Iida H, Onitsuka T, Kashiwagi H, Takeshima M, Hashimoto N, Nagasawa T, Usami M, Yamagata H, Takaesu Y, Miura K, Matsumoto J, Ohi K, Yamada H, Hori H, Inada K, Watanabe K, Hashimoto R, Yasui-Furukori N. The effect of education regarding treatment guidelines for schizophrenia and major depressive disorders on psychiatrists' hypnotic medication prescribing behavior: a multicenter study. BMC Psychiatry. 2024;24(1):399. doi: 10.1186/s12888-024-05816-x.

Dupl 361PM

91

Zhang M, Chen T, Lu X, Lan X, Chen Z, Lu S. G protein-coupled receptors (GPCRs): advances in structures, mechanisms, and drug discovery. Signal Transduct Target Ther. 2024;9(1):88. doi: 10.1038/s41392-024-01803-6.

Review

92

Sogawa R, Hatano M, Nishimura F, Nishi J, Matsuoka A, Shinada K, Yamada H, Tateishi H, Mizoguchi Y, Monji A, Shimanoe C. Association between hypnotics, accidents, and injuries: A study based on the Adverse Drug Event Reporting Database in Japan. In Vivo. 2025;39(1):433-439. doi: 10.21873/invivo.13846.

Unfocused

93

Ngomana C, Komape KD, Bronkhorst E. A review on holistic and pharmacological management of insomnia. SA Pharm J. 2025;92(1):11-16. doi: 10.36303/SAPJ.2283.

Review

94

Chopra A, Rustad JK, Hall DL, Mak MSB, Stern TA. Management of Insomnia in the General Hospital. Prim Care Companion CNS Disord. 2025;27(1):24f03793. doi: 10.4088/PCC.24f03793.

Case

95

Nozu T, Miyagishi S, Ishioh M, Takakusaki K, Okumura T. Irisin prevents visceral hypersensitivity and colonic hyperpermeability in a rat model of irritable bowel syndrome. Peptides. 2025;188:171394. doi: 10.1016/j.peptides.2025.171394. Epub 2025 Mar 26.

Dupl 398PM

96

Mori K, Ohashi K, Kimura M, Yoshida M, Tomida K, Usami E (森 光輝，大橋健吾，木村美智男，吉田光代，富田顕旨，宇佐美英績). オレキシン受容体拮抗薬推進活動が睡眠薬・抗精神病薬の処方動向に与える影響：分割時系列解析研究 [Impact of promotional activities on orexin receptor antagonists prescription rates and usage of sleep and antipsychotic medications: An interrupted time-series analysis study]. Yakugaku Zasshi. 2025;145(7):629-637. Japanese. doi: 10.1248/yakushi.24-00170.

Dupl 394PM

97

Tanaka A, Arai Y, Yasaki T, Saito K, Nakashizuka E, Yoshida S, Nakajima Y, Koido M, Suzuki K, Nakamura T, Sasayama D, Washizuka S. Longitudinal associations of serum orexin-A with physical activity and sleep in schizophrenia: A preliminary study. Neuropsychopharmacol Rep. 2025;45(4):e70079. doi: 10.1002/npr2.70079.

Dupl PM415

98

IsHak WW, Hirsch D, Renteria S, Totlani J, Murphy N, Chang T, Abdelsalam R, Salem M, Meyer A, Keerthana S, Liu A, Contreras L, Tadros E, Hedrick R, Danovitch I, Pechnick RN. Depressive disorders: systematic review of approved psychiatric medications (2009-April 2025) and pipeline phase 3 medications. BMC Psychiatry. 2025;25(1):939. doi: 10.1186/s12888-025-07141-3.

Review

99

Fuglsang NFB, Madsen NM, Jacobsen SL, Frøkjær JE, Ladegaard N, Sørensen MA, Correll CU, Otte C, Højlund M, Köhler-Forsberg O. Efficacy and Acceptability of Licensed and Off-Label Pharmacological Interventions for Insomnia in Patients With Severe Mental Illness: A Systematic Review and Meta-Analysis of Randomised Trials. Acta Psychiatr Scand. 2025;152(6):405-421. doi: 10.1111/acps.70032. Epub 2025 Sep 2.

Review

(schizophr* OR psychosis OR psychotic OR paranoi* OR schizoaffect* OR antipsychotic* OR neuroleptic* OR phenothiazine* OR butyrophenon* OR (dopamine AND antagonist*) OR (dopamine AND receptor AND antagonist*) OR (substituted AND benzamide*) OR thioxanthene* OR dibenzoazepine* OR benzisoxazole* OR haloperidol OR chlorpromazine OR promazine OR thioridazine OR clothiapine OR loxapine OR clozapine OR quetiapine OR olanzapine OR fluperlapin* OR zotepine OR remoxipride OR sertindole OR risperidone OR paliperidone OR aripiprazole OR brexpiprazole OR cariprazine OR ziprasidone OR asenapine OR bromperidol OR spiroperidol OR spiperone OR perphenazine OR fluphenazine OR flupentixol* OR clopenthixol OR zuclopenthixol OR droperidol OR thioxanthene OR sulpiride OR sultopride OR metoclopramide OR amisulpride) AND (orexin* OR hypocretin* OR Hcrts OR OX1 OR OX2 OR OX1R OR OX2R OR suvorexant OR MK-4305 OR lemborexant OR daridorexant OR almorexant OR ACT-078573 OR filorexant OR MK-6096 OR seltorexant OR MIN-202 OR JNJ-42847922 OR JNJ-922 OR Fazamorexant OR YZJ-1139 OR Nivasorexant OR ACT-539313 OR Tebideutorexant OR JNJ-61393215 OR JNJ-3215 OR Vornorexant OR ORN-0829 OR TS-142 OR ACT-335827 OR EMPA OR GSK-649868 OR SB-649868 OR JNJ-10397049 OR RTIOX-276 OR SB-334867 OR SB-408124 OR TCS-OX2-29) PsycINFO/PsycARTICLES 3.3.2026 → 167 results

1

Garfield SL. An angry and critical appraisal of professional psychology. Clin Psychol Sci Pract. 1996;3(3):268-272.

Opinion

2

Nakamura T, Uramura K, Nambu T, Yada T, Goto K, Yanagisawa M, Sakurai T. Orexin-induced hyperlocomotion and stereotypy are mediated by the dopaminergic system. Brain Res. 2000;873(1):181-7. doi: 10.1016/s0006-8993(00)02555-5.
Dupl 12PM
3
Nishino S, Ripley B, Mignot E, Benson KL, Zarcone VP. CSF hypocretin-1 levels in schizophrenics and controls: relationship to sleep architecture. Psychiatry Res. 2002;110(1):1-7. doi: 10.1016/s0165-1781(02)00032-x.
Dupl 30PM
4
Fadel J, Bubser M, Deutch AY. Differential activation of orexin neurons by antipsychotic drugs associated with weight gain. J Neurosci. 2002;22(15):6742-6. doi: 10.1523/JNEUROSCI.22-15-06742.2002.
Dupl 31PM
5
Katz JD, Ropper AH. Familial Kleine-Levin syndrome: two siblings with unusually long hypersomnic spells. Arch Neurol. 2002;59(12):1959-61. doi: 10.1001/archneur.59.12.1959.
Dupl 32PM
6
Đukić Dejanović SM, Janjić V, Milovanović D. Etiopatogeneza nesanice [Aetiopathogenesis of insomnia]. Psihijatrija Danas 2003;35(1):5-21.
Review
7
Monda M, Viggiano A, De Luca V. Haloperidol reduces the sympathetic and thermogenic activation induced by orexin A. Neurosci Res. 2003;45(1):17-23. doi: 10.1016/s0168-0102(02)00191-8.
Dupl 33PM 2S
8
Midei A, Licinio J. General summary. Mol Psychiatry. 2003;8(10):833-834. doi: 10.1038/sj.mp.4001416.
Opinion
9
Alkemade A, Unmehopa UA, Brouwer JP, Hoogendijk WJ, Wiersinga WM, Swaab DF, Fliers E. Decreased thyrotropin-releasing hormone gene expression in the hypothalamic paraventricular nucleus of patients with major depression. Mol Psychiatry. 2003;8(10):838-9. doi: 10.1038/sj.mp.4001364.
Unrelated
10
Douglass AB. Narcolepsy: differential diagnosis or etiology in some cases of bipolar disorder and schizophrenia? CNS Spectr. 2003;8(2):120-6. doi: 10.1017/s1092852900018344.
Dupl 35PM
11
Young WB, Piovesan EJ, Biglan KM. Restless legs syndrome and drug-induced akathisia in headache patients. CNS Spectr. 2003;8(6):450-6. doi: 10.1017/s1092852900018769.
Dupl 36PM
12
Sheitman BB, Knable MB, Jarskog LF, Chakos M, Boyce LH, Early J, Lieberman JA. Secretin for refractory schizophrenia. Schizophr Res. 2004;66(2-3):177-81. doi: 10.1016/S0920-9964(03)00068-9.
Dupl 43PM
13
Figlewicz DP, Bennett J, Evans SB, Kaiyala K, Sipols AJ, Benoit SC. Intraventricular insulin and leptin reverse place preference conditioned with high-fat diet in rats. Behav Neurosci. 2004;118(3):479-87. doi: 10.1037/0735-7044.118.3.479.
Animal
14
Annual Meeting of the Biological Psychologists and Neuropsychologists of the German Society of Psychology (DGPs) as well as the German Society of Psychophysiology and its Applications (DGPA) - 31st APM: Psychology and Brain, Bochum, Germany, May 25-27, 2005. J Psychophysiol. 2005;19(2):106-156.
Abstr
15
Pecoraro N, Dallman MF. c-Fos after incentive shifts: expectancy, incredulity, and recovery. Behav Neurosci. 2005;119(2):366-87. doi: 10.1037/0735-7044.119.2.366.
Animal
16
Walterfang M, Upjohn E, Velakoulis D. Is schizophrenia associated with narcolepsy? Cogn Behav Neurol. 2005;18(2):113-8. doi: 10.1097/01.wnn.0000160822.53577.2c.
Dupl 53PM
17
Meerabux J, Iwayama Y, Sakurai T, Ohba H, Toyota T, Yamada K, Nagata R, Irukayama-Tomobe Y, Shimizu H, Yoshitsugu K, Ohta K, Yoshikawa T. Association of an orexin 1 receptor 408Val variant with polydipsia-hyponatremia in schizophrenic subjects. Biol Psychiatry. 2005;58(5):401-7. doi: 10.1016/j.biopsych.2005.04.015.
Dupl 55PM
18
Baldo BA, Alsene KM, Negron A, Kelley AE. Hyperphagia induced by GABAA receptor-mediated inhibition of the nucleus accumbens shell: dependence on intact neural output from the central amygdaloid region. Behav Neurosci. 2005;119(5):1195-206. doi: 10.1037/0735-7044.119.5.1195.
Animal
19

Barbano MF, Cador M. Various aspects of feeding behavior can be partially dissociated in the rat by the incentive properties of food and the physiological state. Behav Neurosci. 2005;119(5):1244-53. doi: 10.1037/0735-7044.119.5.1244.
Animal
20
Cochen V, Arnulf I, Demeret S, Neulat ML, Gourlet V, Drouot X, Moutereau S, Derenne JP, Similowski T, Willer JC, Pierrot-Deseilligny C, Bolgert F. Vivid dreams, hallucinations, psychosis and REM sleep in Guillain-Barré syndrome. Brain. 2005;128(Pt 11):2535-45. doi: 10.1093/brain/awh585. Epub 2005 Jul 6.
Dupl 56PM
21
Narita M, Nagumo Y, Hashimoto S, Narita M, Khotib J, Miyatake M, Sakurai T, Yanagisawa M, Nakamachi T, Shioda S, Suzuki T. Direct involvement of orexinergic systems in the activation of the mesolimbic dopamine pathway and related behaviors induced by morphine. J Neurosci. 2006;26(2):398-405. doi: 10.1523/JNEUROSCI.2761-05.2006.
Dupl 59PM 8S
22
Alberto CO, Trask RB, Quinlan ME, Hirasawa M. Bidirectional dopaminergic modulation of excitatory synaptic transmission in orexin neurons. J Neurosci. 2006;26(39):10043-50. doi: 10.1523/JNEUROSCI.1819-06.2006. Retraction in: J Neurosci. 2012;32(26):9116. doi: 10.1523/JNEUROSCI.1889-12.2012.
Dupl 65PM 11Sc
23
Monda M, Viggiano A, Viggiano A, Viggiano E, Messina G, Tafuri D, De Luca V. Quetiapine lowers sympathetic and hyperthermic reactions due to cerebral injection of orexin A. Neuropeptides. 2006;40(5):357-63. doi: 10.1016/j.npep.2006.07.003. Epub 2006 Sep 28.
Dupl 66PM
24
De Clercq B, De Fruyt F, Van Leeuwen K, Mervielde I. The structure of maladaptive personality traits in childhood: a step toward an integrative developmental perspective for DSM-V. J Abnorm Psychol. 2006;115(4):639-57. doi: 10.1037/0021-843X.115.4.639.
Unrelated
25
Rasmussen K, Hsu MA, Yang Y. The orexin-1 receptor antagonist SB-334867 blocks the effects of antipsychotics on the activity of A9 and A10 dopamine neurons: implications for antipsychotic therapy. Neuropsychopharmacology. 2007;32(4):786-92. doi: 10.1038/sj.npp.1301239. Epub 2006 Oct 25.
Dupl 68PM 11S
26
Brundin L, Petersén A, Björkqvist M, Träskman-Bendz L. Orexin and psychiatric symptoms in suicide attempters. J Affect Disord. 2007;100(1-3):259-63. doi: 10.1016/j.jad.2006.10.019. Epub 2006 Dec 4.
Dupl 71PM
27
Brundin L, Björkqvist M, Petersén A, Träskman-Bendz L. Reduced orexin levels in the cerebrospinal fluid of suicidal patients with major depressive disorder. Eur Neuropsychopharmacol. 2007;17(9):573-9. doi: 10.1016/j.euroneuro.2007.01.005. Epub 2007 Mar 7.
Dupl 72PM
28
Lambe EK, Liu RJ, Aghajanian GK. Schizophrenia, hypocretin (orexin), and the thalamocortical activating system. Schizophr Bull. 2007;33(6):1284-90. doi: 10.1093/schbul/sbm088. Epub 2007 Jul 26.
Dupl 76PM
29
Rasmussen K, Hsu MA, Noone S, Johnson BG, Thompson LK, Hemrick-Luecke SK. The orexin-1 antagonist SB-334867 blocks antipsychotic treatment emergent catalepsy: implications for the treatment of extrapyramidal symptoms. Schizophr Bull. 2007;33(6):1291-7. doi: 10.1093/schbul/sbm087. Epub 2007 Jul 28.
Dupl 77PM 12S
30
Morein-Zamir S, Turner DC, Sahakian BJ. A review of the effects of modafinil on cognition in schizophrenia. Schizophr Bull. 2007;33(6):1298-306. doi: 10.1093/schbul/sbm090. Epub 2007 Jul 18.
Dupl 75PM
31
Janas-Kozik M, Stachowicz M, Mazurek U, Zajdel A, Wilczok A, Krupka-Matuszczyk I, Rybakowski JK. Preliminary study of the expression of genes connected with the orexigenic and anorexigenic system using microarray technique in anorexia nervosa. Neuropsychobiology. 2008;57(3):116-20. doi: 10.1159/000138913. Epub 2008 Jun 13.
Dupl 88PM
32
Minzenberg MJ, Carter CS. Modafinil: a review of neurochemical actions and effects on cognition. Neuropsychopharmacology. 2008;33(7):1477-502. doi: 10.1038/sj.npp.1301534. Epub 2007 Aug 22.
Dupl 78PM
33
Qu WM, Huang ZL, Xu XH, Matsumoto N, Urade Y. Dopaminergic D1 and D2 receptors are essential for the arousal effect of modafinil. J Neurosci. 2008;28(34):8462-9. doi: 10.1523/JNEUROSCI.1819-08.2008.
Dupl 90PM
34
Wallingford NM, Sinnayah P, Bymaster FP, Gadde KM, Krishnan RK, McKinney AA, Landbloom RP, Tollefson GD, Cowley MA. Zonisamide prevents olanzapine-associated hyperphagia, weight gain, and elevated blood glucose in rats. Neuropsychopharmacology. 2008;33(12):2922-33. doi: 10.1038/npp.2008.9. Epub 2008 Mar 5.
Dupl 84PM
35

Stefanidis A, Verty AN, Allen AM, Owens NC, Cowley MA, Oldfield BJ. The role of thermogenesis in antipsychotic drug-induced weight gain. Obesity (Silver Spring). 2009;17(1):16-24. doi: 10.1038/oby.2008.468. Epub 2008 Oct 30.
Dupl 93PM
36
Cope MB, Li X, Jumbo-Lucioni P, DiCostanzo CA, Jamison WG, Kesterson RA, Allison DB, Nagy TR. Risperidone alters food intake, core body temperature, and locomotor activity in mice. Physiol Behav. 2009;96(3):457-63. doi: 10.1016/j.physbeh.2008.11.011. Epub 2008 Nov 27.
Dupl 92PM
37
Brockington IF. Monthly psychosis starting before the menarche. Arch Womens Ment Health. 2009;12(2):121-2. doi: 10.1007/s00737-009-0053-x. Epub 2009 Mar 27.
Opinion
38
Davoodi N, Kalinichev M, Korneev SA, Clifton PG. Hyperphagia and increased meal size are responsible for weight gain in rats treated sub-chronically with olanzapine. Psychopharmacology (Berl). 2009;203(4):693-702. doi: 10.1007/s00213-008-1415-1. Epub 2008 Dec 4.
Dupl 91PM
39
Gritton HJ, Sutton BC, Martinez V, Sarter M, Lee TM. Interactions between cognition and circadian rhythms: attentional demands modify circadian entrainment. Behav Neurosci. 2009;123(5):937-48. doi: 10.1037/a0017128.
Animal
40
Panossian LA, Avidan AY. Narcolepsy and other comorbid medical illnesses. In: Goswami M, Pandi-Perumal S, Thorpy M (eds) Narcolepsy. New York, NY: Springer, 2010; pp. 105-113. doi: 10.1007/978-1-4419-0854-4_10.
Review
41
Schenberg LC. Towards a translational model of panic attack. Psychol Neurosci. 2010;3(1):9-37. doi: 10.3922/j.psns.2010.1.003.
Review
42
Winrow CJ, Tanis KQ, Reiss DR, Rigby AM, Uslaner JM, Uebele VN, Doran SM, Fox SV, Garson SL, Gotter AL, Levine DM, Roecker AJ, Coleman PJ, Koblan KS, Renger JJ. Orexin receptor antagonism prevents transcriptional and behavioral plasticity resulting from stimulant exposure. Neuropharmacology. 2010;58(1):185-94. doi: 10.1016/j.neuropharm.2009.07.008. Epub 2009 Jul 14.
Animal
43
Borgland SL, Labouèbe G. Orexin/hypocretin in psychiatric disorders: present state of knowledge and future potential. Neuropsychopharmacology. 2010;35(1):353-4. doi: 10.1038/npp.2009.119.
Dupl 98PM
44
Basoglu C, Oner O, Gunes C, Semiz UB, Ates AM, Algul A, Ebrinc S, Cetin M, Ozcan O, Ipcioglu O. Plasma orexin A, ghrelin, cholecystokinin, visfatin, leptin and agouti-related protein levels during 6-week olanzapine treatment in first-episode male patients with psychosis. Int Clin Psychopharmacol. 2010;25(3):165-71. doi: 10.1097/YIC.0b013e3283377850.
Dupl 100PM
45
Milella MS, Passarelli F, De Carolis L, Schepisi C, Nativio P, Scaccianoce S, Nencini P. Opposite roles of dopamine and orexin in quinpirole-induced excessive drinking: a rat model of psychotic polydipsia. Psychopharmacology (Berl). 2010;211(3):355-66. doi: 10.1007/s00213-010-1909-5. Epub 2010 Jun 16.
Dupl 101PM 17S
46
Burgess CR, Tse G, Gillis L, Peever JH. Dopaminergic regulation of sleep and cataplexy in a murine model of narcolepsy. Sleep. 2010;33(10):1295-304. doi: 10.1093/sleep/33.10.1295.
Animal
47
Sanislow CA, Pine DS, Quinn KJ, Kozak MJ, Garvey MA, Heinssen RK, Wang PS, Cuthbert BN. Developing constructs for psychopathology research: research domain criteria. J Abnorm Psychol. 2010;119(4):631-9. doi: 10.1037/a0020909.
Opinion
48
Hester R, Lee N, Pennay A, Nielsen S, Ferris J. The effects of modafinil treatment on neuropsychological and attentional bias performance during 7-day inpatient withdrawal from methamphetamine dependence. Exp Clin Psychopharmacol. 2010;18(6):489-97. doi: 10.1037/a0021791.
Unfocused
49
Panariello F, Javaid N, Teo C, Monda M, Viggiano A, De Luca V. The role of orexin system in antipsychotics induced weight gain. Curr Psychiatry Rev. 2011;7(1):12-18. doi: 10.2174/157340011795945793.
Review
50
Fernø J, Varela L, Skrede S, Vázquez MJ, Nogueiras R, Diéguez C, Vidal-Puig A, Steen VM, López M. Olanzapine-induced hyperphagia and weight gain associate with orexigenic hypothalamic neuropeptide signaling without concomitant AMPK phosphorylation. PLoS One. 2011;6(6):e20571. doi: 10.1371/journal.pone.0020571. Epub 2011 Jun 13.
Dupl 107PM
51
Wirtshafter D, Davis JD, Stratford TR. Inactivation of the median raphe nucleus increases intake of sucrose solutions: a microstructural analysis. Behav Neurosci. 2011;125(4):529-40. doi: 10.1037/a0024372.

Animal
52
de Lecea L, Carter ME, Adamantidis A. Shining light on wakefulness and arousal. Biol Psychiatry. 2012;71(12):1046-52. doi: 10.1016/j.biopsych.2012.01.032. Epub 2012 Mar 20.
Review
53
Bradshaw CM, Killeen PR. A theory of behaviour on progressive ratio schedules, with applications in behavioural pharmacology. Psychopharmacology (Berl). 2012;222(4):549-64. doi: 10.1007/s00213-012-2771-4. Epub 2012 Jul 3.
Dupl 114PM
54
Lehner T. The genes in the major histocompatibility complex as risk factors for schizophrenia: de omnibus dubitandum. Biol Psychiatry. 2012;72(8):615-6. doi: 10.1016/j.biopsych.2012.08.002.
Opinion
55
Girault EM, Foppen E, Ackermans MT, Fliers E, Kalsbeek A. Central administration of an orexin receptor 1 antagonist prevents the stimulatory effect of Olanzapine on endogenous glucose production. Brain Res. 2013;1527:238-45. doi: 10.1016/j.brainres.2013.06.034. Epub 2013 Jul 4.
Dupl 122PM 22S
56
Gozzi A, Lepore S, Vicentini E, Merlo-Pich E, Bifone A. Differential effect of orexin-1 and CRF-1 antagonism on stress circuits: a fMRI study in the rat with the pharmacological stressor Yohimbine. Neuropsychopharmacology. 2013;38(11):2120-30. doi: 10.1038/npp.2013.109. Epub 2013 May 8.
Dupl 120PM
57
Abstracts of the annual CSBBCS meeting: 2013 résumés du congrès annuel 2013 de la SCCCSC. Canadian Journal of Experimental Psychology / Revue canadienne de psychologie expérimentale, 2013, 67, 4, 271-312 (Prenatal Activation of Orexinergic Neurons, Maria Pompeiano, Ahn Lee, Kyle E. Godden).
Abstr
58
Huang YS, Guilleminault C, Chen CH, Lai PC, Hwang FM. Narcolepsy-cataplexy and schizophrenia in adolescents. Sleep Med. 2014;15(1):15-22. doi: 10.1016/j.sleep.2013.09.018. Epub 2013 Oct 26.
Dupl 124PM
59
Bonfils KA, Adams EL, Firmin RL, White LM, Salyers MP. Parenthood and severe mental illness: relationships with recovery. Psychiatr Rehabil J. 2014;37(3):186-93. doi: 10.1037/prj0000072. Epub 2014 May 12.
Unrelated
60
Palotai M, Telegdy G, Ekwerike A, Jászberényi M. The action of orexin B on passive avoidance learning. Involvement of neurotransmitters. Behav Brain Res. 2014;272:1-7. doi: 10.1016/j.bbr.2014.06.016. Epub 2014 Jun 13.
Dupl 133PM 25S
61
Inutsuka A, Inui A, Tabuchi S, Tsunematsu T, Lazarus M, Yamanaka A. Concurrent and robust regulation of feeding behaviors and metabolism by orexin neurons. Neuropharmacology. 2014;85:451-60. doi: 10.1016/j.neuropharm.2014.06.015. Epub 2014 Jun 18.
Dupl 134PM
62
Messina G, Viggiano A, Chieffi S, Viggiano E, Tafuri D, De Luca V, Messina A, Monda M. Neuroleptic drugs affect sympathetic and thermogenic reactions to Orexin A. Afr J Psychiatry. 2014;17(6):1000175. doi:10.4172/Psychiatry.1000172
Review
63
Risco S, Mediavilla C. Orexin-1 receptor antagonist in central nucleus of the amygdala attenuates the acquisition of flavor-taste preference in rats. Pharmacol Biochem Behav. 2014;126:7-12. doi: 10.1016/j.pbb.2014.09.002. Epub 2014 Sep 16.
Animal
64
Kolaj M, Zhang L, Renaud LP. Novel coupling between TRPC-like and KNa channels modulates low threshold spike-induced afterpotentials in rat thalamic midline neurons. Neuropharmacology. 2014;86:88-96. doi: 10.1016/j.neuropharm.2014.06.023. Epub 2014 Jul 9.
Dupl 135PM
65
Chien YL, Liu CM, Shan JC, Lee HJ, Hsieh MH, Hwu HG, Chiou LC. Elevated plasma orexin A levels in a subgroup of patients with schizophrenia associated with fewer negative and disorganized symptoms. Psychoneuroendocrinology. 2015;53:1-9. doi: 10.1016/j.psyneuen.2014.12.012. Epub 2014 Dec 20.
Dupl 137PM
66
Bailey MR, Jensen G, Taylor K, Mezias C, Williamson C, Silver R, Simpson EH, Balsam PD. A novel strategy for dissecting goal-directed action and arousal components of motivated behavior with a progressive hold-down task. Behav Neurosci. 2015;129(3):269-80. doi: 10.1037/bne0000060.
Animal
67
Rojczyk E, Pałasz A, Wiaderkiewicz R. Effect of short and long-term treatment with antipsychotics on orexigenic/anorexigenic neuropeptides expression in the rat hypothalamus. Neuropeptides. 2015;51:31-42. doi: 10.1016/j.npep.2015.04.001. Epub 2015 Apr 3.
Dupl 138PM
68
Nishizawa D, Kasai S, Hasegawa J, Sato N, Yamada H, Tanioka F, Nagashima M, Katoh R, Satoh Y, Tagami M, Ujike H, Ozaki N, Inada T, Iwata N, Sora I, Iyo M, Yamada M, Kondo N, Won MJ, Naruse N, Uehara-Aoyama K, Itokawa M, Ohi K, Hashimoto R, Tanisawa K, Arai T, Mori S, Sawabe M, Naka-Mieno

M, Yamada Y, Yamada M, Sato N, Muramatsu M, Tanaka M, Irukayama-Tomobe Y, Saito YC, Sakurai T, Hayashida M, Sugimura H, Ikeda K. Associations between the orexin (hypocretin) receptor 2 gene polymorphism Val308Ile and nicotine dependence in genome-wide and subsequent association studies. Mol Brain. 2015;8:50. doi: 10.1186/s13041-015-0142-x.
Dupl 151PM
69
Moradi M, Yazdanian M, Haghparast A. Role of dopamine D2-like receptors within the ventral tegmental area and nucleus accumbens in antinociception induced by lateral hypothalamus stimulation. Behav Brain Res. 2015;292:508-14. doi: 10.1016/j.bbr.2015.07.007. Epub 2015 Jul 9.
Dupl 147PM
70
Parker KE, McCabe MP, Johns HW, Lund DK, Odu F, Sharma R, Thakkar MM, Cornelison DD, Will MJ. Neural activation patterns underlying basolateral amygdala influence on intra-accumbens opioid-driven consummatory versus appetitive high-fat feeding behaviors in the rat. Behav Neurosci. 2015;129(6):812-21. doi: 10.1037/bne0000095. Epub 2015 Oct 26.
Animal
71
Krystal AD. New developments in insomnia medications of relevance to mental health disorders. Psychiatr Clin North Am. 2015;38(4):843-60. doi: 10.1016/j.psc.2015.08.001. Epub 2015 Sep 11.
Dupl 158PM 31S
72
Vickers SP, Hackett D, Murray F, Hutson PH, Heal DJ. Effects of lisdexamfetamine in a rat model of binge-eating. J Psychopharmacol. 2015;29(12):1290-307. doi: 10.1177/0269881115615107. Epub 2015 Nov 20.
Dupl 156PM 30S
73
Oepen G, Oepen I. Possible motor benefits of modafinil in Parkinsonian antecollis: A case report. Clin Neuropsychiatry. 2016;13(1-2):7-9.
Case
74
Sun HQ, Li SX, Chen FB, Zhang Y, Li P, Jin M, Sun Y, Wang F, Mi WF, Shi L, Yue JL, Yang FD, Lu L. Diurnal neurobiological alterations after exposure to clozapine in first-episode schizophrenia patients. Psychoneuroendocrinology. 2016;64:108-16. doi: 10.1016/j.psyneuen.2015.11.013. Epub 2015 Nov 26.
Dupl 160PM
75
Tiwari AK, Brandl EJ, Zai CC, Goncalves VF, Chowdhury NI, Freeman N, Lieberman JA, Meltzer HY, Kennedy JL, Müller DJ. Association of orexin receptor polymorphisms with antipsychotic-induced weight gain. World J Biol Psychiatry. 2016;17(3):221-9. doi: 10.3109/15622975.2015.1076173. Epub 2015 Oct 8.
Dupl 154PM
76
Altena E, Micoulaud-Franchi JA, Geoffroy PA, Sanz-Arigita E, Bioulac S, Philip P. The bidirectional relation between emotional reactivity and sleep: From disruption to recovery. Behav Neurosci. 2016;130(3):336-50. doi: 10.1037/bne0000128. Epub 2016 Feb 11.
Review
77
Wagner L, Kaestner F, Wolf R, Stiller H, Heiser U, Manhart S, Hoffmann T, Rahfeld JU, Demuth HU, Rothermundt M, von Hörsten S. Identifying neuropeptide Y (NPY) as the main stress-related substrate of dipeptidyl peptidase 4 (DPP4) in blood circulation. Neuropeptides. 2016;57:21-34. doi: 10.1016/j.npep.2016.02.007. Epub 2016 Feb 27.
Dupl 165PM
78
Kelly JF. Proceedings of the American Psychological Association, Incorporated, for the legislative year 2015: Minutes of the Annual Meeting of the Council of Representatives February 20-22, 2015, Washington, DC, and August 5 and August 7, 2015, Washington, DC, and minutes of the February, June, August, and December 2015 meetings of the Board of Directors. Am Psychol. 2016;71(5):369-414. doi: 10.1037/amp0000022.
Abstr
79
Luck C, Vitaterna MH, Wevrick R. Dopamine pathway imbalance in mice lacking Magel2, a Prader-Willi syndrome candidate gene. Behav Neurosci. 2016;130(4):448-59. doi: 10.1037/bne0000150. Epub 2016 Jun 2.
Animal
80
Xue Y, Yang YT, Liu HY, Chen WF, Chen AQ, Sheng Q, Chen XY, Wang Y, Chen H, Liu HX, Pang YY, Chen L. Orexin-A increases the activity of globus pallidus neurons in both normal and parkinsonian rats. Eur J Neurosci. 2016;44(5):2247-57. doi: 10.1111/ejn.13323. Epub 2016 Jul 13.
Dupl 170PM
81
McHenry JA, Robison CL, Bell GA, Vialou VV, Bolaños-Guzmán CA, Nestler EJ, Hull EM. The role of ΔfosB in the medial preoptic area: Differential effects of mating and cocaine history. Behav Neurosci. 2016;130(5):469-78. doi: 10.1037/bne0000160.
Animal
82
Sansa G, Gavaldà A, Gaig C, Monreal J, Ercilla G, Casamitjana R, Ribera G, Iranzo A, Santamaria J. Exploring the presence of narcolepsy in patients with schizophrenia. BMC Psychiatry. 2016;16:177. doi: 10.1186/s12888-016-0859-9.
Dupl 169PM
83
Schweitzer PK, Feren SD. Pharmacological treatment of insomnia. In Attarian HP (ed.), Clinical Handbook of Insomnia, 3rd ed. Totowa, NJ: Humana Press/Springer Nature, 2017; pp. 97-132. https://doi.org/10.1007/978-3-319-41400-3_7.
Review
84

Zink, Anastasia N. Neuromodulation of orexin neurons in the lateral hypothalamus regulates spontaneous physical activity, energy expenditure, and diet-induced obesity. Dissertation Abstracts International: Section B: The Sciences and Engineering, 2017;78:3-B(E). PhD Thesis, University of Minnesota, August 2015.
Animal
85
Mahoney CE, Agostinelli LJ, Brooks JN, Lowell BB, Scammell TE. GABAergic neurons of the central amygdala promote cataplexy. J Neurosci. 2017;37(15):3995-4006. doi: 10.1523/JNEUROSCI.4065-15.2017. Epub 2017 Feb 24.
Dupl 176PM
86
Bunney PE, Zink AN, Holm AA, Billington CJ, Kotz CM. Orexin activation counteracts decreases in nonexercise activity thermogenesis (NEAT) caused by high-fat diet. Physiol Behav. 2017;176:139-148. doi: 10.1016/j.physbeh.2017.03.040. Epub 2017 Mar 28.
Dupl 178PM
87
Colom-Lapetina J, Begley SL, Johnson ME, Bean KJ, Kuwamoto WN, Shansky RM. Strain-dependent sex differences in a long-term forced swim paradigm. Behav Neurosci. 2017;131(5):428-36. doi: 10.1037/bne0000215. Epub 2017 Aug 14.
Animal
88
Dixon ML, Thiruchselvam R, Todd R, Christoff K. Emotion and the prefrontal cortex: An integrative review. Psychol Bull. 2017;143(10):1033-1081. doi: 10.1037/bul0000096. Epub 2017 Jun 15.
Review
89
Campbell EJ, Mitchell CS, Adams CD, Yeoh JW, Hodgson DM, Graham BA, Dayas CV. Chemogenetic activation of the lateral hypothalamus reverses early life stress-induced deficits in motivational drive. Eur J Neurosci. 2017;46(7):2285-2296. doi: 10.1111/ejn.13674. Epub 2017 Sep 22.
Dupl 184PM
90
Keks NA, Hope J, Keogh S. Suvorexant: scientifically interesting, utility uncertain. Australas Psychiatry. 2017;25(6):622-624. doi: 10.1177/1039856217734677. Epub 2017 Oct 10.
Dupl 186PM
91
Bolton JL, Ruiz CM, Rismanchi N, Sanchez GA, Castillo E, Huang J, Cross C, Baram TZ, Mahler SV. Early-life adversity facilitates acquisition of cocaine self-administration and induces persistent anhedonia. Neurobiol Stress. 2018;8:57-67. doi: 10.1016/j.ynstr.2018.01.002.
Dupl 204PM
92
Dujardin S, Pijpers A, Pevernagie D. Prescription drugs used in insomnia. Sleep Med Clin. 2018;13(2):169-182. doi: 10.1016/j.jsmc.2018.03.001.
Dupl 203PM
93
Öz P, Gökalp HK, Göver T, Uzbay T. Dose-dependent and opposite effects of orexin A on prepulse inhibition response in sleep-deprived and non-sleep-deprived rats. Behav Brain Res. 2018;346:73-79. doi: 10.1016/j.bbr.2017.12.002. Epub 2017 Dec 10.
Dupl 190PM
94
Risco S, Mediavilla C. Orexin A in the ventral tegmental area enhances saccharin-induced conditioned flavor preference: The role of D1 receptors in central nucleus of amygdala. Behav Brain Res. 2018;348:192-200. doi: 10.1016/j.bbr.2018.04.010. Epub 2018 Apr 21.
Dupl 201PM
95
Juvodden HT, Alnæs D, Lund MJ, Agartz I, Andreassen OA, Dietrichs E, Thorsby PM, Westlye LT, Knudsen S. Widespread white matter changes in post-H1N1 patients with narcolepsy type 1 and first-degree relatives. Sleep. 2018;41(10):zsy145. doi: 10.1093/sleep/zsy145.
Dupl 207PM
96
Prihodova I, Dudova I, Mohaplova M, Hrdlicka M, Nevsimalova S. Childhood narcolepsy and autism spectrum disorders: four case reports. Sleep Med. 2018;51:167-170. doi: 10.1016/j.sleep.2018.07.017. Epub 2018 Aug 9.
Dupl 216PM
97
Chen P-Y, Chen C-H, Chang C-K, Kao C-F, Lu M-L, Lin S-K, Huang M-C, Hwang L-L, Mondelli V. Orexin-A levels in relation to the risk of metabolic syndrome in patients with schizophrenia taking antipsychotics. Int J Neuropsychopharmacol. 2019;22(1):28-36. doi: 10.1093/ijnp/pyy075.
Dupl 215PM
98
Baykal S, Albayrak Y, Durankuş F, Güzel S, Abbak Ö, Potas N, Beyazyüz M, Karabekiroğlu K, Donma MM. Decreased serum orexin A levels in drug-naive children with attention deficit and hyperactivity disorder. Neurol Sci. 2019;40(3):593-602. doi: 10.1007/s10072-018-3692-8. Epub 2019 Jan 7.
No psychosis
99
Prieto DI, Zehgeer AA, Connor DF. Use of Suvorexant for Sleep Regulation in an Adolescent with Early-Onset Bipolar Disorder. J Child Adolesc Psychopharmacol. 2019;29(5):395. doi: 10.1089/cap.2019.0029. Epub 2019 Apr 26.
Case
100
Misiak B, Bartoli F, Stramecki F, Samochowiec J, Lis M, Kasznia J, Jarosz K, Stańczykiewicz B. Appetite regulating hormones in first-episode psychosis: A systematic review and meta-analysis. Neurosci Biobehav Rev. 2019;102:362-370. doi: 10.1016/j.neubiorev.2019.05.018. Epub 2019 May 20.
Dupl 228PM
101
Tsuchimine S, Hattori K, Ota M, Hidese S, Teraishi T, Sasayama D, Hori H, Noda T, Yoshida S, Yoshida F, Kunugi H. Reduced plasma orexin-A levels in patients with bipolar disorder. Neuropsychiatr Dis Treat. 2019;15:2221-2230. doi: 10.2147/NDT.S209023.
Dupl 235PM
102
Krystal AD, Prather AA, Ashbrook LH. The assessment and management of insomnia: an update. World Psychiatry. 2019;18(3):337-352. doi: 10.1002/wps.20674.
Dupl 234PM

103
Ni P, Tian Y, Gu X, Yang L, Wei J, Wang Y, Zhao L, Zhang Y, Zhang C, Li L, Tang X, Ma X, Hu X, Li T. Plasma neuropeptides as circulating biomarkers of multifactorial schizophrenia. Compr Psychiatry. 2019;94:152114. doi: 10.1016/j.comppsych.2019.152114. Epub 2019 Aug 5.
Dupl 232PM
104
Hanazawa T, Kamijo Y. Effect of suvorexant on nocturnal delirium in elderly patients with Alzheimer's disease: A case-series study. Clin Psychopharmacol Neurosci. 2019;17(4):547-550. doi: 10.9758/cpn.2019.17.4.547.
Dupl 238PM 36S
105
Lu G-L, Lee MT, Chiou L-C. Orexin-mediated restoration of hippocampal synaptic potentiation in mice with established cocaine-conditioned place preference. Addict Biol. 2019;24(6):1153-1166. doi: 10.1111/adb.12672. Epub 2018 Oct 1.
Dupl 218PM
106
Norozpour Y, Zarrabian S, Rezaee L, Haghparast A. D1- and D2-like receptors in the dentate gyrus region of the hippocampus are involved in the reinstatement induced by a subthreshold dose of morphine and forced swim stress in extinguished morphine-CPP in rats. Behav Neurosci. 2019;133(6):545-555. doi: 10.1037/bne0000335.
Animal
107
Hatta K. Delirium. In Chopra A, Das P, Doghramji K (eds.). Management of Sleep Disorders in Psychiatry. Oxford (UK): Oxford University Press, 2020, pp. 402-411. https://doi.org/10.1093/med/9780190929671.001.0001. https://doi.org/10.1093/med/9780190929671.003.0024.
Review
108
Chamera K, Trojan E, Szuster-Głuszczak M, Basta-Kaim A. The potential role of dysfunctions in neuron-microglia communication in the pathogenesis of brain disorders. Curr Neuropharmacol. 2020;18(5):408-430. doi: 10.2174/1570159X17666191113101629.
Dupl 239PM
109
Feketeova E, Tormasiova M, Klobučníková K, Durdik P, Jarcuskova D, Benca M, Vitkova M. Narcolepsy in Slovakia - Epidemiology, clinical and polysomnographic features, comorbid diagnoses: a case-control study. Sleep Med. 2020;67:15-22. doi: 10.1016/j.sleep.2019.10.012. Epub 2019 Nov 11.
Dupl 244PM
110
Choi MR, Cho H, Chun JW, Yoo JH, Kim DJ. Increase of orexin A in the peripheral blood of adolescents with Internet gaming disorder. J Behav Addict. 2020;9(1):93-104. doi: 10.1556/2006.8.2019.65.
No psychosis
111
Rezaee L, Alizadeh AM, Haghparast A. Role of hippocampal dopamine receptors in the antinociceptive responses induced by chemical stimulation of the lateral hypothalamus in animal model of acute pain. Brain Res. 2020;1734:146759. doi: 10.1016/j.brainres.2020.146759. Epub 2020 Mar 2.
Dupl 245PM
112
Akça ÖF, Uzun N, Kılınç İ. Orexin A in adolescents with anxiety disorders. Int J Psychiatry Clin Pract. 2020;24(2):127-134. doi: 10.1080/13651501.2019.1711425.
No psychosis
113
Lis M, Stańczykiewicz B, Liśkiewicz P, Misiak B. Impaired hormonal regulation of appetite in schizophrenia: A narrative review dissecting intrinsic mechanisms and the effects of antipsychotics. Psychoneuroendocrinology. 2020;119:104744. doi: 10.1016/j.psyneuen.2020.104744. Epub 2020 Jun 5.
Dupl 251PM
114
Liu Z, Zhang Y, Zhao T, Wang J, Xia L, Zhong Y, Yang Y, Ning X, Zhang Y, Ren Z, Liu H. A higher body mass index in Chinese inpatients with chronic schizophrenia is associated with elevated plasma orexin-A levels and fewer negative symptoms. Nord J Psychiatry. 2020;74(7):525-532. doi: 10.1080/08039488.2020.1755995. Epub 2020 May 4.
Dupl 248PM
115
Rosenblat, Joshua D. Novel therapeutic drug targets for bipolar disorder. In: Quevedo J, Ferrer Carvalho A, Vieta E (eds.) Neurobiology of Bipolar Disorder: Road to Novel Therapeutics. London (UK): Academic Press-Elsevier, 2021, pp. 393-404. https://doi.org/10.1016/B978-0-12-819182-8.00037-5.
Review
116
Procyshyn RM, Bezchlibnyk-Butler KZ, Jeffries JJ (Eds.). Clinical Handbook of Psychotropic Drugs, 24th ed. Newburyport, MA: Hogrefe, 2021. https://doi.org/10.1027/00593-000.
Review
117
Khaleghzadeh-Ahangar H, Rashvand M, Haghparast A. Role of D1- and D2-like dopamine receptors within the dentate gyrus in antinociception induced by chemical stimulation of the lateral hypothalamus in an animal model of acute pain. Physiol Behav. 2021;229:113214. doi: 10.1016/j.physbeh.2020.113214. Epub 2020 Oct 20.
Dupl 258PM
118
Durairaja A, Fendt M. Orexin deficiency modulates cognitive flexibility in a sex-dependent manner. Genes Brain Behav. 2021;20(3):e12707. doi: 10.1111/gbb.12707. Epub 2020 Nov 3.
Dupl 257PM
119
Perez SM, Lodge DJ. Orexin modulation of VTA dopamine neuron activity: Relevance to schizophrenia. Int J Neuropsychopharmacol. 2021;24(4):344-353. doi: 10.1093/ijnp/pyaa080.
Dupl 268PM
120
Yoon JH, Suchting R, Cassidy RN, Bolin PK, Omar Y, Brown GS, De La Garza R. Assessment of demand for methamphetamine and cigarettes among individuals with methamphetamine use disorder. Exp Clin Psychopharmacol. 2021;29(4):334-344. doi: 10.1037/pha0000367.
No psychosis
121

Charntikov S, Pittenger ST, Swalve N, Barrett ST, Bevins RA. Conditioned enhancement of the nicotine reinforcer. Exp Clin Psychopharmacol. 2021;29(4):385-394. doi: 10.1037/pha0000370.
Animal
122
Lu J, Huang ML, Li JH, Jin KY, Li HM, Mou TT, Fronczek R, Duan JF, Xu WJ, Swaab D, Bao AM. Changes of hypocretin (Orexin) system in schizophrenia: From plasma to brain. Schizophr Bull. 2021;47(5):1310-1319. doi: 10.1093/schbul/sbab042.
Dupl 271PM
123
Akça ÖF, Sağlam E, Kılınç I, Bilgiç A. Orexin A levels of adolescents with major depressive disorder. Int J Psychiatry Clin Pract. 2021;25(4):403-406. doi: 10.1080/13651501.2021.1927106.
No psychosis
124
Lee W-C, Chen P-Y, Kao C-F, Huang M-C. Differences in serum orexin-A levels between the acute and subacute withdrawal phases in individuals who use methamphetamine. Exp Clin Psychopharmacol. 2021;29(6):573-579. doi: 10.1037/pha0000395.
No psychosis
125
Terada T, Hirayama T, Sadahiro R, Wada S, Nakahara R, Matsuoka H. Pilot study of lemborexant for insomnia in cancer patients with delirium. J Palliat Med. 2022;25(5):797-801. doi: 10.1089/jpm.2021.0509. Epub 2022 Jan 28.
Dupl 282PM 49S
126
Li S, Zhang R, Hu S, Lai J. Plasma orexin-A levels in patients with schizophrenia: A systematic review and meta-analysis. Front Psychiatry. 2022;13:879414. doi: 10.3389/fpsyt.2022.879414.
Dupl 296PM
127
Demidova A, Kahl E, Fendt M. Orexin deficiency affects sensorimotor gating and its amphetamine-induced impairment. Prog Neuropsychopharmacol Biol Psychiatry. 2022;116:110517. doi: 10.1016/j.pnpbp.2022.110517. Epub 2022 Jan 29.
Dupl 283PM
128
Brown RE, Spratt TJ, Kaplan GB. Translational approaches to influence sleep and arousal. Brain Res Bull. 2022;185:140-161. doi: 10.1016/j.brainresbull.2022.05.002. Epub 2022 May 10.
Dupl 293PM
129
Palagini L, Hertenstein E, Riemann D, Nissen C. Sleep, insomnia and mental health. J Sleep Res. 2022;31(4):e13628. doi: 10.1111/jsr.13628. Epub 2022 May 4.
Dupl 292PM
130
González-Blanco L, Moya-Lacasa C, Jiménez-Fernández S, Martínez-Cao C, Valtueña-García M, Dal Santo F, García-Portilla MP, Luis Gutiérrez-Rojas L. Endocrine biomarkers related to sleep-wake cycle and sleep disturbances in patients with bipolar disorder: A systematic review. Eur J Psychiatry. 2022;36(4):223-229. DOI: 10.1016/j.ejpsy.2022.04.004.
Review
131
Bergamini G, Coloma P, Massinet H, Steiner MA. What evidence is there for implicating the brain orexin system in neuropsychiatric symptoms in dementia? Front Psychiatry. 2022;13:1052233. doi: 10.3389/fpsyt.2022.1052233.
Dupl 312PM
132
Smith DM, Terhune DB. Pedunculopontine-induced cortical decoupling as the neurophysiological locus of dissociation. Psychol Rev. 2023;130(1):183-210. doi: 10.1037/rev0000353.
Review
133
Seugnet L, Anaclet C, Perier M, Ghersi-Egea JF, Lin JS. A marked enhancement of a BLOC-1 gene, pallidin, associated with somnolent mouse models deficient in histamine transmission. CNS Neurosci Ther. 2023;29(1):483-486. doi: 10.1111/cns.13995.
Opinion
134
Pintwala, Sara Katherine. Transplanting immortal orexin cells in narcoleptic mice rescues cataplexy. PhD Thesis, University of Toronto, Dissertation Abstracts International: Section B: The Sciences and Engineering, 2023, volume 84, issue 5-BAPA.
Animal
135
Finkenberg DB, Shalev H, Vannikov-Lugassi M, Soffer-Dudek N. No evidence for selective serotonin reuptake inhibitors (SSRIs) use as explaining the relationship between sleep experiences and psychopathological distress. Psychol Conscious (Wash D C). 2023;10(1):50–61. https://doi.org/10.1037/cns0000275.
No psychosis
136
Yu H, Ni P, Zhao L, Tian Y, Li M, Li X, Wei W, Wei J, Deng W, Du X, Wang Q, Guo W, Ma X, Coid J, Li T. Decreased plasma neuropeptides in first-episode schizophrenia, bipolar disorder, major depressive disorder: associations with clinical symptoms and cognitive function. Front Psychiatry. 2023;14:1180720. doi: 10.3389/fpsyt.2023.1180720.
Dupl 330PM
137
Fagan HA, Baldwin DS. Pharmacological treatment of generalised anxiety disorder: Current practice and future directions. Expert Rev Neurother. 2023;23(6):535-548. doi: 10.1080/14737175.2023.2211767. Epub 2023 May 15.
Dupl 326PM 55S
138
Maness EB, Blumenthal SA, Burk JA. Dual orexin/hypocretin receptor antagonism attenuates NMDA receptor hypofunction-induced attentional impairments in a rat model of schizophrenia. Behav Brain Res. 2023;450:114497. doi: 10.1016/j.bbr.2023.114497. Epub 2023 May 16.
Dupl 327PM
139
Webber HE, Yoon JH, de Dios C, Suchting R, Dang V, Versace F, Green CE, Wardle MC, Lane SD, Schmitz JM. Assessing cocaine motivational value: Comparison of brain reactivity bias toward cocaine cues and cocaine demand. Exp Clin Psychopharmacol. 2023;31(4):861-867. doi: 10.1037/pha0000622.

No psychosis
140
Nakamura T, Yoshizawa T, Toya R, Terasawa M, Takahashi K, Kitazawa K, Suzuki K, Sasayama D, Washizuka S. Orexin receptor antagonists versus antipsychotics for the management of delirium in intensive care unit patients with cardiovascular disease: A retrospective observational study. Gen Hosp Psychiatry. 2023;84:96-101. doi: 10.1016/j.genhosppsych.2023.06.019. Epub 2023 Jul 1.
Dupl 331PM 60S
141
Hansen BH, Andresen HN, Gjesvik J, Thorsby PM, Naerland T, Knudsen-Heier S. Associations between psychiatric comorbid disorders and executive dysfunctions in hypocretin-1 deficient pediatric narcolepsy type1. Sleep Med. 2023;109:149-157. doi: 10.1016/j.sleep.2023.06.021. Epub 2023 Jul 1.
Dupl 332PM
142
Riemann D, Espie CA, Altena E, Arnardottir ES, Baglioni C, Bassetti CLA, Bastien C, Berzina N, Bjorvatn B, Dikeos D, Dolenc Groselj L, Ellis JG, Garcia-Borreguero D, Geoffroy PA, Gjerstad M, Gonçalves M, Hertenstein E, Hoedlmoser K, Hion T, Holzinger B, Janku K, Jansson-Fröjmark M, Järnefelt H, Jernelöv S, Jennum PJ, Khachatryan S, Krone L, Kyle SD, Lancee J, Leger D, Lupusor A, Marques DR, Nissen C, Palagini L, Paunio T, Perogamvros L, Pevernagie D, Schabus M, Shochat T, Szentkiralyi A, Van Someren E, van Straten A, Wichniak A, Verbraecken J, Spiegelhalder K. The European Insomnia Guideline: An update on the diagnosis and treatment of insomnia 2023. J Sleep Res. 2023;32(6):e14035. doi: 10.1111/jsr.14035.
Dupl 341PM
143
Lafrenière S, Blier P. Remission from severe treatment-resistant depression with moclobemide: A case report. Encéphale. 2023;49(6):649-650. doi: 10.1016/j.encep.2023.01.014.
Case
144
Mishima K. Pharmacologic treatment of insomnia. In: Tasman A, Riba MB, Alarcón RD, Alfonso CA, Kanba S, Lecic-Tosevski D, Ndetei DM, Ng CH, Schulze TG (eds.) Tasman's Psychiatry. Cham, CH: Springer 2024, pp. 4281-4302, https://doi.org/10.1007/978-3-030-51366-5_137.
Review
145
Pillai, M., Chandrasekhara, S. (2024). Insomnia. In: Tampi RR, Tampi DJ (eds) Treatment of Psychiatric Disorders Among Older Adults. Cham, CH: Springer, pp. 153-171. https://doi.org/10.1007/978-3-031-55711-8_11.
Review
146
Chen P-Y, Chiu C-C, Chang C-K, Lu M-L, Huang C-Y, Chen C-H, Huang M-C. Higher orexin-A levels are associated with treatment response to clozapine in patients with schizophrenia: A cross-sectional study. J Psychopharmacol. 2024;38(3):258-267. doi: 10.1177/02698811231225610. Epub 2024 Jan 27.
Dupl 347PM
147
DeGutis J, Sullivan DR, Agnoli S, Stumps A, Logue M, Brown E, Verfaellie M, Milberg W, McGlinchey R, Esterman M. Less is more: Smaller hippocampal subfield volumes predict greater improvements in posttraumatic stress disorder symptoms over 2 years. Behav Neurosci. 2024;138(2):94-107. doi: 10.1037/bne0000578.
No orexin
148
Liu X, Lan X, Zhang X, Ye H, Shen L, Hu M, Chen X, Zheng M, Weston-Green K, Jin T, Cui X, Zhou Y, Lu X, Huang XF, Yu Y. Olanzapine attenuates 5-HT2cR and GHSR1a interaction to increase orexigenic hypothalamic NPY: Implications for neuronal molecular mechanism of metabolic side effects of antipsychotics. Behav Brain Res. 2024;463:114885. doi: 10.1016/j.bbr.2024.114885.
Animal
149
Öz P, Kamalı O, Saka HB, Gör C, Uzbay İT. Baseline prepulse inhibition dependency of orexin A and REM sleep deprivation. Psychopharmacology (Berl). 2024;241(6):1213-1225. doi: 10.1007/s00213-024-06555-3. Epub 2024 Mar 1.
Dupl 352PM
150
Felix PC, Flagel SB. Leveraging individual differences in cue-reward learning to investigate the psychological and neural basis of shared psychiatric symptomatology: The sign-tracker/goal-tracker model. Behav Neurosci. 2024;138(4):260-271. doi: 10.1037/bne0000590.
Review
151
Kikuchi Y, Kurosawa M, Sakata M, Takahashi Y, Yamamoto K, Tomita H, Yoshio T, Yasui-Furukori N. Effects of titration speed, gender, obesity and concomitant medications on the risk and onset time of clozapine-associated fever among Japanese patients with schizophrenia: retrospective review of charts from 21 hospitals. Br J Psychiatry. 2024;225(5):492-498. doi: 10.1192/bjp.2024.113.
Dupl 368PM 72S
152
Geldmacher DS. Treatment of Alzheimer disease. Continuum (Minneap Minn). 2024;30(6):1823-1844. doi: 10.1212/CON.0000000000001503.
Dupl 379PM
153
Hur J, Tillman RM, Kim HC, Didier P, Anderson AS, Islam S, Stockbridge MD, De Los Reyes A, DeYoung KA, Smith JF, Shackman AJ. Adolescent social anxiety is associated with diminished discrimination of anticipated threat and safety in the bed nucleus of the stria terminalis. J Psychopathol Clin Sci. 2025;134(1):41-56. doi: 10.1037/abn0000940. Epub 2024 Nov 7.
No psychosis
154
Spiegelhalder K, Nissen C, Riemann D. The impact of prescription and recreational drugs on sleep. In Espie CA, Zee PC, Morin CM (eds.) The Oxford Handbook of Sleep and Sleep Disorders., 2nd ed. Oxford (UK): Oxford University Press, 2025, pp. 458-477. https://doi.org/10.1093/oxfordhb/9780197602751.013.10.
Review
155
Havlik JL, Rhee TG, Rosenheck RA. Correlates of impaired sleep duration among adults with trauma alone and with posttraumatic stress disorder. Psychol Trauma. 2025. doi: 10.1037/tra0001846. Epub ahead of print 2025 Jan 20.
No orexin
156
Barrett ST, McNealy KR, Knabel ML, Burrichter RM, Steck KA, Bevins RA. The inside story: Interoceptive Pavlovian conditioning with the nicotine stimulus. J Exp Psychol Anim Learn Cogn. 2025;51(2):61-72. doi: 10.1037/xan0000393.
Review

157

Chekani F, Mirchandani K, Zaki S, Goswami S, Sharma M. Utilization of potentially inappropriate sedative-hypnotic and atypical antipsychotic medications among elderly individuals with insomnia and Alzheimer's disease. Sleep. 2025;48(4):zsaf003. doi: 10.1093/sleep/zsaf003.

Dupl 392PM

158

Dumont S, Bloch V, Lillo-Lelouet A, Le Beller C, Geoffroy PA, Veyrier M. Parasomnias and sleep-related movement disorders induced by drugs in the adult population: a review about iatrogenic medication effects. J Sleep Res. 2025;34(2):e14306. doi: 10.1111/jsr.14306. Epub 2024 Sep 7.

Dupl 371PM

159

Regnier SD, Chirica MG, Acuff SF, Strickland JC. Toward better outcomes: Evolving psychopharmacologic approaches in youth psychiatry. Transl Issues Psychol Sci. 2025;11(3):277-280. https://doi.org/10.1037/tps0000472.

Review

160

Torres C, Papini MR, Huffman MA. Psychological self-medication in mammals: A dialogue between natural observations and laboratory research. J Comp Psychol. 2025. doi: 10.1037/com0000428. Epub ahead of print 2025 Sep 15.

Animal

161

Funayama M, Sato K, Nakagawa Y. Thermoregulatory dysfunction in the chronic phase of cerebrovascular disease: Hypothalamus damage and behavior control deficits. Neuropsychology. 2025. doi: 10.1037/neu0001046. Epub ahead of print 2025 Oct 16.

Unfocused

162

Barrett ST, McNealy KR, Knabel ML, Burrichter RM, Steck KA, Bevins RA. The inside story: Interoceptive Pavlovian conditioning with the nicotine stimulus. J Exp Psychol Anim Learn Cogn. 2025;51(2):61-72. doi: 10.1037/xan0000393.

Dupl 156PM

163

Dumont S, Bloch V, Lillo-Lelouet A, Le Beller C, Geoffroy PA, Veyrier M. Parasomnias and sleep-related movement disorders induced by drugs in the adult population: a review about iatrogenic medication effects. J Sleep Res. 2025;34(2):e14306. doi: 10.1111/jsr.14306. Epub 2024 Sep 7.

Review

164

Baskerville W-A, Grodin EN, Meredith LR, Ray LA. Interplay between alcohol cues and mood states during early abstinence: A daily diary study. Exp Clin Psychopharmacol. 2025;33(3):260-268. doi: 10.1037/pha0000770. Epub 2025 Mar 13.

No orexin

165

Kawashima H, Waddington JL, Saigusa T. Reduced accumbal dopamine efflux via orexin OX2 receptors in chronic pain models. Eur J Neurosci. 2025;62(1):e70192. doi: 10.1111/ejn.70192.

Animal

166

Garcia-Borreguero D, Anguizola D, Carvallo C, Lopez A, Garcia Aragón A, Moncada B, Ferré S. Subclinical Augmentation in Relation to Previous Dopaminergic Treatment in Patients with Restless Legs Syndrome: A Post Hoc Analysis of Two Randomized, Placebo-Controlled, Crossover Trials. CNS Drugs. 2025;39(8):779-793. doi: 10.1007/s40263-025-01192-6. Epub 2025 Jun 4.

No psychosis

167

Clark PJ, Migovich VM, Das S, Xu W, Zhang Y, Kortagere S, España RA. Hypocretin receptor 1 blockade early in abstinence prevents incubation of cocaine seeking and normalizes dopamine transmission. Neuropsychopharmacology. 2026. doi: 10.1038/s41386-025-02315-9. Epub ahead of print 2026 Feb 4.

Animal

(schizophr* OR psychosis OR psychotic OR paranoi* OR schizoaffect* OR antipsychotic* OR neuroleptic* OR phenothiazine* OR butyrophenon* OR "dopamine antagonist*" OR "dopamine receptor antagonist*" OR "substituted benzamide*" OR thioxanthene* OR dibenzoazepine* OR benzisoxazole* OR haloperidol OR chlorpromazine OR promazine OR thioridazine OR clothiapine OR loxapine OR clozapine OR quetiapine OR olanzapine OR fluperlapin* OR zotepine OR remoxipride OR sertindole OR risperidone OR paliperidone OR aripiprazole OR brexpiprazole OR cariprazine OR ziprasidone OR asenapine OR bromperidol OR spiroperidol OR spiperone OR perphenazine OR fluphenazine OR flupentixol* OR clopenthixol OR zuclopenthixol OR droperidol OR thioxanthene OR sulpiride OR sultopride OR metoclopramide OR amisulpride) AND (orexin* OR hypocretin* OR Hcrts OR OX1 OR OX2 OR OX1R OR OX2R OR suvorexant OR MK-4305 OR lemborexant OR daridorexant OR almorexant OR ACT-078573 OR filorexant OR MK-6096 OR seltorexant OR MIN-202 OR JNJ-42847922 OR JNJ-922 OR Fazamorexant OR YZJ-1139 OR Nivasorexant OR ACT-539313 OR Tebideutorexant OR JNJ-61393215 OR JNJ-3215 OR Vornorexant OR ORN-0829 OR TS-142 OR ACT-335827 OR EMPA OR GSK-649868 OR SB-649868 OR JNJ-10397049 OR RTIOX-276 OR SB-334867 OR SB-408124 OR TCS-OX2-29) CINAHL 3.3.2026 → 30 results

1

Monda M, Viggiano A, Viggiano A, Viggiano E, De Luca V. Risperidone potentiates the sympathetic and hyperthermic reactions induced by orexin A in the rat. Physiol Res. 2006;55(1):73-78. doi: 10.33549/physiolres.930906. Epub 2005 Apr 26.

Dupl 51PM

2

Stefanidis A, Verty AN, Allen AM, Owens NC, Cowley MA, Oldfield BJ. The role of thermogenesis in antipsychotic drug-induced weight gain. Obesity (Silver Spring). 2009;17(1):16-24. doi: 10.1038/oby.2008.468. Epub 2008 Oct 30.

Dupl 93PM 35Ps

3

Burgess CR, Tse G, Gillis L, Peever JH. Dopaminergic regulation of sleep and cataplexy in a murine model of narcolepsy. Sleep. 2010;33(10):1295-304. doi: 10.1093/sleep/33.10.1295.

Dupl 103PM

4

Li X, Johnson MS, Smith DL Jr, Li Y, Kesterson RA, Allison DB, Nagy TR. Effects of risperidone on energy balance in female C57BL/6J mice. Obesity (Silver Spring). 2013;21(9):1850-7. doi: 10.1002/oby.20350. Epub 2013 May 29.

Dupl 118PM

5

Pizza F, Magnani M, Indrio C, Plazzi G. The hypocretin system and psychiatric disorders. Curr Psychiatry Rep. 2014;16(2):433. doi: 10.1007/s11920-013-0433-9.

Dupl 127PM

6

Hasegawa E, Yanagisawa M, Sakurai T, Mieda M. Orexin neurons suppress narcolepsy via 2 distinct efferent pathways. J Clin Invest. 2014;124(2):604-16. doi: 10.1172/JCI71017. Epub 2014 Jan 2.

Dupl 128PM

7

Chen YW, Morganstern I, Barson JR, Hoebel BG, Leibowitz SF. Differential role of D1 and D2 receptors in the perifornical lateral hypothalamus in controlling ethanol drinking and food intake: possible interaction with local orexin neurons. Alcohol Clin Exp Res. 2014;38(3):777-86. doi: 10.1111/acer.12313. Epub 2013 Nov 15.
Dupl 128PM 28Sc
8
Chen YW, Morganstern I, Barson JR, Hoebel BG, Leibowitz SF. Differential role of D1 and D2 receptors in the perifornical lateral hypothalamus in controlling ethanol drinking and food intake: possible interaction with local orexin neurons. Alcohol Clin Exp Res. 2014;38(3):777-86. doi: 10.1111/acer.12313. Epub 2013 Nov 15.
Dupl 128PM 28Sc 7C
9
Szabo ST, Kinon BJ, Brannan SK, Krystal AK, van Gerven JM, Mahableshwarkar A, Sachs GS. Lessons learned and potentials for improvement in CNS drug development: ISCTM Section on Designing the right series of experiments. Innov Clin Neurosci. 2015;12(3Suppl A):11S-25S.
Dupl 26S
10
Barandas R, Landgraf D, McCarthy MJ, Welsh DK. Circadian Clocks as Modulators of Metabolic Comorbidity in Psychiatric Disorders. Curr Psychiatry Rep. 2015;17(12):98. doi: 10.1007/s11920-015-0637-2.
Dupl 155PM
11
Vickers SP, Hackett D, Murray F, Hutson PH, Heal DJ. Effects of lisdexamfetamine in a rat model of binge-eating. J Psychopharmacol. 2015;29(12):1290-307. doi: 10.1177/0269881115615107. Epub 2015 Nov 20.
Dupl 156PM 30S 72P
12
Nakamura M, Nagamine T. Neuroendocrine, autonomic, and metabolic responses to an orexin antagonist, suvorexant, in psychiatric patients with insomnia. Innov Clin Neurosci. 2017;14(3-4):30-37.
Lumping
13
Matheson E, Hainer BL. Insomnia: Pharmacologic Therapy. Am Fam Physician. 2017;96(1):29-35.
Opinion
14
Keks NA, Hope J, Keogh S. Suvorexant: scientifically interesting, utility uncertain. Australas Psychiatry. 2017;25(6):622-624. doi: 10.1177/1039856217734677. Epub 2017 Oct 10.
Dupl 186PM 90Ps
15
McElroy SL, Guerdjikova AI, Mori N, Romo-Nava F. Progress in Developing Pharmacologic Agents to Treat Bulimia Nervosa. CNS Drugs. 2019;33(1):31-46. doi: 10.1007/s40263-018-0594-5.
Dupl 223PM
16
Ni P, Tian Y, Gu X, Yang L, Wei J, Wang Y, Zhao L, Zhang Y, Zhang C, Li L, Tang X, Ma X, Hu X, Li T. Plasma neuropeptides as circulating biomarkers of multifactorial schizophrenia. Compr Psychiatry. 2019;94:152114. doi: 10.1016/j.comppsych.2019.152114. Epub 2019 Aug 5.
Dupl 232PM 103Ps
17
Lu G-L, Lee MT, Chiou L-C. Orexin-mediated restoration of hippocampal synaptic potentiation in mice with established cocaine-conditioned place preference. Addict Biol. 2019;24(6):1153-1166. doi: 10.1111/adb.12672. Epub 2018 Oct 1.
Dupl 218PM 105Ps
18
BaHammam AS, Alnakshabandi K, Pandi-Perumal SR. Neuropsychiatric Correlates of Narcolepsy. Curr Psychiatry Rep. 2020;22(8):36. doi: 10.1007/s11920-020-01159-y.
Dupl 250PM
19
Lu J, Huang ML, Li JH, Jin KY, Li HM, Mou TT, Fronczek R, Duan JF, Xu WJ, Swaab D, Bao AM. Changes of hypocretin (Orexin) system in schizophrenia: From plasma to brain. Schizophr Bull. 2021;47(5):1310-1319. doi: 10.1093/schbul/sbab042.
Dupl 271PM 122Ps
20
Porwal A, Yadav YC, Pathak K, Yadav R. An update on assessment, therapeutic management, and patents on insomnia. Biomed Res Int. 2021;2021:6068952. doi: 10.1155/2021/6068952.
Dupl 278PM
21
Terada T, Hirayama T, Sadahiro R, Wada S, Nakahara R, Matsuoka H. Pilot study of lemborexant for insomnia in cancer patients with delirium. J Palliat Med. 2022;25(5):797-801. doi: 10.1089/jpm.2021.0509. Epub 2022 Jan 28.
Dupl 282PM 49S 125Ps
22
Chen PY, Chang CK, Chen CH, Fang SC, Mondelli V, Chiu CC, Lu ML, Hwang LL, Huang MC. Orexin-a elevation in antipsychotic-treated compared to drug-free patients with schizophrenia: A medication effect independent of metabolic syndrome. J Formos Med Assoc. 2022;121(11):2172-2181. doi: 10.1016/j.jfma.2022.03.008. Epub 2022 Apr 6.
Dupl 289PM
23
Matsuoka A, Sogawa R, Murakawa-Hirachi T, Mizoguchi Y, Monji A, Shimanoe C, Shinada K, Koami H, Sakamoto Y. Evaluation of the delirium preventive effect of dual orexin receptor antagonist (DORA) in critically ill adult patients requiring ventilation with tracheal intubation at an advanced emergency center: A single-center, retrospective, observational study. Gen Hosp Psychiatry. 2023;83:123-129. doi: 10.1016/j.genhosppsych.2023.03.010. Epub 2023 Mar 31.
Unfocused
24
Matsumoto S, Tamiya H, Yamana H, Hosoi T, Matsui H, Fushimi K, Akishita M, Yasunaga H, Ogawa S. Association between the type of hypnotic drug and in-hospital fractures in older patients with neurocognitive disorders: A case-control study using a nationwide database. Geriatr Gerontol Int. 2023;23(7):500-505. doi: 10.1111/ggi.14600. Epub 2023 May 22.
Unfocused
25

Nakamura T, Yoshizawa T, Toya R, Terasawa M, Takahashi K, Kitazawa K, Suzuki K, Sasayama D, Washizuka S. Orexin receptor antagonists versus antipsychotics for the management of delirium in intensive care unit patients with cardiovascular disease: A retrospective observational study. Gen Hosp Psychiatry. 2023;84:96-101. doi: 10.1016/j.genhosppsych.2023.06.019. Epub 2023 Jul 1.
Dupl 331PM 60S 140Ps
26
Chen P-Y, Chiu C-C, Chang C-K, Lu M-L, Huang C-Y, Chen C-H, Huang M-C. Higher orexin-A levels are associated with treatment response to clozapine in patients with schizophrenia: A cross-sectional study. J Psychopharmacol. 2024;38(3):258-267. doi: 10.1177/02698811231225610. Epub 2024 Jan 27.
Dupl 347PM 146Ps
27
Öz P, Kamalı O, Saka HB, Gör C, Uzbay İT. Baseline prepulse inhibition dependency of orexin A and REM sleep deprivation. Psychopharmacology (Berl). 2024;241(6):1213-1225. doi: 10.1007/s00213-024-06555-3. Epub 2024 Mar 1.
Dupl 352PM 149Ps
28
Takeshima M, Sakurai H, Inada K, Aoki Y, Ie K, Kise M, Yoshida E, Matsui K, Utsumi T, Shimura A, Okajima I, Kotorii N, Yamashita H, Suzuki M, Kuriyama K, Shimizu E, Mishima K, Watanabe K, Takaesu Y. Treatment strategies for insomnia in Japanese primary care physicians' practice: A Web-based questionnaire survey. BMC Prim Care. 2024;25(1):219. doi: 10.1186/s12875-024-02449-7.
Unrelated
29
Baum E, Philipp M, Spiegelhalder K. Insomnie – Update der S3-Leitlinie. Relevantes für den hausärztlichen Bereich [Insomnia—update of the S3 guideline. With relevance for general practice]. ZFA Z Allg Med (Stuttgart). 2025;101(6):303-307. doi: 10.1007/s44266-025-00387-w.
Review
30
Seltorexant improved sleep outcomes in patients with insomnia disorder. Brown University Psychopharmacology Update, 2025;36(12):6-7. doi: 10.1002/pu.31383 (refers to Mesens S, Krystal AD, Melkote R, Xu H, Pandina G, Saoud JB, Luthringer R, Savitz A, Drevets WC. Efficacy and safety of seltorexant in insomnia disorder: A randomized clinical trial. JAMA Psychiatry. 2025;82(10):967-76. doi: 10.1001/jamapsychiatry.2025.1999. Epub ahead of print. No psychosis).
Opinion

**List of articles found in all searched databases and results per label.**

| N° | Article (authors, title, source) | Label |
|---|---|---|
| 1. | Boullin DJ, Grimes RP. Increased platelet aggregation in patients receiving chlorpromazine: responses to 5-hydroxytryptamine, dopamine and N-dimethyl dopamine. Br J Clin Pharmacol. 1976;3(4):649-53. doi: 10.1111/j.1365-2125.1976.tb04889.x. | No orexin |
| 2. | Iversen SD. Interactions between excitatory amino acids and dopamine systems in the forebrain: implications for schizophrenia and Parkinson's disease. Behav Pharmacol. 1995;6(5-6):478-491. | Review |
| 3. | Pedersen ME, Dorrington KL, Robbins PA. Effects of haloperidol on ventilation during isocapnic hypoxia in humans. J Appl Physiol (1985). 1997;83(4):1110-5. doi: 10.1152/jappl.1997.83.4.1110. | No orexin |
| 4. | Thornley B, Adams C. Content and quality of 2000 controlled trials in schizophrenia over 50 years. BMJ. 1998;317(7167):1181-4. doi: 10.1136/bmj.317.7167.1181. | Review |
| 5. | Pedersen ME, Dorrington KL, Robbins PA. Effects of dopamine and domperidone on ventilatory sensitivity to hypoxia after 8 h of isocapnic hypoxia. J Appl Physiol (1985). 1999;86(1):222-9. doi: 10.1152/jappl.1999.86.1.222. | No orexin |
| 6. | Stein J, Richardson A. Cognitive disorders: A question of misattribution. Curr Biol. 1999;9(10):R374-6. doi: 10.1016/s0960-9822(99)80231-0. | No orexin |
| 7. | Newberry NR, Footitt DR, Papanastassiou V, Reynolds DJ. Actions of 5-HT on human neocortical neurones in vitro. Brain Res. 1999;833(1):93-100. doi: 10.1016/s0006-8993(99)01540-1. | No orexin |
| 8. | Thornley B, Adams CE, Awad G. Chlorpromazine versus placebo for schizophrenia. Cochrane Database Syst Rev. 2000;(2):CD000284. doi: 10.1002/14651858.CD000284. Update in: Cochrane Database Syst Rev. 2003;(2):CD000284. doi: 10.1002/14651858.CD000284. | No orexin |
| 9. | Adams CE, Eisenbruch M. Depot fluphenazine for schizophrenia. Cochrane Database Syst Rev. 2000;(2):CD000307. doi: 10.1002/14651858.CD000307. Update in: Cochrane Database Syst Rev. 2005;(1):CD000307. doi: 10.1002/14651858.CD000307. | Review |
| 10. | Joy CB, Adams CE, Rice K. Crisis intervention for people with severe mental illnesses. Cochrane Database Syst Rev. 2000;(2):CD001087. doi: 10.1002/14651858.CD001087. Update in: Cochrane Database Syst Rev. 2004;(4):CD001087. doi: 10.1002/14651858.CD001087.pub2. | Review |
| 11. | Joy CB, Mumby-Croft R, Joy LA. Polyunsaturated fatty acid (fish or evening primrose oil) for schizophrenia. Cochrane Database Syst Rev. 2000;(2):CD001257. doi: 10.1002/14651858.CD001257. Update in: Cochrane Database Syst Rev. 2003;(2):CD001257. doi: 10.1002/14651858.CD001257. | Review |
| 12. | Nakamura T, Uramura K, Nambu T, Yada T, Goto K, Yanagisawa M, Sakurai T. Orexin-induced hyperlocomotion and stereotypy are mediated by the dopaminergic system. Brain Res. 2000;873(1):181-7. doi: 10.1016/s0006-8993(00)02555-5. | Animal |
| 13. | Russell SH, Kim MS, Small CJ, Abbott CR, Morgan DG, Taheri S, Murphy KG, Todd JF, Ghatei MA, Bloom SR. Central administration of orexin A suppresses basal and domperidone stimulated plasma prolactin. J Neuroendocrinol. 2000;12(12):1213-8. | Animal |
| 14. | Adams C, Wilson P, Bagnall AM. Psychosocial interventions for schizophrenia. Qual Health Care. 2000;9(4):251-6. doi: 10.1136/qhc.9.4.251. | Review |
| 15. | Canales JJ, Iversen SD. Psychomotor-activating effects mediated by dopamine $D_2$ and $D_3$ receptors in the nucleus accumbens. Pharmacol Biochem Behav. 2000;67(1):161-8. doi: 10.1016/s0091-3057(00)00311-7. | Animal |
| 16. | Howson N, Adams CE. Always search this year's disc. Health Libr Rev. 2000;17(3):171-2. doi: 10.1046/j.1365-2532.2000.00282.x. | Review |
| 17. | Greene JR, Kerkhoff JE, Guiver L, Totterdell S. Structural and functional abnormalities of the hippocampal formation in rats with environmentally induced reductions in prepulse inhibition of acoustic startle. Neuroscience. 2001;103(2):315-23. doi: 10.1016/s0306-4522(00)00560-1. | Animal |
| 18. | Carpenter S, Berk M. Clotiapine for acute psychotic illnesses. Cochrane Database Syst Rev. 2001;(1):CD002304. doi: 10.1002/14651858.CD002304. Update in: Cochrane Database Syst Rev. 2004;(4):CD002304. doi: 10.1002/14651858.CD002304.pub2. | Review |
| 19. | Wolfart J, Neuhoff H, Franz O, Roeper J. Differential expression of the small-conductance, calcium-activated potassium channel SK3 is critical for pacemaker control in dopaminergic midbrain neurons. J Neurosci. 2001;21(10):3443-56. doi: 10.1523/JNEUROSCI.21-10-03443.2001. | Animal |
| 20. | Joy CB, Adams CE, Lawrie SM. Haloperidol versus placebo for schizophrenia. Cochrane Database Syst Rev. 2001;(2):CD003082. doi: 10.1002/14651858.CD003082. Update in: Cochrane Database Syst Rev. 2006;(4):CD003082. doi: 10.1002/14651858.CD003082.pub2. | Review |
| 21. | Deacon RM, Raley JM, Perry VH, Rawlins JN. Burrowing into prion disease. Neuroreport. 2001;12(9):2053-7. doi: 10.1097/00001756-200107030-00052. | Animal |
| 22. | Shaw J, Claridge G, Clark K. Schizotypy and the shift from dextrality: a study of handedness in a large non-clinical sample. Schizophr Res. 2001;50(3):181-9. doi: 10.1016/s0920-9964(00)00167-5. | Unrelated |
| 23. | Lo D, Hilbush B, Sutcliffe JG. TOGA analysis of gene expression to accelerate target development. Eur J Pharm Sci. 2001;14(3):191-6. doi: 10.1016/s0928-0987(01)00174-9. | Unfocused |

| | |
|---|---|
| 24. Law AJ, Deakin JF. Asymmetrical reductions of hippocampal NMDAR1 glutamate receptor mRNA in the psychoses. Neuroreport. 2001;12(13):2971-4. doi: 10.1097/00001756-200109170-00043. | *Post-mortem* |
| 25. Hille CJ, Fox SH, Maneuf YP, Crossman AR, Brotchie JM. Antiparkinsonian action of a delta opioid agonist in rodent and primate models of Parkinson's disease. Exp Neurol. 2001;172(1):189-98. doi: 10.1006/exnr.2001.7763. | Animal |
| 26. Fenton M, Coutinho ES, Campbell C. Zuclopenthixol acetate in the treatment of acute schizophrenia and similar serious mental illnesses. Cochrane Database Syst Rev. 2001;(3):CD000525. doi: 10.1002/14651858.CD000525. Update in: Cochrane Database Syst Rev. 2004;(3):CD000525. doi: 10.1002/14651858.CD000525.pub2. | Review |
| 27. Telegdy G, Adamik A. The action of orexin A on passive avoidance learning. Involvement of transmitters. Regul Pept. 2002;104(1-3):105-10. doi: 10.1016/s0167-0115(01)00341-x. | Animal |
| 28. Matsuzaki I, Sakurai T, Kunii K, Nakamura T, Yanagisawa M, Goto K. Involvement of the serotonergic system in orexin-induced behavioral alterations in rats. Regul Pept. 2002;104(1-3):119-23. doi: 10.1016/s0167-0115(01)00355-x. | Animal |
| 29. Neuhoff H, Neu A, Liss B, Roeper J. I(h) channels contribute to the different functional properties of identified dopaminergic subpopulations in the midbrain. J Neurosci. 2002;22(4):1290-302. doi: 10.1523/JNEUROSCI.22-04-01290.2002. | Animal |
| **30. Nishino S, Ripley B, Mignot E, Benson KL, Zarcone VP. CSF hypocretin-1 levels in schizophrenics and controls: relationship to sleep architecture. Psychiatry Res. 2002;110(1):1-7. doi: 10.1016/s0165-1781(02)00032-x.** | **Included** |
| 31. Fadel J, Bubser M, Deutch AY. Differential activation of orexin neurons by antipsychotic drugs associated with weight gain. J Neurosci. 2002;22(15):6742-6. doi: 10.1523/JNEUROSCI.22-15-06742.2002. | Animal |
| 32. Katz JD, Ropper AH. Familial Kleine-Levin syndrome: two siblings with unusually long hypersomnic spells. Arch Neurol. 2002;59(12):1959-61. doi: 10.1001/archneur.59.12.1959. | Case |
| 33. Monda M, Viggiano A, De Luca V. Haloperidol reduces the sympathetic and thermogenic activation induced by orexin A. Neurosci Res. 2003;45(1):17-23. doi: 10.1016/s0168-0102(02)00191-8. | Animal |
| 34. Cullen TJ, Walker MA, Parkinson N, Craven R, Crow TJ, Esiri MM, Harrison PJ. A postmortem study of the mediodorsal nucleus of the thalamus in schizophrenia. Schizophr Res. 2003;60(2-3):157-66. doi: 10.1016/s0920-9964(02)00297-9. | *Post-mortem* |
| 35. Douglass AB. Narcolepsy: differential diagnosis or etiology in some cases of bipolar disorder and schizophrenia? CNS Spectr. 2003;8(2):120-6. doi: 10.1017/s1092852900018344. | Review |
| 36. Young WB, Piovesan EJ, Biglan KM. Restless legs syndrome and drug-induced akathisia in headache patients. CNS Spectr. 2003;8(6):450-6. doi: 10.1017/s1092852900018769. | Unfocused |
| 37. Sasa M, Nishi A, Kobayashi K, Sano H, Momiyama T, Uramura K, Yada T, Mori N, Suzuki K, Minabe Y. ドパミンによる運動精神機能調節：新たな研究への展開 [Regulation of psychomotor functions by dopamine: integration of various approaches]. Nihon Yakurigaku Zasshi (Folia Pharmacol Jpn). 2003;122(3):215-25. Japanese. doi: 10.1254/fpj.122.215. | Animal |
| 38. Highley JR, DeLisi LE, Roberts N, Webb JA, Relja M, Razi K, Crow TJ. Sex-dependent effects of schizophrenia: an MRI study of gyral folding, and cortical and white matter volume. Psychiatry Res. 2003;124(1):11-23. doi: 10.1016/s0925-4927(03)00076-3. | No orexin |
| **39. Dalal MA, Schuld A, Pollmächer T. Lower CSF orexin A (hypocretin-1) levels in patients with schizophrenia treated with haloperidol compared to unmedicated subjects. Mol Psychiatry. 2003;8(10):836-7. doi: 10.1038/sj.mp.4001363.** | **Included** |
| 40. Pei Q, Zetterström TS, Sprakes M, Tordera R, Sharp T. Antidepressant drug treatment induces Arc gene expression in the rat brain. Neuroscience. 2003;121(4):975-82. doi: 10.1016/s0306-4522(03)00504-9. | Animal |
| 41. Le Masurier M, Houston G, Cowen P, Grasby P, Sharp T, Hume S. Tyrosine-free amino acid mixture attenuates amphetamine-induced displacement of [$^{11}$C]raclopride in striatum in vivo: a rat PET study. Synapse. 2004;51(2):151-7. doi: 10.1002/syn.10285. | Animal |
| 42. Richardson AJ, Cyhlarova E, Ross MA. Omega-3 and omega-6 fatty acid concentrations in red blood cell membranes relate to schizotypal traits in healthy adults. Prostaglandins Leukot Essent Fatty Acids. 2003;69(6):461-6. doi: 10.1016/j.plefa.2003.08.018. | No orexin |
| 43. Sheitman BB, Knable MB, Jarskog LF, Chakos M, Boyce LH, Early J, Lieberman JA. Secretin for refractory schizophrenia. Schizophr Res. 2004;66(2-3):177-81. doi: 10.1016/S0920-9964(03)00068-9. | Unfocused |
| 44. Benson MA, Sillitoe RV, Blake DJ. Schizophrenia genetics: dysbindin under the microscope. Trends Neurosci. 2004;27(9):516-9. doi: 10.1016/j.tins.2004.06.004. | Review |
| 45. Chance SA, Tzotzoli PM, Vitelli A, Esiri MM, Crow TJ. The cytoarchitecture of sulcal folding in Heschl's sulcus and the temporal cortex in the normal brain and schizophrenia: lamina thickness and cell density. Neurosci Lett. 2004;367(3):384-8. doi: 10.1016/j.neulet.2004.06.041. | No orexin |
| 46. Monda M, Viggiano A, Viggiano A, Fuccio F, De Luca V. Clozapine blocks sympathetic and thermogenic reactions induced by orexin A in rat. Physiol Res. 2004;53(5):507-13. | Animal |
| 47. Cure S, Rathbone J, Carpenter S. Droperidol for acute psychosis. Cochrane Database Syst Rev. 2004;(4):CD002830. doi: 10.1002/14651858.CD002830.pub2. Update in: Cochrane Database Syst Rev. 2016;12:CD002830. doi: 10.1002/14651858.CD002830.pub3. | Review |
| 48. Hardwick C, French SJ, Southam E, Totterdell S. A comparison of possible markers for chandelier cartridges in rat medial prefrontal cortex and hippocampus. Brain Res. 2005;1031(2):238-44. doi: 10.1016/j.brainres.2004.10.047. | Animal |
| 49. Buckingham SD, Pym L, Jones AK, Brown L, Sansom MS, Sattelle DB, Biggin PC. A7DB: a relational database for mutational, physiological and pharmacological data related to the alpha7 nicotinic acetylcholine receptor. BMC Neurosci. 2005;6:2. doi: 10.1186/1471-2202-6-2. | No orexin |
| 50. Chance SA, Esiri MM, Crow TJ. Macroscopic brain asymmetry is changed along the antero-posterior axis in schizophrenia. Schizophr Res. 2005;74(2-3):163-70. doi: 10.1016/j.schres.2004.09.001. | *Post-mortem* |
| 51. Monda M, Viggiano A, Viggiano A, Viggiano E, De Luca V. Risperidone potentiates the sympathetic and hyperthermic reactions induced by orexin A in the rat. Physiol Res. 2006;55(1):73-78. doi: 10.33549/physiolres.930906. Epub 2005 Apr 26. | Animal |
| 52. Denk F, Walton ME, Jennings KA, Sharp T, Rushworth MF, Bannerman DM. Differential involvement of serotonin and dopamine systems in cost-benefit decisions about delay or effort. Psychopharmacology (Berl). 2005;179(3):587-96. doi: 10.1007/s00213-004-2059-4. Epub 2004 Dec 10. | Animal |
| 53. Walterfang M, Upjohn E, Velakoulis D. Is schizophrenia associated with narcolepsy? Cogn Behav Neurol. 2005;18(2):113-8. doi: 10.1097/01.wnn.0000160822.53577.2c. | Case |
| 54. Bubser M, Fadel JR, Jackson LL, Meador-Woodruff JH, Jing D, Deutch AY. Dopaminergic regulation of orexin neurons. Eur J Neurosci. 2005;21(11):2993-3001. doi: 10.1111/j.1460-9568.2005.04121.x. | Animal |
| **55. Meerabux J, Iwayama Y, Sakurai T, Ohba H, Toyota T, Yamada K, Nagata R, Irukayama-Tomobe Y, Shimizu H, Yoshitsugu K, Ohta K, Yoshikawa T. Association of an orexin 1 receptor 408Val variant with polydipsia-hyponatremia in schizophrenic subjects. Biol Psychiatry. 2005;58(5):401-7. doi: 10.1016/j.biopsych.2005.04.015.** | **Included** |
| **56. Cochen V, Arnulf I, Demeret S, Neulat ML, Gourlet V, Drouot X, Moutereau S, Derenne JP, Similowski T, Willer JC, Pierrot-Deseilligny C, Bolgert F. Vivid dreams, hallucinations, psychosis and REM sleep in Guillain-Barré syndrome. Brain. 2005;128(Pt 11):2535-45. doi: 10.1093/brain/awh585. Epub 2005 Jul 6.** | **Included** |
| 57. Rasmussen K, Benvenga MJ, Bymaster FP, Calligaro DO, Cohen IR, Falcone JF, Hemrick-Luecke SK, Martin FM, Moore NA, Nisenbaum LK, Schaus JM, Sundquist SJ, Tupper DE, Wiernicki TR, Nelson DL. Preclinical pharmacology of FMPD [6-fluoro-10-[3-(2-methoxyethyl)-4-methyl-piperazin-1-yl]-2-methyl-4H-3-thia-4,9-diaza-benzo[f]azulene]: a potential novel antipsychotic with lower histamine H1 receptor affinity than olanzapine. J Pharmacol Exp Ther. 2005;315(3):1265-77. doi: 10.1124/jpet.105.089326. Epub 2005 Sep 1. | *In vitro* |

| | |
|---|---|
| 58. Cyhlarova E, Claridge G. Development of a version of the Schizotypy Traits Questionnaire (STA) for screening children. Schizophr Res. 2005;80(2-3):253-61. doi: 10.1016/j.schres.2005.07.037. Epub 2005 Sep 19. | Unrelated |
| 59. Narita M, Nagumo Y, Hashimoto S, Narita M, Khotib J, Miyatake M, Sakurai T, Yanagisawa M, Nakamachi T, Shioda S, Suzuki T. Direct involvement of orexinergic systems in the activation of the mesolimbic dopamine pathway and related behaviors induced by morphine. J Neurosci. 2006;26(2):398-405. doi: 10.1523/JNEUROSCI.2761-05.2006. | Animal |
| 60. Webster JP, Lamberton PH, Donnelly CA, Torrey EF. Parasites as causative agents of human affective disorders? The impact of anti-psychotic, mood- stabilizer and anti-parasite medication on *Toxoplasma gondii*'s ability to alter host behaviour. Proc Biol Sci. 2006;273(1589):1023-30. doi: 10.1098/rspb.2005.3413. | Animal |
| 61. Steffen KJ, Roerig JL, Mitchell JE, Uppala S. Emerging drugs for eating disorder treatment. Expert Opin Emerg Drugs. 2006;11(2):315-36. doi: 10.1517/14728214.11.2.315. | Review |
| 62. Day-Wilson KM, Jones DN, Southam E, Cilia J, Totterdell S. Medial prefrontal cortex volume loss in rats with isolation rearing-induced deficits in prepulse inhibition of acoustic startle. Neuroscience. 2006;141(3):1113-21. doi: 10.1016/j.neuroscience.2006.04.048. Epub 2006 Jun 5. | Animal |
| 63. Kotz CM, Wang C, Teske JA, Thorpe AJ, Novak CM, Kiwaki K, Levine JA. Orexin A mediation of time spent moving in rats: neural mechanisms. Neuroscience. 2006;142(1):29-36. doi: 10.1016/j.neuroscience.2006.05.028. Epub 2006 Jun 30. | Animal |
| 64. Hamlin AS, Blatchford KE, McNally GP. Renewal of an extinguished instrumental response: neural correlates and the role of D1 dopamine receptors. Neuroscience. 2006;143(1):25-38. doi: 10.1016/j.neuroscience.2006.07.035. Epub 2006 Sep 1. | Animal |
| 65. Alberto CO, Trask RB, Quinlan ME, Hirasawa M. Bidirectional dopaminergic modulation of excitatory synaptic transmission in orexin neurons. J Neurosci. 2006;26(39):10043-50. doi: 10.1523/JNEUROSCI.1819-06.2006. **Retraction** in: J Neurosci. 2012;32(26):9116. doi: 10.1523/JNEUROSCI.1889-12.2012. | **Retract** |
| 66. Monda M, Viggiano A, Viggiano A, Viggiano E, Messina G, Tafuri D, De Luca V. Quetiapine lowers sympathetic and hyperthermic reactions due to cerebral injection of orexin A. Neuropeptides. 2006;40(5):357-63. doi: 10.1016/j.npep.2006.07.003. Epub 2006 Sep 28. | Animal |
| 67. Totterdell S. The anatomy of co-morbid neuropsychiatric disorders based on cortico-limbic synaptic interactions. Neurotox Res. 2006;10(2):65-85. doi: 10.1007/BF03033236. | Review |
| 68. Rasmussen K, Hsu MA, Yang Y. The orexin-1 receptor antagonist SB-334867 blocks the effects of antipsychotics on the activity of A9 and A10 dopamine neurons: implications for antipsychotic therapy. Neuropsychopharmacology. 2007;32(4):786-92. doi: 10.1038/sj.npp.1301239. Epub 2006 Oct 25. | Animal |
| 69. Jones AK, Raymond-Delpech V, Thany SH, Gauthier M, Sattelle DB. The nicotinic acetylcholine receptor gene family of the honey bee, *Apis mellifera*. Genome Res. 2006;16(11):1422-30. doi: 10.1101/gr.4549206. Epub 2006 Oct 25. | Animal |
| 70. Monda M, Viggiano A, Viggiano A, Viggiano E, Messina G, Tafuri D, De Luca V. Sympathetic and hyperthermic reactions by orexin A: role of cerebral catecholaminergic neurons. Regul Pept. 2007;139(1-3):39-44. doi: 10.1016/j.regpep.2006.10.002. Epub 2006 Nov 28. | Animal |
| 71. Brundin L, Petersén A, Björkqvist M, Träskman-Bendz L. Orexin and psychiatric symptoms in suicide attempters. J Affect Disord. 2007;100(1-3):259-63. doi: 10.1016/j.jad.2006.10.019. Epub 2006 Dec 4. | Unfocused |
| 72. Brundin L, Björkqvist M, Petersén A, Träskman-Bendz L. Reduced orexin levels in the cerebrospinal fluid of suicidal patients with major depressive disorder. Eur Neuropsychopharmacol. 2007;17(9):573-9. doi: 10.1016/j.euroneuro.2007.01.005. Epub 2007 Mar 7. | No antipsyc |
| 73. Iversen SD, Iversen LL. Dopamine: 50 years in perspective. Trends Neurosci. 2007;30(5):188-93. doi: 10.1016/j.tins.2007.03.002. Epub 2007 Mar 26. | Review |
| 74. Terrar DA, Wilson CM, Graham SG, Bryant SM, Heath BM. Comparison of guinea-pig ventricular myocytes and dog Purkinje fibres for in vitro assessment of drug-induced delayed repolarization. J Pharmacol Toxicol Methods. 2007;56(2):171-85. doi: 10.1016/j.vascn.2007.04.005. Epub 2007 May 26. | Animal |
| 75. Morein-Zamir S, Turner DC, Sahakian BJ. A review of the effects of modafinil on cognition in schizophrenia. Schizophr Bull. 2007;33(6):1298-306. doi: 10.1093/schbul/sbm090. Epub 2007 Jul 18. | Review |
| 76. Lambe EK, Liu RJ, Aghajanian GK. Schizophrenia, hypocretin (orexin), and the thalamocortical activating system. Schizophr Bull. 2007;33(6):1284-90. doi: 10.1093/schbul/sbm088. Epub 2007 Jul 26. | Review |
| 77. Rasmussen K, Hsu MA, Noone S, Johnson BG, Thompson LK, Hemrick-Luecke SK. The orexin-1 antagonist SB-334867 blocks antipsychotic treatment emergent catalepsy: implications for the treatment of extrapyramidal symptoms. Schizophr Bull. 2007;33(6):1291-7. doi: 10.1093/schbul/sbm087. Epub 2007 Jul 28. | Animal |
| 78. Minzenberg MJ, Carter CS. Modafinil: a review of neurochemical actions and effects on cognition. Neuropsychopharmacology. 2008;33(7):1477-502. doi: 10.1038/sj.npp.1301534. Epub 2007 Aug 22. | Review |
| 79. Deutch AY, Bubser M. The orexins/hypocretins and schizophrenia. Schizophr Bull. 2007;33(6):1277-83. doi: 10.1093/schbul/sbm096. Epub 2007 Aug 28. | Review |
| 80. Miskowiak K, Inkster B, O'Sullivan U, Selvaraj S, Goodwin GM, Harmer CJ. Differential effects of erythropoietin on neural and cognitive measures of executive function 3 and 7 days post-administration. Exp Brain Res. 2008;184(3):313-21. doi: 10.1007/s00221-007-1102-1. Epub 2007 Sep 8. | Unrelated |
| **81. Fukunaka Y, Shinkai T, Hwang R, Hori H, Utsunomiya K, Sakata S, Naoe Y, Shimizu K, Matsumoto C, Ohmori O, Nakamura J. The orexin 1 receptor (HCRTR1) gene as a susceptibility gene contributing to polydipsia-hyponatremia in schizophrenia. Neuromolecular Med. 2007;9(4):292-7. doi: 10.1007/s12017-007-8001-2. Epub 2007 Aug 1.** | **Included** |
| 82. McWilliams S, Pennington N, Aziz TZ, Brophy J. Globus pallidus deep brain stimulators for a case of severe neuroleptic-related dystonia and dyskinesia. Ir J Psychol Med. 2007;24(4):159-160. doi: 10.1017/S0790966700010612. | Case |
| 83. Monda M, Viggiano A, Viggiano A, Mondola R, Viggiano E, Messina G, Tafuri D, De Luca V. Olanzapine blocks the sympathetic and hyperthermic reactions due to cerebral injection of orexin A. Peptides. 2008;29(1):120-6. doi: 10.1016/j.peptides.2007.10.016. Epub 2007 Oct 24. | Animal |
| 84. Wallingford NM, Sinnayah P, Bymaster FP, Gadde KM, Krishnan RK, McKinney AA, Landbloom RP, Tollefson GD, Cowley MA. Zonisamide prevents olanzapine-associated hyperphagia, weight gain, and elevated blood glucose in rats. Neuropsychopharmacology. 2008;33(12):2922-33. doi: 10.1038/npp.2008.9. Epub 2008 Mar 5. | Animal |
| 85. Mallet N, Pogosyan A, Sharott A, Csicsvari J, Bolam JP, Brown P, Magill PJ. Disrupted dopamine transmission and the emergence of exaggerated beta oscillations in subthalamic nucleus and cerebral cortex. J Neurosci. 2008;28(18):4795-806. doi: 10.1523/JNEUROSCI.0123-08.2008. | Animal |
| 86. Molnár Z, Hoerder-Suabedissen A, Wang WZ, DeProto J, Davies K, Lee S, Jacobs EC, Campagnoni AT, Paulsen O, Piñon MC, Cheung AF. Genes involved in the formation of the earliest cortical circuits. Novartis Found Symp. 2007;288:212-24; discussion 224-9, 276-81. | Review |
| 87. Bloomfield C, French SJ, Jones DN, Reavill C, Southam E, Cilia J, Totterdell S. Chandelier cartridges in the prefrontal cortex are reduced in isolation reared rats. Synapse. 2008;62(8):628-31. doi: 10.1002/syn.20521. | Animal |
| 88. Janas-Kozik M, Stachowicz M, Mazurek U, Zajdel A, Wilczok A, Krupka-Matuszczyk I, Rybakowski JK. Preliminary study of the expression of genes connected with the orexigenic and anorexigenic system using microarray technique in anorexia nervosa. Neuropsychobiology. 2008;57(3):116-20. doi: 10.1159/000138913. Epub 2008 Jun 13. | Case |
| 89. Lamberton PH, Donnelly CA, Webster JP. Specificity of the *Toxoplasma gondii*-altered behaviour to definitive versus non-definitive host predation risk. Parasitology. 2008;135(10):1143-50. doi: 10.1017/S0031182008004666. Epub 2008 Jul 14. | Animal |

| | |
|---|---|
| 90. Qu WM, Huang ZL, Xu XH, Matsumoto N, Urade Y. Dopaminergic $D_1$ and $D_2$ receptors are essential for the arousal effect of modafinil. J Neurosci. 2008;28(34):8462-9. doi: 10.1523/JNEUROSCI.1819-08.2008. | Animal |
| 91. Davoodi N, Kalinichev M, Korneev SA, Clifton PG. Hyperphagia and increased meal size are responsible for weight gain in rats treated sub-chronically with olanzapine. Psychopharmacology (Berl). 2009;203(4):693-702. doi: 10.1007/s00213-008-1415-1. Epub 2008 Dec 4. | Animal |
| 92. Cope MB, Li X, Jumbo-Lucioni P, DiCostanzo CA, Jamison WG, Kesterson RA, Allison DB, Nagy TR. Risperidone alters food intake, core body temperature, and locomotor activity in mice. Physiol Behav. 2009;96(3):457-63. doi: 10.1016/j.physbeh.2008.11.011. Epub 2008 Nov 27. | Animal |
| 93. Stefanidis A, Verty AN, Allen AM, Owens NC, Cowley MA, Oldfield BJ. The role of thermogenesis in antipsychotic drug-induced weight gain. Obesity (Silver Spring). 2009;17(1):16-24. doi: 10.1038/oby.2008.468. Epub 2008 Oct 30. | Animal |
| 94. Walton ME, Groves J, Jennings KA, Croxson PL, Sharp T, Rushworth MF, Bannerman DM. Comparing the role of the anterior cingulate cortex and 6-hydroxydopamine nucleus accumbens lesions on operant effort-based decision making. Eur J Neurosci. 2009;29(8):1678-91. doi: 10.1111/j.1460-9568.2009.06726.x. | Animal |
| 95. Jänsch C, Harmer C, Cooper MJ. Emotional processing in women with anorexia nervosa and in healthy volunteers. Eat Behav. 2009;10(3):184-91. doi: 10.1016/j.eatbeh.2009.06.001. Epub 2009 Jun 11. | Unrelated |
| 96. Oliver PL, Davies KE. Interaction between environmental and genetic factors modulates schizophrenic endophenotypes in the Snap-25 mouse mutant blind-drunk. Hum Mol Genet. 2009;18(23):4576-89. doi: 10.1093/hmg/ddp425. Epub 2009 Sep 3. | Animal |
| 97. Simons G, Ellgring JH, Beck-Dossler K, Gaebel W, Wölwer W. Facial expression in male and female schizophrenia patients. Eur Arch Psychiatry Clin Neurosci. 2010;260(3):267-76. doi: 10.1007/s00406-009-0074-5. Epub 2009 Oct 9. | No orexin |
| 98. Borgland SL, Labouèbe G. Orexin/hypocretin in psychiatric disorders: present state of knowledge and future potential. Neuropsychopharmacology. 2010;35(1):353-4. doi: 10.1038/npp.2009.119. | Review |
| 99. Suzuki G, Satow A, Ohta H. Effect of CFMTI, an allosteric metabotropic glutamate receptor 1 antagonist with antipsychotic activity, on Fos expression in regions of the brain related to schizophrenia. Neuroscience. 2010;168(3):787-96. doi: 10.1016/j.neuroscience.2010.04.016. Epub 2010 Apr 18. | Animal |
| **100. Basoglu C, Oner O, Gunes C, Semiz UB, Ates AM, Algul A, Ebrinc S, Cetin M, Ozcan O, Ipcioglu O. Plasma orexin A, ghrelin, cholecystokinin, visfatin, leptin and agouti-related protein levels during 6-week olanzapine treatment in first-episode male patients with psychosis. Int Clin Psychopharmacol. 2010;25(3):165-71. doi: 10.1097/YIC.0b013e3283377850.** | **Included** |
| 101. Milella MS, Passarelli F, De Carolis L, Schepisi C, Nativio P, Scaccianoce S, Nencini P. Opposite roles of dopamine and orexin in quinpirole-induced excessive drinking: a rat model of psychotic polydipsia. Psychopharmacology (Berl). 2010;211(3):355-66. doi: 10.1007/s00213-010-1909-5. Epub 2010 Jun 16. | Animal |
| 102. Martins PJ, Haas M, Obici S. Central nervous system delivery of the antipsychotic olanzapine induces hepatic insulin resistance. Diabetes. 2010;59(10):2418-25. doi: 10.2337/db10-0449. Epub 2010 Aug 3. | Animal |
| 103. Mori K, Kim J, Sasaki K. Electrophysiological effects of orexin-B and dopamine on rat nucleus accumbens shell neurons in vitro. Peptides. 2011;32(2):246-52. doi: 10.1016/j.peptides.2010.10.023. Epub 2010 Nov 3. | Animal |
| 104. Burgess CR, Tse G, Gillis L, Peever JH. Dopaminergic regulation of sleep and cataplexy in a murine model of narcolepsy. Sleep. 2010;33(10):1295-304. doi: 10.1093/sleep/33.10.1295. | Animal |
| 105. Cao J, de Lecea L, Ikemoto S. Intraventricular administration of neuropeptide S has reward-like effects. Eur J Pharmacol. 2011;658(1):16-21. doi: 10.1016/j.ejphar.2011.02.009. Epub 2011 Feb 22. | Animal |
| 106. Ito R, Hayen A. Opposing roles of nucleus accumbens core and shell dopamine in the modulation of limbic information processing. J Neurosci. 2011;31(16):6001-7. doi: 10.1523/JNEUROSCI.6588-10.2011. | Animal |
| 107. Sasaki K, Suzuki M, Mieda M, Tsujino N, Roth B, Sakurai T. Pharmacogenetic modulation of orexin neurons alters sleep/wakefulness states in mice. PLoS One. 2011;6(5):e20360. doi: 10.1371/journal.pone.0020360. Epub 2011 May 27. | Animal |
| 108. Fernø J, Varela L, Skrede S, Vázquez MJ, Nogueiras R, Diéguez C, Vidal-Puig A, Steen VM, López M. Olanzapine-induced hyperphagia and weight gain associate with orexigenic hypothalamic neuropeptide signaling without concomitant AMPK phosphorylation. PLoS One. 2011;6(6):e20571. doi: 10.1371/journal.pone.0020571. Epub 2011 Jun 13. | Animal |
| 109. Jeans A, Malins R, Padamsey Z, Reinhart M, Emptage N. Increased expression of dysbindin-1A leads to a selective deficit in NMDA receptor signaling in the hippocampus. Neuropharmacology. 2011;61(8):1345-53. doi: 10.1016/j.neuropharm.2011.08.007. Epub 2011 Aug 16. | Animal |
| 110. Oliver PL, Sobczyk MV, Maywood ES, Edwards B, Lee S, Livieratos A, Oster H, Butler R, Godinho SI, Wulff K, Peirson SN, Fisher SP, Chesham JE, Smith JW, Hastings MH, Davies KE, Foster RG. Disrupted circadian rhythms in a mouse model of schizophrenia. Curr Biol. 2012;22(4):314-9. doi: 10.1016/j.cub.2011.12.051. Epub 2012 Jan 19. | Animal |
| 111. Barkus C, Dawson LA, Sharp T, Bannerman DM. GluN1 hypomorph mice exhibit wide-ranging behavioral alterations. Genes Brain Behav. 2012;11(3):342-51. doi: 10.1111/j.1601-183X.2012.00767.x. Epub 2012 Feb 9. | Animal |
| 112. Ioachimescu OC, El-Solh AA. Pharmacotherapy of insomnia. Expert Opin Pharmacother. 2012;13(9):1243-60. doi: 10.1517/14656566.2012.683860. Epub 2012 May 11. | Review |
| 113. Ma J, Tai SK, Leung LS. Septohippocampal GABAergic neurons mediate the altered behaviors induced by n-methyl-D-aspartate receptor antagonists. Hippocampus. 2012;22(12):2208-18. doi: 10.1002/hipo.22039. Epub 2012 May 17. | Animal |
| 114. Jiang R, Song X, Bali P, Smith A, Bayona CR, Lin L, Cameron MD, McDonald PH, Kenny PJ, Kamenecka TM. Disubstituted piperidines as potent orexin (hypocretin) receptor antagonists. Bioorg Med Chem Lett. 2012;22(12):3890-4. doi: 10.1016/j.bmcl.2012.04.122. Epub 2012 May 4. | *In vitro* |
| 115. Bradshaw CM, Killeen PR. A theory of behaviour on progressive ratio schedules, with applications in behavioural pharmacology. Psychopharmacology (Berl). 2012;222(4):549-64. doi: 10.1007/s00213-012-2771-4. Epub 2012 Jul 3. | Review |
| 116. Vasudevan SR, Moore JB, Schymura Y, Churchill GC. Shape-based reprofiling of FDA-approved drugs for the $H_1$ histamine receptor. J Med Chem. 2012;55(16):7054-60. doi: 10.1021/jm300671m. Epub 2012 Aug 6. | *In vitro* |
| 117. Taslimi Z, Arezoomandan R, Omranifard A, Ghalandari-Shamami M, Riahi E, Vafaei AA, Rashidy-Pour A, Haghparast A. Orexin A in the ventral tegmental area induces conditioned place preference in a dose-dependent manner: involvement of D1/D2 receptors in the nucleus accumbens. Peptides. 2012;37(2):225-32. doi: 10.1016/j.peptides.2012.07.023. Epub 2012 Aug 3. | Animal |
| 118. Ikeda H, Kamei J, Koshikawa N, Cools AR. Nucleus accumbens and dopamine-mediated turning behavior of the rat: role of accumbal non-dopaminergic receptors. J Pharmacol Sci. 2012;120(3):152-64. doi: 10.1254/jphs.12r02cr. Epub 2012 Oct 10. | Animal |
| 119. Hoyer D, Bartfai T. Neuropeptides and neuropeptide receptors: drug targets, and peptide and non-peptide ligands: a tribute to Prof. Dieter Seebach. Chem Biodivers. 2012;9(11):2367-87. doi: 10.1002/cbdv.201200288. | Review |
| 120. Hoerder-Suabedissen A, Oeschger FM, Krishnan ML, Belgard TG, Wang WZ, Lee S, Webber C, Petretto E, Edwards AD, Molnár Z. Expression profiling of mouse subplate reveals a dynamic gene network and disease association with autism and schizophrenia. Proc Natl Acad Sci U S A. 2013;110(9):3555-60. doi: 10.1073/pnas.1218510110. Epub 2013 Feb 11. | Animal |
| 121. Li X, Johnson MS, Smith DL Jr, Li Y, Kesterson RA, Allison DB, Nagy TR. Effects of risperidone on energy balance in female C57BL/6J mice. Obesity (Silver Spring). 2013;21(9):1850-7. doi: 10.1002/oby.20350. Epub 2013 May 29. | Animal |
| 122. LaCrosse AL, Olive MF. Neuropeptide systems and schizophrenia. CNS Neurol Disord Drug Targets. 2013;12(5):619-32. doi: 10.2174/1871527311312050010. | Review |

| | |
|---|---|
| 123. Gozzi A, Lepore S, Vicentini E, Merlo-Pich E, Bifone A. Differential effect of orexin-1 and CRF-1 antagonism on stress circuits: a fMRI study in the rat with the pharmacological stressor Yohimbine. Neuropsychopharmacology. 2013;38(11):2120-30. doi: 10.1038/npp.2013.109. Epub 2013 May 8. | Animal |
| 124. Ortega-Roldan JL, Ossa F, Schnell JR. Characterization of the human sigma-1 receptor chaperone domain structure and binding immunoglobulin protein (BiP) interactions. J Biol Chem. 2013;288(29):21448-21457. doi: 10.1074/jbc.M113.450379. Epub 2013 Jun 12. | *In vitro* |
| 125. Girault EM, Foppen E, Ackermans MT, Fliers E, Kalsbeek A. Central administration of an orexin receptor 1 antagonist prevents the stimulatory effect of Olanzapine on endogenous glucose production. Brain Res. 2013;1527:238-45. doi: 10.1016/j.brainres.2013.06.034. Epub 2013 Jul 4. | Animal |
| 126. Haghparast A, Omranifard A, Arezoomandan R, Ghalandari-Shamami M, Taslimi Z, Vafaei AA, Rashidy-Pour A. Involvement of dopaminergic receptors of the rat nucleus accumbens in decreasing the conditioned place preference induced by lateral hypothalamus stimulation. Neurosci Lett. 2013;556:10-4. doi: 10.1016/j.neulet.2013.09.062. Epub 2013 Oct 5. | Animal |
| 127. Teske JA, Billington CJ, Kotz CM. Mechanisms underlying obesity resistance associated with high spontaneous physical activity. Neuroscience. 2014;256:91-100. doi: 10.1016/j.neuroscience.2013.10.028. Epub 2013 Oct 22. | Animal |
| 128. Chen YW, Morganstern I, Barson JR, Hoebel BG, Leibowitz SF. Differential role of D1 and D2 receptors in the perifornical lateral hypothalamus in controlling ethanol drinking and food intake: possible interaction with local orexin neurons. Alcohol Clin Exp Res. 2014;38(3):777-86. doi: 10.1111/acer.12313. Epub 2013 Nov 15. | Animal |
| **129. Huang YS, Guilleminault C, Chen CH, Lai PC, Hwang FM. Narcolepsy-cataplexy and schizophrenia in adolescents. Sleep Med. 2014;15(1):15-22. doi: 10.1016/j.sleep.2013.09.018. Epub 2013 Oct 26.** | **Included** |
| 130. Brandler WM, Paracchini S. The genetic relationship between handedness and neurodevelopmental disorders. Trends Mol Med. 2014;20(2):83-90. doi: 10.1016/j.molmed.2013.10.008. Epub 2013 Nov 23. | Review |
| 131. Brown R, Taylor MJ, Geddes J. Aripiprazole alone or in combination for acute mania. Cochrane Database Syst Rev. 2013;2013(12):CD005000. doi: 10.1002/14651858.CD005000.pub2. | Review |
| 132. Pizza F, Magnani M, Indrio C, Plazzi G. The hypocretin system and psychiatric disorders. Curr Psychiatry Rep. 2014;16(2):433. doi: 10.1007/s11920-013-0433-9. | Review |
| 133. Hasegawa E, Yanagisawa M, Sakurai T, Mieda M. Orexin neurons suppress narcolepsy via 2 distinct efferent pathways. J Clin Invest. 2014;124(2):604-16. doi: 10.1172/JCI71017. Epub 2014 Jan 2. | Animal |
| 134. Hayen A, Meese-Tamuri S, Gates A, Ito R. Opposing roles of prelimbic and infralimbic dopamine in conditioned cue and place preference. Psychopharmacology (Berl). 2014;231(12):2483-92. doi: 10.1007/s00213-013-3414-0. Epub 2014 Jan 16. | Animal |
| 135. Ekert A, Renner R. The ultimate physical limits of privacy. Nature. 2014;507(7493):443-7. doi: 10.1038/nature13132. | Unrelated |
| 136. Thompson MD, Xhaard H, Sakurai T, Rainero I, Kukkonen JP. OX1 and OX2 orexin/hypocretin receptor pharmacogenetics. Front Neurosci. 2014;8:57. doi: 10.3389/fnins.2014.00057. | Review |
| 137. Palotai M, Telegdy G, Jászberényi M. Orexin A-induced anxiety-like behavior is mediated through GABA-ergic, α- and β-adrenergic neurotransmissions in mice. Peptides. 2014;57:129-34. doi: 10.1016/j.peptides.2014.05.003. Epub 2014 May 27. | Animal |
| 138. Palotai M, Telegdy G, Ekwerike A, Jászberényi M. The action of orexin B on passive avoidance learning. Involvement of neurotransmitters. Behav Brain Res. 2014;272:1-7. doi: 10.1016/j.bbr.2014.06.016. Epub 2014 Jun 13. | Animal |
| 139. Inutsuka A, Inui A, Tabuchi S, Tsunematsu T, Lazarus M, Yamanaka A. Concurrent and robust regulation of feeding behaviors and metabolism by orexin neurons. Neuropharmacology. 2014;85:451-60. doi: 10.1016/j.neuropharm.2014.06.015. Epub 2014 Jun 18. | Animal |
| 140. Kolaj M, Zhang L, Renaud LP. Novel coupling between TRPC-like and KNa channels modulates low threshold spike-induced afterpotentials in rat thalamic midline neurons. Neuropharmacology. 2014;86:88-96. doi: 10.1016/j.neuropharm.2014.06.023. Epub 2014 Jul 9. | Animal |
| 141. Schweimer JV, Coullon GS, Betts JF, Burnet PW, Engle SJ, Brandon NJ, Harrison PJ, Sharp T. Increased burst-firing of ventral tegmental area dopaminergic neurons in D-amino acid oxidase knockout mice in vivo. Eur J Neurosci. 2014;40(7):2999-3009. doi: 10.1111/ejn.12667. Epub 2014 Jul 5. | Animal |
| 142. Yazdi-Ravandi S, Razavi Y, Haghparast A, Goudarzvand M, Haghparast A. Orexin A induced antinociception in the ventral tegmental area involves D1 and D2 receptors in the nucleus accumbens. Pharmacol Biochem Behav. 2014;126:1-6. doi: 10.1016/j.pbb.2014.08.009. Epub 2014 Aug 30. | Animal |
| **143. Chien YL, Liu CM, Shan JC, Lee HJ, Hsieh MH, Hwu HG, Chiou LC. Elevated plasma orexin A levels in a subgroup of patients with schizophrenia associated with fewer negative and disorganized symptoms. Psychoneuroendocrinology. 2015;53:1-9. doi: 10.1016/j.psyneuen.2014.12.012. Epub 2014 Dec 20.** | **Included** |
| 144. Rojczyk E, Pałasz A, Wiaderkiewicz R. Effect of short and long-term treatment with antipsychotics on orexigenic/anorexigenic neuropeptides expression in the rat hypothalamus. Neuropeptides. 2015;51:31-42. doi: 10.1016/j.npep.2015.04.001. Epub 2015 Apr 3. | Animal |
| 145. Pritchett D, Jagannath A, Brown LA, Tam SK, Hasan S, Gatti S, Harrison PJ, Bannerman DM, Foster RG, Peirson SN. Deletion of metabotropic glutamate receptors 2 and 3 (mGlu2 & mGlu3) in mice disrupts sleep and wheel-running activity, and increases the sensitivity of the circadian system to light. PLoS One. 2015;10(5):e0125523. doi: 10.1371/journal.pone.0125523. | Animal |
| 146. Rocca FL, Pizza F, Ricci E, Plazzi G. Narcolepsy during childhood: An update. Neuropediatrics. 2015;46(3):181-98. doi: 10.1055/s-0035-1550152. Epub 2015 May 11. | Review |
| 147. Li AJ, Wang Q, Elsarelli MM, Brown RL, Ritter S. Hindbrain catecholamine neurons activate orexin neurons during systemic glucoprivation in male rats. Endocrinology. 2015;156(8):2807-20. doi: 10.1210/en.2015-1138. Epub 2015 May 15. | Animal |
| 148. Freeman D, Sheaves B, Goodwin GM, Yu LM, Harrison PJ, Emsley R, Bostock S, Foster RG, Wadekar V, Hinds C, Espie CA. Effects of cognitive behavioural therapy for insomnia on the mental health of university students: study protocol for a randomized controlled trial. Trials. 2015;16:236. doi: 10.1186/s13063-015-0756-4. | Protocol |
| 149. Moradi M, Fatahi Z, Haghparast A. Blockade of D1-like dopamine receptors within the ventral tegmental area and nucleus accumbens attenuates antinociceptive responses induced by chemical stimulation of the lateral hypothalamus. Neurosci Lett. 2015;599:61-6. doi: 10.1016/j.neulet.2015.05.047. Epub 2015 May 26. | Animal |
| 150. Ouhaz Z, Ba-M'hamed S, Mitchell AS, Elidrissi A, Bennis M. Behavioral and cognitive changes after early postnatal lesions of the rat mediodorsal thalamus. Behav Brain Res. 2015;292:219-32. doi: 10.1016/j.bbr.2015.06.017. Epub 2015 Jun 12. | Animal |
| 151. Ganzetti M, Wenderoth N, Mantini D. Mapping pathological changes in brain structure by combining T1- and T2-weighted MR imaging data. Neuroradiology. 2015;57(9):917-28. doi: 10.1007/s00234-015-1550-4. Epub 2015 Jun 24. | Unrelated |
| 152. Ma J, Domicevica L, Schnell JR, Biggin PC. Position and orientational preferences of drug-like compounds in lipid membranes: a computational and NMR approach. Phys Chem Chem Phys. 2015;17(30):19766-76. doi: 10.1039/c5cp03218k. | Unrelated |
| 153. Moradi M, Yazdanian M, Haghparast A. Role of dopamine D2-like receptors within the ventral tegmental area and nucleus accumbens in antinociception induced by lateral hypothalamus stimulation. Behav Brain Res. 2015;292:508-14. doi: 10.1016/j.bbr.2015.07.007. Epub 2015 Jul 9. | Animal |
| 154. Jacobs BM. A dangerous method? The use of induced pluripotent stem cells as a model for schizophrenia. Schizophr Res. 2015;168(1-2):563-8. doi: 10.1016/j.schres.2015.07.005. Epub 2015 Jul 17. | Unrelated |
| 155. Majercikova Z, Kiss A. Effect of asenapine on the activity of hypocretin neurons in normal and unpredictable mild stress preconditioned rats. Folia Biol (Praha). 2015;61(3):110-5. doi: 10.14712/fb2015061030110. | Animal |

| | |
|---|---|
| 156. Okumura T, Nozu T, Kumei S, Takakusaki K, Miyagishi S, Ohhira M. Involvement of the dopaminergic system in the central orexin-induced antinociceptive action against colonic distension in conscious rats. Neurosci Lett. 2015;605:34-8. doi: 10.1016/j.neulet.2015.08.013. Epub 2015 Aug 12. | Animal |
| 157. Nishizawa D, Kasai S, Hasegawa J, Sato N, Yamada H, Tanioka F, Nagashima M, Katoh R, Satoh Y, Tagami M, Ujike H, Ozaki N, Inada T, Iwata N, Sora I, Iyo M, Yamada M, Kondo N, Won MJ, Naruse N, Uehara-Aoyama K, Itokawa M, Ohi K, Hashimoto R, Tanisawa K, Arai T, Mori S, Sawabe M, Naka-Mieno M, Yamada Y, Yamada M, Sato N, Muramatsu M, Tanaka M, Irukayama-Tomobe Y, Saito YC, Sakurai T, Hayashida M, Sugimura H, Ikeda K. Associations between the orexin (hypocretin) receptor 2 gene polymorphism Val308Ile and nicotine dependence in genome-wide and subsequent association studies. Mol Brain. 2015;8:50. doi: 10.1186/s13041-015-0142-x. | No psychosis |
| 158. Krystal AD. Current, emerging, and newly available insomnia medications. J Clin Psychiatry. 2015;76(8):e1045. doi: 10.4088/JCP.14046tx2c. | Review |
| 159. Parrott M, Koralus P. The erotetic theory of delusional thinking. Cogn Neuropsychiatry. 2015;20(5):398-415. doi: 10.1080/13546805.2015.1067601. Epub 2015 Sep 14. Erratum in: Cogn Neuropsychiatry. 2015;20(6):555. doi: 10.1080/13546805.2015.1126136. | Unrelated |
| **160. Tiwari AK, Brandl EJ, Zai CC, Goncalves VF, Chowdhury NI, Freeman N, Lieberman JA, Meltzer HY, Kennedy JL, Müller DJ. Association of orexin receptor polymorphisms with antipsychotic-induced weight gain. World J Biol Psychiatry. 2016;17(3):221-9. doi: 10.3109/15622975.2015.1076173. Epub 2015 Oct 8.** | **Included** |
| 161. Barandas R, Landgraf D, McCarthy MJ, Welsh DK. Circadian clocks as modulators of metabolic comorbidity in psychiatric disorders. Curr Psychiatry Rep. 2015;17(12):98. doi: 10.1007/s11920-015-0637-2. | Review |
| 162. Vickers SP, Hackett D, Murray F, Hutson PH, Heal DJ. Effects of lisdexamfetamine in a rat model of binge-eating. J Psychopharmacol. 2015;29(12):1290-307. doi: 10.1177/0269881115615107. Epub 2015 Nov 20. | Animal |
| 163. Boss C, Roch C. Substituted cyclopentanes, tetrahydrofurans and pyrrolidines as orexin-1-receptor antagonists for treatment of various CNS disorders (WO2015/055994; WO2015/124932; WO2015/124934). Expert Opin Ther Pat. 2016;26(3):409-15. doi: 10.1517/13543776.2016.1124087. Epub 2015 Dec 19. | *In vitro* |
| 164. Krystal AD. New developments in insomnia medications of relevance to mental health disorders. Psychiatr Clin North Am. 2015;38(4):843-60. doi: 10.1016/j.psc.2015.08.001. Epub 2015 Sep 11. | Review |
| 165. Hayward A, Tomlinson A, Neill JC. Low attentive and high impulsive rats: A translational animal model of ADHD and disorders of attention and impulse control. Pharmacol Ther. 2016;158:41-51. doi: 10.1016/j.pharmthera.2015.11.010. Epub 2015 Nov 23. | Review |
| **166. Sun HQ, Li SX, Chen FB, Zhang Y, Li P, Jin M, Sun Y, Wang F, Mi WF, Shi L, Yue JL, Yang FD, Lu L. Diurnal neurobiological alterations after exposure to clozapine in first-episode schizophrenia patients. Psychoneuroendocrinology. 2016;64:108-16. doi: 10.1016/j.psyneuen.2015.11.013. Epub 2015 Nov 26.** | **Included** |
| 167. Pritchett D, Taylor AM, Barkus C, Engle SJ, Brandon NJ, Sharp T, Foster RG, Harrison PJ, Peirson SN, Bannerman DM. Searching for cognitive enhancement in the Morris water maze: better and worse performance in D-amino acid oxidase knockout (*Dao*$^{-/-}$) mice. Eur J Neurosci. 2016;43(7):979-89. doi: 10.1111/ejn.13192. Epub 2016 Mar 23. | Animal |
| 168. Okumura T, Nozu T, Kumei S, Takakusaki K, Miyagishi S, Ohhira M. Levodopa acts centrally to induce an antinociceptive action against colonic distension through activation of D2 dopamine receptors and the orexinergic system in the brain in conscious rats. J Pharmacol Sci. 2016;130(2):123-7. doi: 10.1016/j.jphs.2016.01.007. Epub 2016 Jan 29. | Animal |
| 169. Majercikova Z, Kiss A. Stress alters asenapine-induced Fos expression in the Meynert's nucleus: response of adjacent hypocretin and melanin-concentrating hormone neurons in rat. Neurol Res. 2016;38(1):32-9. doi: 10.1080/01616412.2015.1105585. Epub 2016 Feb 19. | Animal |
| 170. Gilmour G, Gastambide F, Marston HM, Walton ME. Using intermediate cognitive endpoints to facilitate translational research in psychosis. Curr Opin Behav Sci. 2015;4:128-135. doi: 10.1016/j.cobeha.2015.04.011. | Review |
| 171. Wagner L, Kaestner F, Wolf R, Stiller H, Heiser U, Manhart S, Hoffmann T, Rahfeld JU, Demuth HU, Rothermundt M, von Hörsten S. Identifying neuropeptide Y (NPY) as the main stress-related substrate of dipeptidyl peptidase 4 (DPP4) in blood circulation. Neuropeptides. 2016;57:21-34. doi: 10.1016/j.npep.2016.02.007. Epub 2016 Feb 27. | No psychosis |
| 172. Sum-Ping O, Guilleminault C. Kleine-Levin Syndrome. Curr Treat Options Neurol. 2016;18(6):24. doi: 10.1007/s11940-016-0409-2. | Review |
| 173. Sheaves B, Bebbington PE, Goodwin GM, Harrison PJ, Espie CA, Foster RG, Freeman D. Insomnia and hallucinations in the general population: Findings from the 2000 and 2007 British Psychiatric Morbidity Surveys. Psychiatry Res. 2016;241:141-6. doi: 10.1016/j.psychres.2016.03.055. Epub 2016 Apr 26. | No orexin |
| 174. Okbay A, Beauchamp JP, Fontana MA, Lee JJ, Pers TH, Rietveld CA, Turley P, Chen GB, Emilsson V, Meddens SF, Oskarsson S, Pickrell JK, Thom K, Timshel P, de Vlaming R, Abdellaoui A, Ahluwalia TS, Bacelis J, Baumbach C, Bjornsdottir G, Brandsma JH, Pina Concas M, Derringer J, Furlotte NA, Galesloot TE, Girotto G, Gupta R, Hall LM, Harris SE, Hofer E, Horikoshi M, Huffman JE, Kaasik K, Kalafati IP, Karlsson R, Kong A, Lahti J, van der Lee SJ, deLeeuw C, Lind PA, Lindgren KO, Liu T, Mangino M, Marten J, Mihailov E, Miller MB, van der Most PJ, Oldmeadow C, Payton A, Pervjakova N, Peyrot WJ, Qian Y, Raitakari O, Rueedi R, Salvi E, Schmidt B, Schraut KE, Shi J, Smith AV, Poot RA, St Pourcain B, Teumer A, Thorleifsson G, Verweij N, Vuckovic D, Wellmann J, Westra HJ, Yang J, Zhao W, Zhu Z, Alizadeh BZ, Amin N, Bakshi A, Baumeister SE, Biino G, Bønnelykke K, Boyle PA, Campbell H, Cappuccio FP, Davies G, De Neve JE, Deloukas P, Demuth I, Ding J, Eibich P, Eisele L, Eklund N, Evans DM, Faul JD, Feitosa MF, Forstner AJ, Gandin I, Gunnarsson B, Halldórsson BV, Harris TB, Heath AC, Hocking LJ, Holliday EG, Homuth G, Horan MA, Hottenga JJ, de Jager PL, Joshi PK, Jugessur A, Kaakinen MA, Kähönen M, Kanoni S, Keltigangas-Järvinen L, Kiemeney LA, Kolcic I, Koskinen S, Kraja AT, Kroh M, Kutalik Z, Latvala A, Launer LJ, Lebreton MP, Levinson DF, Lichtenstein P, Lichtner P, Liewald DC; LifeLines Cohort Study; Loukola A, Madden PA, Mägi R, Mäki-Opas T, Marioni RE, Marques-Vidal P, Meddens GA, McMahon G, Meisinger C, Meitinger T, Milaneschi Y, Milani L, Montgomery GW, Myhre R, Nelson CP, Nyholt DR, Ollier WE, Palotie A, Paternoster L, Pedersen NL, Petrovic KE, Porteous DJ, Räikkönen K, Ring SM, Robino A, Rostapshova O, Rudan I, Rustichini A, Salomaa V, Sanders AR, Sarin AP, Schmidt H, Scott RJ, Smith BH, Smith JA, Staessen JA, Steinhagen-Thiessen E, Strauch K, Terracciano A, Tobin MD, Ulivi S, Vaccargiu S, Quaye L, van Rooij FJ, Venturini C, Vinkhuyzen AA, Völker U, Völzke H, Vonk JM, Vozzi D, Waage J, Ware EB, Willemsen G, Attia JR, Bennett DA, Berger K, Bertram L, Bisgaard H, Boomsma DI, Borecki IB, Bültmann U, Chabris CF, Cucca F, Cusi D, Deary IJ, Dedoussis GV, van Duijn CM, Eriksson JG, Franke B, Franke L, Gasparini P, Gejman PV, Gieger C, Grabe HJ, Gratten J, Groenen PJ, Gudnason V, van der Harst P, Hayward C, Hinds DA, Hoffmann W, Hyppönen E, Iacono WG, Jacobsson B, Järvelin MR, Jöckel KH, Kaprio J, Kardia SL, Lehtimäki T, Lehrer SF, Magnusson PK, Martin NG, McGue M, Metspalu A, Pendleton N, Penninx BW, Perola M, Pirastu N, Pirastu M, Polasek O, Posthuma D, Power C, Province MA, Samani NJ, Schlessinger D, Schmidt R, Sørensen TI, Spector TD, Stefansson K, Thorsteinsdottir U, Thurik AR, Timpson NJ, Tiemeier H, Tung JY, Uitterlinden AG, Vitart V, Vollenweider P, Weir DR, Wilson JF, Wright AF, Conley DC, Krueger RF, Davey Smith G, Hofman A, Laibson DI, Medland SE, Meyer MN, Yang J, Johannesson M, Visscher PM, Esko T, Koellinger PD, Cesarini D, Benjamin DJ. Genome-wide association study identifies 74 loci associated with educational attainment. Nature. 2016;533(7604):539-42. doi: 10.1038/nature17671. Epub 2016 May 11. | No orexin |
| **175. Sansa G, Gavaldà A, Gaig C, Monreal J, Ercilla G, Casamitjana R, Ribera G, Iranzo A, Santamaria J. Exploring the presence of narcolepsy in patients with schizophrenia. BMC Psychiatry. 2016;16:177. doi: 10.1186/s12888-016-0859-9.** | **Included** |
| 176. Xue Y, Yang YT, Liu HY, Chen WF, Chen AQ, Sheng Q, Chen XY, Wang Y, Chen H, Liu HX, Pang YY, Chen L. Orexin-A increases the activity of globus pallidus neurons in both normal and parkinsonian rats. Eur J Neurosci. 2016;44(5):2247-57. doi: 10.1111/ejn.13323. Epub 2016 Jul 13. | Animal |

| | |
|---|---|
| 177. Bakken TE, Miller JA, Ding SL, Sunkin SM, Smith KA, Ng L, Szafer A, Dalley RA, Royall JJ, Lemon T, Shapouri S, Aiona K, Arnold J, Bennett JL, Bertagnolli D, Bickley K, Boe A, Brouner K, Butler S, Byrnes E, Caldejon S, Carey A, Cate S, Chapin M, Chen J, Dee N, Desta T, Dolbeare TA, Dotson N, Ebbert A, Fulfs E, Gee G, Gilbert TL, Goldy J, Gourley L, Gregor B, Gu G, Hall J, Haradon Z, Haynor DR, Hejazinia N, Hoerder-Suabedissen A, Howard R, Jochim J, Kinnunen M, Kriedberg A, Kuan CL, Lau C, Lee CK, Lee F, Luong L, Mastan N, May R, Melchor J, Mosqueda N, Mott E, Ngo K, Nyhus J, Oldre A, Olson E, Parente J, Parker PD, Parry S, Pendergraft J, Potekhina L, Reding M, Riley ZL, Roberts T, Rogers B, Roll K, Rosen D, Sandman D, Sarreal M, Shapovalova N, Shi S, Sjoquist N, Sodt AJ, Townsend R, Velasquez L, Wagley U, Wakeman WB, White C, Bennett C, Wu J, Young R, Youngstrom BL, Wohnoutka P, Gibbs RA, Rogers J, Hohmann JG, Hawrylycz MJ, Hevner RF, Molnár Z, Phillips JW, Dang C, Jones AR, Amaral DG, Bernard A, Lein ES. A comprehensive transcriptional map of primate brain development. Nature. 2016;535(7612):367-75. doi: 10.1038/nature18637. Epub 2016 Jul 13. | Animal |
| 178. Sharpee TO, Destexhe A, Kawato M, Sekulić V, Skinner FK, Wójcik DK, Chintaluri C, Cserpán D, Somogyvári Z, Kim JK, Kilpatrick ZP, Bennett MR, Josić K, Elices I, Arroyo D, Levi R, Rodriguez FB, Varona P, Hwang E, Kim B, Han HB, Kim T, McKenna JT, Brown RE, McCarley RW, Choi JH, Rankin J, Popp PO, Rinzel J, Tabas A, Rupp A, Balaguer-Ballester E, Maturana MI, Grayden DB, Cloherty SL, Kameneva T, Ibbotson MR, Meffin H, Koren V, Lochmann T, Dragoi V, Obermayer K, Psarrou M, Schilstra M, Davey N, Torben-Nielsen B, Steuber V, Ju H, Yu J, Hines ML, Chen L, Yu Y, Kim J, Leahy W, Shlizerman E, Birgiolas J, Gerkin RC, Crook SM, Viriyopase A, Memmesheimer RM, Gielen S, Dabaghian Y, DeVito J, Perotti L, Kim AJ, Fenk LM, Cheng C, Maimon G, Zhao C, Widmer Y, Sprecher S, Senn W, Halnes G, Mäki-Marttunen T, Keller D, Pettersen KH, Andreassen OA, Einevoll GT, Yamada Y, Steyn-Ross ML, Alistair Steyn-Ross D, Mejias JF, Murray JD, Kennedy H, Wang XJ, Kruscha A, Grewe J, Benda J, Lindner B, Badel L, Ohta K, Tsuchimoto Y, Kazama H, Kahng B, Tam ND, Pollonini L, Zouridakis G, Soh J, Kim D, Yoo M, Palmer SE, Culmone V, Bojak I, Ferrario A, Merrison-Hort R, Borisyuk R, Kim CS, Tezuka T, Joo P, Rho YA, Burton SD, Bard Ermentrout G, Jeong J, Urban NN, Marsalek P, Kim HH, Moon SH, Lee DW, Lee SB, Lee JY, Molkov YI, Hamade K, Teka W, Barnett WH, Kim T, Markin S, Rybak IA, Forro C, Dermutz H, Demkó L, Vörös J, Babichev A, Huang H, Verduzco-Flores S, Dos Santos F, Andras P, Metzner C, Schweikard A, Zurowski B, Roach JP, Sander LM, Zochowski MR, Skilling QM, Ognjanovski N, Aton SJ, Zochowski M, Wang SJ, Ouyang G, Guang J, Zhang M, Michael Wong KY, Zhou C, Robinson PA, Sanz-Leon P, Drysdale PM, Fung F, Abeysuriya RG, Rennie CJ, Zhao X, Choe Y, Yang HF, Mi Y, Lin X, Wu S, Liedtke J, Schottdorf M, Wolf F, Yamamura Y, Wickens JR, Rumbell T, Ramsey J, Reyes A, Draguljić D, Hof PR, Luebke J, Weaver CM, He H, Yang X, Ma H, Xu Z, Wang Y, Baek K, Morris LS, Kundu P, Voon V, Agnes EJ, Vogels TP, Podlaski WF, Giese M, Kuravi P, Vogels R, Seeholzer A, Podlaski W, Ranjan R, Vogels T, Torres JJ, Baroni F, Latorre R, Gips B, Lowet E, Roberts MJ, de Weerd P, Jensen O, van der Eerden J, Goodarzinick A, Niry MD, Valizadeh A, Pariz A, Parsi SS, Warburton JM, Marucci L, Tamagnini F, Brown J, Tsaneva-Atanasova K, Kleberg FI, Triesch J, Moezzi B, Iannella N, Schaworonkow N, Plogmacher L, Goldsworthy MR, Hordacre B, McDonnell MD, Ridding MC, Zapotocky M, Smit D, Fouquet C, Trembleau A, Dasgupta S, Nishikawa I, Aihara K, Toyoizumi T, Robb DT, Mellen N, Toporikova N, Tang R, Tang YY, Liang G, Kiser SA, Howard JH Jr, Goncharenko J, Voronenko SO, Ahamed T, Stephens G, Yger P, Lefebvre B, Spampinato GLB, Esposito E, et Olivier Marre MS, Choi H, Song MH, Chung S, Lee DD, Sompolinsky H, Phillips RS, Smith J, Chatzikalymniou AP, Ferguson K, Alex Cayco Gajic N, Clopath C, Angus Silver R, Gleeson P, Marin B, Sadeh S, Quintana A, Cantarelli M, Dura-Bernal S, Lytton WW, Davison A, Li L, Zhang W, Wang D, Song Y, Park S, Choi I, Shin HS, Choi H, Pasupathy A, Shea-Brown E, Huh D, Sejnowski TJ, Vogt SM, Kumar A, Schmidt R, Van Wert S, Schiff SJ, Veale R, Scheutz M, Lee SW, Gallinaro J, Rotter S, Rubchinsky LL, Cheung CC, Ratnadurai-Giridharan S, Shomali SR, Ahmadabadi MN, Shimazaki H, Nader Rasuli S, Zhao X, Rasch MJ, Wilting J, Priesemann V, Levina A, Rudelt L, Lizier JT, Spinney RE, Rubinov M, Wibral M, Bak JH, Pillow J, Zaho Y, Park IM, Kang J, Park HJ, Jang J, Paik SB, Choi W, Lee C, Song M, Lee H, Park Y, Yilmaz E, Baysal V, Ozer M, Saska D, Nowotny T, Chan HK, Diamond A, Herrmann CS, Murray MM, Ionta S, Hutt A, Lefebvre J, Weidel P, Duarte R, Morrison A, Lee JH, Iyer R, Mihalas S, Koch C, Petrovici MA, Leng L, Breitwieser O, Stöckel D, Bytschok I, Martel R, Bill J, Schemmel J, Meier K, Esler TB, Burkitt AN, Kerr RR, Tahayori B, Nolte M, Reimann MW, Muller E, Markram H, Parziale A, Senatore R, Marcelli A, Skiker K, Maouene M, Neymotin SA, Seidenstein A, Lakatos P, Sanger TD, Menzies RJ, McLauchlan C, van Albada SJ, Kedziora DJ, Neymotin S, Kerr CC, Suter BA, Shepherd GMG, Ryu J, Lee SH, Lee J, Lee HJ, Lim D, Wang J, Lee H, Jung N, Anh Quang L, Maeng SE, Lee TH, Lee JW, Park CH, Ahn S, Moon J, Choi YS, Kim J, Jun SB, Lee S, Lee HW, Jo S, Jun E, Yu S, Goetze F, Lai PY, Kim S, Kwag J, Jang HJ, Filipović M, Reig R, Aertsen A, Silberberg G, Bachmann C, Buttler S, Jacobs H, Dillen K, Fink GR, Kukolja J, Kepple D, Giaffar H, Rinberg D, Shea S, Koulakov A, Bahuguna J, Tetzlaff T, Kotaleski JH, Kunze T, Peterson A, Knösche T, Kim M, Kim H, Park JS, Yeon JW, Kim SP, Kang JH, Lee C, Spiegler A, Petkoski S, Palva MJ, Jirsa VK, Saggio ML, Siep SF, Stacey WC, Bernar C, Choung OH, Jeong Y, Lee YI, Kim SH, Jeong M, Lee J, Kwon J, Kralik JD, Jahng J, Hwang DU, Kwon JH, Park SM, Kim S, Kim H, Kim PS, Yoon S, Lim S, Park C, Miller T, Clements K, Ahn S, Ji EH, Issa FA, Baek J, Oba S, Yoshimoto J, Doya K, Ishii S, Mosqueiro TS, Strube- Bloss MF, Smith B, Huerta R, Hadrava M, Hlinka J, Bos H, Helias M, Welzig CM, Harper ZJ, Kim WS, Shin IS, Baek HM, Han SK, Richter R, Vitay J, Beuth F, Hamker FH, Toppin K, Guo Y, Graham BP, Kale PJ, Gollo LL, Stern M, Abbott LF, Fedorov LA, Giese MA, Ardestani MH, Faraji MJ, Preuschoff K, Gerstner W, van Gendt MJ, Briaire JJ, Kalkman RK, Frijns JHM, Lee WH, Frangou S, Fulcher BD, Tran PHP, Fornito A, Gliske SV, Lim E, Holman KA, Fink CG, Kim JS, Mu S, Briggman KL, Sebastian Seung H; the EyeWirers; Wegener D, Bohnenkamp L, Ernst UA, Devor A, Dale AM, Lines GT, Edwards A, Tveito A, Hagen E, Senk J, Diesmann M, Schmidt M, Bakker R, Shen K, Bezgin G, Hilgetag CC, van Albada SJ, Sun H, Sourina O, Huang GB, Klanner F, Denk C, Glomb K, Ponce-Alvarez A, Gilson M, Ritter P, Deco G, Witek MAG, Clarke EF, Hansen M, Wallentin M, Kringelbach ML, Vuust P, Klingbeil G, De Schutter E, Chen W, Zang Y, Hong S, Takashima A, Zamora C, Gallimore AR, Goldschmidt D, Manoonpong P, Karoly PJ, Freestone DR, Soundry D, Kuhlmann L, Paninski L, Cook M, Lee J, Fishman YI, Cohen YE, Roberts JA, Cocchi L, Sweeney Y, Lee S, Jung WS, Kim Y, Jung Y, Song YK, Chavane F, Soman K, Muralidharan V, Srinivasa Chakravarthy V, Shivkumar S, Mandali A, Pragathi Priyadharsini B, Mehta H, Davey CE, Brinkman BAW, Kekona T, Rieke F, Buice M, De Pittà M, Berry H, Brunel N, Breakspear M, Marsat G, Drew J, Chapman PD, Daly KC, Bradle SP, Seo SB, Su J, Kavalali ET, Blackwell J, Shiau L, Buhry L, Basnayake K, Lee SH, Levy BA, Baker CI, Leleu T, Philips RT, Chhabria K. 25th Annual Computational Neuroscience Meeting: CNS-2016. BMC Neurosci. 2016;17 Suppl 1(Suppl 1):54. doi: 10.1186/s12868-016-0283-6. | Abstr |
| 179. Sarkanen T, Alén R, Partinen M. Transient impact of rituximab in H1N1 vaccination-associated narcolepsy with severe psychiatric symptoms. Neurologist. 2016;21(5):85-6. doi: 10.1097/NRL.0000000000000099. | Case |
| 180. Astle WJ, Elding H, Jiang T, Allen D, Ruklisa D, Mann AL, Mead D, Bouman H, Riveros-Mckay F, Kostadima MA, Lambourne JJ, Sivapalaratnam S, Downes K, Kundu K, Bomba L, Berentsen K, Bradley JR, Daugherty LC, Delaneau O, Freson K, Garner SF, Grassi L, Guerrero J, Haimel M, Janssen-Megens EM, Kaan A, Kamat M, Kim B, Mandoli A, Marchini J, Martens JHA, Meacham S, Megy K, O'Connell J, Petersen R, Sharifi N, Sheard SM, Staley JR, Tuna S, van der Ent M, Walter K, Wang SY, Wheeler E, Wilder SP, Iotchkova V, Moore C, Sambrook J, Stunnenberg HG, Di Angelantonio E, Kaptoge S, Kuijpers TW, Carrillo-de-Santa-Pau E, Juan D, Rico D, Valencia A, Chen L, Ge B, Vasquez L, Kwan T, Garrido-Martín D, Watt S, Yang Y, Guigo R, Beck S, Paul DS, Pastinen T, Bujold D, Bourque G, Frontini M, Danesh J, Roberts DJ, Ouwehand WH, Butterworth AS, Soranzo N. The allelic landscape of human blood cell trait variation and links to common complex disease. Cell. 2016;167(5):1415-1429.e19. doi: 10.1016/j.cell.2016.10.042. | No orexin |
| 181. Boerner T, Bygrave AM, Chen J, Fernando A, Jackson S, Barkus C, Sprengel R, Seeburg PH, Harrison PJ, Gilmour G, Bannerman DM, Sanderson DJ. The group II metabotropic glutamate receptor agonist LY354740 and the D2 receptor antagonist haloperidol reduce locomotor hyperactivity but fail to rescue spatial working memory in GluA1 knockout mice. Eur J Neurosci. 2017;45(7):912-921. doi: 10.1111/ejn.13539. Epub 2017 Mar 4. | Animal |
| 182. Mahoney CE, Agostinelli LJ, Brooks JN, Lowell BB, Scammell TE. GABAergic neurons of the central amygdala promote cataplexy. J Neurosci. 2017;37(15):3995-4006. doi: 10.1523/JNEUROSCI.4065-15.2017. Epub 2017 Feb 24. | Animal |
| 183. Webber C. Epistasis in neuropsychiatric disorders. Trends Genet. 2017;33(4):256-265. doi: 10.1016/j.tig.2017.01.009. Epub 2017 Mar 6. | Review |

| | |
|---|---|
| 184. Bunney PE, Zink AN, Holm AA, Billington CJ, Kotz CM. Orexin activation counteracts decreases in nonexercise activity thermogenesis (NEAT) caused by high-fat diet. Physiol Behav. 2017;176:139-148. doi: 10.1016/j.physbeh.2017.03.040. Epub 2017 Mar 28. | Animal |
| 185. Giannoccaro MP, Waters P, Pizza F, Liguori R, Plazzi G, Vincent A. Antibodies against hypocretin receptor 2 are rare in narcolepsy. Sleep. 2017;40(2):zsw056. doi: 10.1093/sleep/zsw056. | No antipsyc |
| 186. Sanderson DJ, Lee A, Sprengel R, Seeburg PH, Harrison PJ, Bannerman DM. Altered balance of excitatory and inhibitory learning in a genetically modified mouse model of glutamatergic dysfunction relevant to schizophrenia. Sci Rep. 2017;7(1):1765. doi: 10.1038/s41598-017-01925-8. | Animal |
| 187. Barron HC, Vogels TP, Behrens TE, Ramaswami M. Inhibitory engrams in perception and memory. Proc Natl Acad Sci U S A. 2017;114(26):6666-6674. doi: 10.1073/pnas.1701812114. Epub 2017 Jun 13. | Opinion |
| 188. Matheson E, Hainer BL. Insomnia: Pharmacologic therapy. Am Fam Physician. 2017;96(1):29-35. | Review |
| 189. Cañellas-Dols F, Delgado C, Arango-Lopez C, Peraita-Adrados R. Narcolepsy-cataplexy and psychosis: a case study. Rev Neurol. 2017;65(2):70-74. Spanish, English. | Case |
| 190. Campbell EJ, Mitchell CS, Adams CD, Yeoh JW, Hodgson DM, Graham BA, Dayas CV. Chemogenetic activation of the lateral hypothalamus reverses early life stress-induced deficits in motivational drive. Eur J Neurosci. 2017;46(7):2285-2296. doi: 10.1111/ejn.13674. Epub 2017 Sep 22. | Animal |
| 191. Thompson MD, Sakurai T, Rainero I, Maj MC, Kukkonen JP. Orexin receptor multimerization versus functional interactions: Neuropharmacological implications for opioid and cannabinoid signalling and pharmacogenetics. Pharmaceuticals (Basel). 2017;10(4):79. doi: 10.3390/ph10040079. | Review |
| 192. Keks NA, Hope J, Keogh S. Suvorexant: scientifically interesting, utility uncertain. Australas Psychiatry. 2017;25(6):622-624. doi: 10.1177/1039856217734677. Epub 2017 Oct 10. | Opinion |
| 193. Alderson-Day B, Lima CF, Evans S, Krishnan S, Shanmugalingam P, Fernyhough C, Scott SK. Distinct processing of ambiguous speech in people with non-clinical auditory verbal hallucinations. Brain. 2017;140(9):2475-2489. doi: 10.1093/brain/awx206. | No orexin |
| 194. Sheaves B, Freeman D, Isham L, McInerney J, Nickless A, Yu LM, Rek S, Bradley J, Reeve S, Attard C, Espie CA, Foster R, Wirz-Justice A, Chadwick E, Barrera A. Stabilising sleep for patients admitted at acute crisis to a psychiatric hospital (OWLS): an assessor-blind pilot randomised controlled trial. Psychol Med. 2018;48(10):1694-1704. doi: 10.1017/S0033291717003191. Epub 2017 Nov 7. | No orexin |
| 195. Zink AN, Bunney PE, Holm AA, Billington CJ, Kotz CM. Neuromodulation of orexin neurons reduces diet-induced adiposity. Int J Obes (Lond). 2018;42(4):737-745. doi: 10.1038/ijo.2017.276. Epub 2017 Nov 28. | Animal |
| 196. Öz P, Gökalp HK, Göver T, Uzbay T. Dose-dependent and opposite effects of orexin A on prepulse inhibition response in sleep-deprived and non-sleep- deprived rats. Behav Brain Res. 2018;346:73-79. doi: 10.1016/j.bbr.2017.12.002. Epub 2017 Dec 10. | Animal |
| 197. Vergunst F, Jenkinson C, Burns T, Anand P, Gray A, Rugkåsa J, Simon J. Psychometric validation of a multi-dimensional capability instrument for outcome measurement in mental health research (OxCAP-MH). Health Qual Life Outcomes. 2017;15(1):250. doi: 10.1186/s12955-017-0825-3. | No orexin |
| 198. Ross RM, McKay R. Shamanism and the psychosis continuum. Behav Brain Sci. 2018;41:e84. doi: 10.1017/S0140525X17002151. | Opinion |
| 199. Monda V, Salerno M, Sessa F, Bernardini R, Valenzano A, Marsala G, Zammit C, Avola R, Carotenuto M, Messina G, Messina A. Functional changes of orexinergic reaction to psychoactive substances. Mol Neurobiol. 2018;55(8):6362-6368. doi: 10.1007/s12035-017-0865-z. Epub 2018 Jan 6. | Animal |
| 200. Vann Jones S, Banerjee S, Smith AD, Refsum H, Lennox B. Elevated homocysteine and *N*-methyl-D-aspartate-receptor antibodies as a cause of behavioural and cognitive decline in 22q11.2 deletion syndrome. Oxf Med Case Reports. 2017;2017(12):omx076. doi: 10.1093/omcr/omx076. | Case |
| 201. Green AR, Haddad PM, Aronson JK. Marketing medicines: charting the rise of modern therapeutics through a systematic review of adverts in UK medical journals (1950-1980). Br J Clin Pharmacol. 2018;84(8):1668-1685. doi: 10.1111/bcp.13549. Epub 2018 Mar 25. | Review |
| 202. Barton J, Kyle SD, Varese F, Jones SH, Haddock G. Are sleep disturbances causally linked to the presence and severity of psychotic-like, dissociative and hypomanic experiences in non-clinical populations? A systematic review. Neurosci Biobehav Rev. 2018;89:119-131. doi: 10.1016/j.neubiorev.2018.02.008. Epub 2018 Feb 13. | Review |
| 203. Okumura T, Nozu T, Kumei S, Takakusaki K, Ohhira M. Ghrelin acts centrally to induce an antinociceptive action during colonic distension through the orexinergic, dopaminergic and opioid systems in conscious rats. Brain Res. 2018;1686:48-54. doi: 10.1016/j.brainres.2018.02.024. Epub 2018 Feb 21. | Animal |
| 204. Atkin T, Comai S, Gobbi G. Drugs for insomnia beyond benzodiazepines: Pharmacology, clinical applications, and discovery. Pharmacol Rev. 2018;70(2):197-245. doi: 10.1124/pr.117.014381. | Review |
| 205. Kao AC, Spitzer S, Anthony DC, Lennox B, Burnet PWJ. Prebiotic attenuation of olanzapine-induced weight gain in rats: analysis of central and peripheral biomarkers and gut microbiota. Transl Psychiatry. 2018;8(1):66. doi: 10.1038/s41398-018-0116-8. | Animal |
| 206. Wykes T, Joyce E, Velikonja T, Watson A, Aarons G, Birchwood M, Cella M, Dopson S, Fowler D, Greenwood K, Johnson S, McCrone P, Perez J, Pickles A, Reeder C, Rose D, Singh S, Stringer D, Taylor M, Taylor R, Upthegrove R. The CIRCuiTS study (Implementation of cognitive remediation in early intervention services): protocol for a randomised controlled trial. Trials. 2018;19(1):183. doi: 10.1186/s13063-018-2553-3. | Protocol |
| 207. Luo YJ, Li YD, Wang L, Yang SR, Yuan XS, Wang J, Cherasse Y, Lazarus M, Chen JF, Qu WM, Huang ZL. Nucleus accumbens controls wakefulness by a subpopulation of neurons expressing dopamine $D_1$ receptors. Nat Commun. 2018;9(1):1576. doi: 10.1038/s41467-018-03889-3. | Animal |
| 208. Risco S, Mediavilla C. Orexin A in the ventral tegmental area enhances saccharin-induced conditioned flavor preference: The role of D1 receptors in central nucleus of amygdala. Behav Brain Res. 2018;348:192-200. doi: 10.1016/j.bbr.2018.04.010. Epub 2018 Apr 21. | Animal |
| 209. Kotzadimitriou D, Nissen W, Paizs M, Newton K, Harrison PJ, Paulsen O, Lamsa K. Neuregulin 1 type I overexpression is associated with reduced NMDA receptor-mediated synaptic signaling in hippocampal interneurons expressing PV or CCK. eNeuro. 2018;5(2):ENEURO.0418-17.2018. doi: 10.1523/ENEURO.0418-17.2018. | Animal |
| 210. Dujardin S, Pijpers A, Pevernagie D. Prescription drugs used in insomnia. Sleep Med Clin. 2018;13(2):169-182. doi: 10.1016/j.jsmc.2018.03.001. | Review |
| 211. Bolton JL, Ruiz CM, Rismanchi N, Sanchez GA, Castillo E, Huang J, Cross C, Baram TZ, Mahler SV. Early-life adversity facilitates acquisition of cocaine self-administration and induces persistent anhedonia. Neurobiol Stress. 2018;8:57-67. doi: 10.1016/j.ynstr.2018.01.002. | Animal |
| 212. Isham L, Grafahrend H, Nickless A, Pugh K, Pleasants S, Smedley N, Freeman D, Mulligan A. Group-based worry intervention for persecutory delusions: an initial feasibility study. Behav Cogn Psychother. 2018;46(5):619-625. doi: 10.1017/S1352465818000383. Epub 2018 Jun 20. | Unrelated |
| 213. Miskoff JA, Chaudhri M. Off-label sodium oxybate in childhood narcolepsy: A comprehensive report. Cureus. 2018;10(4):e2526. doi: 10.7759/cureus.2526. | Case |
| 214. Juvodden HT, Alnæs D, Lund MJ, Agartz I, Andreassen OA, Dietrichs E, Thorsby PM, Westlye LT, Knudsen S. Widespread white matter changes in post-H1N1 patients with narcolepsy type 1 and first-degree relatives. Sleep. 2018;41(10):zsy145. doi: 10.1093/sleep/zsy145. | No psychosis |
| 215. Bobrovitz N, Heneghan C, Onakpoya I, Fletcher B, Collins D, Tompson A, Lee J, Nunan D, Fisher R, Scott B, O'Sullivan J, Van Hecke O, Nicholson BD, Stevens S, Roberts N, Mahtani KR. Medications that reduce emergency hospital admissions: an overview of systematic reviews and prioritisation of treatments. BMC Med. 2018;16(1):115. doi: 10.1186/s12916-018-1104-9. | Review |

| | |
|---|---|
| 216. Palman AD. Сон и его нарушения при хронической обструктивной болезни легких [Sleep and its' disturbanses in chronic obstructive pulmonary disease]. Zh Nevrol Psikhiatr Im S S Korsakova. 2018;118(4. Vyp. 2):113-118. Russian. doi: 10.17116/jnevro201811842113. | Review |
| 217. Liu C, Xue Y, Liu MF, Wang Y, Liu ZR, Diao HL, Chen L. Orexins increase the firing activity of nigral dopaminergic neurons and participate in motor control in rats. J Neurochem. 2018;147(3):380-394. doi: 10.1111/jnc.14568. Epub 2018 Oct 15. | Animal |
| 218. López-Jury L, Meza RC, Brown MTC, Henny P, Canavier CC. Morphological and biophysical determinants of the intracellular and extracellular waveforms in nigral dopaminergic neurons: A computational study. J Neurosci. 2018;38(38):8295-8310. doi: 10.1523/JNEUROSCI.0651-18.2018. Epub 2018 Aug 13. | *In vitro* |
| 219. Ang G, McKillop LE, Purple R, Blanco-Duque C, Peirson SN, Foster RG, Harrison PJ, Sprengel R, Davies KE, Oliver PL, Bannerman DM, Vyazovskiy VV. Absent sleep EEG spindle activity in GluA1 (Gria1) knockout mice: relevance to neuropsychiatric disorders. Transl Psychiatry. 2018;8(1):154. doi: 10.1038/s41398-018-0199-2. | Animal |
| 220. Naganuma F, Bandaru SS, Absi G, Mahoney CE, Scammell TE, Vetrivelan R. Melanin-concentrating hormone neurons contribute to dysregulation of rapid eye movement sleep in narcolepsy. Neurobiol Dis. 2018;120:12-20. doi: 10.1016/j.nbd.2018.08.012. Epub 2018 Aug 24. | Animal |
| 221. Frase L, Nissen C, Riemann D, Spiegelhalder K. Making sleep easier: pharmacological interventions for insomnia. Expert Opin Pharmacother. 2018;19(13):1465-1473. doi: 10.1080/14656566.2018.1511705. Epub 2018 Sep 3. | Review |
| **222. Chen P-Y, Chen C-H, Chang C-K, Kao C-F, Lu M-L, Lin S-K, Huang M-C, Hwang L-L, Mondelli V. Orexin-A levels in relation to the risk of metabolic syndrome in patients with schizophrenia taking antipsychotics. Int J Neuropsychopharmacol. 2019;22(1):28-36. doi: 10.1093/ijnp/pyy075.** | **Included** |
| 223. Prihodova I, Dudova I, Mohaplova M, Hrdlicka M, Nevsimalova S. Childhood narcolepsy and autism spectrum disorders: four case reports. Sleep Med. 2018;51:167-170. doi: 10.1016/j.sleep.2018.07.017. Epub 2018 Aug 9. | Case |
| 224. Heiss JE, Yamanaka A, Kilduff TS. Parallel arousal pathways in the lateral hypothalamus. eNeuro. 2018;5(4):ENEURO.0228-18.2018. doi: 10.1523/ENEURO.0228-18.2018. | Animal |
| 225. Lu G-L, Lee MT, Chiou L-C. Orexin-mediated restoration of hippocampal synaptic potentiation in mice with established cocaine-conditioned place preference. Addict Biol. 2019;24(6):1153-1166. doi: 10.1111/adb.12672. Epub 2018 Oct 1. | Animal |
| 226. Okumura T, Nozu T, Kumei S, Ohhira M. Central oxytocin signaling mediates the central orexin-induced visceral antinociception through the opioid system in conscious rats. Physiol Behav. 2019;198:96-101. doi: 10.1016/j.physbeh.2018.10.007. Epub 2018 Oct 18. | Animal |
| 227. Zhou W, Cheung K, Kyu S, Wang L, Guan Z, Kurien PA, Bickler PE, Jan LY. Activation of orexin system facilitates anesthesia emergence and pain control. Proc Natl Acad Sci U S A. 2018;115(45):E10740-E10747. doi: 10.1073/pnas.1808622115. Epub 2018 Oct 22. | Animal |
| 228. Gerry CJ. The economic case for deinstitutionalisation in post-communist Europe. Lancet Psychiatry. 2018;5(12):950-952. doi: 10.1016/S2215-0366(18)30435-8. Epub 2018 Nov 8. | Unrelated |
| 229. Linehan V, Rowe TM, Hirasawa M. Dopamine modulates excitatory transmission to orexin neurons in a receptor subtype-specific manner. Am J Physiol Regul Integr Comp Physiol. 2019;316(1):R68-R75. doi: 10.1152/ajpregu.00150.2018. Epub 2018 Nov 21. | Animal |
| 230. Singh R, Bansal Y, Medhi B, Kuhad A. Antipsychotics-induced metabolic alterations: Recounting the mechanistic insights, therapeutic targets and pharmacological alternatives. Eur J Pharmacol. 2019;844:231-240. doi: 10.1016/j.ejphar.2018.12.003. Epub 2018 Dec 7. | Review |
| 231. McElroy SL, Guerdjikova AI, Mori N, Romo-Nava F. Progress in developing pharmacologic agents to treat bulimia nervosa. CNS Drugs. 2019;33(1):31-46. doi: 10.1007/s40263-018-0594-5. | Review |
| 232. Magdaleno-Madrigal VM, Morales-Mulia S, Nicolini H, Genis-Mendoza A, Cázares-Martínez Claudia E, Pérez-Luna José M, Morales-Mulia M. Orexin-A promotes EEG changes but fails to induce anxiety in rats. Behav Brain Res. 2019;361:26-31. doi: 10.1016/j.bbr.2018.12.037. Epub 2018 Dec 21. | Animal |
| 233. Drugs for chronic insomnia. Med Lett Drugs Ther. 2018;60(1562):201-205. | Opinion |
| 234. Kao AC, Chan KW, Anthony DC, Lennox BR, Burnet PW. Prebiotic reduction of brain histone deacetylase (HDAC) activity and olanzapine-mediated weight gain in rats, are acetate independent. Neuropharmacology. 2019;150:184-191. doi: 10.1016/j.neuropharm.2019.02.014. Epub 2019 Feb 11. | Animal |
| 235. Akinnusi M, El Solh AA. Drug treatment strategies for insomnia in patients with post-traumatic stress disorder. Expert Opin Pharmacother. 2019;20(6):691-699. doi: 10.1080/14656566.2019.1574745. Epub 2019 Feb 22. | Review |
| 236. Juvodden HT, Alnæs D, Lund MJ, Dietrichs E, Thorsby PM, Westlye LT, Knudsen S. Hypocretin-deficient narcolepsy patients have abnormal brain activation during humor processing. Sleep. 2019;42(7):zsz082. doi: 10.1093/sleep/zsz082. | No psychosis |
| 237. Naghavi FS, Namvar P, Sadeghzadeh F, Haghparast A. The involvement of intra-hippocampal dopamine receptors in the conditioned place preference induced by orexin administration into the rat ventral tegmental area. Iran J Pharm Res. 2019;18(1):328-338. | Animal |
| 238. Misiak B, Bartoli F, Stramecki F, Samochowiec J, Lis M, Kasznia J, Jarosz K, Stańczykiewicz B. Appetite regulating hormones in first-episode psychosis: A systematic review and meta-analysis. Neurosci Biobehav Rev. 2019;102:362-370. doi: 10.1016/j.neubiorev.2019.05.018. Epub 2019 May 20. | Review |
| 239. Soneson E, Russo D, Knight C, Lafortune L, Heslin M, Stochl J, Georgiadis A, Galante J, Duschinsky R, Grey N, Gonzalez-Blanco L, Couche J, Griffiths M, Murray H, Reeve N, Hodgekins J, French P, Fowler D, Byford S, Dixon-Woods M, Jones PB, Perez J. Psychological interventions for people with psychotic experiences: protocol for a systematic review and meta-analysis. Syst Rev. 2019;8(1):124. doi: 10.1186/s13643-019-1041-5. | Protocol |
| 240. Lennox B, Yeeles K, Jones PB, Zandi M, Joyce E, Yu LM, Tomei G, Pollard R, Vincent SA, Shimazaki M, Cairns I, Dowling F, Kabir T, Barnes TRE, Lingford Hughes A, Hosseini AA, Harrower T, Buckley C, Coles A. Intravenous immunoglobulin and rituximab versus placebo treatment of antibody-associated psychosis: study protocol of a randomised phase IIa double-blinded placebo- controlled trial (SINAPPS2). Trials. 2019;20(1):331. doi: 10.1186/s13063-019-3336-1. | Protocol |
| 241. Rolls ET, Cheng W, Gilson M, Gong W, Deco G, Lo CZ, Yang AC, Tsai SJ, Liu ME, Lin CP, Feng J. Beyond the disconnectivity hypothesis of schizophrenia. Cereb Cortex. 2020;30(3):1213-1233. doi: 10.1093/cercor/bhz161. | No orexin |
| 242. Ni P, Tian Y, Gu X, Yang L, Wei J, Wang Y, Zhao L, Zhang Y, Zhang C, Li L, Tang X, Ma X, Hu X, Li T. Plasma neuropeptides as circulating biomarkers of multifactorial schizophrenia. Compr Psychiatry. 2019;94:152114. doi: 10.1016/j.comppsych.2019.152114. Epub 2019 Aug 5. | Animal |
| 243. Vanda D, Zajdel P, Soural M. Imidazopyridine-based selective and multifunctional ligands of biological targets associated with psychiatric and neurodegenerative diseases. Eur J Med Chem. 2019;181:111569. doi: 10.1016/j.ejmech.2019.111569. Epub 2019 Jul 31. | Review |
| 244. Krystal AD, Prather AA, Ashbrook LH. The assessment and management of insomnia: an update. World Psychiatry. 2019;18(3):337-352. doi: 10.1002/wps.20674. | Review |
| **245. Tsuchimine S, Hattori K, Ota M, Hidese S, Teraishi T, Sasayama D, Hori H, Noda T, Yoshida S, Yoshida F, Kunugi H. Reduced plasma orexin-A levels in patients with bipolar disorder. Neuropsychiatr Dis Treat. 2019;15:2221-2230. doi: 10.2147/NDT.S209023.** | **Included** |
| 246. Bruni O, Angriman M, Melegari MG, Ferri R. Pharmacotherapeutic management of sleep disorders in children with neurodevelopmental disorders. Expert Opin Pharmacother. 2019;20(18):2257-2271. doi: 10.1080/14656566.2019.1674283. Epub 2019 Oct 22. | Review |
| 247. Stanojlovic M, Pallais JP, Lee MK, Kotz CM. Pharmacological and chemogenetic orexin/hypocretin intervention ameliorates Hipp-dependent memory impairment in the A53T mice model of Parkinson's disease. Mol Brain. 2019;12(1):87. doi: 10.1186/s13041-019-0514-8. | Animal |
| 248. Hanazawa T, Kamijo Y. Effect of suvorexant on nocturnal delirium in elderly patients with Alzheimer's disease: A case-series study. Clin Psychopharmacol Neurosci. 2019;17(4):547-550. doi: 10.9758/cpn.2019.17.4.547. | Case |

| | |
|---|---|
| 249. Chamera K, Trojan E, Szuster-Głuszczak M, Basta-Kaim A. The potential role of dysfunctions in neuron-microglia communication in the pathogenesis of brain disorders. Curr Neuropharmacol. 2020;18(5):408-430. doi: 10.2174/1570159X17666191113101629. | Review |
| 250. Sarker G, Litwan K, Kastli R, Peleg-Raibstein D. Maternal overnutrition during critical developmental periods leads to different health adversities in the offspring: relevance of obesity, addiction and schizophrenia. Sci Rep. 2019;9(1):17322. doi: 10.1038/s41598-019-53652-x. | Animal |
| 251. Chen XY, Xue Y, Chen H, Chen L. The globus pallidus as a target for neuropeptides and endocannabinoids participating in central activities. Peptides. 2020;124:170210. doi: 10.1016/j.peptides.2019.170210. Epub 2019 Nov 26. | Review |
| 252. Bada Juarez JF, Muñoz-García JC, Inácio Dos Reis R, Henry A, McMillan D, Kriek M, Wood M, Vandenplas C, Sands Z, Castro L, Taylor R, Watts A. Detergent- free extraction of a functional low-expressing GPCR from a human cell line. Biochim Biophys Acta Biomembr. 2020;1862(3):183152. doi: 10.1016/j.bbamem.2019.183152. Epub 2019 Dec 13. | *In vitro* |
| 253. Murai H, Suzuki H, Tanji H, Kimura T, Iba Y. A simple method using anesthetics to test effects of sleep-inducing substances in mice. J Pharmacol Sci. 2020;142(2):79-82. doi: 10.1016/j.jphs.2019.12.003. Epub 2019 Dec 6. | Animal |
| 254. Feketeova E, Tormasiova M, Klobučníková K, Durdik P, Jarcuskova D, Benca M, Vitkova M. Narcolepsy in Slovakia - Epidemiology, clinical and polysomnographic features, comorbid diagnoses: a case-control study. Sleep Med. 2020;67:15-22. doi: 10.1016/j.sleep.2019.10.012. Epub 2019 Nov 11. | No psychosis |
| 255. Rezaee L, Alizadeh AM, Haghparast A. Role of hippocampal dopamine receptors in the antinociceptive responses induced by chemical stimulation of the lateral hypothalamus in animal model of acute pain. Brain Res. 2020;1734:146759. doi: 10.1016/j.brainres.2020.146759. Epub 2020 Mar 2. | Animal |
| 256. Hou Y, Liu Y, Liu C, Yan Z, Ma Q, Chen J, Zhang M, Yan Q, Li X, Chen J. Xiaoyaosan regulates depression-related behaviors with physical symptoms by modulating Orexin A/OxR1 in the hypothalamus. Anat Rec (Hoboken). 2020;303(8):2144-2153. doi: 10.1002/ar.24386. Epub 2020 Mar 16. | Animal |
| 257. Su J, Li Z, Yamashita A, Kusumoto-Yoshida I, Isomichi T, Hao L, Kuwaki T. Involvement of the nucleus accumbens in chocolate-induced cataplexy. Sci Rep. 2020;10(1):4958. doi: 10.1038/s41598-020-61823-4. | Animal |
| **258. Liu Z, Zhang Y, Zhao T, Wang J, Xia L, Zhong Y, Yang Y, Ning X, Zhang Y, Ren Z, Liu H. A higher body mass index in Chinese inpatients with chronic schizophrenia is associated with elevated plasma orexin-A levels and fewer negative symptoms. Nord J Psychiatry. 2020;74(7):525-532. doi: 10.1080/08039488.2020.1755995. Epub 2020 May 4.** | **Included** |
| 259. Dujardin S, Pijpers A, Pevernagie D. Prescription drugs used in insomnia. Sleep Med Clin. 2020;15(2):133-145. doi: 10.1016/j.jsmc.2020.02.002. | Review |
| 260. BaHammam AS, Alnakshabandi K, Pandi-Perumal SR. Neuropsychiatric correlates of narcolepsy. Curr Psychiatry Rep. 2020;22(8):36. doi: 10.1007/s11920-020-01159-y. | Review |
| 261. Lis M, Stańczykiewicz B, Liśkiewicz P, Misiak B. Impaired hormonal regulation of appetite in schizophrenia: A narrative review dissecting intrinsic mechanisms and the effects of antipsychotics. Psychoneuroendocrinology. 2020;119:104744. doi: 10.1016/j.psyneuen.2020.104744. Epub 2020 Jun 5. | Review |
| 262. Matini T, Haghparast A, Rezaee L, Salehi S, Tehranchi A, Haghparast A. Role of dopaminergic receptors within the ventral tegmental area in antinociception induced by chemical stimulation of the lateral hypothalamus in an animal model of orofacial pain. J Pain Res. 2020;13:1449-1460. doi: 10.2147/JPR.S255250. | Animal |
| 263. Purple RJ, Cosgrave J, Vyazovskiy V, Foster RG, Porcheret K, Wulff K. Sleep-related memory consolidation in the psychosis spectrum phenotype. Neurobiol Learn Mem. 2020;174:107273. doi: 10.1016/j.nlm.2020.107273. Epub 2020 Jul 10. | No orexin |
| 264. Chan SY, Probert F, Radford-Smith DE, Hebert JC, Claridge TDW, Anthony DC, Burnet PWJ. Post-inflammatory behavioural despair in male mice is associated with reduced cortical glutamate-glutamine ratios, and circulating lipid and energy metabolites. Sci Rep. 2020;10(1):16857. doi: 10.1038/s41598-020-74008-w. | Animal |
| 265. Al-Kuraishy HM, Abdulhadi MH, Hussien NR, Al-Niemi MS, Rasheed HA, Al- Gareeb AI. Involvement of orexinergic system in psychiatric and neurodegenerative disorders: A scoping review. Brain Circ. 2020;6(2):70-80. doi: 10.4103/bc.bc_42_19. | Review |
| 266. Molnár Z, Luhmann HJ, Kanold PO. Transient cortical circuits match spontaneous and sensory-driven activity during development. Science. 2020;370(6514):eabb2153. doi: 10.1126/science.abb2153. | Review |
| 267. Durairaja A, Fendt M. Orexin deficiency modulates cognitive flexibility in a sex-dependent manner. Genes Brain Behav. 2021;20(3):e12707. doi: 10.1111/gbb.12707. Epub 2020 Nov 3. | Animal |
| 268. Khaleghzadeh-Ahangar H, Rashvand M, Haghparast A. Role of D1- and D2-like dopamine receptors within the dentate gyrus in antinociception induced by chemical stimulation of the lateral hypothalamus in an animal model of acute pain. Physiol Behav. 2021;229:113214. doi: 10.1016/j.physbeh.2020.113214. Epub 2020 Oct 20. | Animal |
| 269. Černis E, Evans R, Ehlers A, Freeman D. Dissociation in relation to other mental health conditions: An exploration using network analysis. J Psychiatr Res. 2021;136:460-467. doi: 10.1016/j.jpsychires.2020.08.023. Epub 2020 Aug 20. | No orexin |
| 270. Kenaan K, Zafar M, Bond R, Gracious BL. Perampanel-induced cataplexy in a young male with generalized epilepsy. HCA Healthc J Med. 2020;1(5):283-288. doi: 10.36518/2689-0216.1040. | Case |
| 271. Chén OY, Cao H, Phan H, Nagels G, Reinen JM, Gou J, Qian T, Di J, Prince J, Cannon TD, de Vos M. Identifying neural signatures mediating behavioral symptoms and psychosis onset: High-dimensional whole brain functional mediation analysis. Neuroimage. 2021;226:117508. doi: 10.1016/j.neuroimage.2020.117508. Epub 2020 Nov 4. | No orexin |
| 272. Ashraf GM, Alghamdi BS, Alshehri FS, Alam MZ, Tayeb HO, Tarazi FI. Standardizing the effective correlated dosage of olanzapine and empagliflozin in female Wistar rats. Curr Gene Ther. 2021;21(1):53-59. doi: 10.2174/1566523220999201111195047. | Animal |
| 273. Nigam M, Leu-Semenescu S, Arnulf I. Successful treatment of drug-resistant cataplexy with the anticholinergic drug tropatepine. J Clin Sleep Med. 2021;17(4):849-851. doi: 10.5664/jcsm.9030. | Case |
| 274. Geraghty Z, Barnard C, Uluocak P, Gruneberg U. The association of Plk1 with the astrin-kinastrin complex promotes formation and maintenance of a metaphase plate. J Cell Sci. 2021;134(1):jcs251025. doi: 10.1242/jcs.251025. | Unrelated |
| 275. Martins D, Rademacher L, Gabay AS, Taylor R, Richey JA, Smith DV, Goerlich KS, Nawijn L, Cremers HR, Wilson R, Bhattacharyya S, Paloyelis Y. Mapping social reward and punishment processing in the human brain: A voxel-based meta-analysis of neuroimaging findings using the social incentive delay task. Neurosci Biobehav Rev. 2021;122:1-17. doi: 10.1016/j.neubiorev.2020.12.034. Epub 2021 Jan 6. | Review |
| 276. Elam HB, Perez SM, Donegan JJ, Lodge DJ. Orexin receptor antagonists reverse aberrant dopamine neuron activity and related behaviors in a rodent model of stress-induced psychosis. Transl Psychiatry. 2021;11(1):114. doi: 10.1038/s41398-021-01235-8. | Animal |
| 277. Crawford K, Oliver PL, Agnew T, Hunn BHM, Ahel I. Behavioural characterisation of *Macrod1* and *Macrod2* knockout mice. Cells. 2021;10(2):368. doi: 10.3390/cells10020368. | Animal |
| 278. Perez SM, Lodge DJ. Orexin modulation of VTA dopamine neuron activity: Relevance to schizophrenia. Int J Neuropsychopharmacol. 2021;24(4):344-353. doi: 10.1093/ijnp/pyaa080. | Animal |
| 279. Veeraraghavan V. Obesogenic behavior and binge eating disorder in an elderly female with schizophrenia. J Obes Metab Syndr. 2021;30(2):184-187. doi: 10.7570/jomes20096. | Case |
| 280. Ashraf GM, Alghamdi BS, Alshehri FS, Alam MZ, Tayeb HO, Tarazi FI. Empagliflozin effectively attenuates olanzapine-induced body weight gain in female Wistar rats. Front Pharmacol. 2021;12:578716. doi: 10.3389/fphar.2021.578716. | Animal |

| | |
|---|---|
| **281. Lu J, Huang ML, Li JH, Jin KY, Li HM, Mou TT, Fronczek R, Duan JF, Xu WJ, Swaab D, Bao AM. Changes of hypocretin (Orexin) system in schizophrenia: From plasma to brain. Schizophr Bull. 2021;47(5):1310-1319. doi: 10.1093/schbul/sbab042.** | **Included** |
| 282. Saatci D, van Nieuwenhuizen A, Handunnetthi L. Maternal infection in gestation increases the risk of non-affective psychosis in offspring: a meta-analysis. J Psychiatr Res. 2021;139:125-131. doi: 10.1016/j.jpsychires.2021.05.039. Epub 2021 May 23. | No orexin |
| 283. Perry BAL, Lomi E, Mitchell AS. Thalamocortical interactions in cognition and disease: The mediodorsal and anterior thalamic nuclei. Neurosci Biobehav Rev. 2021;130:162-177. doi: 10.1016/j.neubiorev.2021.05.032. Epub 2021 Jun 30. | Review |
| 284. Nasrollahi S, Karimi S, Hamidi G, Naderitehrani M, Abed A. Blockade of the orexin 1 receptors in the nucleus accumbens' shell reversed the reduction effect of olanzapine on motivation for positive reinforcers. Neurosci Lett. 2021;762:136137. doi: 10.1016/j.neulet.2021.136137. Epub 2021 Jul 24. | Animal |
| 285. Molina JD, Avila S, Rubio G, López-Muñoz F. Metabolomic connections between schizophrenia, antipsychotic drugs and metabolic syndrome: A variety of players. Curr Pharm Des. 2021;27(39):4049-4061. doi: 10.2174/1381612827666210804110139. | Review |
| 286. Grünwald LM, Duddy C, Byng R, Crellin N, Moncrieff J. The role of trust and hope in antipsychotic medication reviews between GPs and service users a realist review. BMC Psychiatry. 2021;21(1):390. doi: 10.1186/s12888-021-03355-3. | No orexin |
| 287. Pisani S, Murphy J, Conway J, Millgate E, Catmur C, Bird G. The relationship between alexithymia and theory of mind: A systematic review. Neurosci Biobehav Rev. 2021;131:497-524. doi: 10.1016/j.neubiorev.2021.09.036. Epub 2021 Sep 29. | Review |
| 288. Porwal A, Yadav YC, Pathak K, Yadav R. An update on assessment, therapeutic management, and patents on insomnia. Biomed Res Int. 2021;2021:6068952. doi: 10.1155/2021/6068952. | Review |
| 289. Černis E, Molodynski A, Ehlers A, Freeman D. Dissociation in patients with non-affective psychosis: Prevalence, symptom associations, and maintenance factors. Schizophr Res. 2022;239:11-18. doi: 10.1016/j.schres.2021.11.008. Epub 2021 Nov 17. | No orexin |
| 290. Handunnetthi L, Saatci D, Hamley JC, Knight JC. Maternal immune activation downregulates schizophrenia genes in the foetal mouse brain. Brain Commun. 2021;3(4):fcab275. doi: 10.1093/braincomms/fcab275. | Animal |
| 291. Iakovleva OV, Levin OS (Яковлева О.В., Левин О.С.). Речевые и поведенческие контаминации как неэпилептические автоматизмы при болезни Паркинсона [Speech and behavioral contaminations as non-epileptic automatic behavior in Parkinson's disease]. Zh Nevrol Psikhiatr Im S S Korsakova. 2021;121(10. Vyp. 2):58-63 (Журнал неврологии и психиатрии им. С.С. Корсакова 2021;т. 121(№10, вып. 2)с. 58-63). Russian. doi: 10.17116/jnevro202112110258. | Review |
| 292. Terada T, Hirayama T, Sadahiro R, Wada S, Nakahara R, Matsuoka H. Pilot study of lemborexant for insomnia in cancer patients with delirium. J Palliat Med. 2022;25(5):797-801. doi: 10.1089/jpm.2021.0509. Epub 2022 Jan 28. | Unfocused |
| 293. Demidova A, Kahl E, Fendt M. Orexin deficiency affects sensorimotor gating and its amphetamine-induced impairment. Prog Neuropsychopharmacol Biol Psychiatry. 2022;116:110517. doi: 10.1016/j.pnpbp.2022.110517. Epub 2022 Jan 29. | Animal |
| 294. Chyr J, Gong H, Zhou X. DOTA: Deep Learning Optimal Transport Approach to advance drug repositioning for Alzheimer's disease. Biomolecules. 2022;12(2):196. doi: 10.3390/biom12020196. | Unfocused |
| 295. Cope TE, Hughes LE, Phillips HN, Adams NE, Jafarian A, Nesbitt D, Assem M, Woolgar A, Duncan J, Rowe JB. Causal evidence for the multiple demand network in change detection: Auditory mismatch magnetoencephalography across focal neurodegenerative diseases. J Neurosci. 2022;42(15):3197-3215. doi: 10.1523/JNEUROSCI.1622-21.2022. Epub 2022 Mar 8. | No orexin |
| 296. Maidment ID, Wong G, Duddy C, Upthegrove R, Oduola S, Robotham D, Higgs S, Ahern A, Birdi G. REalist Synthesis Of non-pharmacologicaL interVEntions for antipsychotic-induced weight gain (RESOLVE) in people living with severe mental illness (SMI). Syst Rev. 2022;11(1):42. doi: 10.1186/s13643-022-01912-9. | Protocol |
| 297. Guma E, Bordeleau M, González Ibáñez F, Picard K, Snook E, Desrosiers-Grégoire G, Spring S, Lerch JP, Nieman BJ, Devenyi GA, Tremblay ME, Chakravarty MM. Differential effects of early or late exposure to prenatal maternal immune activation on mouse embryonic neurodevelopment. Proc Natl Acad Sci U S A. 2022;119(12):e2114545119. doi: 10.1073/pnas.2114545119. Epub 2022 Mar 14. | Animal |
| 298. Phiri P, Engelthaler T, Carr H, Delanerolle G, Holmes C, Rathod S. Associated mortality risk of atypical antipsychotic medication in individuals with dementia. World J Psychiatry. 2022;12(2):298-307. doi: 10.5498/wjp.v12.i2.298. | No orexin |
| **299. Chen PY, Chang CK, Chen CH, Fang SC, Mondelli V, Chiu CC, Lu ML, Hwang LL, Huang MC. Orexin-a elevation in antipsychotic-treated compared to drug-free patients with schizophrenia: A medication effect independent of metabolic syndrome. J Formos Med Assoc. 2022;121(11):2172-2181. doi: 10.1016/j.jfma.2022.03.008. Epub 2022 Apr 6.** | **Included** |
| 300. Lammas F, Phillips A, Dopson S, Joyce E, Csipke E, Wykes T. The organisational climate of NHS Early Intervention Services (EIS) for psychosis: a qualitative analysis. BMC Health Serv Res. 2022;22(1):509. doi: 10.1186/s12913-022-07790-0. | No orexin |
| 301. Radley J, Barlow J, Johns LC. Sociodemographic characteristics associated with parenthood amongst patients with a psychotic diagnosis: a cross-sectional study using patient clinical records. Soc Psychiatry Psychiatr Epidemiol. 2022;57(9):1897-1906. doi: 10.1007/s00127-022-02279-x. Epub 2022 Apr 21. | No orexin |
| 302. Palagini L, Hertenstein E, Riemann D, Nissen C. Sleep, insomnia and mental health. J Sleep Res. 2022;31(4):e13628. doi: 10.1111/jsr.13628. Epub 2022 May 4. | Review |
| 303. Brown RE, Spratt TJ, Kaplan GB. Translational approaches to influence sleep and arousal. Brain Res Bull. 2022;185:140-161. doi: 10.1016/j.brainresbull.2022.05.002. Epub 2022 May 10. | Review |
| **304. Ren J, Chen Y, Fang X, Wang D, Wang Y, Yu L, Wu Z, Liu R, Zhang C. Correlation of Orexin-A and brain-derived neurotrophic factor levels in metabolic syndrome and cognitive impairment in schizophrenia treated with clozapine. Neurosci Lett. 2022;782:136695. doi: 10.1016/j.neulet.2022.136695. Epub 2022 May 24.** | **Included** |
| 305. Delanerolle G, Zeng Y, Shi JQ, Yeng X, Goodison W, Shetty A, Shetty S, Haque N, Elliot K, Ranaweera S, Ramakrishnan R, Raymont V, Rathod S, Phiri P. Mental health impact of the Middle East respiratory syndrome, SARS, and COVID-19: A comparative systematic review and meta-analysis. World J Psychiatry. 2022;12(5):739-765. doi: 10.5498/wjp.v12.i5.739. | Review |
| 306. Li S, Zhang R, Hu S, Lai J. Plasma orexin-A levels in patients with schizophrenia: A systematic review and meta-analysis. Front Psychiatry. 2022;13:879414. doi: 10.3389/fpsyt.2022.879414. | Review |
| 307. Vasiliu O. Investigational drugs for the treatment of depression (Part 1): Monoaminergic, orexinergic, GABA-ergic, and anti-inflammatory agents. Front Pharmacol. 2022;13:884143. doi: 10.3389/fphar.2022.884143. | Review |
| 308. Daridorexant (Quviviq) for insomnia. Med Lett Drugs Ther. 2022;64(1654):107-110. | Opinion |
| 309. Gool JK, Fronczek R, Bosma P, van der Meer JN, van der Werf YD, Lammers GJ. Enhanced visual cortex activation in people with Narcolepsy Type 1 during active sleep resistance: An fMRI-EEG study. Front Neurosci. 2022;16:904820. doi: 10.3389/fnins.2022.904820. | No psychosis |
| 310. Musa A, Khan S, Mujahid M, El-Gaby M. The shallow cognitive map hypothesis: A hippocampal framework for thought disorder in schizophrenia. Schizophrenia (Heidelb). 2022;8(1):34. doi: 10.1038/s41537-022-00247-7. | Review |
| 311. Saatci D, Johnson T, Smee M, van Nieuwenhuizen A, Handunnetthi L. The role of latitude and infections in the month-of-birth effect linked to schizophrenia. Brain Behav Immun Health. 2022;24:100486. doi: 10.1016/j.bbih.2022.100486. | Review |
| 312. Lin CC, Huang TL. Orexin/hypocretin and major psychiatric disorders. Adv Clin Chem. 2022;109:185-212. doi: 10.1016/bs.acc.2022.03.006. Epub 2022 Apr 18. | Review |
| 313. Osipov EM, Munawar AH, Beelen S, Fearon D, Douangamath A, Wild C, Weeks SD, Van Aerschot A, von Delft F, Strelkov SV. Discovery of novel druggable pockets on polyomavirus VP1 through crystallographic fragment-based screening to develop capsid assembly inhibitors. RSC Chem Biol. 2022;3(8):1013-1027. doi: 10.1039/d2cb00052k. | *In vitro* |

| | |
|---|---|
| 314. Zhu Z, Gu Y, Zeng C, Yang M, Yu H, Chen H, Zhang B, Cai H. Olanzapine-induced lipid disturbances: A potential mechanism through the gut microbiota-brain axis. Front Pharmacol. 2022;13:897926. doi: 10.3389/fphar.2022.897926. | Animal |
| 315. Hong J, Vernon D, Kunovac J, Stahl S. Emerging drugs for the treatment of major depressive disorder. Expert Opin Emerg Drugs. 2022;27(3):263-275. doi: 10.1080/14728214.2022.2117297. Epub 2022 Sep 20. | Review |
| 316. Mikutta CA, Pervilhac C, Znoj H, Federspiel A, Müller TJ. The impact of foehn wind on mental distress among patients in a Swiss psychiatric hospital. Int J Environ Res Public Health. 2022;19(17):10831. doi: 10.3390/ijerph191710831. | No orexin |
| 317. Hintze JP, Edinger JD. Hypnotic discontinuation in chronic insomnia. Sleep Med Clin. 2022;17(3):523-530. doi: 10.1016/j.jsmc.2022.06.014. | Review |
| 318. Kalra S, Bathla M, Verma S. Lemborexant: An adjuvant in difficult-to-control diabetes? J Pak Med Assoc. 2022;72(9):1874-1875. doi: 10.47391/JPMA.22-93. | Opinion |
| 319. Karimi S, Zibaii MI, Hamidi GA, Haghparast A. Differential effects of the lateral hypothalamus lesion as an origin of orexin and blockade of orexin-1 receptor in the orbitofrontal cortex and anterior cingulate cortex on their neuronal activity. Basic Clin Neurosci. 2022;13(3):407-420. doi: 10.32598/bcn.2022.2029.1. Epub 2022 May 1. | Animal |
| 320. Panayi MC, Boerner T, Jahans-Price T, Huber A, Sprengel R, Gilmour G, Sanderson DJ, Harrison PJ, Walton ME, Bannerman DM. Glutamatergic dysfunction leads to a hyper-dopaminergic phenotype through deficits in short-term habituation: a mechanism for aberrant salience. Mol Psychiatry. 2023;28(2):579-587. doi: 10.1038/s41380-022-01861-8. Epub 2022 Dec 2. | Animal |
| 321. Tang PY, Tee SF, Su KP. Editorial: The link between nutrition and schizophrenia. Front Psychiatry. 2022;13:1074120. doi: 10.3389/fpsyt.2022.1074120. | Opinion |
| 322. Bergamini G, Coloma P, Massinet H, Steiner MA. What evidence is there for implicating the brain orexin system in neuropsychiatric symptoms in dementia? Front Psychiatry. 2022;13:1052233. doi: 10.3389/fpsyt.2022.1052233. | Review |
| 323. Drugs for chronic insomnia. Med Lett Drugs Ther. 2023;65(1667):1-6. doi: 10.58347/tml.2023.1667a. | Opinion |
| 324. Feketeová E, Dragašek J, Klobučníková K, Ďurdík P, Čarnakovič S, Slavkovská M, Chylová M. Psychotic episode and schizophrenia in Slovakian Narcolepsy Database. Brain Sci. 2022;13(1):43. doi: 10.3390/brainsci13010043. | No psychosis |
| 325. Malik JA, Yaseen Z, Thotapalli L, Ahmed S, Shaikh MF, Anwar S. Understanding translational research in schizophrenia: A novel insight into animal models. Mol Biol Rep. 2023;50(4):3767-3785. doi: 10.1007/s11033-023-08241-7. Epub 2023 Jan 24. Erratum in: Mol Biol Rep. 2023;50(5):4755. doi: 10.1007/s11033-023-08352-1. | Review |
| 326. Han AH, Burroughs CR, Falgoust EP, Hasoon J, Hunt G, Kakazu J, Lee T, Kaye AM, Kaye AD, Ganti L. Suvorexant, a novel dual orexin receptor antagonist, for the management of insomnia. Health Psychol Res. 2023;10(5):67898. doi: 10.52965/001c.67898. | Review |
| 327. Maness EB, Blumenthal SA, Burk JA. Dual orexin/hypocretin receptor antagonism attenuates attentional impairments in an NMDA receptor hypofunction model of schizophrenia. bioRxiv [Preprint]. 2023 Feb 5:2023.02.05.527043. doi: 10.1101/2023.02.05.527043. Update in: Behav Brain Res. 2023;450:114497. doi: 10.1016/j.bbr.2023.114497. | Dupl |
| 328. Lee C, Waite F, Piernas C, Aveyard P. Development and initial evaluation of a behavioural intervention to support weight management for people with serious mental illness: an uncontrolled feasibility and acceptability study. BMC Psychiatry. 2023;23(1):130. doi: 10.1186/s12888-023-04517-1. | No orexin |
| 329. Okuda S, Qureshi ZP, Yanagida Y, Ito C, Homma Y, Tokita S. Factors associated with prescriptions for an orexin receptor antagonist among Japanese patients with insomnia: Analysis of a nationwide Japanese claims database. Drugs Real World Outcomes. 2023;10(2):271-281. doi: 10.1007/s40801-023-00356-4. Epub 2023 Mar 3. | No psychosis |
| 330. Mana L, Vila-Vidal M, Köckeritz C, Aquino K, Fornito A, Kringelbach ML, Deco G. Using in silico perturbational approach to identify critical areas in schizophrenia. Cereb Cortex. 2023;33(12):7642-7658. doi: 10.1093/cercor/bhad067. | No orexin |
| 331. Wang MO, Ma J, Li SX, Zhang L. 王美鸥，马　菁，李思迅，张　岚. 1型发作性睡病共病精神分裂症1例报告 [Narcolepsy Type 1 With Comorbid Schizophrenia: A Case Report]. Sichuan Da Xue Xue Bao Yi Xue Ban 四川大学学报（医学版）. 2023;54(2):444-446. Chinese. doi: 10.12182/20230360104. | Case |
| 332. Sariaslan A, Fanshawe T, Pitkänen J, Cipriani A, Martikainen P, Fazel S. Predicting suicide risk in 137,112 people with severe mental illness in Finland: external validation of the Oxford Mental Illness and Suicide tool (OxMIS). Transl Psychiatry. 2023;13(1):126. doi: 10.1038/s41398-023-02422-5. | No orexin |
| 333. Lin Y, Roy K, Ioka S, Otani R, Amezawa M, Ishikawa Y, Cherasse Y, Kaushik MK, Klewe-Nebenius D, Zhou L, Yanagisawa M, Oishi Y, Saitoh T, Lazarus M. Positive allosteric adenosine $A_{2A}$ receptor modulation suppresses insomnia associated with mania- and schizophrenia-like behaviors in mice. Front Pharmacol. 2023;14:1138666. doi: 10.3389/fphar.2023.1138666. | Animal |
| 334. Leonardsen EH, Vidal-Piñeiro D, Roe JM, Frei O, Shadrin AA, Iakunchykova O, de Lange AG, Kaufmann T, Taschler B, Smith SM, Andreassen OA, Wolfers T, Westlye LT, Wang Y. Genetic architecture of brain age and its causal relations with brain and mental disorders. Mol Psychiatry. 2023;28(7):3111-3120. doi: 10.1038/s41380-023-02087-y. Epub 2023 May 10. | No orexin |
| 335. Williams SG, Rodriguez-Cué D. Use of daridorexant among patients with chronic insomnia: A retrospective observational analysis. J Clin Med. 2023;12(9):3240. doi: 10.3390/jcm12093240. | No psychosis |
| 336. Fagan HA, Baldwin DS. Pharmacological treatment of generalised anxiety disorder: Current practice and future directions. Expert Rev Neurother. 2023;23(6):535-548. doi: 10.1080/14737175.2023.2211767. Epub 2023 May 15. | Review |
| 337. Maness EB, Blumenthal SA, Burk JA. Dual orexin/hypocretin receptor antagonism attenuates NMDA receptor hypofunction-induced attentional impairments in a rat model of schizophrenia. Behav Brain Res. 2023;450:114497. doi: 10.1016/j.bbr.2023.114497. Epub 2023 May 16. | Animal |
| 338. Horikoshi S, Miura I, Suzuki Y, Kobayashi Y, Hirata Y, Goto M, Ichinose M, Yamamoto S, Kanno-Nozaki K, Watanabe K, Yabe H. Switching to lemborexant for the management of insomnia in mental disorders: the SLIM study. J Clin Sleep Med. 2023;19(10):1753-1758. doi: 10.5664/jcsm.10668. | Unfocused |
| 339. Abdelzaher WY, De Waard M, Abdelmonaem AA, Ali DM, El-Tahawy NFG, Rifaai RA, Mohamed HA, Shaheen K, Zeen El-Din MA, Welson NN, Tawfeek SE, Batiha GE, Abdel-Aziz AM. Empagliflozin protects against haloperidol experimentally-induced ovarian toxicity. Pharmaceuticals (Basel). 2023;16(2):168. doi: 10.3390/ph16020168. | Animal |
| **340. Yu H, Ni P, Zhao L, Tian Y, Li M, Li X, Wei W, Wei J, Deng W, Du X, Wang Q, Guo W, Ma X, Coid J, Li T. Decreased plasma neuropeptides in first-episode schizophrenia, bipolar disorder, major depressive disorder: associations with clinical symptoms and cognitive function. Front Psychiatry. 2023;14:1180720. doi: 10.3389/fpsyt.2023.1180720.** | **Included** |
| 341. Nakamura T, Yoshizawa T, Toya R, Terasawa M, Takahashi K, Kitazawa K, Suzuki K, Sasayama D, Washizuka S. Orexin receptor antagonists versus antipsychotics for the management of delirium in intensive care unit patients with cardiovascular disease: A retrospective observational study. Gen Hosp Psychiatry. 2023;84:96-101. doi: 10.1016/j.genhosppsych.2023.06.019. Epub 2023 Jul 1. | Unfocused |
| 342. Hansen BH, Andresen HN, Gjesvik J, Thorsby PM, Naerland T, Knudsen-Heier S. Associations between psychiatric comorbid disorders and executive dysfunctions in hypocretin-1 deficient pediatric narcolepsy type1. Sleep Med. 2023;109:149-157. doi: 10.1016/j.sleep.2023.06.021. Epub 2023 Jul 1. | No psychosis |
| 343. Yao Y, Baronio D, Chen YC, Jin C, Panula P. The roles of histamine receptor 1 (hrh1) in neurotransmitter system regulation, behavior, and neurogenesis in zebrafish. Mol Neurobiol. 2023;60(11):6660-6675. doi: 10.1007/s12035-023-03447-z. Epub 2023 Jul 20. | Animal |

| | |
|---|---|
| 344. Watanabe K, Misaka S, Kanno-Nozaki K, Chiyoda T, Suzuki Y, Sato A, Suto T, Kuroda J, Shimomura K, Miura I, Yabe H. Effect of lemborexant on pharmacokinetics of clozapine: A potential drug-drug interaction mediated by time-dependent inhibition of CYP3A4. Br J Clin Pharmacol. 2024;90(1):354-359. doi: 10.1111/bcp.15889. Epub 2023 Sep 4. | Case |
| 345. Harrison PJ, Bannerman DM. GRIN2A (NR2A): a gene contributing to glutamatergic involvement in schizophrenia. Mol Psychiatry. 2023;28(9):3568-3572. doi: 10.1038/s41380-023-02265-y. Epub 2023 Sep 22. | Opinion |
| 346. Tong Z, Smith PJ, Pickford HD, Christensen KE, Anderson EA. Gold-catalyzed cyclization of yndiamides with isoxazoles via α-imino gold Fischer carbenes. Chemistry. 2023;29(70):e202302821. doi: 10.1002/chem.202302821. Epub 2023 Oct 25. | *In vitro* |
| 347. Diepenbroek C, Rijnsburger M, van Irsen AAS, Eggels L, Kisner A, Foppen E, Unmehopa UA, Berland C, Dólleman S, Hardonk M, Cruciani-Guglielmacci C, Faust RP, Wenning R, Maya-Monteiro CM, Kalsbeek A, Aponte Y, Luquet S, Serlie MJM, la Fleur SE. Dopamine in the nucleus accumbens shell controls systemic glucose metabolism via the lateral hypothalamus and hepatic vagal innervation in rodents. Metabolism. 2024;150:155696. doi: 10.1016/j.metabol.2023.155696. Epub 2023 Oct 5. | Animal |
| 348. Uematsu T, Tomita T, Obara R, Gonai T, Hattori K, Aonuma T, Usui K, Tanifuji H, Ishizawa F, Ishii H, Suzuki E. Reducing the use of psychotropics in a convalescent rehabilitation ward. Neuropsychopharmacol Rep. 2024;44(1):227-233. doi: 10.1002/npr2.12388. Epub 2023 Oct 26. | Unfocused |
| 349. Montastruc F, Taillefer de Laportaliere T. Drug-induced psychiatric disorders: A pharmacovigilance update. Thérapie. 2024;79(2):173-179. doi: 10.1016/j.therap.2023.09.007. Epub 2023 Oct 31. | Review |
| 350. Guaiana G, Meader N, Barbui C, Davies SJ, Furukawa TA, Imai H, Dias S, Caldwell DM, Koesters M, Tajika A, Bighelli I, Pompoli A, Cipriani A, Dawson S, Robertson L. Pharmacological treatments in panic disorder in adults: a network meta-analysis. Cochrane Database Syst Rev. 2023;11(11):CD012729. doi: 10.1002/14651858.CD012729.pub3. | Review |
| 351. Riemann D, Espie CA, Altena E, Arnardottir ES, Baglioni C, Bassetti CLA, Bastien C, Berzina N, Bjorvatn B, Dikeos D, Dolenc Groselj L, Ellis JG, Garcia-Borreguero D, Geoffroy PA, Gjerstad M, Gonçalves M, Hertenstein E, Hoedlmoser K, Hion T, Holzinger B, Janku K, Jansson-Fröjmark M, Järnefelt H, Jernelöv S, Jennum PJ, Khachatryan S, Krone L, Kyle SD, Lancee J, Leger D, Lupusor A, Marques DR, Nissen C, Palagini L, Paunio T, Perogamvros L, Pevernagie D, Schabus M, Shochat T, Szentkiralyi A, Van Someren E, van Straten A, Wichniak A, Verbraecken J, Spiegelhalder K. The European Insomnia Guideline: An update on the diagnosis and treatment of insomnia 2023. J Sleep Res. 2023;32(6):e14035. doi: 10.1111/jsr.14035. | Review |
| 352. Varadharajan A, Davis AD, Ghosh A, Jagtap T, Xavier A, Menon AJ, Roy D, Gandhi S, Gregor T. Guidelines for pharmacotherapy in Alzheimer's disease - A primer on FDA-approved drugs. J Neurosci Rural Pract. 2023;14(4):566-573. doi: 10.25259/JNRP_356_2023. Epub 2023 Oct 7. | Review |
| 353. Cipriani A, Agunbiade A, Salanti G. Muscarinic drug shows efficacy in schizophrenia but much is left to be discovered. Lancet. 2024;403(10422):120-122. doi: 10.1016/S0140-6736(23)02415-7. Epub 2023 Dec 14. | Opinion |
| 354. Hu J, Wei SS, Jiang HZ, Luo JY, Yang W, Zhang YM, Wang XB, Wen CN (胡金;韦姗姗;姜海洲;罗静怡;杨薇;张云敏;王欣波;文朝楠;来源). 失眠的药物治疗研究进展 [Research progress in pharmacotherapy of insomnia]. Zhongguo Zhong Yao Za Zhi (中国中药杂志). 2023;48(19):5122-5130. Chinese. doi: 10.19540/j.cnki.cjcmm.20230721.601. | Review |
| 355. Efthimiou O, Taipale H, Radua J, Schneider-Thoma J, Pinzón-Espinosa J, Ortuño M, Vinkers CH, Mittendorfer-Rutz E, Cardoner N, Tanskanen A, Fusar-Poli P, Cipriani A, Vieta E, Leucht S, Tiihonen J, Luykx JJ. Efficacy and effectiveness of antipsychotics in schizophrenia: network meta-analyses combining evidence from randomised controlled trials and real-world data. Lancet Psychiatry. 2024;11(2):102-111. doi: 10.1016/S2215-0366(23)00366-8. Epub 2024 Jan 9. | Review |
| 356. Piri F, Salmani ME, Sepehri H. Improvement of autistic-like behaviors in adult rats prenatally exposed to valproic acid through early suppression of orexin receptor. Ann Med Surg (Lond). 2023;86(1):166-171. doi: 10.1097/MS9.0000000000000788. | Animal |
| **357. Chen PY, Chiu CC, Chang CK, Lu ML, Huang CY, Chen CH, Huang MC. Higher orexin-A levels are associated with treatment response to clozapine in patients with schizophrenia: A cross-sectional study. J Psychopharmacol. 2024;38(3):258-267. doi: 10.1177/02698811231225610. Epub 2024 Jan 27.** | **Included** |
| 358. Glen A, Bürli RW, Livermore D, Buffham W, Merison S, Rowland AE, Newman R, Fieldhouse C, Miller DJ, Dawson LA, Matthews K, Carlton MB, Brice NL. Discovery and first-time disclosure of CVN766, an exquisitely selective orexin 1 receptor antagonist. Bioorg Med Chem Lett. 2024;100:129629. doi: 10.1016/j.bmcl.2024.129629. Epub 2024 Jan 30. | *In vitro* |
| 359. Mori Y, Watanabe K, Suzuki Y, Ono H, Tojo M, Kawasaki Y, Kanno-Nozaki K, Nozaki M, Miura I. Clozapine-associated myocarditis in a patient with schizophrenia taking lemborexant: A case report. J Clin Psychopharmacol. 2024;44(2):193-195. doi: 10.1097/JCP.0000000000001817. Epub 2024 Feb 7. | Case |
| 360. Meyer N, Lok R, Schmidt C, Kyle SD, McClung CA, Cajochen C, Scheer FAJL, Jones MW, Chellappa SL. The sleep-circadian interface: A window into mental disorders. Proc Natl Acad Sci U S A. 2024;121(9):e2214756121. doi: 10.1073/pnas.2214756121. Epub 2024 Feb 23. | Review |
| 361. Fornaro M, Caiazza C, Rossano F, Cilmi F, De Prisco M, Vieta E, Thompson T, Solmi M, Carvalho AF, Iasevoli F, de Bartolomeis A. Residual effects of medications for sleep disorders on driving performance: A systematic review and network meta-analysis of randomized controlled trials: NMA driving and hypnotics. Eur Neuropsychopharmacol. 2024;81:53-63. doi: 10.1016/j.euroneuro.2024.01.011. Epub 2024 Feb 23. | Review |
| 362. Öz P, Kamalı O, Saka HB, Gör C, Uzbay İT. Baseline prepulse inhibition dependency of orexin A and REM sleep deprivation. Psychopharmacology (Berl). 2024;241(6):1213-1225. doi: 10.1007/s00213-024-06555-3. Epub 2024 Mar 1. | Animal |
| 363. Mutz J, Wong WLE, Powell TR, Young AH, Dawe GS, Lewis CM. The duration of lithium use and biological ageing: telomere length, frailty, metabolomic age and all-cause mortality. Geroscience. 2024;46(6):5981-5994. doi: 10.1007/s11357-024-01142-y. Epub 2024 Mar 28. | No orexin |
| 364. Aubin H-J. Repurposing drugs for treatment of alcohol use disorder. Int Rev Neurobiol. 2024;175:153-185. doi: 10.1016/bs.irn.2024.02.002. Epub 2024 Mar 12. | Review |
| 365. Haniff ZR, Bocharova M, Mantingh T, Rucker JJ, Velayudhan L, Taylor DM, Young AH, Aarsland D, Vernon AC, Thuret S. Psilocybin for dementia prevention? The potential role of psilocybin to alter mechanisms associated with major depression and neurodegenerative diseases. Pharmacol Ther. 2024;258:108641. doi: 10.1016/j.pharmthera.2024.108641. Epub 2024 Apr 6. | Review |
| 366. Álamo C, Sáiz Ruiz J, Zaragozá Arnáez C. Orexinergic receptor antagonists as a new therapeutic target to overcome limitations of current pharmacological treatment of insomnia disorder. Actas Esp Psiquiatr. 2024;52(2):172-182. doi: 10.62641/aep.v52i2.1659. | Review |
| 367. Dolz M, Tor J, Puig O, de la Serna E, Muñoz-Samons D, Pardo M, Alvarez-Subiela X, Rodriguez-Pascual M, Sugranyes G, Ilzarbe D, Baeza I. Clinical and neurodevelopmental predictors of psychotic disorders in children and adolescents at clinical high risk for psychosis: the CAPRIS study. Eur Child Adolesc Psychiatry. 2024;33(11):3925-3935. doi: 10.1007/s00787-024-02436-4. Epub 2024 Apr 20. | No orexin |
| 368. Jelen LA, McShane R, Young AH. Guidelines for ketamine use in clinical psychiatry practice. BJPsych Open. 2024;10(3):e107. doi: 10.1192/bjo.2024.62. | Opinion |
| 369. Thangwaritorn S, Lee C, Metchikoff E, Razdan V, Ghafary S, Rivera D, Pinto A, Pemminati S. A review of recent advances in the management of Alzheimer's disease. Cureus. 2024;16(4):e58416. doi: 10.7759/cureus.58416. | Review |
| 370. Lodge P. "Pyrrhonism" as a therapeutic response to the allure of mania. Schizophr Bull. 2024:sbae068. doi: 10.1093/schbul/sbae068. Epub ahead of Print 2024 May 24. | Opinion |
| 371. Nakamura T, Furihata R, Hasegawa N, Kodaka F, Muraoka H, Ichihashi K, Ochi S, Numata S, Tsuboi T, Makinodan M, Iida H, Onitsuka T, Kashiwagi H, Takeshima M, Hashimoto N, Nagasawa T, Usami M, Yamagata H, Takaesu Y, Miura K, Matsumoto J, Ohi K, Yamada H, Hori | Unfocused |

| | |
|---|---|
| H, Inada K, Watanabe K, Hashimoto R, Yasui-Furukori N. The effect of education regarding treatment guidelines for schizophrenia and major depressive disorders on psychiatrists' hypnotic medication prescribing behavior: a multicenter study. BMC Psychiatry. 2024;24(1):399. doi: 10.1186/s12888-024-05816-x. | |
| 372. Jia R, Coupland C, Vinogradova Y, Qureshi N, Turner E, Vedhara K. Mental health conditions and COVID-19 vaccine outcomes: A scoping review. J Psychosom Res. 2024;183:111826. doi: 10.1016/j.jpsychores.2024.111826. Epub 2024 Jun 8. | Review |
| 373. Rogdaki M, McCutcheon RA, D'Ambrosio E, Mancini V, Watson CJ, Fanshawe JB, Carr R, Telesia L, Martini MG, Philip A, Gilbert BJ, Salazar-de-Pablo G, Kyriakopoulos M, Siskind D, Correll CU, Cipriani A, Efthimiou O, Howes OD, Pillinger T. Comparative physiological effects of antipsychotic drugs in children and young people: a network meta-analysis. Lancet Child Adolesc Health. 2024;8(7):510-521. doi: 10.1016/S2352-4642(24)00098-1. | Review |
| 374. Kukkonen JP, Jacobson LH, Hoyer D, Rinne MK, Borgland SL. International Union of Basic and Clinical Pharmacology CXIV: Orexin receptor function, nomenclature and pharmacology. Pharmacol Rev. 2024;76(5):625-688. doi: 10.1124/pharmrev.123.000953. | Review |
| 375. Malafouris L, Röhricht F. Re-*thinging* embodied and enactive psychiatry: A material engagement approach. Cult Med Psychiatry. 2024;48(4):816-839. doi: 10.1007/s11013-024-09872-6. Epub 2024 Jul 19. | Opinion |
| 376. Wei S, Freeman D, Harris V, Rovira A. A randomised controlled test in virtual reality of the effects on paranoid thoughts of virtual humans' facial animation and expression. Sci Rep. 2024;14(1):17102. doi: 10.1038/s41598-024-67534-4. | No orexin |
| 377. Hong JSW, Ostinelli EG, Kamvar R, Smith KA, Walsh AEL, Kabir T, Tomlinson A, Cipriani A. An online evidence-based dictionary of common adverse events of antidepressants: a new tool to empower patients and clinicians in their shared decision-making process. BMC Psychiatry. 2024;24(1):532. doi: 10.1186/s12888-024-05950-6. | Unrelated |
| 378. Kikuchi Y, Kurosawa M, Sakata M, Takahashi Y, Yamamoto K, Tomita H, Yoshio T, Yasui-Furukori N. Effects of titration speed, gender, obesity and concomitant medications on the risk and onset time of clozapine-associated fever among Japanese patients with schizophrenia: retrospective review of charts from 21 hospitals. Br J Psychiatry. 2024;225(5):492-498. doi: 10.1192/bjp.2024.113. | Unfocused |
| 379. Cao F, Guo Z, Ma X, Li X, Wang Q. Regulation of neuronal plasticity associated with neuropsychiatric disorders by the orexinergic system. Heliyon. 2024;10(14):e34182. doi: 10.1016/j.heliyon.2024.e34182. | Review |
| 380. Ghazi-Noori AR, Woodham RD, Rezaei H, Sharif MS, Bramon E, Ritter P, Bauer M, Young AH, Fu CHY. Home-based transcranial direct current stimulation in bipolar depression: an open-label treatment study of clinical outcomes, acceptability and adverse events. Int J Bipolar Disord. 2024;12(1):30. doi: 10.1186/s40345-024-00352-9. | No orexin |
| 381. Dumont S, Bloch V, Lillo-Lelouet A, Le Beller C, Geoffroy PA, Veyrier M. Parasomnias and sleep-related movement disorders induced by drugs in the adult population: a review about iatrogenic medication effects. J Sleep Res. 2025;34(2):e14306. doi: 10.1111/jsr.14306. Epub 2024 Sep 7. | Review |
| 382. Xiao W, Moncy JC, Ghazi-Noori AR, Woodham RD, Rezaei H, Bramon E, Ritter P, Bauer M, Young AH, Fu CHY. Enhanced network synchronization connectivity following transcranial direct current stimulation (tDCS) in bipolar depression: Effects on EEG oscillations and deep learning-based predictors of clinical remission. J Affect Disord. 2025;369:576-587. doi: 10.1016/j.jad.2024.09.054. Epub 2024 Sep 16. | Unrelated |
| 383. Nozu T, Miyagishi S, Ishioh M, Takakusaki K, Okumura T. The neurotensin receptor 1 agonist PD149163 alleviates visceral hypersensitivity and colonic hyperpermeability in rat irritable bowel syndrome model. Neurogastroenterol Motil. 2024;36(12):e14925. doi: 10.1111/nmo.14925. Epub 2024 Sep 24. | Animal |
| 384. Guo J, Guo J, Rao X, Zhang R, Li Q, Zhang K, Ma S, Zhao J, Ji C. Exploring the pathogenesis of insomnia and acupuncture intervention strategies based on the microbiota-gut-brain axis. Front Microbiol. 2024;15:1456848. doi: 10.3389/fmicb.2024.1456848. | Review |
| 385. Vadisiute A, Meijer E, Therpurakal RN, Mueller M, Szabó F, Messore F, Jursenas A, Bredemeyer O, Krone LB, Mann E, Vyazovskiy V, Hoerder-Suabedissen A, Molnár Z. Glial cells undergo rapid changes following acute chemogenetic manipulation of cortical layer 5 projection neurons. Commun Biol. 2024;7(1):1286. doi: 10.1038/s42003-024-06994-w. | Animal |
| 386. Haubjerg Østerby NC, Baandrup L, Jennum PJ. Psychiatric comorbidity in Danish patients with narcolepsy type 1, narcolepsy type 2, and idiopathic hypersomnia: a case-control study. Sleep Adv. 2024;5(1):zpae073. doi: 10.1093/sleepadvances/zpae073. | No antipsyc |
| 387. Woodham RD, Selvaraj S, Lajmi N, Hobday H, Sheehan G, Ghazi-Noori AR, Lagerberg PJ, Rizvi M, Kwon SS, Orhii P, Maislin D, Hernandez L, Machado-Vieira R, Soares JC, Young AH, Fu CHY. Home-based transcranial direct current stimulation treatment for major depressive disorder: a fully remote phase 2 randomized sham-controlled trial. Nat Med. 2025;31(1):87-95. doi: 10.1038/s41591-024-03305-y. Epub 2024 Oct 21. | No orexin |
| 388. Mori K, Kimura M, Usami E. Short-term efficacy and safety of suvorexant and lemborexant: A retrospective study. Cureus. 2024;16(10):e71049. doi: 10.7759/cureus.71049. | Unfocused |
| 389. Vringer M, Bijlenga D, Zhou J, Meijer OC, Vinkers CH, Lammers GJ, Fronczek R. Physiological and psychological stress reactivity in narcolepsy type 1. Sleep. 2025;48(3):zsae265. doi: 10.1093/sleep/zsae265. | No psychosis |
| 390. Geldmacher DS. Treatment of Alzheimer disease. Continuum (Minneap Minn). 2024;30(6):1823-1844. doi: 10.1212/CON.0000000000001503. | Review |
| 391. Rezaei H, Woodham RD, Ghazi-Noori AR, Ritter P, Bramon E, Bauer M, Young AH, Fu CHY. Effect of home-based transcranial direct current stimulation (tDCS) on cognitive functioning in bipolar depression. Res Sq [Preprint]. 2024:rs.3.rs-5396838. doi: 10.21203/rs.3.rs-5396838/v1. Update in: Int J Bipolar Disord. 2025;13(1):11. doi: 10.1186/s40345-025-00376-9. | Dupl |
| 392. Chekani F, Mirchandani K, Zaki S, Goswami S, Sharma M. Utilization of potentially inappropriate sedative-hypnotic and atypical antipsychotic medications among elderly individuals with insomnia and Alzheimer's disease. Sleep. 2025;48(4):zsaf003. doi: 10.1093/sleep/zsaf003. | Unfocused |
| 393. Xie X, Xu H, Shu R, Du S, Fan H, Zhang M, Sun L, Zhou J, Wang L, Li Z, Anthony DC. Period3 modulates the $NAD^+$-SIRT3 axis to alleviate depression-like behaviour by enhancing NAMPT activity in mice. J Adv Res. 2025;77:309-320. doi: 10.1016/j.jare.2025.01.043. Epub 2025 Feb 1. | Animal |
| 394. Ferini-Strambi L. Insomnia disorder. Minerva Med. 2025;116(4):309-322. doi: 10.23736/S0026-4806.25.09690-9. Epub 2025 Feb 11. | Review |
| 395. Arnone D, Ramaraj R, Östlundh L, Arora T, Javaid S, Govender RD, Stip E, Young AH. Assessment of cognitive domains in major depressive disorders using the Cambridge Neuropsychological Test Automated Battery (CANTAB): Systematic review and meta-analysis of cross-sectional and longitudinal studies. Prog Neuropsychopharmacol Biol Psychiatry. 2025;138:111301. doi: 10.1016/j.pnpbp.2025.111301. Epub 2025 Feb 24. | No orexin |
| 396. Pozuelo Moyano B, Gomez Bautista D, Porras Ibarra KJ, Mueller C, von Gunten A, Vandel P, Ranjbar S, Howard R, Young AH, Stewart R, Reeves S, Orgeta V; European Task Force for treatment resistant depression in older people. Systematic review of clinical effectiveness of interventions for treatment resistant late-life depression. Ageing Res Rev. 2025;107:102710. doi: 10.1016/j.arr.2025.102710. Epub 2025 Feb 28. | Review |
| 397. Rezaei H, Woodham RD, Ghazi-Noori AR, Ritter P, Bramon E, Bauer M, Young AH, Fu CHY. Effect of home-based transcranial direct current stimulation (tDCS) on cognitive functioning in bipolar depression: an open-label, single-arm acceptability and feasibility study. Int J Bipolar Disord. 2025;13(1):11. doi: 10.1186/s40345-025-00376-9. | No orexin |
| 398. Nozu T, Miyagishi S, Ishioh M, Takakusaki K, Okumura T. Irisin prevents visceral hypersensitivity and colonic hyperpermeability in a rat model of irritable bowel syndrome. Peptides. 2025;188:171394. doi: 10.1016/j.peptides.2025.171394. Epub 2025 Mar 26. | Animal |
| 399. Woodham RD, Selvaraj S, Lajmi N, Hobday H, Sheehan G, Ghazi-Noori AR, Lagerberg PJ, Machado-Vieira R, Soares JC, Young AH, Fu CHY. Home-based transcranial direct current stimulation for major depressive disorder: 6-month follow-up from randomised sham-controlled trial and open-label treatment phases. J Psychiatr Res. 2025;186:23-32. doi: 10.1016/j.jpsychires.2025.03.047. Epub 2025 Mar 26. | No orexin |

| | |
|---|---|
| 400. Rezaei H, Woodham RD, Ghazi-Noori AR, Ritter P, Bauer M, Young AH, Bramon E, Fu CHY. Acceptability of Home-Based Transcranial Direct Current Stimulation (tDCS) in Bipolar Depression: Thematic Analysis of Individual Views. Res Sq [Preprint]. 2025 Apr 15:rs.3.rs-5967699. doi: 10.21203/rs.3.rs-5967699/v1. Update in: BMC Psychiatry. 2025;25(1):549. doi: 10.1186/s12888-025-06948-4. | Dupl |
| 401. Attaallah B, Petitet P, Husain M. Active information sampling in health and disease. Neurosci Biobehav Rev. 2025;175:106197. doi: 10.1016/j.neubiorev.2025.106197. Epub 2025 May 3. | Review |
| 402. Beech MJ, Toma EC, Smith HG, Trush MM, Ang JHJ, Wong MY, Wong CHJ, Ali HS, Butt Z, Goel V, Duarte F, Farley AJM, Walsh TR, Schofield CJ. Binding assays enable discovery of Tet(X) inhibitors that combat tetracycline destructase resistance. Chem Sci. 2025;16(22):9691-9704. doi: 10.1039/d5sc00964b. | *In vitro* |
| 403. Rezaei H, Woodham RD, Ghazi-Noori AR, Ritter P, Bauer M, Young AH, Bramon E, Fu CHY. Acceptability of home-based transcranial direct current stimulation (tDCS) in bipolar depression: thematic analysis of individual views. BMC Psychiatry. 2025;25(1):549. doi: 10.1186/s12888-025-06948-4. | No orexin |
| 404. Beckley A, Glogowska M, Waite F, Bee P, Freeman D. Research assistants' experiences recruiting patients with psychosis into clinical trials: a qualitative study. Trials. 2025;26(1):180. doi: 10.1186/s13063-025-08882-y. | No orexin |
| 405. Jenner L, Payne M, Waite F, Beckwith H, Diamond R, Isham L, Collett N, Emsley R, Freeman D. Learning how to improve the treatment of persecutory delusions: Using a principal trajectories analysis to examine differential effects of two psychological interventions (Feeling Safe, Befriending) in distinct groups of patients. Schizophr Bull. 2026;52:sbaf083. doi: 10.1093/schbul/sbaf083. Epub ahead of print 2025 Jun 17. | No orexin |
| 406. Mori K, Ohashi K, Kimura M, Yoshida M, Tomida K, Usami E (森 光輝，大橋健吾，木村美智男，吉田光代，富田顕旨，宇佐美英績). オレキシン受容体拮抗薬推進活動が睡眠薬・抗精神病薬の処方動向に与える影響：分割時系列解析研究 [Impact of promotional activities on orexin receptor antagonists prescription rates and usage of sleep and antipsychotic medications: An interrupted time-series analysis study]. Yakugaku Zasshi. 2025;145(7):629-637. Japanese. doi: 10.1248/yakushi.24-00170. | Unfocused |
| 407. Takahashi K, Kiryu K, Harada H, Kato T, Tamune H. Management of subsyndromal delirium with daridorexant and quetiapine: A case report. Cureus. 2025;17(6):e85232. doi: 10.7759/cureus.85232. | Case |
| 408. Liaskopoulos A, Kakouris V, Liaskopoulos N, Lappas AS, Christodoulou N, Samara M. Medical professionals and pharmacological intervention for the treatment of insomnia: A cross-sectional study. Sleep Sci. 2024;18(2):e155-e164. doi: 10.1055/s-0044-1791238. | Unfocused |
| 409. Berger M, Helter T, Azim L, Chadwick T, Courtney P, Fouweather T, Geddes J, Hindmarch P, Morriss R, Stokes PRA, Watson S, Weetman C, Young AH, McAllister-Williams RH, Simon J. Cost-effectiveness of pramipexole in addition to mood stabilisers for patients with treatment-resistant bipolar depression: Economic evaluation of the PAX-BD randomised controlled trial. J Affect Disord. 2025;391:119937. doi: 10.1016/j.jad.2025.119937. Epub 2025 Jul 18. | No orexin |
| 410. Jelen LA, Lythgoe DJ, Stone JM, Young AH, Mehta MA. Effect of naltrexone pretreatment on ketamine-induced glutamatergic activity and symptoms of depression: a randomized crossover study. Nat Med. 2025;31(9):2958-2966. doi: 10.1038/s41591-025-03800-w. Epub 2025 Jul 24. | No orexin |
| 411. Kim TH, Lee K, Hwang J, Lee S, Jo H, Cho H, Lee H, Baek HJ, Kang J, Nehs CJ, Fond G, Boyer L, Chung EK, Yon DK. Adverse event reports of seizure for insomnia medication from 1967 to 2023. Sci Rep. 2025;15(1):27308. doi: 10.1038/s41598-025-11314-1. | Unfocused |
| 412. Rezaei H, Woodham RD, Ghazi-Noori AR, Bramon E, Bauer M, Young AH, Fu CHY, Ritter P. Home-based transcranial direct current stimulation (tDCS) for bipolar depression: Effects on quality of life and functioning: an open-label study. Res Sq [Preprint]. 2025:rs.3.rs-7186400. doi: 10.21203/rs.3.rs-7186400/v1. | Dupl |
| 413. Imran R, Kenny A, Wong G, Lee C. The role of link workers in weight management for people with severe mental illness: a qualitative study. BMC Prim Care. 2025;26(1):251. doi: 10.1186/s12875-025-02929-4. | Unrelated |
| 414. Jauhar S, McCutcheon RA, Nour MM, Veronese M, Rogdaki M, Bonoldi I, Azis M, Whitehurst T, Arumuham A, Onwordi E, Turkheimer F, McGuire P, Young AH, Howes OD. Dopamine and mood in psychotic disorders: An $^{18}$F-DOPA PET study. JAMA Psychiatry. 2025;82(10):1009-1014. doi: 10.1001/jamapsychiatry.2025.1811. Epub ahead of print 2025 Aug 13. | No orexin |
| 415. Todd KL, Cramb KML, Brimblecombe KR, Cragg SJ. New insights into axonal regulators of dopamine transmission in health and disease. Curr Opin Neurobiol. 2025;94:103093. doi: 10.1016/j.conb.2025.103093. Epub 2025 Aug 16. | Review |
| 416. Nedeljkovic-Kurepa A, Abraham MN, Fernandes TD, Yaipen O, Brewer MR, Taylor MD, Pavlov VA, Deutschman CS. Loss of M1 Acetylcholine receptor-mediated orexinergic activity contributes to immune dysfunction in experimental sepsis. Res Sq [Preprint]. 2025:rs.3.rs-7329263. doi: 10.21203/rs.3.rs-7329263/v1. | Animal |
| 417. Yamaguchi J, Sadahiro R, Wada S, Nishikawa E, Terada T, Nakahara R, Matsuoka H. Delirium derived from dementia with Lewy bodies in the cancer perioperative period: a case report. Ann Palliat Med. 2025;14(5):508-513. doi: 10.21037/apm-25-48. | Case |
| 418. Chinoy J, Meller C, de Wet H. An emerging paradigm for ABCC5/MRP5 function in human physiology. Int J Mol Sci. 2025;26(18):9211. doi: 10.3390/ijms26189211. | Review |
| 419. Volgin AD, Cheresiz SV, Chizhova ND, Smirnova KV, Doroshkov AV, Galstya DS, Abreu MS, Strekalova T, Lipina T, Pletnikov M, Yang L, Lim LW, Stewart AM, Amstislavskaya TG, Kalueff AV. Rethinking the role of *DISC1* in CNS function: Translational cross-taxon insights from rodent and zebrafish models. J Integr Neurosci. 2025;24(9):43162. doi: 10.31083/JIN43162. | Review |
| 420. Nutt DJ, Morgan C, Erritzoe D, Greenway KT, Young AH. A long, strange trip: Ketamine treatment in psychiatry. J Psychopharmacol. 2025;39(10):1039-1044. doi: 10.1177/02698811251379393. Epub 2025 Oct 27. | Opinion |
| 421. Arnone D, Östlundh L, Mosa M, MacDonald B, Oldershaw J, Qassem T, Young AH. Efficacy of lamotrigine in the treatment of unipolar and bipolar depression: Meta-analysis of acute and maintenance randomised controlled trials. Pharmaceuticals (Basel). 2025;18(10):1590. doi: 10.3390/ph18101590. | Review |
| 422. Højlund M, Kafali HY, Kırmızı B, Fusar-Poli P, Correll CU, Cortese S, Sabé M, Fiedorowicz J, Saraf G, Zein J, Berk M, Husain MI, Rosenblat JD, Rubaiyat R, Corace K, Wong S, Hatcher S, Kaluzienski M, Yatham LN, Cipriani A, Gosling CJ, Carhart-Harris R, Tanuseputro P, Myran DT, Fabiano N, Moher D, Mayo LM, Nicholls SG, White T, Prisco M, Radua J, Vieta E, Ladha KS, Katz J, Veroniki AA, Solmi M. Efficacy, all-cause discontinuation, and safety of serotonergic psychedelics and MDMA to treat mental disorders: A living systematic review with meta-analysis. Eur Neuropsychopharmacol. 2025;101:41-55. doi: 10.1016/j.euroneuro.2025.09.011. Epub 2025 Nov 7. | Review |
| 423. Ma W, Warnhoff I, Stephan M, Ma X, Dehne K, Volkmann P, Kannaiyan N, Brankatschk B, Jensen N, Rossner MJ, Scheuss V, Wehr MC. TAOK2 controls synaptic plasticity and anxiety via ERK and calcium signaling. iScience. 2025;28(11):113712. doi: 10.1016/j.isci.2025.113712. | Animal |
| 424. Kono S, Nishiyama T, Nakano S, Ishimoto Y, Horikoshi S. Real-world effectiveness and safety of daridorexant in Japanese patients with insomnia: A multicenter observational study evaluating four-week changes in Athens Insomnia Scale among 54 participants. Cureus. 2025;17(11):e97174. doi: 10.7759/cureus.97174. | No psychosis |
| 425. Ryan S, Munir S, Davies A. Pharmacological interventions for sleep disturbance ("insomnia") in patients with advanced cancer receiving specialist palliative care: a scoping review. Support Care Cancer. 2025;33(12):1124. doi: 10.1007/s00520-025-10202-8. | Review |
| 426. Dong X, Aveyard P, Yang X, Kivimaki M, Chen S, Firth J, Drakesmith CW, Gao M. Adiposity and the first-onset of diagnosed mental illnesses: a population-based cohort study of 10 million UK adults. BMC Med. 2025. doi: 10.1186/s12916-025-04514-z. Epub ahead of print 2025 Nov 29. | No orexin |
| **427. Tanaka A, Arai Y, Yasaki T, Saito K, Nakashizuka E, Yoshida S, Nakajima Y, Koido M, Suzuki K, Nakamura T, Sasayama D, Washizuka S. Longitudinal associations of serum orexin-A with physical activity and sleep in schizophrenia: A preliminary study. Neuropsychopharmacol Rep. 2025;45(4):e70079. doi: 10.1002/npr2.70079.** | **Included** |

| | |
|---|---|
| 428. Lagerberg PJ, Woodham RD, Selvaraj S, Lajmi N, Hobday H, Sheehan G, Ghazi-Noori AR, Rizvi M, Kwon SS, Orhii P, Machado-Vieira R, Soares JC, Young AH, Fidalgo AR, Rezaei H, Fu CHY. Acceptability of active and sham home-based transcranial direct current stimulation in major depression: mixed methods qualitative analysis in a randomised controlled trial. Ann Gen Psychiatry. 2026;25(1):1. doi: 10.1186/s12991-025-00607-4. Epub ahead of print 2025 Dec 4. | No orexin |
| 429. Willard J, Velez D, Baez CS, Palladino C, Pavkovic I, Varughese RT, Kothare SV. Tactile hallucinations in pediatric and young adults with narcolepsy: A case series and review of the literature. Pediatr Neurol. 2025;175:151-155. doi: 10.1016/j.pediatrneurol.2025.11.009. Epub ahead of print 2025 Nov 14. | Case |
| 430. Diamond R, Waite F, Boylan AM, Hicks A, Kabir T, Freeman D. Supporting patients with psychosis in the community to stand up and move more: Perspectives of community mental health staff. Community Ment Health J. 2025. doi: 10.1007/s10597-025-01563-9. Epub ahead of print 2025 Dec 27. | Unrelated |
| 431. Moncy JC, Xiao W, Woodham RD, Ghazi-Noori AR, Rezaei H, Bramon E, Ritter P, Bauer M, Young AH, Fan Y, Fu CHY. Deep learning based treatment remission prediction to transcranial direct current stimulation in bipolar depression using EEG power spectral density. Psychiatry Res Neuroimaging. 2026;357:112115. doi: 10.1016/j.pscychresns.2025.112115. Epub ahead of print 2025 Dec 22. | No orexin |
| 432. Rezaei H, Woodham RD, Ghazi-Noori AR, Bramon E, Bauer M, Young AH, Fu CHY, Ritter P. Home-based transcranial direct current stimulation (tDCS) for bipolar depression: effects on quality of life and functioning-an open-label study. Qual Life Res. 2026;35(2):33. doi: 10.1007/s11136-025-04135-2. | Unrelated |
| 433. Hampsey E, Kalfas M, Carter L, Bloomfield M, Rezaei H, Young AH, Fu CHY. A systematic review of transcranial electrical stimulation and meta-analysis of transcranial direct current stimulation RCTs in unipolar and bipolar depression. J Affect Disord. 2026;400:121009. doi: 10.1016/j.jad.2025.121009. Epub ahead of print 2026 Jan 9. | Review |
| 434. Almahwzi S, Ghabashi S, Alyami W, Alsolami J, Juma R, Alghamdi S, Althobyane L, Alqahtani M, Alotaibi M. Circulating orexin-A levels in patients with schizophrenia, bipolar disorder, and major depressive disorder: A systematic review and meta-analysis. Cureus. 2026;18(1):e101525. doi: 10.7759/cureus.101525. | Review |
| 435. Kas MJH, Do KQ, Sand MS, Kozak R, Tunbridge EM, Oquendo MA, Tamminga C, Koutsouleris N, Knudsen GM, Penninx BWJH, Padberg FJ, Drevets WC, Falkai P, Buhl DL, Reif A. Biomarker innovations in precision psychiatry diagnostics and treatment strategies. Eur Neuropsychopharmacol. 2026;105:112762. doi: 10.1016/j.euroneuro.2026.112762. Epub ahead of print 2026 Jan 15. | Opinion |
| 436. De Zoysa AI, Govinnage J, Giorlando F, Dodd S, Narayanaswamy JC, Berk M. Emerging neurobiological targets in psychiatric treatment. Eur Neuropsychopharmacol. 2026;105:112767. doi: 10.1016/j.euroneuro.2026.112767. Epub ahead of print 2026 Jan 27. | Review |
| 437. Burckhardt A, Rakowsky A, Kahl E, Morchhale S, Mayer D, Panagiotou N, Permien L, Faesel N, Fendt M. Differential effects of orexin system activation on dizocilpine-induced schizophrenia-like behaviors in mice. Neuropeptides. 2026;116:102587. doi: 10.1016/j.npep.2026.102587. Epub ahead of print 2026 Jan 28. | Animal |
| 438. Shimohara Y, Matsuda T, Iwata K. Effects of dual orexin receptor antagonists on seizure quality in modified electroconvulsive therapy: A pilot study. J ECT. 2026. doi: 10.1097/YCT.0000000000001223. Epub ahead of print 2026 Feb 4. | Unfocused |
| 439. Hyndych A, Koval K, Dzeruzhynska N, Mader EC. Sleep and psychiatric disorders: Bidirectional interactions and shared neurobiological mechanisms. PLoS Ment Health. 2025;2(12):e0000531. doi: 10.1371/journal.pmen.0000531. | Review |
| 440. Gnazzo M, Pisanò G, Baldini V, Citeroni F, Canulli F, De Ronchi D, Pizza F, Plazzi G. Unravelling narcolepsy: A series of complex pediatric cases. Neurol Clin Pract. 2026;16(2):e200583. doi: 10.1212/CPJ.0000000000200583. Epub 2026 Jan 28. | Case |
| 441. von Fabeck K, Boudsocq JP, Boye M; French PCC Research Group; Simon N. Clinical toxicity of daridorexant: a retrospective analysis of poison centre data. Clin Toxicol (Phila). 2026:1-6. doi: 10.1080/15563650.2026.2617481. Epub ahead of print Feb 17. | No psychosis |
| 442. Telegdy G, Adamik A. The action of orexin A on passive avoidance learning. Involvement of transmitters. Regul Pept. 2002;104(1-3):105-10. doi: 10.1016/s0167-0115(01)00341-x. | Dupl |
| 443. Monda M, Viggiano A, De Luca V. Haloperidol reduces the sympathetic and thermogenic activation induced by orexin A. Neurosci Res. 2003;45(1):17-23. doi: 10.1016/s0168-0102(02)00191-8. | Dupl |
| 444. Winsky-Sommerer R, Boutrel B, De Lecea L. The role of the hypocretinergic system in the integration of networks that dictate the states of arousal. Drug News Perspect. 2003;16(8):504-12. doi: 10.1358/dnp.2003.16.8.829349. | Review |
| 445. Jandacek RJ, Woods SC. Pharmaceutical approaches to the treatment of obesity. Drug Discov Today. 2004;9(20):874-80. doi: 10.1016/S1359-6446(04)03244-1. | Review |
| 446. Rasmussen K, Benvenga MJ, Bymaster FP, Calligaro DO, Cohen IR, Falcone JF, Hemrick-Luecke SK, Martin FM, Moore NA, Nisenbaum LK, Schaus JM, Sundquist SJ, Tupper DE, Wiernicki TR, Nelson DL. Preclinical pharmacology of FMPD [6-fluoro-10-[3-(2-methoxyethyl)-4-methyl-piperazin-1-yl]-2-methyl-4H-3-thia-4,9-diaza-benzo[f]azulene]: a potential novel antipsychotic with lower histamine H1 receptor affinity than olanzapine. J Pharmacol Exp Ther. 2005;315(3):1265-77. doi: 10.1124/jpet.105.089326. Epub 2005 Sep 1. | Dupl |
| 447. Alexander SP, Mathie A, Peters JA. Guide to receptors and channels, 1st edition (2005 revision). Br J Pharmacol. 2005;144(Suppl 1):S1-128. doi: 10.1038/sj.bjp.0706158. | Review |
| 448. Bubser M, Fadel JR, Jackson LL, Meador-Woodruff JH, Jing D, Deutch AY. Dopaminergic regulation of orexin neurons. Eur J Neurosci. 2005;21(11):2993-3001. doi: 10.1111/j.1460-9568.2005.04121.x. | Dupl |
| 449. Narita M, Nagumo Y, Hashimoto S, Narita M, Khotib J, Miyatake M, Sakurai T, Yanagisawa M, Nakamachi T, Shioda S, Suzuki T. Direct involvement of orexinergic systems in the activation of the mesolimbic dopamine pathway and related behaviors induced by morphine. J Neurosci. 2006;26(2):398-405. doi: 10.1523/JNEUROSCI.2761-05.2006. | Dupl |
| 450. Steffen KJ, Roerig JL, Mitchell JE, Uppala S. Emerging drugs for eating disorder treatment. Expert Opin Emerg Drugs. 2006;11(2):315-36. doi: 10.1517/14728214.11.2.315. | Dupl |
| 451. Strachan RT, Ferrara G, Roth BL. Screening the receptorome: an efficient approach for drug discovery and target validation. Drug Discov Today. 2006;11(15-16):708-16. doi: 10.1016/j.drudis.2006.06.012. | Review |
| 452. Alberto CO, Trask RB, Quinlan ME, Hirasawa M. Bidirectional dopaminergic modulation of excitatory synaptic transmission in orexin neurons. J Neurosci. 2006;26(39):10043-50. doi: 10.1523/JNEUROSCI.1819-06.2006. Retraction in: J Neurosci. 2012;32(26):9116. doi: 10.1523/JNEUROSCI.1889-12.2012. | Dupl |
| 453. Rasmussen K, Hsu MA, Yang Y. The orexin-1 receptor antagonist SB-334867 blocks the effects of antipsychotics on the activity of A9 and A10 dopamine neurons: implications for antipsychotic therapy. Neuropsychopharmacology. 2007;32(4):786-92. doi: 10.1038/sj.npp.1301239. Epub 2006 Oct 25. | Dupl |
| 454. Rasmussen K, Hsu MA, Noone S, Johnson BG, Thompson LK, Hemrick-Luecke SK. The orexin-1 antagonist SB-334867 blocks antipsychotic treatment emergent catalepsy: implications for the treatment of extrapyramidal symptoms. Schizophr Bull. 2007;33(6):1291-7. doi: 10.1093/schbul/sbm087. Epub 2007 Jul 28. | Dupl |
| 455. Deutch AY, Bubser M. The orexins/hypocretins and schizophrenia. Schizophr Bull. 2007;33(6):1277-83. doi: 10.1093/schbul/sbm096. Epub 2007 Aug 28. | Dupl |
| 456. Sullivan SS, Guilleminault C. Emerging drugs for insomnia: new frontiers for old and novel targets. Expert Opin Emerg Drugs. 2009;14(3):411-22. doi: 10.1517/14728210903171948. | Review |
| 457. Eriksson KS, Sergeeva OA, Haas HL, Selbach O. Orexins/hypocretins and aminergic systems. Acta Physiol (Oxf). 2010;198(3):263-75. doi: 10.1111/j.1748-1716.2009.02015.x. Epub 2009 Jun 27. | Review |

| | |
|---|---|
| 458. Suzuki G, Satow A, Ohta H. Effect of CFMTI, an allosteric metabotropic glutamate receptor 1 antagonist with antipsychotic activity, on Fos expression in regions of the brain related to schizophrenia. Neuroscience. 2010;168(3):787-96. doi: 10.1016/j.neuroscience.2010.04.016. Epub 2010 Apr 18. | Dupl |
| 459. Milella MS, Passarelli F, De Carolis L, Schepisi C, Nativio P, Scaccianoce S, Nencini P. Opposite roles of dopamine and orexin in quinpirole-induced excessive drinking: a rat model of psychotic polydipsia. Psychopharmacology (Berl). 2010;211(3):355-66. doi: 10.1007/s00213-010-1909-5. Epub 2010 Jun 16. | Dupl |
| 460. Perna G, Guerriero G, Caldirola D. Emerging drugs for panic disorder. Expert Opin Emerg Drugs. 2011;16(4):631-45. doi: 10.1517/14728214.2011.628313. Epub 2011 Oct 17. | Review |
| 461. Ioachimescu OC, El-Solh AA. Pharmacotherapy of insomnia. Expert Opin Pharmacother. 2012;13(9):1243-60. doi: 10.1517/14656566.2012.683860. Epub 2012 May 11. | Dupl |
| 462. Sullivan S. Update on emerging drugs for insomnia. Expert Opin Emerg Drugs. 2012;17(3):295-8. doi: 10.1517/14728214.2012.693158. | Opinion |
| 463. Taslimi Z, Arezoomandan R, Omranifard A, Ghalandari-Shamami M, Riahi E, Vafaei AA, Rashidy-Pour A, Haghparast A. Orexin A in the ventral tegmental area induces conditioned place preference in a dose-dependent manner: involvement of D1/D2 receptors in the nucleus accumbens. Peptides. 2012;37(2):225-32. doi: 10.1016/j.peptides.2012.07.023. Epub 2012 Aug 3. | Dupl |
| 464. Katwala J, Kumar AK, Sejpal JJ, Terrence M, Mishra M. Therapeutic rationale for low dose doxepin in insomnia patients. Asian Pac J Trop Dis. 2013;3(4):331-6. doi: 10.1016/S2222-1808(13)60080-8. | Review |
| 465. Girault EM, Foppen E, Ackermans MT, Fliers E, Kalsbeek A. Central administration of an orexin receptor 1 antagonist prevents the stimulatory effect of Olanzapine on endogenous glucose production. Brain Res. 2013;1527:238-45. doi: 10.1016/j.brainres.2013.06.034. Epub 2013 Jul 4. | Dupl |
| 466. LaCrosse AL, Olive MF. Neuropeptide systems and schizophrenia. CNS Neurol Disord Drug Targets. 2013;12(5):619-32. doi: 10.2174/1871527311312050010. | Dupl |
| 467. Haghparast A, Omranifard A, Arezoomandan R, Ghalandari-Shamami M, Taslimi Z, Vafaei AA, Rashidy-Pour A. Involvement of dopaminergic receptors of the rat nucleus accumbens in decreasing the conditioned place preference induced by lateral hypothalamus stimulation. Neurosci Lett. 2013;556:10-4. doi: 10.1016/j.neulet.2013.09.062. Epub 2013 Oct 5. | Dupl |
| 468. Palotai M, Telegdy G, Jászberényi M. Orexin A-induced anxiety-like behavior is mediated through GABA-ergic, α- and β-adrenergic neurotransmissions in mice. Peptides. 2014;57:129-34. doi: 10.1016/j.peptides.2014.05.003. Epub 2014 May 27. | Dupl |
| 469. Chen YW, Morganstern I, Barson JR, Hoebel BG, Leibowitz SF. Differential role of D1 and D2 receptors in the perifornical lateral hypothalamus in controlling ethanol drinking and food intake: possible interaction with local orexin neurons. Alcohol Clin Exp Res. 2014;38(3):777-86. doi: 10.1111/acer.12313. Epub 2013 Nov 15. | Dupl |
| 470. Palotai M, Telegdy G, Ekwerike A, Jászberényi M. The action of orexin B on passive avoidance learning. Involvement of neurotransmitters. Behav Brain Res. 2014;272:1-7. doi: 10.1016/j.bbr.2014.06.016. Epub 2014 Jun 13. | Dupl |
| 471. Yazdi-Ravandi S, Razavi Y, Haghparast A, Goudarzvand M, Haghparast A. Orexin A induced antinociception in the ventral tegmental area involves D1 and D2 receptors in the nucleus accumbens. Pharmacol Biochem Behav. 2014;126:1-6. doi: 10.1016/j.pbb.2014.08.009. Epub 2014 Aug 30. | Dupl |
| 472. Szabo ST, Kinon BJ, Brannan SK, Krystal AK, van Gerven JM, Mahableshwarkar A, Sachs GS. Lessons learned and potentials for improvement in CNS drug development: ISCTM Section on Designing the right series of experiments. Innov Clin Neurosci. 2015;12(3Suppl A):11S-25S. | Opinion |
| 473. Uto Y. 1,2-Benzisoxazole compounds: a patent review (2009 - 2014). Expert Opin Ther Pat. 2015;25(6):643-62. doi: 10.1517/13543776.2015.1027192. Epub 2015 Mar 23. | Review |
| 474. Shan L, Dauvilliers Y, Siegel JM. Interactions of the histamine and hypocretin systems in CNS disorders. Nat Rev Neurol. 2015;11(7):401-13. doi: 10.1038/nrneurol.2015.99. Epub 2015 Jun 23. | Review |
| 475. Li A-J, Wang Q, Elsarelli MM, Brown RL, Ritter S. Hindbrain catecholamine neurons activate orexin neurons during systemic glucoprivation in male rats. Endocrinology. 2015;156(8):2807-20. doi: 10.1210/en.2015-1138. Epub 2015 May 15. | Dupl |
| 476. Okumura T, Nozu T, Kumei S, Takakusaki K, Miyagishi S, Ohhira M. Involvement of the dopaminergic system in the central orexin-induced antinociceptive action against colonic distension in conscious rats. Neurosci Lett. 2015;605:34-8. doi: 10.1016/j.neulet.2015.08.013. Epub 2015 Aug 12. | Dupl |
| 477. Vickers SP, Hackett D, Murray F, Hutson PH, Heal DJ. Effects of lisdexamfetamine in a rat model of binge-eating. J Psychopharmacol. 2015;29(12):1290-307. doi: 10.1177/0269881115615107. Epub 2015 Nov 20. | Dupl |
| 478. Krystal AD. New developments in insomnia medications of relevance to mental health disorders. Psychiatr Clin North Am. 2015;38(4):843-60. doi: 10.1016/j.psc.2015.08.001. Epub 2015 Sep 11. | Dupl |
| 479. Okumura T, Nozu T, Kumei S, Takakusaki K, Miyagishi S, Ohhira M. Levodopa acts centrally to induce an antinociceptive action against colonic distension through activation of D2 dopamine receptors and the orexinergic system in the brain in conscious rats. J Pharmacol Sci. 2016;130(2):123-7. doi: 10.1016/j.jphs.2016.01.007. Epub 2016 Jan 29. | Dupl |
| 480. Clifton PG. Neural circuits of eating behaviour: Opportunities for therapeutic development. J Psychopharmacol. 2017;31(11):1388-1402. doi: 10.1177/0269881117738629. Epub 2017 Nov 14. | Review |
| 481. Atkin T, Comai S, Gobbi G. Drugs for insomnia beyond benzodiazepines: Pharmacology, clinical applications, and discovery. Pharmacol Rev. 2018;70(2):197-245. doi: 10.1124/pr.117.014381. | Dupl |
| 482. Okumura T, Nozu T, Kumei S, Takakusaki K, Ohhira M. Ghrelin acts centrally to induce an antinociceptive action during colonic distension through the orexinergic, dopaminergic and opioid systems in conscious rats. Brain Res. 2018;1686:48-54. doi: 10.1016/j.brainres.2018.02.024. Epub 2018 Feb 21. | Dupl |
| 483. Heiss JE, Yamanaka A, Kilduff TS. Parallel arousal pathways in the lateral hypothalamus. eNeuro. 2018;5(4):ENEURO.0228-18.2018. doi: 10.1523/ENEURO.0228-18.2018. | Dupl |
| 484. Liu C, Xue Y, Liu MF, Wang Y, Liu ZR, Diao HL, Chen L. Orexins increase the firing activity of nigral dopaminergic neurons and participate in motor control in rats. J Neurochem. 2018;147(3):380-394. doi: 10.1111/jnc.14568. Epub 2018 Oct 15. | Dupl |
| 485. Linehan V, Rowe TM, Hirasawa M. Dopamine modulates excitatory transmission to orexin neurons in a receptor subtype-specific manner. Am J Physiol Regul Integr Comp Physiol. 2019;316(1):R68-R75. doi: 10.1152/ajpregu.00150.2018. Epub 2018 Nov 21. | Dupl |
| 486. Okumura T, Nozu T, Kumei S, Ohhira M. Central oxytocin signaling mediates the central orexin-induced visceral antinociception through the opioid system in conscious rats. Physiol Behav. 2019;198:96-101. doi: 10.1016/j.physbeh.2018.10.007. Epub 2018 Oct 18. | Dupl |
| 487. Hanazawa T, Kamijo Y. Effect of suvorexant on nocturnal delirium in elderly patients with Alzheimer's disease: A case-series study. Clin Psychopharmacol Neurosci. 2019;17(4):547-550. doi: 10.9758/cpn.2019.17.4.547. | Dupl |
| 488. Dunn KE, Huhn AS, Bergeria CL, Gipson CD, Weerts EM. Non-opioid neurotransmitter systems that contribute to the opioid withdrawal syndrome: A review of preclinical and human evidence. J Pharmacol Exp Ther. 2019;371(2):422-452. doi: 10.1124/jpet.119.258004. Epub 2019 Aug 7. | Review |
| 489. Magdaleno-Madrigal VM, Morales-Mulia S, Nicolini H, Genis-Mendoza A, Cázares-Martínez Claudia E, Pérez-Luna José M, Morales-Mulia M. Orexin-A promotes EEG changes but fails to induce anxiety in rats. Behav Brain Res. 2019;361:26-31. doi: 10.1016/j.bbr.2018.12.037. Epub 2018 Dec 21. | Dupl |

| | |
|---|---|
| 490. Naghavi FS, Namvar P, Sadeghzadeh F, Haghparast A. The involvement of intra-hippocampal dopamine receptors in the conditioned place preference induced by orexin administration into the rat ventral tegmental area. Iran J Pharm Res. 2019;18(1):328-338. | Dupl |
| 491. Lebedev AA, Bessolova YN, Efimov NS, Bychkov ER, Droblenkov AV, Shabanov PD. Role of orexin peptide system in emotional overeating induced by brain reward stimulation in fed rats. Res Results Pharmacol. 2020;6(1):81-91. doi: 10.3897/rrpharmacology.6.52180. | Animal |
| 492. Matini T, Haghparast A, Rezaee L, Salehi S, Tehranchi A, Haghparast A. Role of dopaminergic receptors within the ventral tegmental area in antinociception induced by chemical stimulation of the lateral hypothalamus in an animal model of orofacial pain. J Pain Res. 2020;13:1449-1460. doi: 10.2147/JPR.S255250. | Dupl |
| 493. Dujardin S, Pijpers A, Pevernagie D. Prescription drugs used in insomnia. Sleep Med Clin. 2020;15(2):133-145. doi: 10.1016/j.jsmc.2020.02.002. | Dupl |
| 494. Lebedev AA, Bessolova YN, Efimov NS, Rusanovskii VV, Shabanov PD. Lateral hypothalamic self-stimulation with threshold current intensity induces emotional overeating in self-deprivation paradigm in well-fed rats: Role of orexin and dopaminergic systems of the brain. Reviews on Clinical Pharmacology and Drug Therapy, 19(4):421-429. | Animal |
| 495. Pennington S, Stutzman D, Sannar E. Pitolisant in an adolescent with Prader-Willi syndrome. J Pediatr Pharmacol Ther. 2021;26(4):405-410. doi: 10.5863/1551-6776-26.4.405. Epub 2021 May 19. | Case |
| 496. Borgland SL. Can treatment of obesity reduce depression or vice versa? J Psychiatry Neurosci. 2021;46(2):E313-E318. doi: 10.1503/jpn.210036. | Opinion |
| 497. Joshi R, Bansal S, Malik D, Singla R, Mishra A, Prakash A, Medhi B. Computational modeling of ACE2 inhibitors for development of drugs against coronaviruses. In Roy K (Ed.) In Silico Modeling of Drugs Against Coronaviruses: Computational Tools and Protocols. Methods in Pharmacology and Toxicology, Springer Protocols. Totowa, New Jersey: Humana Press, 2021; pp. 615–629. doi: 10.1007/7653_2020_71. ISBN: 1071613677, 9781071613672. | Unrelated |
| 498. Nigam M, Leu-Semenescu S, Arnulf I. Successful treatment of drug-resistant cataplexy with the anticholinergic drug tropatepine. J Clin Sleep Med. 2021;17(4):849-851. doi: 10.5664/jcsm.9030. | Dupl |
| 499. Greenwald MK, Moses TEH, Roehrs TA. At the intersection of sleep deficiency and opioid use: mechanisms and therapeutic opportunities. Transl Res. 2021;234:58-73. doi: 10.1016/j.trsl.2021.03.006. Epub 2021 Mar 9. | Review |
| 500. Nasrollahi S, Karimi S, Hamidi G, Naderitehrani M, Abed A. Blockade of the orexin 1 receptors in the nucleus accumbens' shell reversed the reduction effect of olanzapine on motivation for positive reinforcers. Neurosci Lett. 2021;762:136137. doi: 10.1016/j.neulet.2021.136137. Epub 2021 Jul 24. | Dupl |
| 501. Hong J, Vernon D, Kunovac J, Stahl S. Emerging drugs for the treatment of major depressive disorder. Expert Opin Emerg Drugs. 2022;27(3):263-275. doi: 10.1080/14728214.2022.2117297. Epub 2022 Sep 20. | Dupl |
| 502. Berger AA, Sottosanti ER, Winnick A, Keefe J, Gilbert E, Hasoon J, Thase ME, Kaye AD, Viswanath O, Urits I. Suvorexant in the treatment of difficulty falling and staying asleep (insomnia). Psychopharmacol Bull. 2022;52(1):68-90. doi: 10.64719/pb.4429. | Review |
| 503. Koob GF. Anhedonia, hyperkatifeia, and negative reinforcement in substance use disorders. Curr Top Behav Neurosci. 2022;58:147-165. doi: 10.1007/7854_2021_288. | Review |
| 504. Benca R, Herring WJ, Khandker R, Qureshi ZP. Burden of Insomnia and Sleep Disturbances and the Impact of Sleep Treatments in Patients with Probable or Possible Alzheimer's Disease: A Structured Literature Review. J Alzheimers Dis. 2022;86(1):83-109. doi: 10.3233/JAD-215324. | Review |
| 505. Terada T, Hirayama T, Sadahiro R, Wada S, Nakahara R, Matsuoka H. Pilot study of lemborexant for insomnia in cancer patients with delirium. J Palliat Med. 2022;25(5):797-801. doi: 10.1089/jpm.2021.0509. Epub 2022 Jan 28. | Dupl |
| 506. Heal DJ, Smith SL. Prospects for new drugs to treat binge-eating disorder: Insights from psychopathology and neuropharmacology. J Psychopharmacol. 2022;36(6):680-703. doi: 10.1177/02698811211032475. Epub 2021 Jul 28. | Review |
| 507. Vasiliu O. Investigational drugs for the treatment of depression (Part 1): Monoaminergic, orexinergic, GABA-ergic, and anti-inflammatory agents. Front Pharmacol. 2022;13:884143. doi: 10.3389/fphar.2022.884143. | Dupl |
| 508. Ishioh M, Nozu T, Miyagishi S, Igarashi S, Funayama T, Ohhira M, Okumura T. Activation of basal forebrain cholinergic neurons improves colonic hyperpermeability through the vagus nerve and adenosine A2B receptors in rats. Biochem Pharmacol. 2022;206:115331. doi: 10.1016/j.bcp.2022.115331. Epub 2022 Oct 29. | Animal |
| 509. Serretti A. Fine-tuning of psychopharmacological treatments. Int Clin Psychopharmacol. 2023 Jan 1;38(1):1-3. doi: 10.1097/YIC.0000000000000447. Epub 2022 Nov 18. | Opinion |
| 510. Rosenberg RP, Benca R, Doghramji P, Roth T. A 2023 Update on managing insomnia in primary care: Insights from an expert consensus group. Prim Care Companion CNS Disord. 2023;25(1):22nr03385. doi: 10.4088/PCC.22nr03385. | Review |
| 511. Javed B, Javed A, Kow CS, Hasan SS. Pharmacological and non-pharmacological treatment options for sleep disturbances in Alzheimer's disease. Expert Rev Neurother. 2023;23(6):501-514. doi: 10.1080/14737175.2023.2214316. Epub 2023 Jun 2. | Review |
| 512. Fagan HA, Baldwin DS. Pharmacological treatment of generalised anxiety disorder: Current practice and future directions. Expert Rev Neurother. 2023;23(6):535-548. doi: 10.1080/14737175.2023.2211767. Epub 2023 May 15. | Dupl |
| 513. Hori H. Successful treatment of switching from benzodiazepine to orexin receptor antagonists improves cognitive function in psychiatric disorders: four case reports. Int Clin Psychopharmacol. 2023;38(3):192-194. doi: 10.1097/YIC.0000000000000450. Epub 2022 Dec 16. | Case |
| 514. Pan B, Ge L, Lai H, Hou L, Tian C, Wang Q, Yang K, Lu Y, Zhu H, Li M, Wang D, Li X, Zhang Y, Gao Y, Liu M, Ding G, Tian J, Yang K. The comparative effectiveness and safety of insomnia drugs: A systematic review and network meta-analysis of 153 randomized trials. Drugs. 2023;83(7):587-619. doi: 10.1007/s40265-023-01859-8. Epub 2023 Mar 22. | Review |
| 515. Dabrowska J. From recent advances in underlying neurocircuitry of fear and anxiety to promising pharmacotherapies for PTSD: The saga of heart, sex and the developing brain. Neuropharmacology. 2023;232:109529. doi: 10.1016/j.neuropharm.2023.109529. Epub 2023 Mar 31. | Opinion |
| 516. Wu A. Updates and confounding factors in delayed sleep-wake phase disorder. Sleep Biol Rhythms. 2023;21(3):279-287. doi: 10.1007/s41105-023-00454-4. | Review |
| 517. Nakamura T, Yoshizawa T, Toya R, Terasawa M, Takahashi K, Kitazawa K, Suzuki K, Sasayama D, Washizuka S. Orexin receptor antagonists versus antipsychotics for the management of delirium in intensive care unit patients with cardiovascular disease: A retrospective observational study. Gen Hosp Psychiatry. 2023;84:96-101. doi: 10.1016/j.genhosppsych.2023.06.019. Epub 2023 Jul 1. | Dupl |
| 518. Bonifazi A, Del Bello F, Giorgioni G, Piergentili A, Saab E, Botticelli L, Cifani C, Micioni Di Bonaventura E, Micioni Di Bonaventura MV, Quaglia W. Targeting orexin receptors: Recent advances in the development of subtype selective or dual ligands for the treatment of neuropsychiatric disorders. Med Res Rev. 2023;43(5):1607-1667. doi: 10.1002/med.21959. Epub 2023 Apr 10. | Review |
| 519. Arai Y, Sasayama D, Kuraishi A, Sahara R, Murata S, Tanaka A, Amemiya K, Usuda N, Kuraishi K, Washizuka S. Sodium valproate use in Japanese patients with schizophrenia and Coronavirus disease is associated with an increased risk of pneumonia. J Clin Med. 2023;12(18):5953. doi: 10.3390/jcm12185953. | Unfocused |
| 520. Varadharajan A, Davis AD, Ghosh A, Jagtap T, Xavier A, Menon AJ, Roy D, Gandhi S, Gregor T. Guidelines for pharmacotherapy in Alzheimer's disease - A primer on FDA-approved drugs. J Neurosci Rural Pract. 2023;14(4):566-573. doi: 10.25259/JNRP_356_2023. Epub 2023 Oct 7. | Dupl |
| 521. Klugherz LJ, Kolla BP, Mansukhani MP. Pharmacological treatments for insomnia. Curr Sleep Medicine Rep. 2023;9(4):265-273. doi: 10.1007/s40675-023-00265-6 | Review |

| | |
|---|---|
| 522. Watanabe K, Misaka S, Kanno-Nozaki K, Chiyoda T, Suzuki Y, Sato A, Suto T, Kuroda J, Shimomura K, Miura I, Yabe H. Effect of lemborexant on pharmacokinetics of clozapine: A potential drug-drug interaction mediated by time-dependent inhibition of CYP3A4. Br J Clin Pharmacol. 2024;90(1):354-359. doi: 10.1111/bcp.15889. Epub 2023 Sep 4. | Dupl |
| 523. Aubin H-J. Repurposing drugs for treatment of alcohol use disorder. Int Rev Neurobiol. 2024;175:153-185. doi: 10.1016/bs.irn.2024.02.002. Epub 2024 Mar 12. | Dupl |
| 524. Tachibana M, Kanahara N, Oda Y, Hasegawa T, Kimura A, Iyo M. A retrospective clinical practice study comparing the usefulness of dual-orexin receptor antagonists and a melatonin receptor agonist in patients switching from long-term benzodiazepine receptor agonists. J Clin Sleep Med. 2024;20(4):603-613. doi: 10.5664/jcsm.10946. | Unfocused |
| 525. Kamata Y, Takashio O, Sato R, Kawai H, Ishii H, Aoyagi K, Tomita A, Toda S, Iwanami A. Relationship between insomnia and continued outpatient treatment in psychiatric patients. Neuropsychiatr Dis Treat. 2024;20:697-723. doi: 10.2147/NDT.S454757. | Unfocused |
| 526. Mori Y, Watanabe K, Suzuki Y, Ono H, Tojo M, Kawasaki Y, Kanno-Nozaki K, Nozaki M, Miura I. Clozapine-associated myocarditis in a patient with schizophrenia taking lemborexant: A case report. J Clin Psychopharmacol. 2024;44(2):193-195. doi: 10.1097/JCP.0000000000001817. Epub 2024 Feb 7. | Dupl |
| 527. Devlin JW. Pharmacologic treatment strategies for delirium in hospitalized adults: Past, present, and future. Semin Neurol. 2024;44(6):762-776. doi: 10.1055/s-0044-1791246. Epub 2024 Sep 23. | Review |
| 528. Liaskopoulos A, Kakouris V, Liaskopoulos N, Lappas AS, Christodoulou N, Samara M. Medical professionals and pharmacological intervention for the treatment of insomnia: A cross-sectional study. Sleep Sci. 2024;18(2):e155-e164. doi: 10.1055/s-0044-1791238. | Dupl |
| 529. Kikuchi Y, Kurosawa M, Sakata M, Takahashi Y, Yamamoto K, Tomita H, Yoshio T, Yasui-Furukori N. Effects of titration speed, gender, obesity and concomitant medications on the risk and onset time of clozapine-associated fever among Japanese patients with schizophrenia: retrospective review of charts from 21 hospitals. Br J Psychiatry. 2024;225(5):492-498. doi: 10.1192/bjp.2024.113. | Dupl |
| 530. Nozu T, Miyagishi S, Ishioh M, Takakusaki K, Okumura T. The neurotensin receptor 1 agonist PD149163 alleviates visceral hypersensitivity and colonic hyperpermeability in rat irritable bowel syndrome model. Neurogastroenterol Motil. 2024;36(12):e14925. doi: 10.1111/nmo.14925. Epub 2024 Sep 24. | Dupl |
| 531. Nakamura T, Furihata R, Hasegawa N, Kodaka F, Muraoka H, Ichihashi K, Ochi S, Numata S, Tsuboi T, Makinodan M, Iida H, Onitsuka T, Kashiwagi H, Takeshima M, Hashimoto N, Nagasawa T, Usami M, Yamagata H, Takaesu Y, Miura K, Matsumoto J, Ohi K, Yamada H, Hori H, Inada K, Watanabe K, Hashimoto R, Yasui-Furukori N. The effect of education regarding treatment guidelines for schizophrenia and major depressive disorders on psychiatrists' hypnotic medication prescribing behavior: a multicenter study. BMC Psychiatry. 2024;24(1):399. doi: 10.1186/s12888-024-05816-x. | Dupl |
| 532. Zhang M, Chen T, Lu X, Lan X, Chen Z, Lu S. G protein-coupled receptors (GPCRs): advances in structures, mechanisms, and drug discovery. Signal Transduct Target Ther. 2024;9(1):88. doi: 10.1038/s41392-024-01803-6. | Review |
| 533. Sogawa R, Hatano M, Nishimura F, Nishi J, Matsuoka A, Shinada K, Yamada H, Tateishi H, Mizoguchi Y, Monji A, Shimanoe C. Association between hypnotics, accidents, and injuries: A study based on the Adverse Drug Event Reporting Database in Japan. In Vivo. 2025;39(1):433-439. doi: 10.21873/invivo.13846. | Unfocused |
| 534. Ngomana C, Komape KD, Bronkhorst E. A review on holistic and pharmacological management of insomnia. SA Pharm J. 2025;92(1):11-16. doi: 10.36303/SAPJ.2283. | Review |
| 535. Chopra A, Rustad JK, Hall DL, Mak MSB, Stern TA. Management of Insomnia in the General Hospital. Prim Care Companion CNS Disord. 2025;27(1):24f03793. doi: 10.4088/PCC.24f03793. | Case |
| 536. Nozu T, Miyagishi S, Ishioh M, Takakusaki K, Okumura T. Irisin prevents visceral hypersensitivity and colonic hyperpermeability in a rat model of irritable bowel syndrome. Peptides. 2025;188:171394. doi: 10.1016/j.peptides.2025.171394. Epub 2025 Mar 26. | Dupl |
| 537. Mori K, Ohashi K, Kimura M, Yoshida M, Tomida K, Usami E (森 光輝，大橋健吾，木村美智男，吉田光代，富田顕旨，宇佐美英績). オレキシン受容体拮抗薬推進活動が睡眠薬・抗精神病薬の処方動向に与える影響：分割時系列解析研究 [Impact of promotional activities on orexin receptor antagonists prescription rates and usage of sleep and antipsychotic medications: An interrupted time-series analysis study]. Yakugaku Zasshi. 2025;145(7):629-637. Japanese. doi: 10.1248/yakushi.24-00170. | Dupl |
| 538. Tanaka A, Arai Y, Yasaki T, Saito K, Nakashizuka E, Yoshida S, Nakajima Y, Koido M, Suzuki K, Nakamura T, Sasayama D, Washizuka S. Longitudinal associations of serum orexin-A with physical activity and sleep in schizophrenia: A preliminary study. Neuropsychopharmacol Rep. 2025;45(4):e70079. doi: 10.1002/npr2.70079. | Dupl |
| 539. IsHak WW, Hirsch D, Renteria S, Totlani J, Murphy N, Chang T, Abdelsalam R, Salem M, Meyer A, Keerthana S, Liu A, Contreras L, Tadros E, Hedrick R, Danovitch I, Pechnick RN. Depressive disorders: systematic review of approved psychiatric medications (2009-April 2025) and pipeline phase 3 medications. BMC Psychiatry. 2025;25(1):939. doi: 10.1186/s12888-025-07141-3. | Review |
| 540. Fuglsang NFB, Madsen NM, Jacobsen SL, Frøkjær JE, Ladegaard N, Sørensen MA, Correll CU, Otte C, Højlund M, Köhler-Forsberg O. Efficacy and Acceptability of Licensed and Off-Label Pharmacological Interventions for Insomnia in Patients With Severe Mental Illness: A Systematic Review and Meta-Analysis of Randomised Trials. Acta Psychiatr Scand. 2025;152(6):405-421. doi: 10.1111/acps.70032. Epub 2025 Sep 2. | Review |
| 541. Garfield SL. An angry and critical appraisal of professional psychology. Clin Psychol Sci Pract. 1996;3(3):268-272. | Opinion |
| 542. Nakamura T, Uramura K, Nambu T, Yada T, Goto K, Yanagisawa M, Sakurai T. Orexin-induced hyperlocomotion and stereotypy are mediated by the dopaminergic system. Brain Res. 2000;873(1):181-7. doi: 10.1016/s0006-8993(00)02555-5. | Dupl |
| 543. Nishino S, Ripley B, Mignot E, Benson KL, Zarcone VP. CSF hypocretin-1 levels in schizophrenics and controls: relationship to sleep architecture. Psychiatry Res. 2002;110(1):1-7. doi: 10.1016/s0165-1781(02)00032-x. | Dupl |
| 544. Fadel J, Bubser M, Deutch AY. Differential activation of orexin neurons by antipsychotic drugs associated with weight gain. J Neurosci. 2002;22(15):6742-6. doi: 10.1523/JNEUROSCI.22-15-06742.2002. | Dupl |
| 545. Katz JD, Ropper AH. Familial Kleine-Levin syndrome: two siblings with unusually long hypersomnic spells. Arch Neurol. 2002;59(12):1959-61. doi: 10.1001/archneur.59.12.1959. | Dupl |
| 546. Đukić Dejanović SM, Janjić V, Milovanović D. Etiopatogeneza nesanice [Aetiopathogenesis of insomnia]. Psihijatrija Danas 2003;35(1):5-21. | Review |
| 547. Monda M, Viggiano A, De Luca V. Haloperidol reduces the sympathetic and thermogenic activation induced by orexin A. Neurosci Res. 2003;45(1):17-23. doi: 10.1016/s0168-0102(02)00191-8. | Dupl |
| 548. Midei A, Licinio J. General summary. Mol Psychiatry. 2003;8(10):833-834. doi: 10.1038/sj.mp.4001416. | Opinion |
| 549. Alkemade A, Unmehopa UA, Brouwer JP, Hoogendijk WJ, Wiersinga WM, Swaab DF, Fliers E. Decreased thyrotropin-releasing hormone gene expression in the hypothalamic paraventricular nucleus of patients with major depression. Mol Psychiatry. 2003;8(10):838-9. doi: 10.1038/sj.mp.4001364. | Unrelated |
| 550. Douglass AB. Narcolepsy: differential diagnosis or etiology in some cases of bipolar disorder and schizophrenia? CNS Spectr. 2003;8(2):120-6. doi: 10.1017/s1092852900018344. | Dupl |
| 551. Young WB, Piovesan EJ, Biglan KM. Restless legs syndrome and drug-induced akathisia in headache patients. CNS Spectr. 2003;8(6):450-6. doi: 10.1017/s1092852900018769. | Dupl |

| | |
|---|---|
| 552. Sheitman BB, Knable MB, Jarskog LF, Chakos M, Boyce LH, Early J, Lieberman JA. Secretin for refractory schizophrenia. Schizophr Res. 2004;66(2-3):177-81. doi: 10.1016/S0920-9964(03)00068-9. | Dupl |
| 553. Figlewicz DP, Bennett J, Evans SB, Kaiyala K, Sipols AJ, Benoit SC. Intraventricular insulin and leptin reverse place preference conditioned with high-fat diet in rats. Behav Neurosci. 2004;118(3):479-87. doi: 10.1037/0735-7044.118.3.479. | Animal |
| 554. Annual Meeting of the Biological Psychologists and Neuropsychologists of the German Society of Psychology (DGPs) as well as the German Society of Psychophysiology and its Applications (DGPA) - 31st APM: Psychology and Brain, Bochum, Germany, May 25-27, 2005. J Psychophysiol. 2005;19(2):106-156. | Abstr |
| 555. Pecoraro N, Dallman MF. c-Fos after incentive shifts: expectancy, incredulity, and recovery. Behav Neurosci. 2005;119(2):366-87. doi: 10.1037/0735-7044.119.2.366. | Animal |
| 556. Walterfang M, Upjohn E, Velakoulis D. Is schizophrenia associated with narcolepsy? Cogn Behav Neurol. 2005;18(2):113-8. doi: 10.1097/01.wnn.0000160822.53577.2c. | Dupl |
| 557. Meerabux J, Iwayama Y, Sakurai T, Ohba H, Toyota T, Yamada K, Nagata R, Irukayama-Tomobe Y, Shimizu H, Yoshitsugu K, Ohta K, Yoshikawa T. Association of an orexin 1 receptor 408Val variant with polydipsia-hyponatremia in schizophrenic subjects. Biol Psychiatry. 2005;58(5):401-7. doi: 10.1016/j.biopsych.2005.04.015. | Dupl |
| 558. Baldo BA, Alsene KM, Negron A, Kelley AE. Hyperphagia induced by GABAA receptor-mediated inhibition of the nucleus accumbens shell: dependence on intact neural output from the central amygdaloid region. Behav Neurosci. 2005;119(5):1195-206. doi: 10.1037/0735-7044.119.5.1195. | Animal |
| 559. Barbano MF, Cador M. Various aspects of feeding behavior can be partially dissociated in the rat by the incentive properties of food and the physiological state. Behav Neurosci. 2005;119(5):1244-53. doi: 10.1037/0735-7044.119.5.1244. | Animal |
| 560. Cochen V, Arnulf I, Demeret S, Neulat ML, Gourlet V, Drouot X, Moutereau S, Derenne JP, Similowski T, Willer JC, Pierrot-Deseiligny C, Bolgert F. Vivid dreams, hallucinations, psychosis and REM sleep in Guillain-Barré syndrome. Brain. 2005;128(Pt 11):2535-45. doi: 10.1093/brain/awh585. Epub 2005 Jul 6. | Dupl |
| 561. Narita M, Nagumo Y, Hashimoto S, Narita M, Khotib J, Miyatake M, Sakurai T, Yanagisawa M, Nakamachi T, Shioda S, Suzuki T. Direct involvement of orexinergic systems in the activation of the mesolimbic dopamine pathway and related behaviors induced by morphine. J Neurosci. 2006;26(2):398-405. doi: 10.1523/JNEUROSCI.2761-05.2006. | Dupl |
| 562. Alberto CO, Trask RB, Quinlan ME, Hirasawa M. Bidirectional dopaminergic modulation of excitatory synaptic transmission in orexin neurons. J Neurosci. 2006;26(39):10043-50. doi: 10.1523/JNEUROSCI.1819-06.2006. Retraction in: J Neurosci. 2012;32(26):9116. doi: 10.1523/JNEUROSCI.1889-12.2012. | Dupl |
| 563. Monda M, Viggiano A, Viggiano A, Viggiano E, Messina G, Tafuri D, De Luca V. Quetiapine lowers sympathetic and hyperthermic reactions due to cerebral injection of orexin A. Neuropeptides. 2006;40(5):357-63. doi: 10.1016/j.npep.2006.07.003. Epub 2006 Sep 28. | Dupl |
| 564. De Clercq B, De Fruyt F, Van Leeuwen K, Mervielde I. The structure of maladaptive personality traits in childhood: a step toward an integrative developmental perspective for DSM-V. J Abnorm Psychol. 2006;115(4):639-57. doi: 10.1037/0021-843X.115.4.639. | Unrelated |
| 565. Rasmussen K, Hsu MA, Yang Y. The orexin-1 receptor antagonist SB-334867 blocks the effects of antipsychotics on the activity of A9 and A10 dopamine neurons: implications for antipsychotic therapy. Neuropsychopharmacology. 2007;32(4):786-92. doi: 10.1038/sj.npp.1301239. Epub 2006 Oct 25. | Dupl |
| 566. Brundin L, Petersén A, Björkqvist M, Träskman-Bendz L. Orexin and psychiatric symptoms in suicide attempters. J Affect Disord. 2007;100(1-3):259-63. doi: 10.1016/j.jad.2006.10.019. Epub 2006 Dec 4. | Dupl |
| 567. Brundin L, Björkqvist M, Petersén A, Träskman-Bendz L. Reduced orexin levels in the cerebrospinal fluid of suicidal patients with major depressive disorder. Eur Neuropsychopharmacol. 2007;17(9):573-9. doi: 10.1016/j.euroneuro.2007.01.005. Epub 2007 Mar 7. | Dupl |
| 568. Lambe EK, Liu RJ, Aghajanian GK. Schizophrenia, hypocretin (orexin), and the thalamocortical activating system. Schizophr Bull. 2007;33(6):1284-90. doi: 10.1093/schbul/sbm088. Epub 2007 Jul 26. | Dupl |
| 569. Rasmussen K, Hsu MA, Noone S, Johnson BG, Thompson LK, Hemrick-Luecke SK. The orexin-1 antagonist SB-334867 blocks antipsychotic treatment emergent catalepsy: implications for the treatment of extrapyramidal symptoms. Schizophr Bull. 2007;33(6):1291-7. doi: 10.1093/schbul/sbm087. Epub 2007 Jul 28. | Dupl |
| 570. Morein-Zamir S, Turner DC, Sahakian BJ. A review of the effects of modafinil on cognition in schizophrenia. Schizophr Bull. 2007;33(6):1298-306. doi: 10.1093/schbul/sbm090. Epub 2007 Jul 18. | Dupl |
| 571. Janas-Kozik M, Stachowicz M, Mazurek U, Zajdel A, Wilczok A, Krupka-Matuszczyk I, Rybakowski JK. Preliminary study of the expression of genes connected with the orexigenic and anorexigenic system using microarray technique in anorexia nervosa. Neuropsychobiology. 2008;57(3):116-20. doi: 10.1159/000138913. Epub 2008 Jun 13. | Dupl |
| 572. Minzenberg MJ, Carter CS. Modafinil: a review of neurochemical actions and effects on cognition. Neuropsychopharmacology. 2008;33(7):1477-502. doi: 10.1038/sj.npp.1301534. Epub 2007 Aug 22. | Dupl |
| 573. Qu WM, Huang ZL, Xu XH, Matsumoto N, Urade Y. Dopaminergic D1 and D2 receptors are essential for the arousal effect of modafinil. J Neurosci. 2008;28(34):8462-9. doi: 10.1523/JNEUROSCI.1819-08.2008. | Dupl |
| 574. Wallingford NM, Sinnayah P, Bymaster FP, Gadde KM, Krishnan RK, McKinney AA, Landbloom RP, Tollefson GD, Cowley MA. Zonisamide prevents olanzapine-associated hyperphagia, weight gain, and elevated blood glucose in rats. Neuropsychopharmacology. 2008;33(12):2922-33. doi: 10.1038/npp.2008.9. Epub 2008 Mar 5. | Dupl |
| 575. Stefanidis A, Verty AN, Allen AM, Owens NC, Cowley MA, Oldfield BJ. The role of thermogenesis in antipsychotic drug-induced weight gain. Obesity (Silver Spring). 2009;17(1):16-24. doi: 10.1038/oby.2008.468. Epub 2008 Oct 30. | Dupl |
| 576. Cope MB, Li X, Jumbo-Lucioni P, DiCostanzo CA, Jamison WG, Kesterson RA, Allison DB, Nagy TR. Risperidone alters food intake, core body temperature, and locomotor activity in mice. Physiol Behav. 2009;96(3):457-63. doi: 10.1016/j.physbeh.2008.11.011. Epub 2008 Nov 27. | Dupl |
| 577. Brockington IF. Monthly psychosis starting before the menarche. Arch Womens Ment Health. 2009;12(2):121-2. doi: 10.1007/s00737-009-0053-x. Epub 2009 Mar 27. | Opinion |
| 578. Davoodi N, Kalinichev M, Korneev SA, Clifton PG. Hyperphagia and increased meal size are responsible for weight gain in rats treated sub-chronically with olanzapine. Psychopharmacology (Berl). 2009;203(4):693-702. doi: 10.1007/s00213-008-1415-1. Epub 2008 Dec 4. | Dupl |
| 579. Gritton HJ, Sutton BC, Martinez V, Sarter M, Lee TM. Interactions between cognition and circadian rhythms: attentional demands modify circadian entrainment. Behav Neurosci. 2009;123(5):937-48. doi: 10.1037/a0017128. | Animal |

| | |
|---|---|
| 580. Panossian LA, Avidan AY. Narcolepsy and other comorbid medical illnesses. In: Goswami M, Pandi-Perumal S, Thorpy M (eds) Narcolepsy. New York, NY: Springer, 2010; pp. 105-113. doi: 10.1007/978-1-4419-0854-4_10. | Review |
| 581. Schenberg LC. Towards a translational model of panic attack. Psychol Neurosci. 2010;3(1):9-37. doi: 10.3922/j.psns.2010.1.003. | Review |
| 582. Winrow CJ, Tanis KQ, Reiss DR, Rigby AM, Uslaner JM, Uebele VN, Doran SM, Fox SV, Garson SL, Gotter AL, Levine DM, Roecker AJ, Coleman PJ, Koblan KS, Renger JJ. Orexin receptor antagonism prevents transcriptional and behavioral plasticity resulting from stimulant exposure. Neuropharmacology. 2010;58(1):185-94. doi: 10.1016/j.neuropharm.2009.07.008. Epub 2009 Jul 14. | Animal |
| 583. Borgland SL, Labouèbe G. Orexin/hypocretin in psychiatric disorders: present state of knowledge and future potential. Neuropsychopharmacology. 2010;35(1):353-4. doi: 10.1038/npp.2009.119. | Dupl |
| 584. Basoglu C, Oner O, Gunes C, Semiz UB, Ates AM, Algul A, Ebrinc S, Cetin M, Ozcan O, Ipcioglu O. Plasma orexin A, ghrelin, cholecystokinin, visfatin, leptin and agouti-related protein levels during 6-week olanzapine treatment in first-episode male patients with psychosis. Int Clin Psychopharmacol. 2010;25(3):165-71. doi: 10.1097/YIC.0b013e3283377850. | Dupl |
| 585. Milella MS, Passarelli F, De Carolis L, Schepisi C, Nativio P, Scaccianoce S, Nencini P. Opposite roles of dopamine and orexin in quinpirole-induced excessive drinking: a rat model of psychotic polydipsia. Psychopharmacology (Berl). 2010;211(3):355-66. doi: 10.1007/s00213-010-1909-5. Epub 2010 Jun 16. | Dupl |
| 586. Burgess CR, Tse G, Gillis L, Peever JH. Dopaminergic regulation of sleep and cataplexy in a murine model of narcolepsy. Sleep. 2010;33(10):1295-304. doi: 10.1093/sleep/33.10.1295. | Animal |
| 587. Sanislow CA, Pine DS, Quinn KJ, Kozak MJ, Garvey MA, Heinssen RK, Wang PS, Cuthbert BN. Developing constructs for psychopathology research: research domain criteria. J Abnorm Psychol. 2010;119(4):631-9. doi: 10.1037/a0020909. | Opinion |
| 588. Hester R, Lee N, Pennay A, Nielsen S, Ferris J. The effects of modafinil treatment on neuropsychological and attentional bias performance during 7-day inpatient withdrawal from methamphetamine dependence. Exp Clin Psychopharmacol. 2010;18(6):489-97. doi: 10.1037/a0021791. | Unfocused |
| 589. Panariello F, Javaid N, Teo C, Monda M, Viggiano A, De Luca V. The role of orexin system in antipsychotics induced weight gain. Curr Psychiatry Rev. 2011;7(1):12-18. doi: 10.2174/157340011795945793. | Review |
| 590. Fernø J, Varela L, Skrede S, Vázquez MJ, Nogueiras R, Diéguez C, Vidal-Puig A, Steen VM, López M. Olanzapine-induced hyperphagia and weight gain associate with orexigenic hypothalamic neuropeptide signaling without concomitant AMPK phosphorylation. PLoS One. 2011;6(6):e20571. doi: 10.1371/journal.pone.0020571. Epub 2011 Jun 13. | Dupl |
| 591. Wirtshafter D, Davis JD, Stratford TR. Inactivation of the median raphe nucleus increases intake of sucrose solutions: a microstructural analysis. Behav Neurosci. 2011;125(4):529-40. doi: 10.1037/a0024372. | Animal |
| 592. de Lecea L, Carter ME, Adamantidis A. Shining light on wakefulness and arousal. Biol Psychiatry. 2012;71(12):1046-52. doi: 10.1016/j.biopsych.2012.01.032. Epub 2012 Mar 20. | Review |
| 593. Bradshaw CM, Killeen PR. A theory of behaviour on progressive ratio schedules, with applications in behavioural pharmacology. Psychopharmacology (Berl). 2012;222(4):549-64. doi: 10.1007/s00213-012-2771-4. Epub 2012 Jul 3. | Dupl |
| 594. Lehner T. The genes in the major histocompatibility complex as risk factors for schizophrenia: de omnibus dubitandum. Biol Psychiatry. 2012;72(8):615-6. doi: 10.1016/j.biopsych.2012.08.002. | Opinion |
| 595. Girault EM, Foppen E, Ackermans MT, Fliers E, Kalsbeek A. Central administration of an orexin receptor 1 antagonist prevents the stimulatory effect of Olanzapine on endogenous glucose production. Brain Res. 2013;1527:238-45. doi: 10.1016/j.brainres.2013.06.034. Epub 2013 Jul 4. | Dupl |
| 596. Gozzi A, Lepore S, Vicentini E, Merlo-Pich E, Bifone A. Differential effect of orexin-1 and CRF-1 antagonism on stress circuits: a fMRI study in the rat with the pharmacological stressor Yohimbine. Neuropsychopharmacology. 2013;38(11):2120-30. doi: 10.1038/npp.2013.109. Epub 2013 May 8. | Dupl |
| 597. Abstracts of the annual CSBBCS meeting: 2013 résumés du congrès annuel 2013 de la SCCCSC. Canadian Journal of Experimental Psychology / Revue canadienne de psychologie expérimentale, 2013, 67, 4, 271-312 (Prenatal Activation of Orexinergic Neurons, Maria Pompeiano, Ahn Lee, Kyle E. Godden). | Abstr |
| 598. Huang YS, Guilleminault C, Chen CH, Lai PC, Hwang FM. Narcolepsy-cataplexy and schizophrenia in adolescents. Sleep Med. 2014;15(1):15-22. doi: 10.1016/j.sleep.2013.09.018. Epub 2013 Oct 26. | Dupl |
| 599. Bonfils KA, Adams EL, Firmin RL, White LM, Salyers MP. Parenthood and severe mental illness: relationships with recovery. Psychiatr Rehabil J. 2014;37(3):186-93. doi: 10.1037/prj0000072. Epub 2014 May 12. | Unrelated |
| 600. Palotai M, Telegdy G, Ekwerike A, Jászberényi M. The action of orexin B on passive avoidance learning. Involvement of neurotransmitters. Behav Brain Res. 2014;272:1-7. doi: 10.1016/j.bbr.2014.06.016. Epub 2014 Jun 13. | Dupl |
| 601. Inutsuka A, Inui A, Tabuchi S, Tsunematsu T, Lazarus M, Yamanaka A. Concurrent and robust regulation of feeding behaviors and metabolism by orexin neurons. Neuropharmacology. 2014;85:451-60. doi: 10.1016/j.neuropharm.2014.06.015. Epub 2014 Jun 18. | Dupl |
| 602. Messina G, Viggiano A, Chieffi S, Viggiano E, Tafuri D, De Luca V, Messina A, Monda M. Neuroleptic drugs affect sympathetic and thermogenic reactions to Orexin A. Afr J Psychiatry. 2014;17(6):1000175. doi:10.4172/Psychiatry.1000172 | Review |
| 603. Risco S, Mediavilla C. Orexin-1 receptor antagonist in central nucleus of the amygdala attenuates the acquisition of flavor-taste preference in rats. Pharmacol Biochem Behav. 2014;126:7-12. doi: 10.1016/j.pbb.2014.09.002. Epub 2014 Sep 16. | Animal |
| 604. Kolaj M, Zhang L, Renaud LP. Novel coupling between TRPC-like and KNa channels modulates low threshold spike-induced afterpotentials in rat thalamic midline neurons. Neuropharmacology. 2014;86:88-96. doi: 10.1016/j.neuropharm.2014.06.023. Epub 2014 Jul 9. | Dupl |
| 605. Chien YL, Liu CM, Shan JC, Lee HJ, Hsieh MH, Hwu HG, Chiou LC. Elevated plasma orexin A levels in a subgroup of patients with schizophrenia associated with fewer negative and disorganized symptoms. Psychoneuroendocrinology. 2015;53:1-9. doi: 10.1016/j.psyneuen.2014.12.012. Epub 2014 Dec 20. | Dupl |
| 606. Bailey MR, Jensen G, Taylor K, Mezias C, Williamson C, Silver R, Simpson EH, Balsam PD. A novel strategy for dissecting goal-directed action and arousal components of motivated behavior with a progressive hold-down task. Behav Neurosci. 2015;129(3):269-80. doi: 10.1037/bne0000060. | Animal |
| 607. Rojczyk E, Pałasz A, Wiaderkiewicz R. Effect of short and long-term treatment with antipsychotics on orexigenic/anorexigenic neuropeptides expression in the rat hypothalamus. Neuropeptides. 2015;51:31-42. doi: 10.1016/j.npep.2015.04.001. Epub 2015 Apr 3. | Dupl |
| 608. Nishizawa D, Kasai S, Hasegawa J, Sato N, Yamada H, Tanioka F, Nagashima M, Katoh R, Satoh Y, Tagami M, Ujike H, Ozaki N, Inada T, Iwata N, Sora I, Iyo M, Yamada M, Kondo N, Won MJ, Naruse N, Uehara-Aoyama K, Itokawa M, Ohi K, Hashimoto R, Tanisawa K, Arai T, Mori S, Sawabe M, Naka-Mieno M, Yamada Y, Yamada M, Sato N, Muramatsu M, Tanaka M, Irukayama-Tomobe Y, Saito YC, Sakurai T, | Dupl |

| | |
|---|---|
| Hayashida M, Sugimura H, Ikeda K. Associations between the orexin (hypocretin) receptor 2 gene polymorphism Val308Ile and nicotine dependence in genome-wide and subsequent association studies. Mol Brain. 2015;8:50. doi: 10.1186/s13041-015-0142-x. | |
| 609. Moradi M, Yazdanian M, Haghparast A. Role of dopamine D2-like receptors within the ventral tegmental area and nucleus accumbens in antinociception induced by lateral hypothalamus stimulation. Behav Brain Res. 2015;292:508-14. doi: 10.1016/j.bbr.2015.07.007. Epub 2015 Jul 9. | Dupl |
| 610. Parker KE, McCabe MP, Johns HW, Lund DK, Odu F, Sharma R, Thakkar MM, Cornelison DD, Will MJ. Neural activation patterns underlying basolateral amygdala influence on intra-accumbens opioid-driven consummatory versus appetitive high-fat feeding behaviors in the rat. Behav Neurosci. 2015;129(6):812-21. doi: 10.1037/bne0000095. Epub 2015 Oct 26. | Animal |
| 611. Krystal AD. New developments in insomnia medications of relevance to mental health disorders. Psychiatr Clin North Am. 2015;38(4):843-60. doi: 10.1016/j.psc.2015.08.001. Epub 2015 Sep 11. | Dupl |
| 612. Vickers SP, Hackett D, Murray F, Hutson PH, Heal DJ. Effects of lisdexamfetamine in a rat model of binge-eating. J Psychopharmacol. 2015;29(12):1290-307. doi: 10.1177/0269881115615107. Epub 2015 Nov 20. | Dupl |
| 613. Oepen G, Oepen I. Possible motor benefits of modafinil in Parkinsonian antecollis: A case report. Clin Neuropsychiatry. 2016;13(1-2):7-9. | Case |
| 614. Sun HQ, Li SX, Chen FB, Zhang Y, Li P, Jin M, Sun Y, Wang F, Mi WF, Shi L, Yue JL, Yang FD, Lu L. Diurnal neurobiological alterations after exposure to clozapine in first-episode schizophrenia patients. Psychoneuroendocrinology. 2016;64:108-16. doi: 10.1016/j.psyneuen.2015.11.013. Epub 2015 Nov 26. | Dupl |
| 615. Tiwari AK, Brandl EJ, Zai CC, Goncalves VF, Chowdhury NI, Freeman N, Lieberman JA, Meltzer HY, Kennedy JL, Müller DJ. Association of orexin receptor polymorphisms with antipsychotic-induced weight gain. World J Biol Psychiatry. 2016;17(3):221-9. doi: 10.3109/15622975.2015.1076173. Epub 2015 Oct 8. | Dupl |
| 616. Altena E, Micoulaud-Franchi JA, Geoffroy PA, Sanz-Arigita E, Bioulac S, Philip P. The bidirectional relation between emotional reactivity and sleep: From disruption to recovery. Behav Neurosci. 2016;130(3):336-50. doi: 10.1037/bne0000128. Epub 2016 Feb 11. | Review |
| 617. Wagner L, Kaestner F, Wolf R, Stiller H, Heiser U, Manhart S, Hoffmann T, Rahfeld JU, Demuth HU, Rothermundt M, von Hörsten S. Identifying neuropeptide Y (NPY) as the main stress-related substrate of dipeptidyl peptidase 4 (DPP4) in blood circulation. Neuropeptides. 2016;57:21-34. doi: 10.1016/j.npep.2016.02.007. Epub 2016 Feb 27. | Dupl |
| 618. Kelly JF. Proceedings of the American Psychological Association, Incorporated, for the legislative year 2015: Minutes of the Annual Meeting of the Council of Representatives February 20-22, 2015, Washington, DC, and August 5 and August 7, 2015, Washington, DC, and minutes of the February, June, August, and December 2015 meetings of the Board of Directors. Am Psychol. 2016;71(5):369-414. doi: 10.1037/amp0000022. | Abstr |
| 619. Luck C, Vitaterna MH, Wevrick R. Dopamine pathway imbalance in mice lacking Magel2, a Prader-Willi syndrome candidate gene. Behav Neurosci. 2016;130(4):448-59. doi: 10.1037/bne0000150. Epub 2016 Jun 2. | Animal |
| 620. Xue Y, Yang YT, Liu HY, Chen WF, Chen AQ, Sheng Q, Chen XY, Wang Y, Chen H, Liu HX, Pang YY, Chen L. Orexin-A increases the activity of globus pallidus neurons in both normal and parkinsonian rats. Eur J Neurosci. 2016;44(5):2247-57. doi: 10.1111/ejn.13323. Epub 2016 Jul 13. | Dupl |
| 621. McHenry JA, Robison CL, Bell GA, Vialou VV, Bolaños-Guzmán CA, Nestler EJ, Hull EM. The role of ΔfosB in the medial preoptic area: Differential effects of mating and cocaine history. Behav Neurosci. 2016;130(5):469-78. doi: 10.1037/bne0000160. | Animal |
| 622. Sansa G, Gavaldà A, Gaig C, Monreal J, Ercilla G, Casamitjana R, Ribera G, Iranzo A, Santamaria J. Exploring the presence of narcolepsy in patients with schizophrenia. BMC Psychiatry. 2016;16:177. doi: 10.1186/s12888-016-0859-9. | Dupl |
| 623. Schweitzer PK, Feren SD. Pharmacological treatment of insomnia. In Attarian HP (ed.), Clinical Handbook of Insomnia, 3rd ed. Totowa, NJ: Humana Press/Springer Nature, 2017; pp. 97-132. https://doi.org/10.1007/978-3-319-41400-3_7. | Review |
| 624. Zink, Anastasia N. Neuromodulation of orexin neurons in the lateral hypothalamus regulates spontaneous physical activity, energy expenditure, and diet-induced obesity. Dissertation Abstracts International: Section B: The Sciences and Engineering, 2017;78:3-B(E). PhD Thesis, University of Minnesota, August 2015. | Animal |
| 625. Mahoney CE, Agostinelli LJ, Brooks JN, Lowell BB, Scammell TE. GABAergic neurons of the central amygdala promote cataplexy. J Neurosci. 2017;37(15):3995-4006. doi: 10.1523/JNEUROSCI.4065-15.2017. Epub 2017 Feb 24. | Dupl |
| 626. Bunney PE, Zink AN, Holm AA, Billington CJ, Kotz CM. Orexin activation counteracts decreases in nonexercise activity thermogenesis (NEAT) caused by high-fat diet. Physiol Behav. 2017;176:139-148. doi: 10.1016/j.physbeh.2017.03.040. Epub 2017 Mar 28. | Dupl |
| 627. Colom-Lapetina J, Begley SL, Johnson ME, Bean KJ, Kuwamoto WN, Shansky RM. Strain-dependent sex differences in a long-term forced swim paradigm. Behav Neurosci. 2017;131(5):428-36. doi: 10.1037/bne0000215. Epub 2017 Aug 14. | Animal |
| 628. Dixon ML, Thiruchselvam R, Todd R, Christoff K. Emotion and the prefrontal cortex: An integrative review. Psychol Bull. 2017;143(10):1033-1081. doi: 10.1037/bul0000096. Epub 2017 Jun 15. | Review |
| 629. Campbell EJ, Mitchell CS, Adams CD, Yeoh JW, Hodgson DM, Graham BA, Dayas CV. Chemogenetic activation of the lateral hypothalamus reverses early life stress-induced deficits in motivational drive. Eur J Neurosci. 2017;46(7):2285-2296. doi: 10.1111/ejn.13674. Epub 2017 Sep 22. | Dupl |
| 630. Keks NA, Hope J, Keogh S. Suvorexant: scientifically interesting, utility uncertain. Australas Psychiatry. 2017;25(6):622-624. doi: 10.1177/1039856217734677. Epub 2017 Oct 10. | Dupl |
| 631. Bolton JL, Ruiz CM, Rismanchi N, Sanchez GA, Castillo E, Huang J, Cross C, Baram TZ, Mahler SV. Early-life adversity facilitates acquisition of cocaine self-administration and induces persistent anhedonia. Neurobiol Stress. 2018;8:57-67. doi: 10.1016/j.ynstr.2018.01.002. | Dupl |
| 632. Dujardin S, Pijpers A, Pevernagie D. Prescription drugs used in insomnia. Sleep Med Clin. 2018;13(2):169-182. doi: 10.1016/j.jsmc.2018.03.001. | Dupl |
| 633. Öz P, Gökalp HK, Gӧver T, Uzbay T. Dose-dependent and opposite effects of orexin A on prepulse inhibition response in sleep-deprived and non-sleep- deprived rats. Behav Brain Res. 2018;346:73-79. doi: 10.1016/j.bbr.2017.12.002. Epub 2017 Dec 10. | Dupl |
| 634. Risco S, Mediavilla C. Orexin A in the ventral tegmental area enhances saccharin-induced conditioned flavor preference: The role of D1 receptors in central nucleus of amygdala. Behav Brain Res. 2018;348:192-200. doi: 10.1016/j.bbr.2018.04.010. Epub 2018 Apr 21. | Dupl |
| 635. Juvodden HT, Alnæs D, Lund MJ, Agartz I, Andreassen OA, Dietrichs E, Thorsby PM, Westlye LT, Knudsen S. Widespread white matter changes in post-H1N1 patients with narcolepsy type 1 and first-degree relatives. Sleep. 2018;41(10):zsy145. doi: 10.1093/sleep/zsy145. | Dupl |
| 636. Prihodova I, Dudova I, Mohaplova M, Hrdlicka M, Nevsimalova S. Childhood narcolepsy and autism spectrum disorders: four case reports. Sleep Med. 2018;51:167-170. doi: 10.1016/j.sleep.2018.07.017. Epub 2018 Aug 9. | Dupl |

| | |
|---|---|
| 637. Chen P-Y, Chen C-H, Chang C-K, Kao C-F, Lu M-L, Lin S-K, Huang M-C, Hwang L-L, Mondelli V. Orexin-A levels in relation to the risk of metabolic syndrome in patients with schizophrenia taking antipsychotics. Int J Neuropsychopharmacol. 2019;22(1):28-36. doi: 10.1093/ijnp/pyy075. | Dupl |
| 638. Baykal S, Albayrak Y, Durankuş F, Güzel S, Abbak Ö, Potas N, Beyazyüz M, Karabekiroğlu K, Donma MM. Decreased serum orexin A levels in drug-naive children with attention deficit and hyperactivity disorder. Neurol Sci. 2019;40(3):593-602. doi: 10.1007/s10072-018-3692-8. Epub 2019 Jan 7. | No psychosis |
| 639. Prieto DI, Zehgeer AA, Connor DF. Use of Suvorexant for Sleep Regulation in an Adolescent with Early-Onset Bipolar Disorder. J Child Adolesc Psychopharmacol. 2019;29(5):395. doi: 10.1089/cap.2019.0029. Epub 2019 Apr 26. | Case |
| 640. Misiak B, Bartoli F, Stramecki F, Samochowiec J, Lis M, Kasznia J, Jarosz K, Stańczykiewicz B. Appetite regulating hormones in first-episode psychosis: A systematic review and meta-analysis. Neurosci Biobehav Rev. 2019;102:362-370. doi: 10.1016/j.neubiorev.2019.05.018. Epub 2019 May 20. | Dupl |
| 641. Tsuchimine S, Hattori K, Ota M, Hidese S, Teraishi T, Sasayama D, Hori H, Noda T, Yoshida S, Yoshida F, Kunugi H. Reduced plasma orexin-A levels in patients with bipolar disorder. Neuropsychiatr Dis Treat. 2019;15:2221-2230. doi: 10.2147/NDT.S209023. | Dupl |
| 642. Krystal AD, Prather AA, Ashbrook LH. The assessment and management of insomnia: an update. World Psychiatry. 2019;18(3):337-352. doi: 10.1002/wps.20674. | Dupl |
| 643. Ni P, Tian Y, Gu X, Yang L, Wei J, Wang Y, Zhao L, Zhang Y, Zhang C, Li L, Tang X, Ma X, Hu X, Li T. Plasma neuropeptides as circulating biomarkers of multifactorial schizophrenia. Compr Psychiatry. 2019;94:152114. doi: 10.1016/j.comppsych.2019.152114. Epub 2019 Aug 5. | Dupl |
| 644. Hanazawa T, Kamijo Y. Effect of suvorexant on nocturnal delirium in elderly patients with Alzheimer's disease: A case-series study. Clin Psychopharmacol Neurosci. 2019;17(4):547-550. doi: 10.9758/cpn.2019.17.4.547. | Dupl |
| 645. Lu G-L, Lee MT, Chiou L-C. Orexin-mediated restoration of hippocampal synaptic potentiation in mice with established cocaine-conditioned place preference. Addict Biol. 2019;24(6):1153-1166. doi: 10.1111/adb.12672. Epub 2018 Oct 1. | Dupl |
| 646. Norozpour Y, Zarrabian S, Rezaee L, Haghparast A. D1- and D2-like receptors in the dentate gyrus region of the hippocampus are involved in the reinstatement induced by a subthreshold dose of morphine and forced swim stress in extinguished morphine-CPP in rats. Behav Neurosci. 2019;133(6):545-555. doi: 10.1037/bne0000335. | Animal |
| 647. Hatta K. Delirium. In Chopra A, Das P, Doghramji K (eds.). Management of Sleep Disorders in Psychiatry. Oxford (UK): Oxford University Press, 2020, pp. 402-411. https://doi.org/10.1093/med/9780190929671.001.0001. https://doi.org/10.1093/med/9780190929671.003.0024. | Review |
| 648. Chamera K, Trojan E, Szuster-Głuszczak M, Basta-Kaim A. The potential role of dysfunctions in neuron-microglia communication in the pathogenesis of brain disorders. Curr Neuropharmacol. 2020;18(5):408-430. doi: 10.2174/1570159X17666191113101629. | Dupl |
| 649. Feketeova E, Tormasiova M, Klobučníková K, Durdik P, Jarcuskova D, Benca M, Vitkova M. Narcolepsy in Slovakia - Epidemiology, clinical and polysomnographic features, comorbid diagnoses: a case-control study. Sleep Med. 2020;67:15-22. doi: 10.1016/j.sleep.2019.10.012. Epub 2019 Nov 11. | Dupl |
| 650. Choi MR, Cho H, Chun JW, Yoo JH, Kim DJ. Increase of orexin A in the peripheral blood of adolescents with Internet gaming disorder. J Behav Addict. 2020;9(1):93-104. doi: 10.1556/2006.8.2019.65. | No psychosis |
| 651. Rezaee L, Alizadeh AM, Haghparast A. Role of hippocampal dopamine receptors in the antinociceptive responses induced by chemical stimulation of the lateral hypothalamus in animal model of acute pain. Brain Res. 2020;1734:146759. doi: 10.1016/j.brainres.2020.146759. Epub 2020 Mar 2. | Dupl |
| 652. Akça ÖF, Uzun N, Kılınç İ. Orexin A in adolescents with anxiety disorders. Int J Psychiatry Clin Pract. 2020;24(2):127-134. doi: 10.1080/13651501.2019.1711425. | No psychosis |
| 653. Lis M, Stańczykiewicz B, Liśkiewicz P, Misiak B. Impaired hormonal regulation of appetite in schizophrenia: A narrative review dissecting intrinsic mechanisms and the effects of antipsychotics. Psychoneuroendocrinology. 2020;119:104744. doi: 10.1016/j.psyneuen.2020.104744. Epub 2020 Jun 5. | Dupl |
| 654. Liu Z, Zhang Y, Zhao T, Wang J, Xia L, Zhong Y, Yang Y, Ning X, Zhang Y, Ren Z, Liu H. A higher body mass index in Chinese inpatients with chronic schizophrenia is associated with elevated plasma orexin-A levels and fewer negative symptoms. Nord J Psychiatry. 2020;74(7):525-532. doi: 10.1080/08039488.2020.1755995. Epub 2020 May 4. | Dupl |
| 655. Rosenblat, Joshua D. Novel therapeutic drug targets for bipolar disorder. In: Quevedo J, Ferrer Carvalho A, Vieta E (eds.) Neurobiology of Bipolar Disorder: Road to Novel Therapeutics. London (UK): Academic Press-Elsevier, 2021, pp. 393-404. https://doi.org/10.1016/B978-0-12-819182-8.00037-5. | Review |
| 656. Procyshyn RM, Bezchlibnyk-Butler KZ, Jeffries JJ (Eds.). Clinical Handbook of Psychotropic Drugs, 24th ed. Newburyport, MA: Hogrefe, 2021. https://doi.org/10.1027/00593-000. | Review |
| 657. Khaleghzadeh-Ahangar H, Rashvand M, Haghparast A. Role of D1- and D2-like dopamine receptors within the dentate gyrus in antinociception induced by chemical stimulation of the lateral hypothalamus in an animal model of acute pain. Physiol Behav. 2021;229:113214. doi: 10.1016/j.physbeh.2020.113214. Epub 2020 Oct 20. | Dupl |
| 658. Durairaja A, Fendt M. Orexin deficiency modulates cognitive flexibility in a sex-dependent manner. Genes Brain Behav. 2021;20(3):e12707. doi: 10.1111/gbb.12707. Epub 2020 Nov 3. | Dupl |
| 659. Perez SM, Lodge DJ. Orexin modulation of VTA dopamine neuron activity: Relevance to schizophrenia. Int J Neuropsychopharmacol. 2021;24(4):344-353. doi: 10.1093/ijnp/pyaa080. | Dupl |
| 660. Yoon JH, Suchting R, Cassidy RN, Bolin PK, Omar Y, Brown GS, De La Garza R. Assessment of demand for methamphetamine and cigarettes among individuals with methamphetamine use disorder. Exp Clin Psychopharmacol. 2021;29(4):334-344. doi: 10.1037/pha0000367. | No psychosis |
| 661. Charntikov S, Pittenger ST, Swalve N, Barrett ST, Bevins RA. Conditioned enhancement of the nicotine reinforcer. Exp Clin Psychopharmacol. 2021;29(4):385-394. doi: 10.1037/pha0000370. | Animal |
| 662. Lu J, Huang ML, Li JH, Jin KY, Li HM, Mou TT, Fronczek R, Duan JF, Xu WJ, Swaab D, Bao AM. Changes of hypocretin (Orexin) system in schizophrenia: From plasma to brain. Schizophr Bull. 2021;47(5):1310-1319. doi: 10.1093/schbul/sbab042. | Dupl |
| 663. Akça ÖF, Sağlam E, Kılınç I, Bilgiç A. Orexin A levels of adolescents with major depressive disorder. Int J Psychiatry Clin Pract. 2021;25(4):403-406. doi: 10.1080/13651501.2021.1927106. | No psychosis |
| 664. Lee W-C, Chen P-Y, Kao C-F, Huang M-C. Differences in serum orexin-A levels between the acute and subacute withdrawal phases in individuals who use methamphetamine. Exp Clin Psychopharmacol. 2021;29(6):573-579. doi: 10.1037/pha0000395. | No psychosis |
| 665. Terada T, Hirayama T, Sadahiro R, Wada S, Nakahara R, Matsuoka H. Pilot study of lemborexant for insomnia in cancer patients with delirium. J Palliat Med. 2022;25(5):797-801. doi: 10.1089/jpm.2021.0509. Epub 2022 Jan 28. | Dupl |
| 666. Li S, Zhang R, Hu S, Lai J. Plasma orexin-A levels in patients with schizophrenia: A systematic review and meta-analysis. Front Psychiatry. 2022;13:879414. doi: 10.3389/fpsyt.2022.879414. | Dupl |
| 667. Demidova A, Kahl E, Fendt M. Orexin deficiency affects sensorimotor gating and its amphetamine-induced impairment. Prog Neuropsychopharmacol Biol Psychiatry. 2022;116:110517. doi: 10.1016/j.pnpbp.2022.110517. Epub 2022 Jan 29. | Dupl |
| 668. Brown RE, Spratt TJ, Kaplan GB. Translational approaches to influence sleep and arousal. Brain Res Bull. 2022;185:140-161. doi: 10.1016/j.brainresbull.2022.05.002. Epub 2022 May 10. | Dupl |

| | |
|---|---|
| 669. Palagini L, Hertenstein E, Riemann D, Nissen C. Sleep, insomnia and mental health. J Sleep Res. 2022;31(4):e13628. doi: 10.1111/jsr.13628. Epub 2022 May 4. | Dupl |
| 670. González-Blanco L, Moya-Lacasa C, Jiménez-Fernández S, Martínez-Cao C, Valtueña-García M, Dal Santo F, García-Portilla MP, Luis Gutiérrez-Rojas L. Endocrine biomarkers related to sleep-wake cycle and sleep disturbances in patients with bipolar disorder: A systematic review. Eur J Psychiatry. 2022;36(4):223-229. DOI: 10.1016/j.ejpsy.2022.04.004. | Review |
| 671. Bergamini G, Coloma P, Massinet H, Steiner MA. What evidence is there for implicating the brain orexin system in neuropsychiatric symptoms in dementia? Front Psychiatry. 2022;13:1052233. doi: 10.3389/fpsyt.2022.1052233. | Dupl |
| 672. Smith DM, Terhune DB. Pedunculopontine-induced cortical decoupling as the neurophysiological locus of dissociation. Psychol Rev. 2023;130(1):183-210. doi: 10.1037/rev0000353. | Review |
| 673. Seugnet L, Anaclet C, Perier M, Ghersi-Egea JF, Lin JS. A marked enhancement of a BLOC-1 gene, pallidin, associated with somnolent mouse models deficient in histamine transmission. CNS Neurosci Ther. 2023;29(1):483-486. doi: 10.1111/cns.13995. | Opinion |
| 674. Pintwala, Sara Katherine. Transplanting immortal orexin cells in narcoleptic mice rescues cataplexy. PhD Thesis, University of Toronto, Dissertation Abstracts International: Section B: The Sciences and Engineering, 2023, volume 84, issue 5-BAPA. | Animal |
| 675. Finkenberg DB, Shalev H, Vannikov-Lugassi M, Soffer-Dudek N. No evidence for selective serotonin reuptake inhibitors (SSRIs) use as explaining the relationship between sleep experiences and psychopathological distress. Psychol Conscious (Wash D C). 2023;10(1):50–61. https://doi.org/10.1037/cns0000275. | No psychosis |
| 676. Yu H, Ni P, Zhao L, Tian Y, Li M, Li X, Wei W, Wei J, Deng W, Du X, Wang Q, Guo W, Ma X, Coid J, Li T. Decreased plasma neuropeptides in first-episode schizophrenia, bipolar disorder, major depressive disorder: associations with clinical symptoms and cognitive function. Front Psychiatry. 2023;14:1180720. doi: 10.3389/fpsyt.2023.1180720. | Dupl |
| 677. Fagan HA, Baldwin DS. Pharmacological treatment of generalised anxiety disorder: Current practice and future directions. Expert Rev Neurother. 2023;23(6):535-548. doi: 10.1080/14737175.2023.2211767. Epub 2023 May 15. | Dupl |
| 678. Maness EB, Blumenthal SA, Burk JA. Dual orexin/hypocretin receptor antagonism attenuates NMDA receptor hypofunction-induced attentional impairments in a rat model of schizophrenia. Behav Brain Res. 2023;450:114497. doi: 10.1016/j.bbr.2023.114497. Epub 2023 May 16. | Dupl |
| 679. Webber HE, Yoon JH, de Dios C, Suchting R, Dang V, Versace F, Green CE, Wardle MC, Lane SD, Schmitz JM. Assessing cocaine motivational value: Comparison of brain reactivity bias toward cocaine cues and cocaine demand. Exp Clin Psychopharmacol. 2023;31(4):861-867. doi: 10.1037/pha0000622. | No psychosis |
| 680. Nakamura T, Yoshizawa T, Toya R, Terasawa M, Takahashi K, Kitazawa K, Suzuki K, Sasayama D, Washizuka S. Orexin receptor antagonists versus antipsychotics for the management of delirium in intensive care unit patients with cardiovascular disease: A retrospective observational study. Gen Hosp Psychiatry. 2023;84:96-101. doi: 10.1016/j.genhosppsych.2023.06.019. Epub 2023 Jul 1. | Dupl |
| 681. Hansen BH, Andresen HN, Gjesvik J, Thorsby PM, Naerland T, Knudsen-Heier S. Associations between psychiatric comorbid disorders and executive dysfunctions in hypocretin-1 deficient pediatric narcolepsy type1. Sleep Med. 2023;109:149-157. doi: 10.1016/j.sleep.2023.06.021. Epub 2023 Jul 1. | Dupl |
| 682. Riemann D, Espie CA, Altena E, Arnardottir ES, Baglioni C, Bassetti CLA, Bastien C, Berzina N, Bjorvatn B, Dikeos D, Dolenc Groselj L, Ellis JG, Garcia-Borreguero D, Geoffroy PA, Gjerstad M, Gonçalves M, Hertenstein E, Hoedlmoser K, Hion T, Holzinger B, Janku K, Jansson-Fröjmark M, Järnefelt H, Jernelöv S, Jennum PJ, Khachatryan S, Krone L, Kyle SD, Lancee J, Leger D, Lupusor A, Marques DR, Nissen C, Palagini L, Paunio T, Perogamvros L, Pevernagie D, Schabus M, Shochat T, Szentkiralyi A, Van Someren E, van Straten A, Wichniak A, Verbraecken J, Spiegelhalder K. The European Insomnia Guideline: An update on the diagnosis and treatment of insomnia 2023. J Sleep Res. 2023;32(6):e14035. doi: 10.1111/jsr.14035. | Dupl |
| 683. Lafrenière S, Blier P. Remission from severe treatment-resistant depression with moclobemide: A case report. Encéphale. 2023;49(6):649-650. doi: 10.1016/j.encep.2023.01.014. | Case |
| 684. Mishima K. Pharmacologic treatment of insomnia. In: Tasman A, Riba MB, Alarcón RD, Alfonso CA, Kanba S, Lecic-Tosevski D, Ndetei DM, Ng CH, Schulze TG (eds.) Tasman's Psychiatry. Cham, CH: Springer 2024, pp. 4281-4302, https://doi.org/10.1007/978-3-030-51366-5_137. | Review |
| 685. Pillai, M., Chandrasekhara, S. (2024). Insomnia. In: Tampi RR, Tampi DJ (eds) Treatment of Psychiatric Disorders Among Older Adults. Cham, CH: Springer, pp. 153-171. https://doi.org/10.1007/978-3-031-55711-8_11. | Review |
| 686. Chen P-Y, Chiu C-C, Chang C-K, Lu M-L, Huang C-Y, Chen C-H, Huang M-C. Higher orexin-A levels are associated with treatment response to clozapine in patients with schizophrenia: A cross-sectional study. J Psychopharmacol. 2024;38(3):258-267. doi: 10.1177/02698811231225610. Epub 2024 Jan 27. | Dupl |
| 687. DeGutis J, Sullivan DR, Agnoli S, Stumps A, Logue M, Brown E, Verfaellie M, Milberg W, McGlinchey R, Esterman M. Less is more: Smaller hippocampal subfield volumes predict greater improvements in posttraumatic stress disorder symptoms over 2 years. Behav Neurosci. 2024;138(2):94-107. doi: 10.1037/bne0000578. | No orexin |
| 688. Liu X, Lan X, Zhang X, Ye H, Shen L, Hu M, Chen X, Zheng M, Weston-Green K, Jin T, Cui X, Zhou Y, Lu X, Huang XF, Yu Y. Olanzapine attenuates 5-HT2cR and GHSR1a interaction to increase orexigenic hypothalamic NPY: Implications for neuronal molecular mechanism of metabolic side effects of antipsychotics. Behav Brain Res. 2024;463:114885. doi: 10.1016/j.bbr.2024.114885. | Animal |
| 689. Öz P, Kamalı O, Saka HB, Gör C, Uzbay İT. Baseline prepulse inhibition dependency of orexin A and REM sleep deprivation. Psychopharmacology (Berl). 2024;241(6):1213-1225. doi: 10.1007/s00213-024-06555-3. Epub 2024 Mar 1. | Dupl |
| 690. Felix PC, Flagel SB. Leveraging individual differences in cue-reward learning to investigate the psychological and neural basis of shared psychiatric symptomatology: The sign-tracker/goal-tracker model. Behav Neurosci. 2024;138(4):260-271. doi: 10.1037/bne0000590. | Review |
| 691. Kikuchi Y, Kurosawa M, Sakata M, Takahashi Y, Yamamoto K, Tomita H, Yoshio T, Yasui-Furukori N. Effects of titration speed, gender, obesity and concomitant medications on the risk and onset time of clozapine-associated fever among Japanese patients with schizophrenia: retrospective review of charts from 21 hospitals. Br J Psychiatry. 2024;225(5):492-498. doi: 10.1192/bjp.2024.113. | Dupl |
| 692. Geldmacher DS. Treatment of Alzheimer disease. Continuum (Minneap Minn). 2024;30(6):1823-1844. doi: 10.1212/CON.0000000000001503. | Dupl |
| 693. Hur J, Tillman RM, Kim HC, Didier P, Anderson AS, Islam S, Stockbridge MD, De Los Reyes A, DeYoung KA, Smith JF, Shackman AJ. Adolescent social anxiety is associated with diminished discrimination of anticipated threat and safety in the bed nucleus of the stria terminalis. J Psychopathol Clin Sci. 2025;134(1):41-56. doi: 10.1037/abn0000940. Epub 2024 Nov 7. | No psychosis |
| 694. Spiegelhalder K, Nissen C, Riemann D. The impact of prescription and recreational drugs on sleep. In Espie CA, Zee PC, Morin CM (eds.) The Oxford Handbook of Sleep and Sleep Disorders., 2nd ed. Oxford (UK): Oxford University Press, 2025, pp. 458-477. https://doi.org/10.1093/oxfordhb/9780197602751.013.10. | Review |
| 695. Havlik JL, Rhee TG, Rosenheck RA. Correlates of impaired sleep duration among adults with trauma alone and with posttraumatic stress disorder. Psychol Trauma. 2025. doi: 10.1037/tra0001846. Epub ahead of print 2025 Jan 20. | No orexin |
| 696. Barrett ST, McNealy KR, Knabel ML, Burrichter RM, Steck KA, Bevins RA. The inside story: Interoceptive Pavlovian conditioning with the nicotine stimulus. J Exp Psychol Anim Learn Cogn. 2025;51(2):61-72. doi: 10.1037/xan0000393. | Review |
| 697. Chekani F, Mirchandani K, Zaki S, Goswami S, Sharma M. Utilization of potentially inappropriate sedative-hypnotic and atypical antipsychotic medications among elderly individuals with insomnia and Alzheimer's disease. Sleep. 2025;48(4):zsaf003. doi: 10.1093/sleep/zsaf003. | Dupl |
| 698. Dumont S, Bloch V, Lillo-Lelouet A, Le Beller C, Geoffroy PA, Veyrier M. Parasomnias and sleep-related movement disorders induced by drugs in the adult population: a review about iatrogenic medication effects. J Sleep Res. 2025;34(2):e14306. doi: 10.1111/jsr.14306. Epub 2024 Sep 7. | Dupl |

| | |
|---|---|
| 699. Regnier SD, Chirica MG, Acuff SF, Strickland JC. Toward better outcomes: Evolving psychopharmacologic approaches in youth psychiatry. Transl Issues Psychol Sci. 2025;11(3):277-280. https://doi.org/10.1037/tps0000472. | Review |
| 700. Torres C, Papini MR, Huffman MA. Psychological self-medication in mammals: A dialogue between natural observations and laboratory research. J Comp Psychol. 2025. doi: 10.1037/com0000428. Epub ahead of print 2025 Sep 15. | Animal |
| 701. Funayama M, Sato K, Nakagawa Y. Thermoregulatory dysfunction in the chronic phase of cerebrovascular disease: Hypothalamus damage and behavior control deficits. Neuropsychology. 2025. doi: 10.1037/neu0001046. Epub ahead of print 2025 Oct 16. | Unfocused |
| 702. Barrett ST, McNealy KR, Knabel ML, Burrichter RM, Steck KA, Bevins RA. The inside story: Interoceptive Pavlovian conditioning with the nicotine stimulus. J Exp Psychol Anim Learn Cogn. 2025;51(2):61-72. doi: 10.1037/xan0000393. | Dupl |
| 703. Dumont S, Bloch V, Lillo-Lelouet A, Le Beller C, Geoffroy PA, Veyrier M. Parasomnias and sleep-related movement disorders induced by drugs in the adult population: a review about iatrogenic medication effects. J Sleep Res. 2025;34(2):e14306. doi: 10.1111/jsr.14306. Epub 2024 Sep 7. | Review |
| 704. Baskerville W-A, Grodin EN, Meredith LR, Ray LA. Interplay between alcohol cues and mood states during early abstinence: A daily diary study. Exp Clin Psychopharmacol. 2025;33(3):260-268. doi: 10.1037/pha0000770. Epub 2025 Mar 13. | No orexin |
| 705. Kawashima H, Waddington JL, Saigusa T. Reduced accumbal dopamine efflux via orexin OX2 receptors in chronic pain models. Eur J Neurosci. 2025;62(1):e70192. doi: 10.1111/ejn.70192. | Animal |
| 706. Garcia-Borreguero D, Anguizola D, Carvallo C, Lopez A, Garcia Aragón A, Moncada B, Ferré S. Subclinical Augmentation in Relation to Previous Dopaminergic Treatment in Patients with Restless Legs Syndrome: A Post Hoc Analysis of Two Randomized, Placebo-Controlled, Crossover Trials. CNS Drugs. 2025;39(8):779-793. doi: 10.1007/s40263-025-01192-6. Epub 2025 Jun 4. | No psychosis |
| 707. Clark PJ, Migovich VM, Das S, Xu W, Zhang Y, Kortagere S, España RA. Hypocretin receptor 1 blockade early in abstinence prevents incubation of cocaine seeking and normalizes dopamine transmission. Neuropsychopharmacology. 2026. doi: 10.1038/s41386-025-02315-9. Epub ahead of print 2026 Feb 4. | Animal |
| 708. Monda M, Viggiano A, Viggiano A, Viggiano E, De Luca V. Risperidone potentiates the sympathetic and hyperthermic reactions induced by orexin A in the rat. Physiol Res. 2006;55(1):73-78. doi: 10.33549/physiolres.930906. Epub 2005 Apr 26. | Dupl |
| 709. Stefanidis A, Verty AN, Allen AM, Owens NC, Cowley MA, Oldfield BJ. The role of thermogenesis in antipsychotic drug-induced weight gain. Obesity (Silver Spring). 2009;17(1):16-24. doi: 10.1038/oby.2008.468. Epub 2008 Oct 30. | Dupl |
| 710. Burgess CR, Tse G, Gillis L, Peever JH. Dopaminergic regulation of sleep and cataplexy in a murine model of narcolepsy. Sleep. 2010;33(10):1295-304. doi: 10.1093/sleep/33.10.1295. | Dupl |
| 711. Li X, Johnson MS, Smith DL Jr, Li Y, Kesterson RA, Allison DB, Nagy TR. Effects of risperidone on energy balance in female C57BL/6J mice. Obesity (Silver Spring). 2013;21(9):1850-7. doi: 10.1002/oby.20350. Epub 2013 May 29. | Dupl |
| 712. Pizza F, Magnani M, Indrio C, Plazzi G. The hypocretin system and psychiatric disorders. Curr Psychiatry Rep. 2014;16(2):433. doi: 10.1007/s11920-013-0433-9. | Dupl |
| 713. Hasegawa E, Yanagisawa M, Sakurai T, Mieda M. Orexin neurons suppress narcolepsy via 2 distinct efferent pathways. J Clin Invest. 2014;124(2):604-16. doi: 10.1172/JCI71017. Epub 2014 Jan 2. | Dupl |
| 714. Chen YW, Morganstern I, Barson JR, Hoebel BG, Leibowitz SF. Differential role of D1 and D2 receptors in the perifornical lateral hypothalamus in controlling ethanol drinking and food intake: possible interaction with local orexin neurons. Alcohol Clin Exp Res. 2014;38(3):777-86. doi: 10.1111/acer.12313. Epub 2013 Nov 15. | Dupl |
| 715. Chen YW, Morganstern I, Barson JR, Hoebel BG, Leibowitz SF. Differential role of D1 and D2 receptors in the perifornical lateral hypothalamus in controlling ethanol drinking and food intake: possible interaction with local orexin neurons. Alcohol Clin Exp Res. 2014;38(3):777-86. doi: 10.1111/acer.12313. Epub 2013 Nov 15. | Dupl |
| 716. Szabo ST, Kinon BJ, Brannan SK, Krystal AK, van Gerven JM, Mahableshwarkar A, Sachs GS. Lessons learned and potentials for improvement in CNS drug development: ISCTM Section on Designing the right series of experiments. Innov Clin Neurosci. 2015;12(3Suppl A):11S-25S. | Dupl |
| 717. Barandas R, Landgraf D, McCarthy MJ, Welsh DK. Circadian Clocks as Modulators of Metabolic Comorbidity in Psychiatric Disorders. Curr Psychiatry Rep. 2015;17(12):98. doi: 10.1007/s11920-015-0637-2. | Dupl |
| 718. Vickers SP, Hackett D, Murray F, Hutson PH, Heal DJ. Effects of lisdexamfetamine in a rat model of binge-eating. J Psychopharmacol. 2015;29(12):1290-307. doi: 10.1177/0269881115615107. Epub 2015 Nov 20. | Dupl |
| 719. Nakamura M, Nagamine T. Neuroendocrine, autonomic, and metabolic responses to an orexin antagonist, suvorexant, in psychiatric patients with insomnia. Innov Clin Neurosci. 2017;14(3-4):30-37. | Lumping |
| 720. Matheson E, Hainer BL. Insomnia: Pharmacologic Therapy. Am Fam Physician. 2017;96(1):29-35. | Opinion |
| 721. Keks NA, Hope J, Keogh S. Suvorexant: scientifically interesting, utility uncertain. Australas Psychiatry. 2017;25(6):622-624. doi: 10.1177/1039856217734677. Epub 2017 Oct 10. | Dupl |
| 722. McElroy SL, Guerdjikova AI, Mori N, Romo-Nava F. Progress in Developing Pharmacologic Agents to Treat Bulimia Nervosa. CNS Drugs. 2019;33(1):31-46. doi: 10.1007/s40263-018-0594-5. | Dupl |
| 723. Ni P, Tian Y, Gu X, Yang L, Wei J, Wang Y, Zhao L, Zhang Y, Zhang C, Li L, Tang X, Ma X, Hu X, Li T. Plasma neuropeptides as circulating biomarkers of multifactorial schizophrenia. Compr Psychiatry. 2019;94:152114. doi: 10.1016/j.comppsych.2019.152114. Epub 2019 Aug 5. | Dupl |
| 724. Lu G-L, Lee MT, Chiou L-C. Orexin-mediated restoration of hippocampal synaptic potentiation in mice with established cocaine-conditioned place preference. Addict Biol. 2019;24(6):1153-1166. doi: 10.1111/adb.12672. Epub 2018 Oct 1. | Dupl |
| 725. BaHammam AS, Alnakshabandi K, Pandi-Perumal SR. Neuropsychiatric Correlates of Narcolepsy. Curr Psychiatry Rep. 2020;22(8):36. doi: 10.1007/s11920-020-01159-y. | Dupl |
| 726. Lu J, Huang ML, Li JH, Jin KY, Li HM, Mou TT, Fronczek R, Duan JF, Xu WJ, Swaab D, Bao AM. Changes of hypocretin (Orexin) system in schizophrenia: From plasma to brain. Schizophr Bull. 2021;47(5):1310-1319. doi: 10.1093/schbul/sbab042. | Dupl |
| 727. Porwal A, Yadav YC, Pathak K, Yadav R. An update on assessment, therapeutic management, and patents on insomnia. Biomed Res Int. 2021;2021:6068952. doi: 10.1155/2021/6068952. | Dupl |
| 728. Terada T, Hirayama T, Sadahiro R, Wada S, Nakahara R, Matsuoka H. Pilot study of lemborexant for insomnia in cancer patients with delirium. J Palliat Med. 2022;25(5):797-801. doi: 10.1089/jpm.2021.0509. Epub 2022 Jan 28. | Dupl |
| 729. Chen PY, Chang CK, Chen CH, Fang SC, Mondelli V, Chiu CC, Lu ML, Hwang LL, Huang MC. Orexin-a elevation in antipsychotic-treated compared to drug-free patients with schizophrenia: A medication effect independent of metabolic syndrome. J Formos Med Assoc. 2022;121(11):2172-2181. doi: 10.1016/j.jfma.2022.03.008. Epub 2022 Apr 6. | Dupl |
| 730. Matsuoka A, Sogawa R, Murakawa-Hirachi T, Mizoguchi Y, Monji A, Shimanoe C, Shinada K, Koami H, Sakamoto Y. Evaluation of the delirium preventive effect of dual orexin receptor antagonist (DORA) in critically ill adult patients requiring ventilation with tracheal intubation at an advanced emergency center: A single-center, retrospective, observational study. Gen Hosp Psychiatry. 2023;83:123-129. doi: 10.1016/j.genhosppsych.2023.03.010. Epub 2023 Mar 31. | Unfocused |
| 731. Matsumoto S, Tamiya H, Yamana H, Hosoi T, Matsui H, Fushimi K, Akishita M, Yasunaga H, Ogawa S. Association between the type of hypnotic drug and in-hospital fractures in older patients with neurocognitive disorders: A case-control study using a nationwide database. Geriatr Gerontol Int. 2023;23(7):500-505. doi: 10.1111/ggi.14600. Epub 2023 May 22. | Unfocused |

| | |
|---|---|
| 732. Nakamura T, Yoshizawa T, Toya R, Terasawa M, Takahashi K, Kitazawa K, Suzuki K, Sasayama D, Washizuka S. Orexin receptor antagonists versus antipsychotics for the management of delirium in intensive care unit patients with cardiovascular disease: A retrospective observational study. Gen Hosp Psychiatry. 2023;84:96-101. doi: 10.1016/j.genhosppsych.2023.06.019. Epub 2023 Jul 1. | Dupl |
| 733. Chen P-Y, Chiu C-C, Chang C-K, Lu M-L, Huang C-Y, Chen C-H, Huang M-C. Higher orexin-A levels are associated with treatment response to clozapine in patients with schizophrenia: A cross-sectional study. J Psychopharmacol. 2024;38(3):258-267. doi: 10.1177/02698811231225610. Epub 2024 Jan 27. | Dupl |
| 734. Öz P, Kamalı O, Saka HB, Gör C, Uzbay İT. Baseline prepulse inhibition dependency of orexin A and REM sleep deprivation. Psychopharmacology (Berl). 2024;241(6):1213-1225. doi: 10.1007/s00213-024-06555-3. Epub 2024 Mar 1. | Dupl |
| 735. Takeshima M, Sakurai H, Inada K, Aoki Y, Ie K, Kise M, Yoshida E, Matsui K, Utsumi T, Shimura A, Okajima I, Kotorii N, Yamashita H, Suzuki M, Kuriyama K, Shimizu E, Mishima K, Watanabe K, Takaesu Y. Treatment strategies for insomnia in Japanese primary care physicians' practice: A Web-based questionnaire survey. BMC Prim Care. 2024;25(1):219. doi: 10.1186/s12875-024-02449-7. | Unrelated |
| 736. Baum E, Philipp M, Spiegelhalder K. Insomnie – Update der S3-Leitlinie. Relevantes für den hausärztlichen Bereich [Insomnia—update of the S3 guideline. With relevance for general practice]. ZFA Z Allg Med (Stuttgart). 2025;101(6):303-307. doi: 10.1007/s44266-025-00387-w. | Review |
| 737. Seltorexant improved sleep outcomes in patients with insomnia disorder. Brown University Psychopharmacology Update, 2025;36(12):6-7. doi: 10.1002/pu.31383 (refers to Mesens S, Krystal AD, Melkote R, Xu H, Pandina G, Saoud JB, Luthringer R, Savitz A, Drevets WC. Efficacy and safety of seltorexant in insomnia disorder: A randomized clinical trial. JAMA Psychiatry. 2025;82(10):967-76. doi: 10.1001/jamapsychiatry.2025.1999. Epub ahead of print. No psychosis). | Opinion |

| | | |
|---|---|---|
| Included | | 20 |
| Excluded | | 717 |
| | Animal | 173 |
| | Reviews | 163 |
| | No orexin | 53 |
| | Opinions | 28 |
| | Case reports and series | 26 |
| | Unfocused | 25 |
| | No psychosis | 23 |
| | Unrelated | 22 |
| | *In vitro* | 11 |
| | Protocols | 5 |
| | Abstracts (congress) | 4 |
| | *Post-mortem* | 3 |
| | No antipsychotics | 3 |
| | Lumping | 1 |
| | Retracted | 1 |
| | Duplicates | 176 |

## Table S2. Counts of studies and samples

### Country (N = 20 studies)

- Taiwan: n = 5 (25.0%)
- China: n = 4 (20.0%)
- Japan: n = 4 (20.0%)
- Multicentre: n = 2 (10.0%)
- United States: n = 1 (5.0%)
- Germany: n = 1 (5.0%)
- France: n = 1 (5.0%)
- Türkiye: n = 1 (5.0%)
- Spain: n = 1 (5.0%)

### Type of study (N = 20 studies)

- Cross-sectional: n = 9 (45.0%)
- Case–control (incl. cross-sectional case–control): n = 5 (25.0%)
- Longitudinal: n = 4 (20.0%)
- Observational (unspecified): n = 2 (10.0%)

### Antipsychotics used (counts per study, N = 20)
### *(not mutually exclusive categories)*

- Clozapine: n = 11 (55.0%)
- Haloperidol: n = 8 (40.0%)
- Olanzapine: n = 6 (30.0%)
- Other antipsychotics (conventional neuroleptics, other SGAs, aripiprazole, mixed treatments): n = 10 (50.0%)
- Antipsychotics not used / drug-free patients / not applicable:
  n = 2 (10.0%)
- Unspecified antipsychotic: n = 4 (20.0%)

### Sample (N = 3.495 participants)

- Total number of participants: N = 3.495
    - Of whom 56 from *post-mortem* samples (1.6%)
- Schizophrenia / psychotic spectrum: N = 2.642 (75.6% of the total sample)
- First-episode psychosis (FEP): N = 150 (5.7% of schizophrenia/spectrum patients)

- Major Depressive Disorder (MDD): N = 80 (2.3%)
- Bipolar Disorder (BD): N = 40 (1.1%)
- Neurological patients (Guillain-Barré syndrome with psychotic symptoms): N = 139 (4.0%)
- Neurological controls without Guillain-Barré syndrome (from Intensive Care Units): N = 55 (1.6%)
- Healthy controls (HC): N = 657 (18.8%)

**Orexin measurement method (N = 20 studies)**

**Type of measurement**

***(not mutually exclusive categories)***

- Orexin-A / Hypocretin-1 (peptide): n = 16 (80.0%)
- Orexin-B / "total orexin": n = 1 (5.0%)
- Orexin receptors (HcrtR1/HcrtR2; genetics or mRNA): n = 5 (25.0%)
- Genetic polymorphisms (Hcrt / HcrtR1 / HcrtR2): n = 4 (20.0%)
- mRNA expression (HcrtR1/HcrtR2): n = 1 (5.0%)
- *Post-mortem* Immunoreactivity: n = 1 (5.0%)

**Biological compartment (N = 20 studies)**

***(not mutually exclusive categories)***

- Cerebrospinal fluid (CSF): n = 8 (40.0%)
- Plasma / serum / peripheral blood: n = 11 (55.0%)
- Post-mortem brain tissue: n = 1 (5.0%)
- Genetics (peripheral organ DNA): n = 5 (25.0%)

**Analytical methods (N = 20 studies)**

***(not mutually exclusive categories)***

- Radioimmunoassay (RIA): n = 7 (35.0%)
- ELISA: n = 8 (40.0%)
- Multiplex immunoassay: n = 1 (5.0%)
- N/A –genetic studies with no peptide assay: n = 4 (20.0%)

**Direction of antipsychotic-related effects**

***(only studies with modulation; N = 12 studies)***

- Antipsychotic treatment-associated orexin reduction: n = 7 (58.3%)
- Orexin increase in specific contexts (for example, clozapine-responders): n = 3 (25.0%)
- Circadian modulation with no variations of mean levels: n = 2 (16.7%)

**Association with psychotic symptom severity (N = 20 studies)**

- No overall association with PANSS/BPRS scores: n = 12 (60.0%)
- Favourable associations in specific subgroups: n = 5 (25.0%)
- Association not assessed / not reported: n = 3 (15.0%)

**Metabolic and behavioural associations (N = 20 studies)**

- Associations with metabolic parameters: n = 8 (40.0%)
- Associations with sleep–wake regulation / physical activity: n = 5 (25.0%)
- No metabolic or behavioural association reported: n = 7 (35.0%)

**Table S3. Risk of bias of included studies according to the Robins-E tool (Higgins et al., 2024) [19]**

- Domain 1: Risk of bias due to confounding
- Domain 2: Risk of bias in classification of interventions
- Domain 3: Risk of bias in selection of participants into the study (or into the analysis)
- Domain 4: Risk of bias due to deviations from intended interventions
- Domain 5: Risk of bias due to missing data
- Domain 6: Risk of bias arising from measurement of the outcome
- Domain 7: Risk of bias in selection of the reported result
- Total Risk: Low, Moderate, Serious, Critical

| Judgement | Interpretation |
|---|---|
| *Low risk of bias** | There is little or no concern about bias with regard to this domain. |
| *Moderate risk of bias* | There is some concern about bias with regard to this domain, although it is not clear that there is an important risk of bias. |
| *Serious risk of bias* | The study has some important problems in this domain: characteristics of the study give rise to a serious risk of bias. |
| *Critical risk of bias* | The study is very problematic in this domain: characteristics of the study give rise to a critical risk of bias, such that and the result should generally be excluded from evidence syntheses. |

| Study | Confounding | Interventions | Participant selection | Deviations | Missing data | Outcome measurement | Selection of report | Overall |
|---|---|---|---|---|---|---|---|---|
| Nishino et al., 2002 | | | | | | | | |
| Dalal et al., 2003 | | | | | | | | |
| Meerabux et al., 2005 | | | | | | | | |
| Cochen et al., 2005 | | | | | | | | |
| Fukunaka et al., 2007 | | | | | | | | |
| Basoglu et al., 2010 | | | | | | | | |

| | | | | | | | | |
|---|---|---|---|---|---|---|---|---|
| Huang et al., 2014 | | | | | | | | |
| Chien et al., 2015 | | | | | | | | |
| Sun et al., 2016 | | | | | | | | |
| Tiwari et al., 2016 | | | | | | | | |
| Sansa et al., 2016 | | | | | | | | |
| Tsuchimine et al., 2019 | | | | | | | | |
| Chen et al., 2019 | | | | | | | | |
| Liu et al., 2020 | | | | | | | | |
| Lu et al., 2021 | | | | | | | | |
| Chen et al., 2022 | | | | | | | | |
| Ren et al., 2022 | | | | | | | | |
| Yu et al., 2023 | | | | | | | | |
| Chen et al., 2024 | | | | | | | | |
| Tanaka et al., 2025 | | | | | | | | |

**Reference**

[19] Higgins JPT, Morgan RL, Rooney AA, Taylor KW, Thayer KA, Silva RA, Lemeris C, Akl EA, Bateson TF, Berkman ND, Glenn BS, Hróbjartsson A, LaKind JS, McAleenan A, Meerpohl JJ, Nachman RM, Obbagy JE, O'Connor A, Radke EG, Savović J, Schünemann HJ, Shea B, Tilling K, Verbeek J, Viswanathan M, Sterne JAC. A tool to assess risk of bias in non-randomized follow-up studies of exposure effects (ROBINS-E). Environ Int. 2024;186:108602. doi: 10.1016/j.envint.2024.108602. Epub 2024 Mar 24.

Table S4. PRISMA 2020 Checklist.

## PRISMA 2020 Checklist

| Section and Topic | Item # | Checklist item | Location where item is reported |
|---|---|---|---|
| **TITLE** | | | 1 |
| Title | 1 | Identify the report as a systematic review. | 1 |
| **ABSTRACT** | | | |
| Abstract | 2 | See the PRISMA 2020 for Abstracts checklist. | 1 |
| **INTRODUCTION** | | | 2 |
| Rationale | 3 | Describe the rationale for the review in the context of existing knowledge. | 2-3 |
| Objectives | 4 | Provide an explicit statement of the objective(s) or question(s) the review addresses. | 3 |
| **METHODS** | | | 3-4 |
| Eligibility criteria | 5 | Specify the inclusion and exclusion criteria for the review and how studies were grouped for the syntheses. | 4 |
| Information sources | 6 | Specify all databases, registers, websites, organisations, reference lists and other sources searched or consulted to identify studies. Specify the date when each source was last searched or consulted. | 3-4 |
| Search strategy | 7 | Present the full search strategies for all databases, registers and websites, including any filters and limits used. | 3-4 |
| Selection process | 8 | Specify the methods used to decide whether a study met the inclusion criteria of the review, including how many reviewers screened each record and each report retrieved, whether they worked independently, and if applicable, details of automation tools used in the process. | 3-4 |
| Data collection process | 9 | Specify the methods used to collect data from reports, including how many reviewers collected data from each report, whether they worked independently, any processes for obtaining or confirming data from study investigators, and if applicable, details of automation tools used in the process. | 3-4 |
| Data items | 10a | List and define all outcomes for which data were sought. Specify whether all results that were compatible with each outcome domain in each study were sought (e.g. for all measures, time points, analyses), and if not, the methods used to decide which results to collect. | 3-4 |
| | 10b | List and define all other variables for which data were sought (e.g. participant and intervention characteristics, funding sources). Describe any assumptions made about any missing or unclear information. | 3-4 |
| Study risk of bias assessment | 11 | Specify the methods used to assess risk of bias in the included studies, including details of the tool(s) used, how many reviewers assessed each study and whether they worked independently, and if applicable, details of automation tools used in the process. | 3-4 |
| Effect measures | 12 | Specify for each outcome the effect measure(s) (e.g. risk ratio, mean difference) used in the synthesis or presentation of results. | 3-4 |
| Synthesis methods | 13a | Describe the processes used to decide which studies were eligible for each synthesis (e.g. tabulating the study intervention characteristics and comparing against the planned groups for each synthesis (item #5)). | 3-4 |
| | 13b | Describe any methods required to prepare the data for presentation or synthesis, such as handling of missing summary statistics, or data conversions. | 3-4 |
| | 13c | Describe any methods used to tabulate or visually display results of individual studies and syntheses. | 3-4 |
| | 13d | Describe any methods used to synthesize results and provide a rationale for the choice(s). If | 3-4 |

| Section and Topic | Item # | Checklist item | Location where item is reported |
|---|---|---|---|
| | | meta-analysis was performed, describe the model(s), method(s) to identify the presence and extent of statistical heterogeneity, and software package(s) used. | |
| | 13e | Describe any methods used to explore possible causes of heterogeneity among study results (e.g. subgroup analysis, meta-regression). | 3-4 |
| | 13f | Describe any sensitivity analyses conducted to assess robustness of the synthesized results. | 3-4 |
| Reporting bias assessment | 14 | Describe any methods used to assess risk of bias due to missing results in a synthesis (arising from reporting biases). | 3-4 Suppl |
| Certainty assessment | 15 | Describe any methods used to assess certainty (or confidence) in the body of evidence for an outcome. | 3-4 |
| **RESULTS** | | | 5-14 |
| Study selection | 16a | Describe the results of the search and selection process, from the number of records identified in the search to the number of studies included in the review, ideally using a flow diagram. | 5 |
| | 16b | Cite studies that might appear to meet the inclusion criteria, but which were excluded, and explain why they were excluded. | 6 Suppl |
| Study characteristics | 17 | Cite each included study and present its characteristics. | 5-14 |
| Risk of bias in studies | 18 | Present assessments of risk of bias for each included study. | 5-14 |
| Results of individual studies | 19 | For all outcomes, present, for each study: (a) summary statistics for each group (where appropriate) and (b) an effect estimate and its precision (e.g. confidence/credible interval), ideally using structured tables or plots. | 5-14 |
| Results of syntheses | 20a | For each synthesis, briefly summarise the characteristics and risk of bias among contributing studies. | 5-14 |
| | 20b | Present results of all statistical syntheses conducted. If meta-analysis was done, present for each the summary estimate and its precision (e.g. confidence/credible interval) and measures of statistical heterogeneity. If comparing groups, describe the direction of the effect. | 5-14 |
| | 20c | Present results of all investigations of possible causes of heterogeneity among study results. | 5-14 |
| | 20d | Present results of all sensitivity analyses conducted to assess the robustness of the synthesized results. | 5-14 |
| Reporting biases | 21 | Present assessments of risk of bias due to missing results (arising from reporting biases) for each synthesis assessed. | 5-14 Suppl |
| Certainty of evidence | 22 | Present assessments of certainty (or confidence) in the body of evidence for each outcome assessed. | 5-14 |
| **DISCUSSION** | | | 15-17 |
| Discussion | 23a | Provide a general interpretation of the results in the context of other evidence. | 15-17 |
| | 23b | Discuss any limitations of the evidence included in the review. | 17 |
| | 23c | Discuss any limitations of the review processes used. | 17 |
| | 23d | Discuss implications of the results for practice, policy, and future research. | 16-17 |
| **OTHER INFORMATION** | | | |
| Registration and protocol | 24a | Provide registration information for the review, including register name and registration number, or state that the review was not registered. | 4-5 |
| | 24b | Indicate where the review protocol can be accessed, or state that a protocol was not prepared. | N/A |
| | 24c | Describe and explain any amendments to information provided at registration or in the protocol. | N/A |
| Support | 25 | Describe sources of financial or non-financial support for the review, and the role of the funders or sponsors in the review. | 18 |
| Competing interests | 26 | Declare any competing interests of review authors. | 18 |
| Availability of data, code and other materials | 27 | Report which of the following are publicly available and where they can be found: template data collection forms; data extracted from included studies; data used for all analyses; analytic code; any other materials used in the review. | N/A |

*From:* Page MJ, McKenzie JE, Bossuyt PM, Boutron I, Hoffmann TC, Mulrow CD, et al. The PRISMA 2020 statement: an updated guideline for reporting systematic reviews. BMJ 2021;372:n71. doi: 10.1136/bmj.n71. This work is licensed under CC BY 4.0. To view a copy of this license, visit https://creativecommons.org/licenses/by/4.0/
